# Dentists' knowledge, skills, attitudes and barriers towards minimal intervention dentistry

**DOI:** 10.1590/1807-3107bor-2026.vol40.041

**Published:** 2026-07-24

**Authors:** Regina Cardoso de Moura, Matheus França Perazzo, Soraya Coelho Leal, Lucianne Cople Maia, Carla Massignan

**Affiliations:** (a) Universidade de Brasília – UnB, School of Health Sciences, Department of Dentistry, Brasília, DF, Brazil.; (b) Universidade Federal de Goiás, School of Dentistry, Department of Social Dentistry8, Goiânia, GO, Brazil.; (c) Universidade Federal do Rio de Janeiro – UFRJ, School of Dentistry, Department of Orthodontics and Pediatric Dentistry, Rio de Janeiro, RJ, Brazil.

**Keywords:** Dental Caries, Evidence-Based Dentistry, Knowledge, Attitude of Health Personnel, Professional Practice Gaps

## Abstract

This cross-sectional study evaluated the knowledge, skills, attitudes, and perceived barriers of dentists from the Federal District, Brazil, regarding Minimal Intervention Dentistry (MID). A validated online questionnaire was administered using non-probabilistic snowball sampling, and 404 dentists participated (74% female; mean professional experience: 12 ± 10.9 years). Data were analyzed using descriptive and inferential statistics, including multiple linear regression (p < 0.05). The dependent variables were knowledge, skills, attitudes, and barriers, while the independent variables included administrative region, years of professional experience, type of graduation institution, academic qualification, professional role, and information-seeking behavior. Overall, participants demonstrated high levels of competency in MID, particularly in the preventive and diagnostic domains; however, selective carious tissue removal was the procedure performed least frequently (38.6%). Regression analyses showed that dentists involved in teaching or research reported significantly fewer barriers (β = −2.56; 95% CI: −3.90 to −1.23; p < 0.001). Dentists who had received formal training in MID achieved higher scores in knowledge (β = 2.95; 95%CI: 2.31 to 3.59; p < 0.001) and in skills and attitudes (β = 3.40; 95%CI: 2.18 to 4.62; p < 0.001), as well as fewer perceived barriers (β = −1.52; 95%CI: −2.35 to −0.68; p < 0.001). Information-seeking behavior was positively associated with greater knowledge (β = 2.35; 95%CI: 1.56 to 3.14; p < 0.001) and improved skills and attitudes (β = 3.06; 95%CI: 1.55 to 4.56; p < 0.001). Graduates from public universities presented higher scores for skills and attitudes (β = 2.17; 95%CI: 1.15 to 3.18; p < 0.001) and reported fewer barriers (β = −0.88; 95%CI: −1.61 to −0.15; p < 0.01). Brazilian dentists demonstrated a strong theoretical understanding of MID; however, practical implementation remains limited. Academic background, involvement in education and research, and proactive learning behaviors were key determinants of higher competency levels and reduced barriers to the adoption of MID

## Introduction

Minimal Intervention Dentistry (MID) is a contemporary team-based approach to oral healthcare that aims to maximize the preservation of tooth structure and extend tooth longevity, thereby enhancing long-term oral health and overall well-being.^1^ MID is founded on four fundamental principles: a) Recognition – early identification and assessment of potential caries risk factors; b) Reduction – implementation of measures to eliminate or reduce caries risk factors; c) Regeneration – control and reversal of initial carious lesions; and d) Repair – conservative carious tissue removal when cavitation is present, in order to optimize the tooth's repair potential while preserving its structural integrity.^2^

This approach offers several advantages for patients, as it promotes better clinical outcomes by emphasizing the maintenance and promotion of oral health.^3^ In addition, MID is less costly in the long term and often more effective than conventional caries management strategies.^4^ Dentists currently have a broader range of treatment options in cariology and unprecedented access to scientific evidence supporting these approaches.^3^ However, a persistent gap remains between research findings and their translation into clinical practice.^5^

The management of dental caries by dentists has become a central focus in efforts to reduce its global burden.^5^ Despite robust evidence supporting noninvasive and nonrestorative treatments, a considerable proportion of dentists continue to adopt overly invasive interventions for initial caries lesions,^6^ as demonstrated by systematic reviews. A substantial number of practitioners still employ surgical approaches for enamel lesions and proximal lesions at the dentin–enamel junction.^6^ Similarly, in the management of deep caries lesions, dentists frequently fail to adopt evidence-based strategies for carious tissue removal.^7^ These findings highlight the need for a new oral health agenda that promotes research aimed at understanding the barriers and facilitators to the systematic incorporation of MID protocols into clinical practice.^8^ A systematic review with meta-analysis evaluating dentists’ knowledge, attitudes, and practices regarding MID revealed that, despite adequate theoretical knowledge, dentists often experience limitations in applying this information in daily practice.^9^

Given the substantial body of evidence supporting MID and its benefits for lifelong oral health, it is essential that dentists, including those practicing in Brazil, adhere to this care approach. Therefore, further investigation into dentists’ competencies in MID-related caries management and the factors limiting its theoretical and practical application is warranted. To date, only one Brazilian study has assessed dentists’ competencies regarding general MID principles, briefly addressing barriers to implementation more than a decade ago.^10^ Within the Brazilian regional context, the present study is the first to evaluate dentists’ competencies and perceived barriers related to the general concepts of MID using a previously validated and psychometrically tested instrument. Accordingly, this cross-sectional study aimed to assess the knowledge, skills, attitudes, and barriers related to MID among dentists from the Federal District (FD), Brazil, and to examine their associations with professional profile and educational background.

## Methods

### Study characterization

This cross-sectional study employed a validated online instrument for data collection. The study was approved by the Ethics Committee of the Faculty of Health Sciences, University of Brasília (CAAE: 47639021.8.0000.0030), and was conducted in accordance with Resolution No. 466/2012 of the Brazilian National Health Council. All participants provided informed consent prior to participation. The study was reported following the STROBE (Strengthening the Reporting of Observational Studies in Epidemiology) guidelines.^11^

The study protocol is available upon request from the authors and can also be accessed through the link provided in the supplementary material.

### Population and sample selection

The target population consisted of dentists practicing in the Federal District (FD), Brazil. Data collection was conducted between August 2022 and March 2023. The inclusion criterion was being a dentist actively practicing in the FD. Dentists without active registration with the Regional Dentistry Council were excluded from the study.

### Instrument design and data collection

The original instrument is available in the supplementary material at: https://osf.io/dy536/overview?view_only=48433d9894b6481882e9248557db2185. The questionnaire was structured into seven sections: (1) informed consent and participant eligibility; (2) sociodemographic characteristics, education, and professional experience; (3) education and knowledge regarding MID; (4) knowledge, skills, and attitudes related to MID; (5) knowledge, skills, and attitudes related to recognition, reduction, and remineralization in MID; (6) knowledge, skills, and attitudes related to minimally invasive restorations and restoration repair; and (7) perceived barriers to practice. Sections 3 through 7 included items designed to assess participants’ knowledge, skills, attitudes, and perceived barriers related to MID, using a 5-point Likert-type scale.

Sections 3, 4, 5, and 6 were developed based on Zarifian's concept of competencies,^12^ which encompasses three dimensions: knowledge (knowing), skills (know-how), and attitudes (willingness to apply know-how). Items assessing competencies were organized into thematic areas corresponding to the four core principles of MID proposed by Walsh^2^: recognition, reduction, regeneration, and repair. Specific items within each thematic area were developed based on adaptations from the MID literature^^2,13,14^^ and a previous study that assessed competencies in MID^15^.

Prior to data collection, the instrument underwent adaptation and psychometric evaluation, as reported in a validation study conducted by the research team. Due to the impracticality of contacting the entire target population to obtain a random sample, a non-probabilistic snowball sampling strategy was adopted.^16,17^ The questionnaire was distributed via URL and QR code through multiple channels: a) email distribution lists provided by a partner radiology center and the Dentists’ Union of the FD; b) social media platforms (WhatsApp, Facebook, and Instagram), both in professional groups and individual accounts; and c) printed flyers distributed in dental clinics across the FD. All invited participants were encouraged to forward the invitation to other dentists practicing in the FD.

The sample size was calculated using the formula available at http://calculoamostral.bauru.usp.br/calculoamostral/, considering a 95% confidence level, a 5% margin of error, a design effect of 1.5, and an anticipated 10% loss. In June 2022, 8,944 dentists were registered with the Regional Dentistry Council of the FD. A pilot study with 30 dentists indicated that 20% (n = 6) were familiar with MID but reported difficulties in its practical application. The parameters regarding dentists’ knowledge of MID and challenges related to its implementation were based on a previously conducted systematic review.^9^ Based on these assumptions, the minimum required sample size was estimated at 399 dentists. Participant losses were attributed to individuals who accessed the questionnaire but declined participation or did not meet the eligibility criteria.

### Data analysis and interpretation methodology

All responses were tabulated in a Microsoft Excel spreadsheet and subsequently exported to the Statistical Package for the Social Sciences (SPSS for Windows, version 21.0; SPSS Inc., Armonk, NY, USA) for analysis.

Dentists’ competencies and perceived barriers were described using absolute and relative frequencies. For analytical purposes, responses of 4 and 5 on the 5-point Likert scale were combined into an "agreement" category, responses of 1 and 2 into a "disagreement" category, and response 3 was classified as "neutral."

For competency-related items, responses of 4 and 5 indicated agreement with the item and alignment with current evidence on MID, thus confirming the presence of competence. For barrier-related items, responses of 4 and 5 indicated perceived difficulty. To synthesize competency levels, the following criteria were adopted^18^ based on the combined frequency of responses 4 and 5: up to 25% was classified as insufficient competence, 26–50% as reasonable, 51–75% as good, and above 76% as excellent.

For multiple linear regression analyses, the dependent variables (knowledge, skills, attitudes, and barriers) were transformed from ordinal variables (Likert scale) into continuous variables, with total scores presented in the supplementary material (https://osf.io/dy536/overview?view_only=48433d9894b6481882e9248557db2185). No violations of the assumptions of multiple linear regression were identified.^19^ Spearman's correlation and simple linear regression analyses were initially performed. Variables with a p-value ≤ 0.20 in simple regression were included in the multiple regression models, along with the adjustment variables "Have you ever heard of MID" and "Have you received training in MID."

## Results

### Participants’ characteristics

A total of 454 dentists accessed the instrument. Excluding professionals who were not eligible for the study (n = 20; 4.4%) or did not agree to participate (n = 30; 6.6%), 404 dentists completed the survey responses and were included in this study. The results of the socioeconomic items are described in Table 1.

**Table 1:** Profile, education, experience and training in MID of dentists of Federal District, 2023.

Variables		n (%)
Gender (n = 404)		
	Female	299 (74)
	Male	105 (26)
	Age in years (n = 404)	38 (10.4)
Professional activity (n = 404)		
	Years of experience (n = 404)	12 (10.9)
	Undergraduate professor	25 (6.2)
	Graduate-level professor	2 (0.5)
	Public health clinic	80 (19.8)
	Owned private clinic	136 (33.7)
	Shared private clinic	133 (32.9)
	Philanthropic clinic	15 (3.7)
	Hospitals	2 (0.4)
	Research	5 (1.2)
	Forensic expertise	1 (0.2)
	Management	2 (0.2)
	Do not work as dentist	3 (0.7)
	Undergraduate university (n = 404)	
	Public university	173 (42.8)
	Private university without aids or scholarships	160 (39.6)
	Private university with aids or scholarships	71 (17.6)
Academic degree (n = 404)		
	Bachelor's	58 (14.4)
	Specialized	247 (61.1)
	Master's	70 (17.3)
	Doctoral	26 (6.4)
	Postdoctoral	3 (0.7)
Specialty (n = 637)		
	None	57 (8.9)
	Pediatric dentistry	79 (12.4)
	Implantology	70 (11.0)
	Dental prosthodontics	65 (10.2)
	Orthodontics	58 (9.1)
	Restorative dentistry	56 (8.8)
	Endodontics	51 (8.0)
	Other dental specialties	170 (35.6)
	Postgraduate in other fields	31 (4.9)
Have you received training in MID? (n = 404)		
	Yes	290 (71.8)
	No	114 (28.2)
	Where did most of the MID training take place? (n = 404)	
	Undergraduate	121 (41.7)
	Specialization	44 (15.2)
	Master's	20 (6.9)
	Doctorate	4 (1.4)
	Free courses – e.g: improvement, update, extension	101 (34.8)
What is your primary source of information on MID? (n = 404)		
	I do not seek information	66 (16.3)
	Courses or lectures	134 (33.2)
	Non-scientific internet pages	8 (2.0)
	Social media (e.g., Facebook, Instagram, WhatsApp, Telegram)	27 (6.7)
	Scientific articles in Portuguese	35 (8.7)
	Scientific articles in English and other languages	95 (23.5)
	Books or clinical practice guides	36 (8.9)
	Various sources	3 (0.7)

Most participants were female (n = 299; 74.0%), worked in their own or shared private clinics (n = 269; 66.6%), and held specialization as their highest qualification (n = 247; 61.1%). Among the specialties, the most frequent were pediatric dentistry (n = 79; 12.4%), implantology (n = 70; 11.0%), prosthodontics (n = 65; 10.2%), and restorative dentistry (n = 56; 8.8%). It is worth noting that there were dentists with more than one specialty.

Concerning training in MID, most dentists reported having received training (n =2 90; 71.8%). Among the training places, the most frequent were during undergraduate (n = 121; 41.7%) and in free courses (n = 101; 33.2%). The most sought-after sources of information about MID were courses or lectures (n = 134; 33.2%), followed by scientific articles in English and other foreign languages (n = 95; 23.5%).

### Main results of competencies and barriers in MID

Table 2 shows the results on knowledge, skills, and attitudes about MID. Knowledge items had more compliance evaluations, particularly in the "Recognition, Reduction, and Remineralization" areas. The item with the highest percentage of compliance was identifying caries risk factors, totaling 86.1% of dentists rating excellent. The item with the lowest percentage of positive assessment was knowledge about MID for managing dental caries, with a 59.9% agreement, rating good.

**Table 2:** Competencies of dentists in the Federal District on Minimal Intervention Dentistry in 2023 (n = 404).

Competence: Knowledge on MID							
Theme – Recognition, reduction and remineralization							
Likert-Scale	1 (Nothing)	2	3	4	5 (Very much)	Sum of scale 4 and 5	Classification*
	n (%)	n (%)	n (%)	n (%)	n (%)	%	
1) How much do you know about identifying caries risk factors?	1 (0.2)	6 (1.5)	49 (12.1)	160 (39.6)	188 (46.5)	86.1	Excellent
2) How much do you know about controlling the onset of caries?	1 (0.2)	14 (3.5)	53 (13.1)	164 (40.6)	172 (42.6)	83.2	Excellent
3) How much do you know about controlling the progression of dental caries?	0 (0.0)	8 (2.0)	62 (15.3)	185 (45.8)	149 (36.9)	82.7	Excellent
**Theme – Minimally invasive restorations and restoration repair**							
4)… about minimally invasive restorative procedures?	6 (1.5)	19 (4.7)	74 (18.3)	181 (44.8)	124 (30.7)	75.5	Excellent
**Theme – MID in General**							
5)… about Minimal Intervention Dentistry for the management of dental caries?	12 (3.0)	40 (9.9)	110 (27.2)	157 (38.9)	85 (21.0)	59.9	Good
**Competence: Skills on MID**							
**Theme – Recognition, reduction and remineralization**							
**Initial sentence: How much do you agree with the following statement:**	**(Totally disagree)**				**(Totally agree)**		
1) "In patients at high risk for dental caries, changing habits, such as reducing the frequency of food sources of sugars, is an important factor in controlling the onset of the disease."	1 (0.2)	11 (2.7)	26 (6.4)	96 (23.8)	270 (66.8)	90.6	Excellent
2) "Assessing the frequency of consumption of food that are sources of sugars is a good indicator of future risk for caries disease."	6 (1.5)	16 (4.0)	50 (12.4)	91 (22.5)	241 (59.7)	82.2	Excellent
3) "Exposure to fluoride sources is a good indicator of protection against caries disease."	9 (2.2)	18 (4.5)	80 (19.8)	114 (28.2)	183 (45.3)	73.5	Good
**Theme – MID in General**							
4) "The goal of Minimal Intervention Dentistry is to keep teeth healthy and functional throughout life, and it involves implementing important strategies to keep teeth free of caries lesions. These strategies are early caries detection and risk assessment; Remineralization of demineralized enamel and dentin; optimal caries prevention measures; minimally invasive operative interventions and repair rather than replacement of restorations."	2 (0.5)	5 (1.2)	32 (7.9)	82 (20.3)	283 (70.0)	90.3	Excellent
**Theme – Minimally invasive restorations and restoration repair**							
5) "The removal of carious dentin differs according to the depth of the caries lesions, and should be more conservative in deep lesions, to avoid pulp exposure in vital teeth".	22 (5.4)	21 (5.2)	42 (10.4)	88 (2.,8)	231 (57.2)	79.0	Excellent
6) "In defective restorations, repair should be considered before opting for removal of the restoration and performing a new one."	24 (5.9)	16 (4.0)	59 (14.6)	72 (17.8)	233 (57.7)	75.5	Good
**Competence: Attitudes on MID**							
**Likert-Scale**	**1 (Never)**	**2**	**3**	**4**	**5 (Always)**	**Sum of scale 4 and 5**	Classification*
**Theme – Recognition, reduction, and remineralization**							
1) How often do you advise on daily toothbrushing with fluoridated toothpaste for patients at high risk for dental caries?	5 (1.2)	2 (0.5)	11 (2.7)	29 (7.2)	357 (88.4)	386 (95.6)	Excellent
2) How often do you advise reducing the consumption of food sources of sugars for patients at high risk for dental caries?	12 (3.0)	16 (4.0)	35 (8.7)	86 (21.3)	255 (63.1)	341 (84.4)	Excellent
3) How often do you assess your patient's risk of dental caries?	19 (4.7)	39 (9.7)	85 (21.0)	103 (25.5)	158 (39.1)	261 (64.6)	Good
**Theme – MID General**							
4) How often do you apply Minimal Intervention Dentistry to the management of dental caries in your daily clinical practice?	22 (5.4)	29 (7.2)	63 (15.6)	119 (29.5)	171 (42.3)	290 (71.8)	Good
**Theme – Minimally invasive restorations and restoration repair**							
5) How often do you perform the repair rather than the complete restoration replacement when managing defective restorations?	18 (4.5)	32 (7.9)	103 (25.5)	128 (31.7)	123 (30.4)	251 (62.1)	Good

Regarding skills in MID, the highest-rated skill item was about agreement with reducing the frequency of consumption of food sources of sugars in patients at high risk for dental caries, with 90.6% agreement, rating excellent. The lowest rate, though still good, was agreement on exposure to fluoride sources as a good indicator of protection against dental caries, with 73.5% agreement.

Concerning attitudes towards MID, the highest-rated attitude item was about advising on daily toothbrushing with fluoridated toothpaste for patients at high risk for dental caries, with 95.6% agreement, rating it excellent. The lowest rate was about leaving soft carious tissue on the pulpal floor wall to avoid pulp exposure in restorations of deep carious lesions of vital teeth, with 38.6% agreement, the only item rating ‘reasonable’.

Most dentists did not consider the situations presented as obstacles to obtaining information about MID, as seen in Table 3. The same can be said about barriers to practicing MID, except for the item about adequate remuneration (Sentence in the questionnaire: For adequate reimbursement for minimal intervention procedures, how much difficulty do you experience in applying Minimal Intervention Dentistry in clinical practice?), which had a higher proportion of dentists who evaluated with Likert levels 1 and 2 (38.3% agreement), indicating it as an obstacle to practicing MID.

**Table 3:** Barriers faced by dentists in Federal District for Minimal Intervention Dentistry in 2023 (n = 404).

Variables	1	2	3	4	5	Sum of scale 4 and 5
	(Never)				(Always)	
	n (%)	n (%)	n (%)	n (%)	n (%)	%
Theme - Challenges in obtaining information						
1) Access to technology for obtaining information.	294 (72.8)	54 (13.4)	39 (9.7)	12 (3.0)	5 (1.2)	4.2
2) Availability of reliable information sources.	147 (36.4)	112 (27.7)	93 (23.0)	42 (10.4)	10 (2.5)	12.9
3) Availability of finances to access information sources	156 (38.6)	104 (25.7)	91 (22.7)	40 (9.9)	13 (3.2)	13.1
4) Foreign languages of information sources	163 (40.3)	100 (24.8)	81 (20.0)	37 (9.2)	23 (5.7)	14.9
5) Time and organization for knowledge updating	54 (13.4)	107 (26.5)	143 (35.4)	59 (14.6)	41 (10.1)	24.7
Theme -Difficulties in practice						
1) Confidence in the effectiveness of minimal intervention	199 (49.3)	71 (17.6)	63 (15.6)	40 (9.9)	31 (7.7)	17.6
2) Interest in practicing MID	222 (55.0)	70 (17.3)	53 (13.1)	31 (7.7)	28 (6.9)	14.6
3) Practical skills in minimal intervention	175 (43.3)	100 (24.8)	67 (16.6)	46 (11.4)	16 (4.0)	15.4
4) Patients’ confidence in minimal intervention procedures	97 (24.0)	102 (25.2)	94 (23.3)	63 (15.6)	48 (11.9)	27.5
5) Patient willingness to pay for minimal intervention procedures	89 (22.0)	65 (16.1)	109 (27.0)	59 (14.6)	82 (20.3)	34.9
6) Adequate remuneration for minimal intervention procedures	71 (17.6)	62 (15.3)	116 (28.7)	72 (17.8)	83 (20.5)	38.3

### Result of multiple linear regression with dependent variables

An assessment of how the variables were related was conducted through Spearman's linear correlation, as seen in Table 4. The multiple linear regression resulted in a statistically significant model for the association between dependent variables and predictive variables, as can be seen in Tables 5, 6, 7 and 8.

**Table 4:** Linear correlation between the average knowledge; skills/attitudes; barriers to knowledge and barriers to practice in MID scores with independent variables (n = 404)

Variables	Knowledge			Skills and Attitudes		
	ρ	p-valor	ρ	p-valor
Gender	0.13	0.004	0.11	0.01
Age (years)	-0.12	0.006	0.11	0.01
Administrative Region of Residence	0.04	0.17	0.13	0.003
Years of experience	-0.14	0.002	0.10	0.02
Graduation Institution	0.11	0.01	0.22	< 0.001
Highest Academic Qualification	-0.05	0.15	0.007	0.44
Professional role	0.16	0.001	0.09	0.02
Heard of MID	0.23	< 0.001	0.15	0.001
Information seeking	0.43	< 0.001	0.36	< 0.001
Received training in MID	0.52	< 0.001	0.33	< 0.001
	Barriers to knowledge			Barriers to practice		
Gender	0.06	0.08	-0.02	0.31
Age (Years)	0.03	0.25	-0.13	0.003
Administrative Region of Residence	-0.01	0.40	-0.09	0.03
Years of experience	0.01	0.35	-0.15	0.001
Undergraduate institution	-0.13	0.003	-0.04	0.21
Highest degree	-0.10	0.01	-0.09	0.02
Professional Activity	-0.19	< 0.001	-0.07	0.08
Heard about MID	0.001	0.49	-0.02	0.33
Information seeking	-0.03	0.25	-0.09	0.02
Received MID training	-0.16	< 0.001	-0.12	0.005

* Spearman's linear correlation; MID = minimum intervention dentistry.

**Table 5:** Multiple linear regression for the association between knowledge: barriers to practice in Minimal Intervention Dentistry (MID) (n = 404).

		Knowledge in MID				
Variable		Unadjusted		Adjusted	
		β 95%CI	p-value	β 95%CI	p-value
Gender					
	Female	1.02 (0.28; 1.76)	0.007	0.56 (-0.04; 1.16)	0.06
	Male				
Age (Years)		-0.04 (-0.07;-0.009)	0.01	0.04 (-0.02; 0.11)	0.22
Administrative Region of Residence					
	Plano Piloto	0.34 (-0.39; 1.07)	0.35		
	Other regions				
Years of experience		-0.04 (-0.07; -0.01)	0.003	-0.06 (-0.13; 0.01)	0.09
Graduation institution					
	Public	0.74 (0.08; 1.40)	0.02	0.56 (0.03; 1.09)	0.03
	Private				
Highest qualification					
	Postgraduate	-0.49 (-1.42; 0.44)	0.30		
	Graduate				
Professional activity					
	Teaching/Research	2.01 (0.81; 3.20)	0.001	1.24 (0.27; 2.21)	0.01
	Other				
Heard of MID					
	Yes	5.06 (3.01; 7.11)	< 0.001	1.00 (-0.78; 2.79)	0.27
	No				
Information seeking					
	Yes	3.97 (3.17; 4.76)	< 0.001	2.35 (1.56; 3.14)	< 0.001
	No				
Received training in MID					
	Yes	3.89 (3.27; 4.51)	< 0.001	2.95 (2.31; 3.59)	< 0.001
	No				

R2: 0.38; Adjusted R2: 0.36. Bold indicates statistical significance. All independent variables with p ≤ 0.20 in simple regression were included in the adjusted model. "Heard of MID" and "Received training in MID" were considered adjustment variables.

**Table 6:** Multiple linear regression for the association between knowledge: skill and attitude in MID. (n = 404).

		Skill and Attitude in MID					
Variable		Unadjusted		Adjusted	
		β 95%CI	p-value	β 95%CI	p-value
Gender					
	Female	1.43 (0.16; 2.70)	0.02	1.22 (0.07; 2.37)	0.03
	Male				
Age (years)		0.06 (0.01; 0.11)	0.02	0.12 (-0.01; 0.26)	0.08
Administrative region of residence					
	Plano Piloto	1.72 (0.48; 2.97)	0.007	0.59 (-0.56; 1.76)	0.31
	Other Regions				
Years of experience		0.05 (0.001; 0.10)	0.04	-0.04 (-0.17; 0.09)	0.55
Undergraduate institution					
	Public	2.57 (1.47; 3.68)	< 0.001	2.17 (1.15; 3.18)	< 0.001
	Private				
Highest degree					
	Postgraduate	0.12 (-1.47; 1.71)	0.88		
	Undergraduate				
Professional Activity					
	Teaching/Research	1.99 (-0.07; 4.05)	0.05	0.86 (-0.99; 2.72)	0.36
	Others				
Heard about MID					
	Yes	5.73 (2.17; 9.29)	0.002	0.83 (-2.55; 4.21)	0.62
	No				
	Information Seeking				
	Yes	3.13 (3.70; 6.56)	< 0.001	3.06 (1.55; 4.56)	< 0.001
	No				
Received MID Training					
	Yes	4.19 (3.02; 5.37)	< 0.001	3.40 (2.18; 4.62)	< 0.001
	No				

R^2^ 0.23; Adjusted R^2^ 0.22. Bold indicates statistical significance. All independent variables with p ≤ 0.20 in simple regression were included in the adjusted model. The variables "Heard about MID" and "Received MID Training" were considered adjustment variables.

**Table 7:** Multiple linear regression for the association between barriers to knowledge (n = 404).

		Barriers to knowledge in MID					
Variable		Unadjusted		Ajusted	
		β 95%CI	p-value	β 95%CI	p-value
Gender					
	Female	0.59 (-0.25; 1.44)	0.16	0.73 (-0.08; -1.56)	0.08
	Male				
Age (years)		0.01 (-0,02;0,04)	0.51		
Administrative region of residence					
	Plano Piloto	-0.10 (-0,93; 0,72)	0.08	0.31 (-0.53; 1.15)	0.46
	Other regions				
Years of experience		0.007 (-0.02; 0.04)	0.07	0.01 (-0.02; 0.04)	0.54
Undergraduate institution					
	Public	-1.05 (-1.80; -0.31)	0.005	-0.88 (-1.61; -0.15)	0.01
	Private				
Highest qualification					
	Postgraduate	-1.11 (-2.17; -0.06)	0.03	-1.11 (-2.22; -0.003)	0.04
	Undergraduate				
Professional activity					
	Teaching/Research	-2.71 (-4.06; -1.35)	< 0.001	-2.56 (-3.90; -1.23)	< 0.001
	Others				
Heard about MID					
	Yes	0.01 (-2.38; 2.41)	0.99	1.10 (-1.25;1.45)	0.36
	No				
Information seeking					
	Yes	-0.34 (-1.34; 0.66)	0.50		
	No				
Received MID training					
	Yes	-1.50 (-2.32; -0.69)	< 0.001	-1.52 (-2.35; -0.68)	< 0.001
	No				

R^2^ 0.10; Adjusted R^2^ 0.08. Bold indicates statistical significance. All independent variables with p ≤ 0.20 in simple regression were included in the adjusted model. The variables "Heard about MID" and "Received MID Training" were considered adjustment variables.

**Table 8:** Multiple linear regression for the association between barriers to practice in MID (n = 404).

Barriers to Practice in MID					
Variable	Unadjusted		Adjusted	
	β 95%CI	p-value	β 95%CI	p-value
Gender					
Female	-0.32 (-1.64; 0.99)	0.63		
Male				
Age (years)	-0.07 (-0.13;-0.02)	0.006	-0.07 (-0.14; 0.18)	0.01
Residential administrative region					
Plano Piloto	-1.24 (-2.53; 0.05)	0.06	-0.49 (-1.82; 0.83)	0.46
Other regions				
Years of Experience (years)	-0.08 (-0.13; -0.02)	0.003		
Undergraduate Institution					
Public	-0.47 (-1.64; 0.69)	0.42		
Private				
Highest degree					
Postgraduate	-1.63 (-3.27; 0.01)	0.05	-0.85 (-2.57; 0.87)	0.33
Undergraduate				
Professional activity					
Teaching/Research	-1.52 (-3.66; 0.61)	0.16	-1,17 (-3.31; 0.96)	0.28
Others				
Heard about MID					
Yes	-0.81 (-4.53; 2.91)	0.66	0.68 (-3.20; 4.57)	0.72
No				
Information seeking					
Yes	-1.53 (-3.09; 0.02)	0.05	-0.67 (-2.40; 1.04)	0.43
No				
Received MID training					
Yes	-1.67 (-2.94; -0.39)	0.01	-1.84 (-3.25; -0.44)	0.01
No				

R2: 0.05; Adjusted R2: 0.03. Statistically significant variables are shown in bold. All independent variables with p ≤ 0.20 in simple regression were included in the adjusted model. The variables "Heard about MID" and "Received MID Training" were considered adjustment variables.

On average, a higher knowledge score of MID of 0.56 points was observed among the dentists who studied in public universities compared to those who studied in private ones (95%CI = 0.03; 1.09; p = 0.03). Additionally, on average, dentists engaged with teaching or research presented 1.24 points more knowledge score in MID (95%CI = 0.27; 2.21; p = 0.01) compared to those working in clinics, private or public, and administrative services. Furthermore, dentists who seek information and those who received training in MID tended to exhibit, on average, higher knowledge scores (β =2.35; 95% CI =1.56; 3.14; p<0.001) and (β = 2.95; 95%CI = 2.31; 3.59; p < 0.001; respectively). The results indicate that this model can explain 36% of the variability in knowledge about MID.

A higher score, on average, in skills and attitudes about MID among female versus male dentists (β =1.22; 95%CI = 0.07; 2.37; p = 0.03), as well as among those who studied in public universities in comparison to those from private universities (β =2.17; 95%CI = 1.15; 3.18; p < 0.001) was observed. Additionally, dentists who seek information and those who have received training in MID tended to exhibit, on average, higher scores in skills and attitudes about MID (β = 3.06; 95%CI = 1.55; 4.56; p < 0.001) and (β = 3.40; 95%CI = 2.18; 4.62; p < 0.001; respectively). The results indicate that this model can account for 22% of the variability in skills and attitudes about MID.

Dentists who studied in public universities had, on average, fewer barriers to knowledge when compared to those who studied in private universities (β =-0.88; 95%CI = -1.61; -0.15; p = 0.01). The same holds for the dentists with postgraduate degrees as their highest qualification, those engaged in teaching or researching, or those who have received training in MID (β =-1.11; 95%CI = -2.22; -0.003; p = 0.04); (β = 2.56; 95%CI = -3.90; -1,23; p < 0.001}) and (β =-1.52; 95%CI = -2.35; -0.68; p < 0.001 respectively) in comparison to graduate dentists, other professional activities, and those who have not received MID training, respectively. The results indicate that this model can account for 8% of the variability in barriers to knowledge about MID.

Younger dentists, on average, had higher scores in barriers to practice (β =-0.07; 95%CI= -0.14; 0.18; p = 0.01). Dentists who have received training in MID tend to have, on average, fewer barriers to practice (β =-1,84; 95% CI = -3,25; -0,44; p=0,01). The results suggest that this model can explain 3 % of the variability in barriers to practicing MID.

## Discussion

This study evaluated the knowledge, skills, attitudes, and barriers dentists face regarding Minimal Intervention Dentistry (MID), correlating them with their profile and education. Overall, for the knowledge, skills, and attitudes, dentists showed a higher percentage of compliance with the items under analysis (Likert levels 4 and 5 summed), resulting in competency percentages classified as excellent or good. Regarding barriers to knowledge and barriers to practicing MID, in general, the higher percentages were related to the absence of difficulties with the items (Likert levels 1 and 2 summed). In the multiple linear regression, associations between dependent variables and predictive variables were found. Highlights were related to dentists engaged with teaching or research, those who have received training in MID, those who seek information, those who studied in public universities, and younger dentists.

In general, the evaluation of knowledge items complied with the literature. Previous studies in other countries within the same thematic areas of ‘Recognition, Reduction and Remineralization’, ‘Minimally invasive restorations’, and ‘Repair of restorations’ also found great outcomes of knowledge.^20-25^ The same can be said about the professionals’ assessments of skills and attitudes.^15,20,24,26^ In contrast, a Brazilian study that assessed MID concepts found knowledge levels ranging from reasonable to insufficient.^10^ However, it is important to highlight that this last study was carried out more than ten years ago, which might explain the divergence of the results, as MID has gained much attention in the last decade.

The item with the lowest percentage of compliance in the entire study, rating reasonable, was in attitudes, regarding leaving soft carious tissue on the pulpal floor walls to avoid pulp exposure. It is noteworthy that a meaningful percentage of dentists indicated not using this technique frequently (scoring 1 or 2 on the Likert scale). A previous systematic review investigating the proportion of dentists using selective removal of carious tissue found that nearly half of the dentists surveyed chose more invasive strategies to manage deep carious lesions in permanent teeth instead of more evidence-based approaches.^7^ Undoubtedly, this theme is a contentious item among professionals, despite all the evidence available supporting the selective caries removal in deep cavities.

In evaluating barriers to knowledge and practice of MID, only one item – ‘adequate remuneration for minimal intervention procedures’- had a higher percentage of dentists identifying it as an obstacle to practice according to their self-perception. This suggests that financial recognition may influence the adoption of MID in clinical settings. The same pattern was also observed in other countries, such as New Zealand, the United States, Germany, and England, as oral health care systems present higher valuation for restorative procedures than less invasive procedures.^27,28^ obstacle according to respondents. As we are not aware of any other Brazilian studies addressing this issue, further research on this topic is necessary.

The result of multiple linear regression showed that, on average, dentists who seek information about MID and have received training in the area presented higher scores in knowledge, skills, and attitudes. Other studies have also confirmed a significant relationship between prior training and knowledge of MID^22^, as well as between prior training and the practice of MID.^24^ However, one study did not find a significant relationship.^21^ An additional finding from the regression analysis was the association between female dentists and those who studied at public institutions, showing higher average scores in skills and attitudes related to MID. Similarly, another study found a positive association between the female gender and agreement with evidence on MID.^15^ A study from the United States suggests a greater caries preventive treatment philosophy with a greater focus on caries prevention by female dentists than by males.^29^ This practice pattern of female dentists may explain the differences observed in the present study, despite the variations across countries.

This study had some methodological limitations. Regarding the sample selection method, a non-probabilistic sample approach was conducted due to the impossibility of conducting a randomized selection for the target population. This choice may have introduced a selection bias, minimized by an extensive and careful selection of participants in the initial stage, including email, social media, and by folders delivered in dental clinics in the FD. Another limitation of the study is its cross-sectional nature, where the assessed data represents a cutout of the participants’ reality. It may not fully reflect their complete situation and could introduce a memory bias. We tried to minimize this by carefully selecting the items presented to participants. Besides, we conducted adjusted statistics with the variables having heard of MID and having received training on MID used as an adjustment in all models. The developed items were analyzed by a team of Brazilian MID experts and two focus groups consisting of independent dentists. After, a pre-test was carried out to verify the writing, sequence, and understanding of the items. Since the study was conducted within a local population, the findings and interpretation derived from it cannot be extrapolated to other populations.

The present study found that most dentists possess knowledge, skills, and attitudes regarding MID by literature, except for the attitude toward selective carious removal, which did not reach a consensus. Some characteristics seem to influence higher scores of competencies and fewer barriers, like studying in public universities, seeking information on MID, receiving training in MID, working with teaching or research, being female, having a postgraduate degree, and being younger. In the Brazilian higher education system, public universities have, in general, a close relation with the public healthcare system and the idea of prevention; this might have influenced the results. The higher scores among graduates from public universities likely reflect stronger emphasis on public health and prevention, while better outcomes among younger dentists may result from recent curricular updates aligned with MID principles. Based on the findings, it is recommended that further studies be conducted to expand the understanding of dentists’ potential and challenges regarding MID. Additionally, a qualitative approach is suggested to provide a more detailed exploration of these issues.

This study utilized an instrument with evidence of validation, comprehensive subject coverage, and an adequate sample size. It is understood that this research contributed to the understanding of the competencies and challenges related to MID.

Based on the findings of this study, a proposal for changes in the training of dentists can be made to achieve excellence in their education and promote minimum intervention oral care grounded on the best available evidence.

## Conclusion

Dentists demonstrated high levels of competency in Minimal Intervention Dentistry (MID), except with regard to the practice of selective carious tissue removal. Higher competency scores and fewer perceived barriers were observed among dentists who graduated from public universities, actively sought information on MID, received formal training in MID, were engaged in teaching or research activities, were female, held postgraduate degrees, and were younger. This study contributes to a broader understanding of the competencies and challenges associated with MID and supports the proposal of improvements in dental education and training. However, the study has limitations, including the use of a non-probabilistic sampling strategy and the possibility of response bias, which may limit the generalizability of the findings. Therefore, the results should be interpreted with caution.

## Data Availability

The authors declare that all data generated or analyzed during this study are included in this published article.
