# Supplementary material for: Dentists' knowledge, skills, attitudes and barriers towards minimal intervention dentistry
Source: Braz Oral Res. 2026 Jul 24;40:e041. doi: 10.1590/1807-3107bor-2026.vol40.041 (PMC13399977; doi:10.1590/1807-3107bor-2026.vol40.041)

## **Frame S1 -** Original online survey questionnaire to dentists in the Federal District on Minimal Intervention Dentistry, 2023


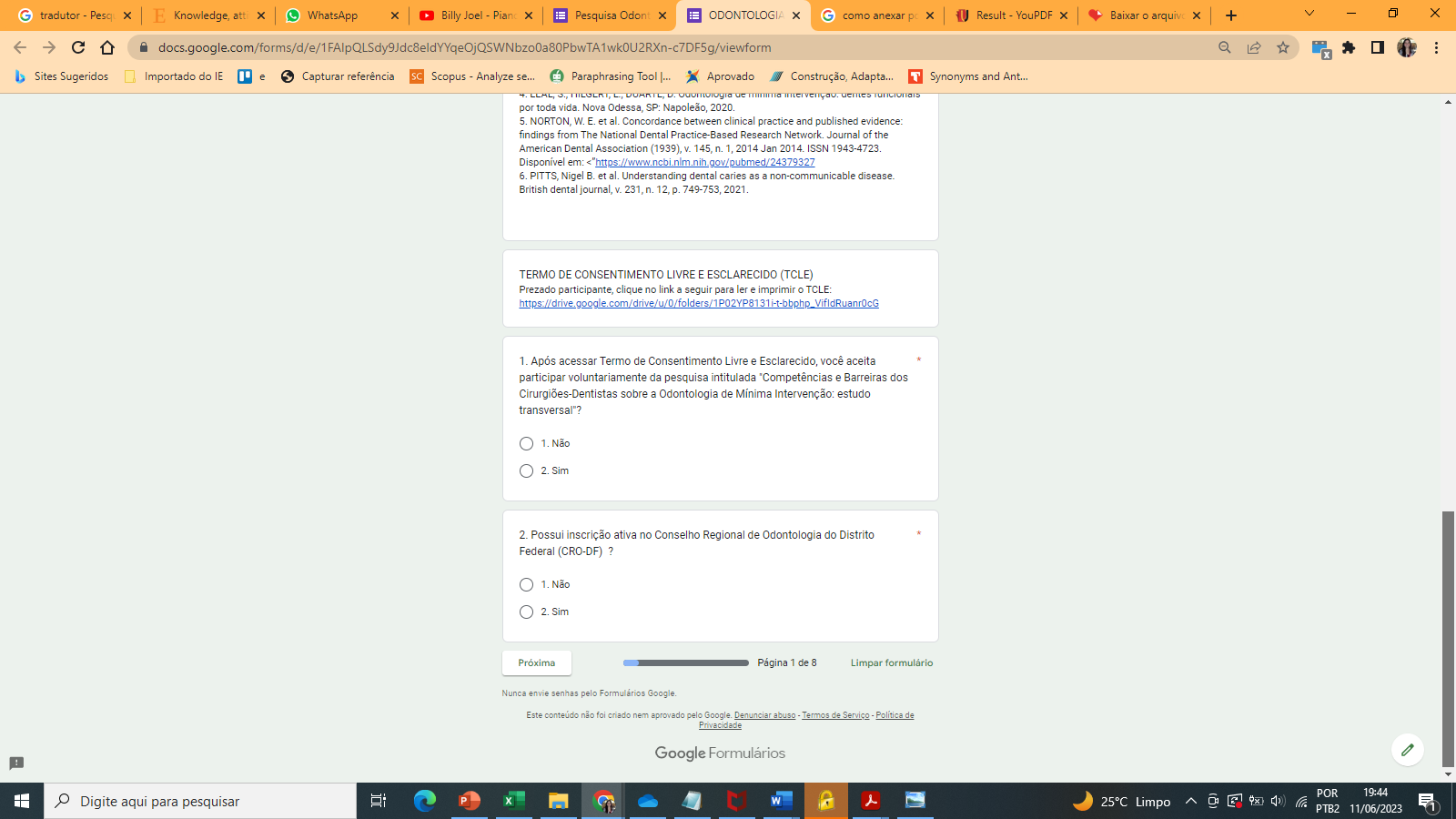


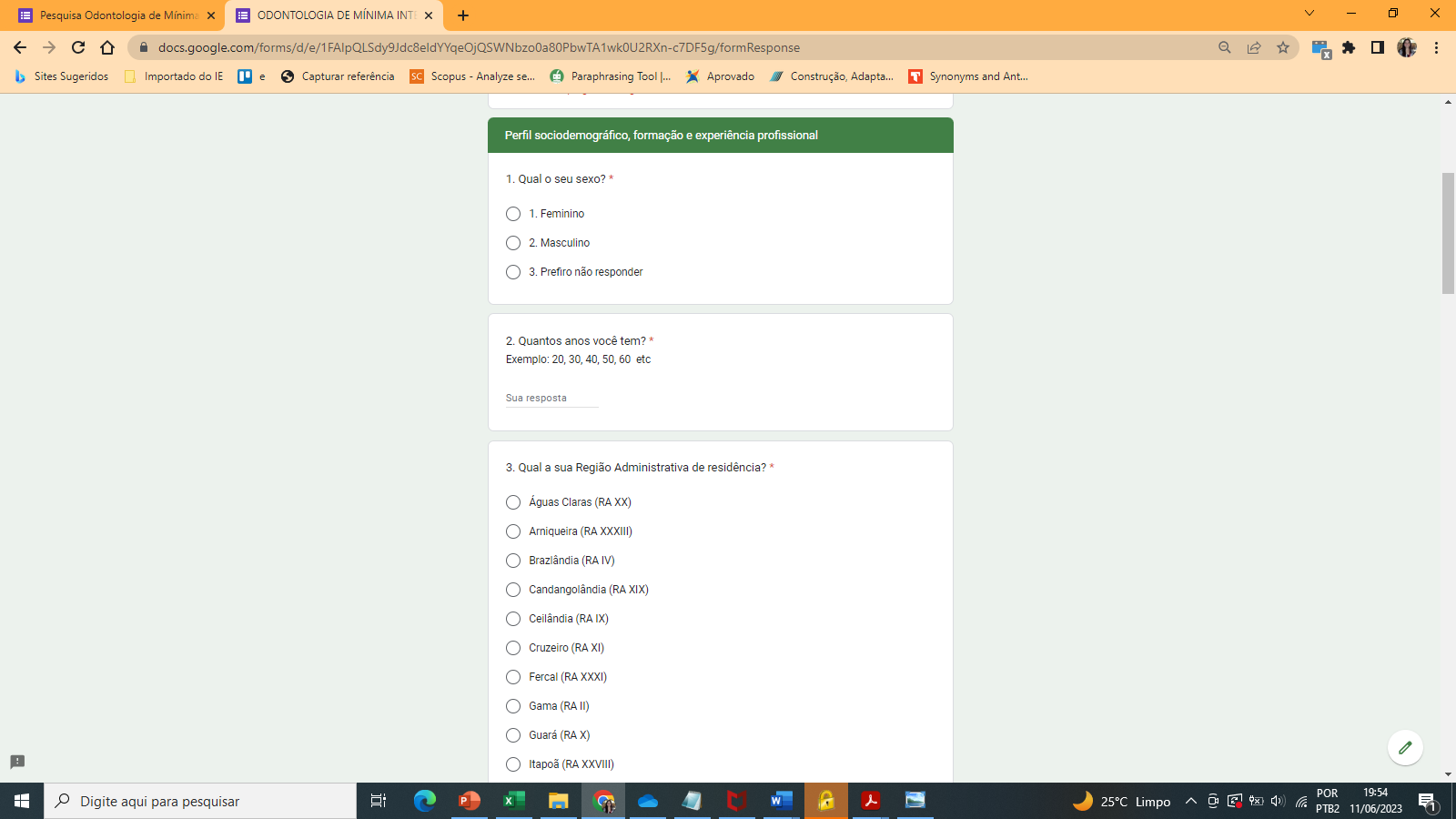


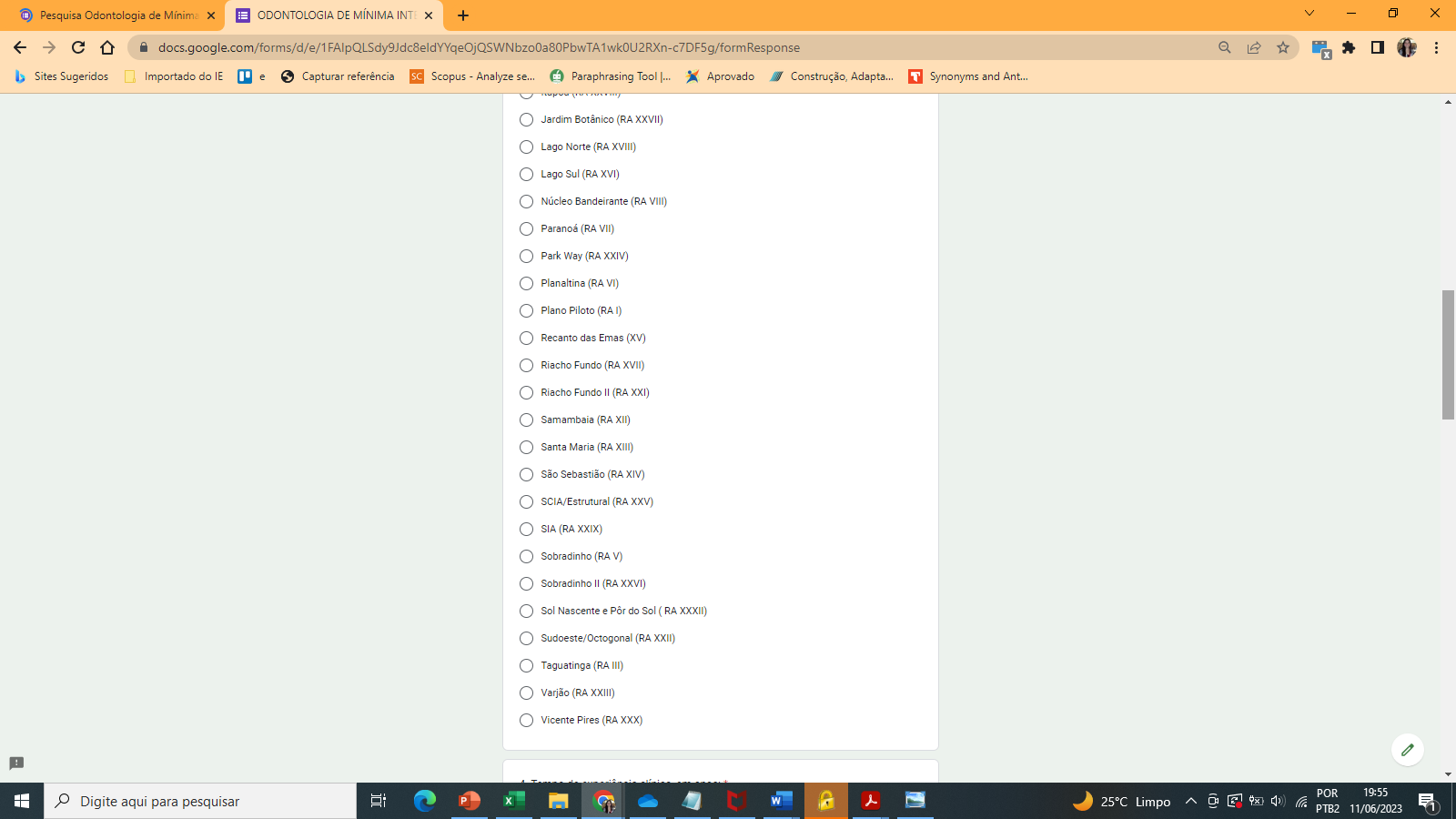


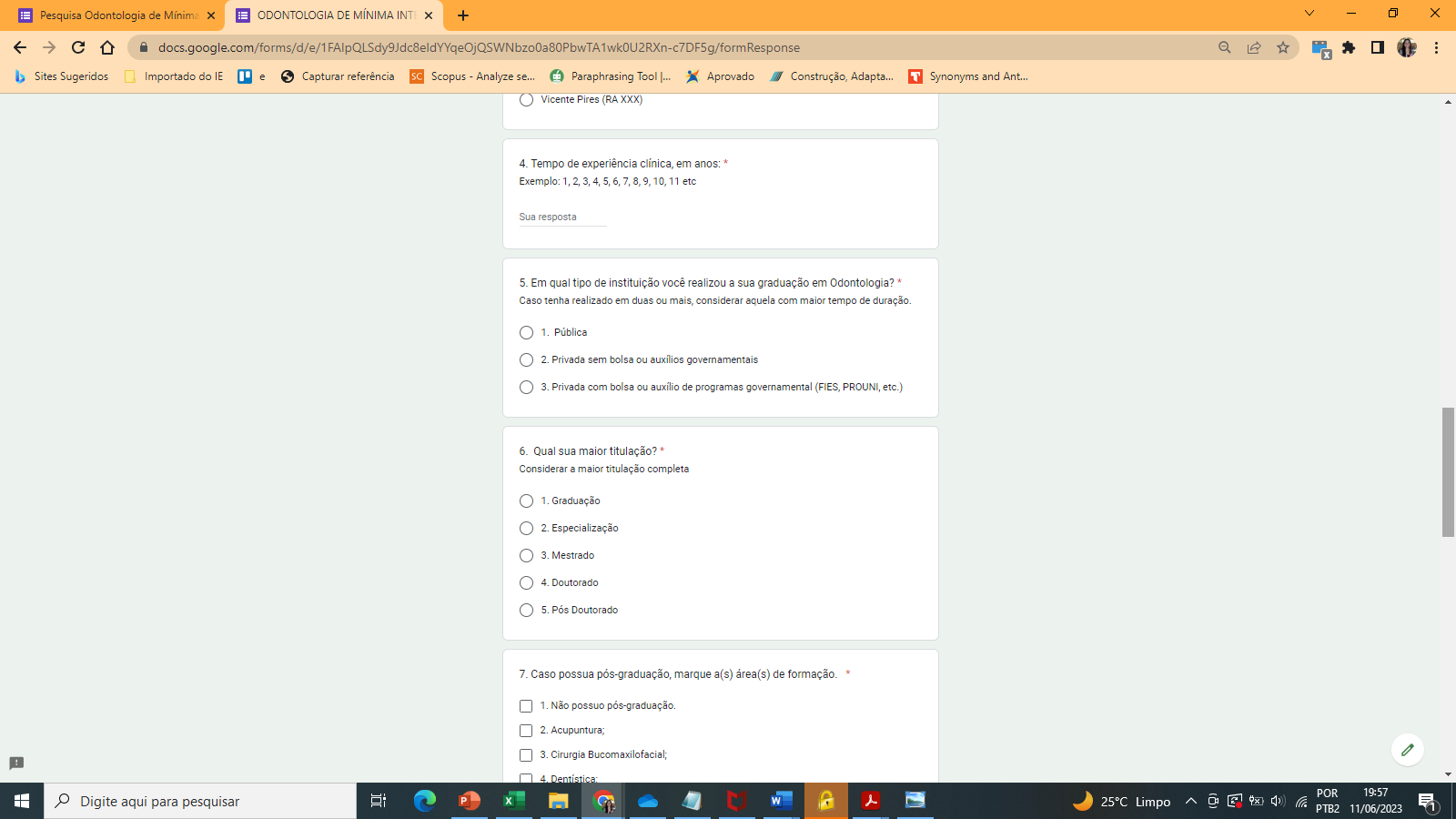


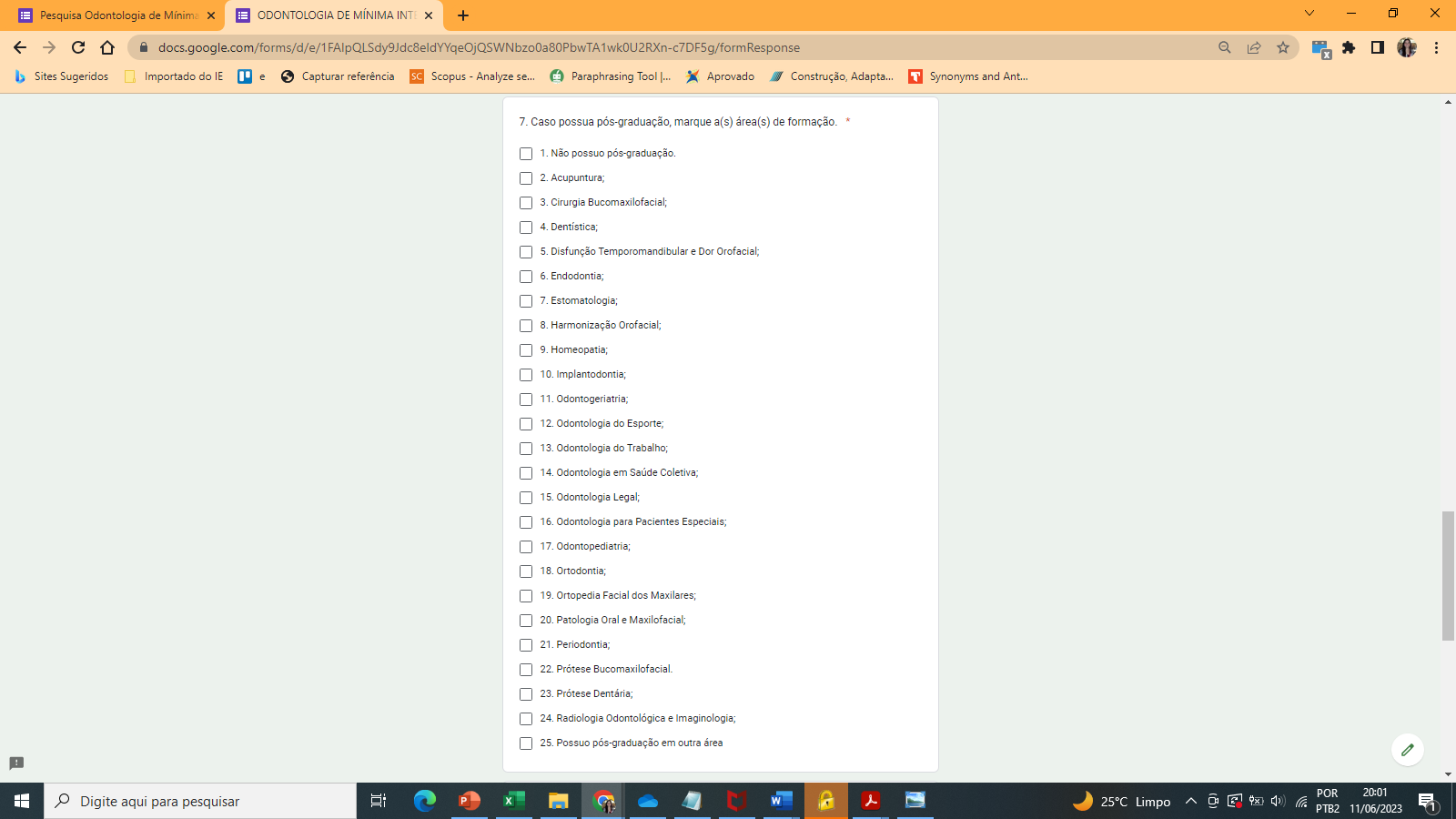


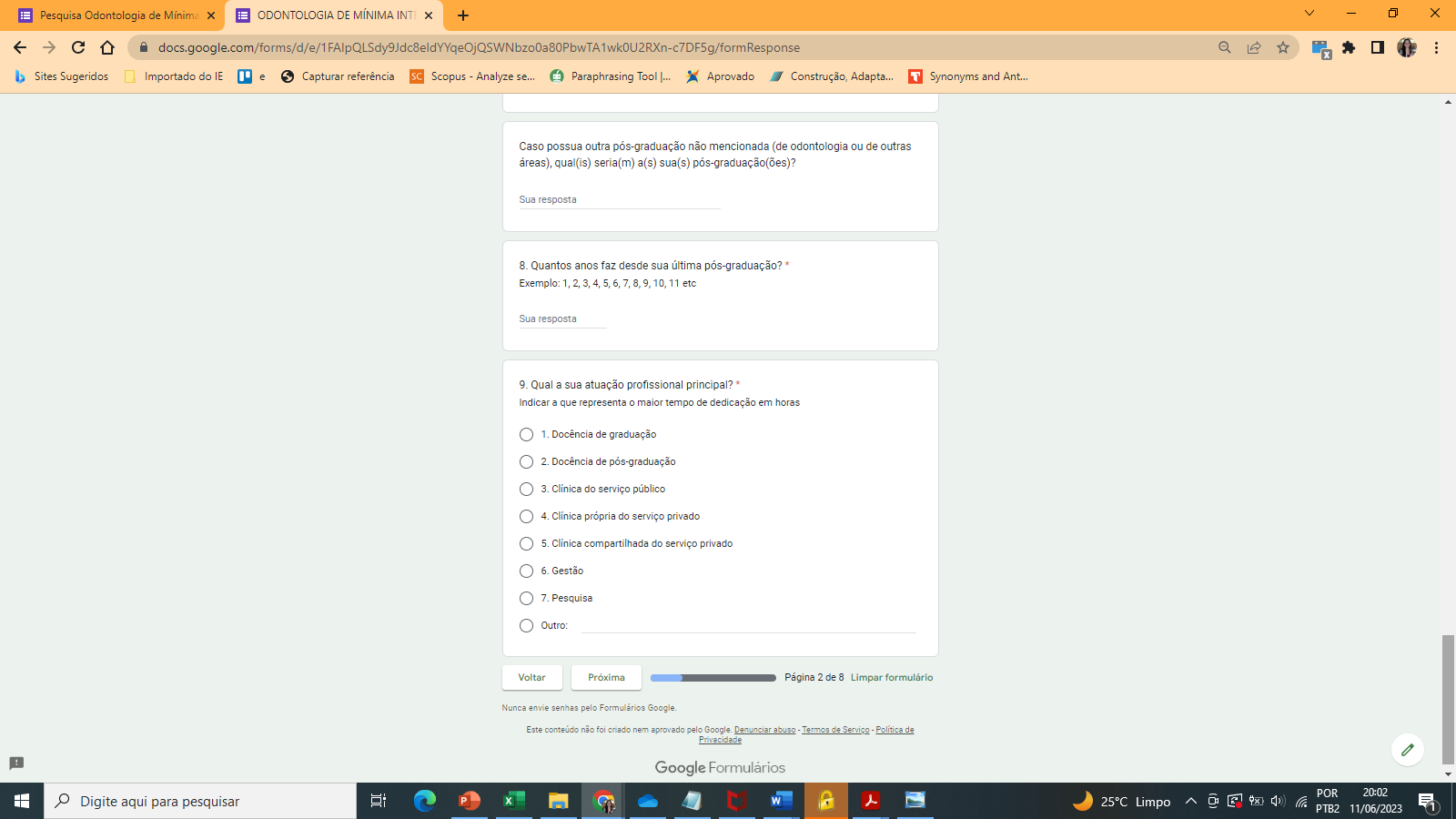


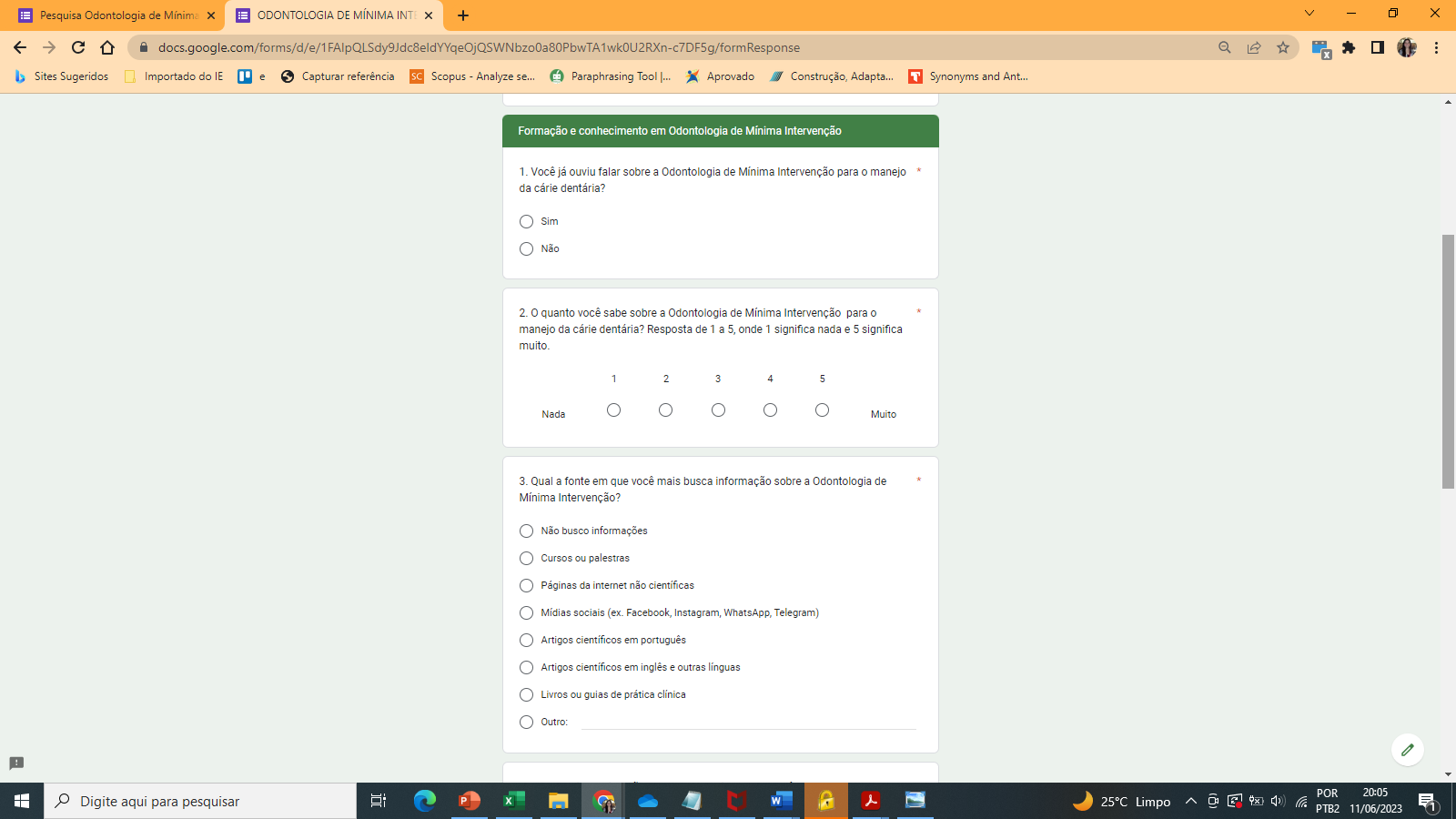


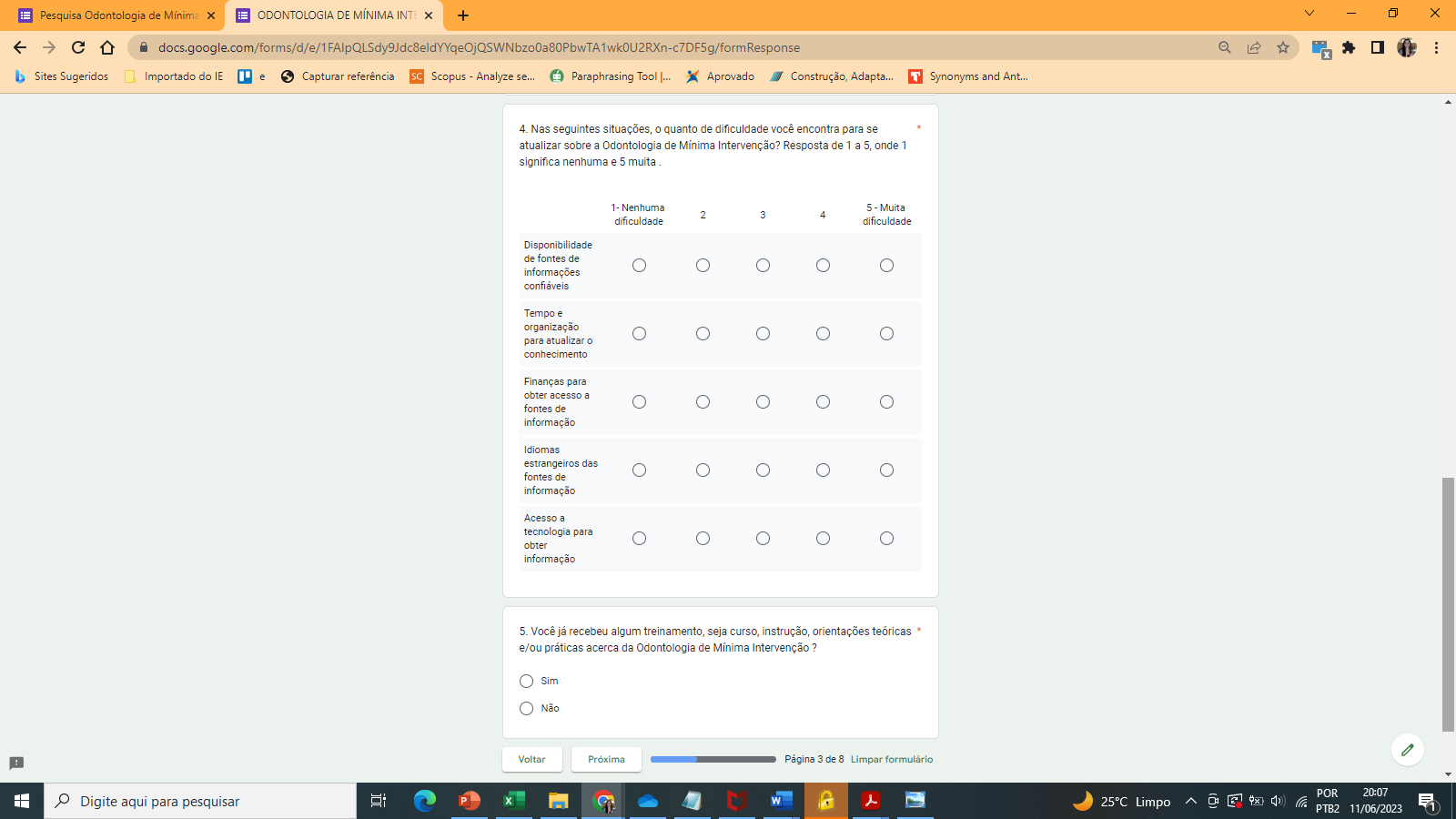


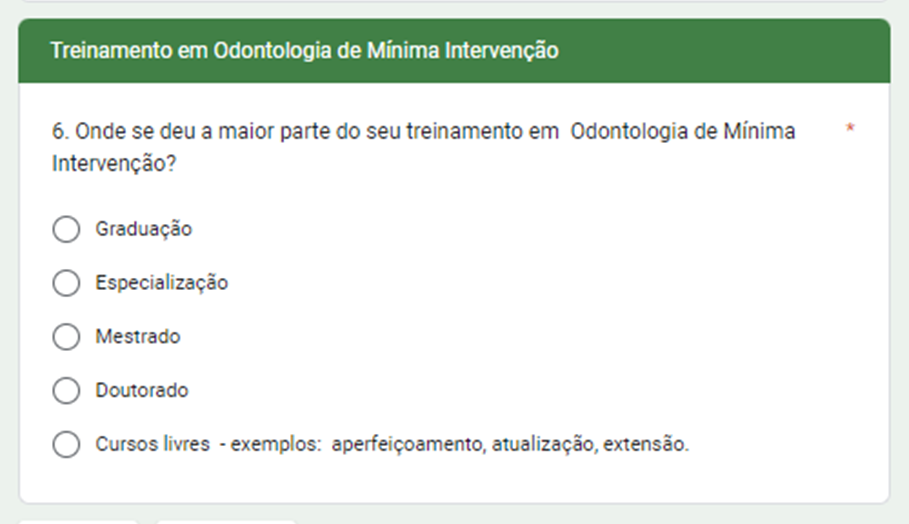


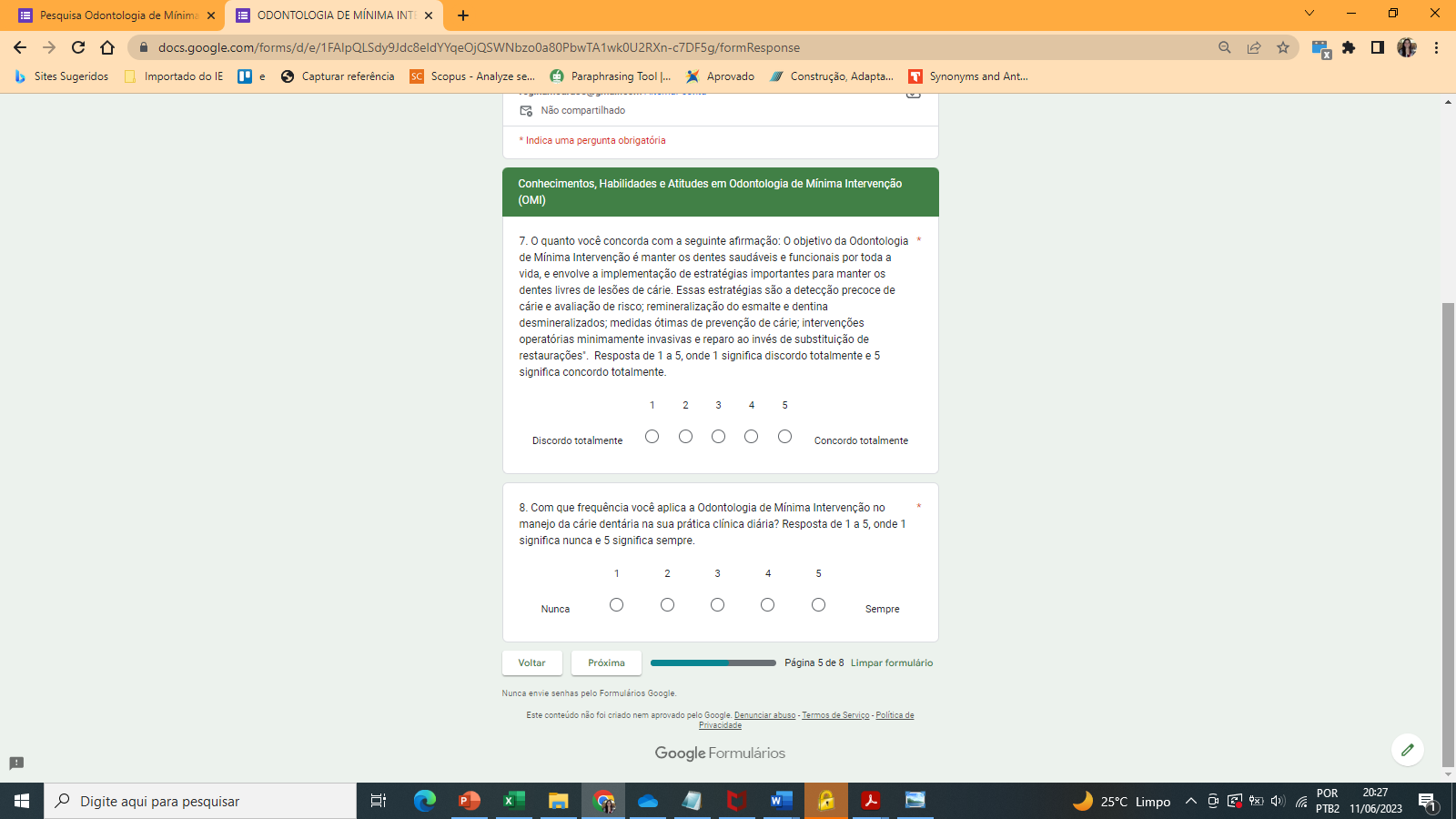


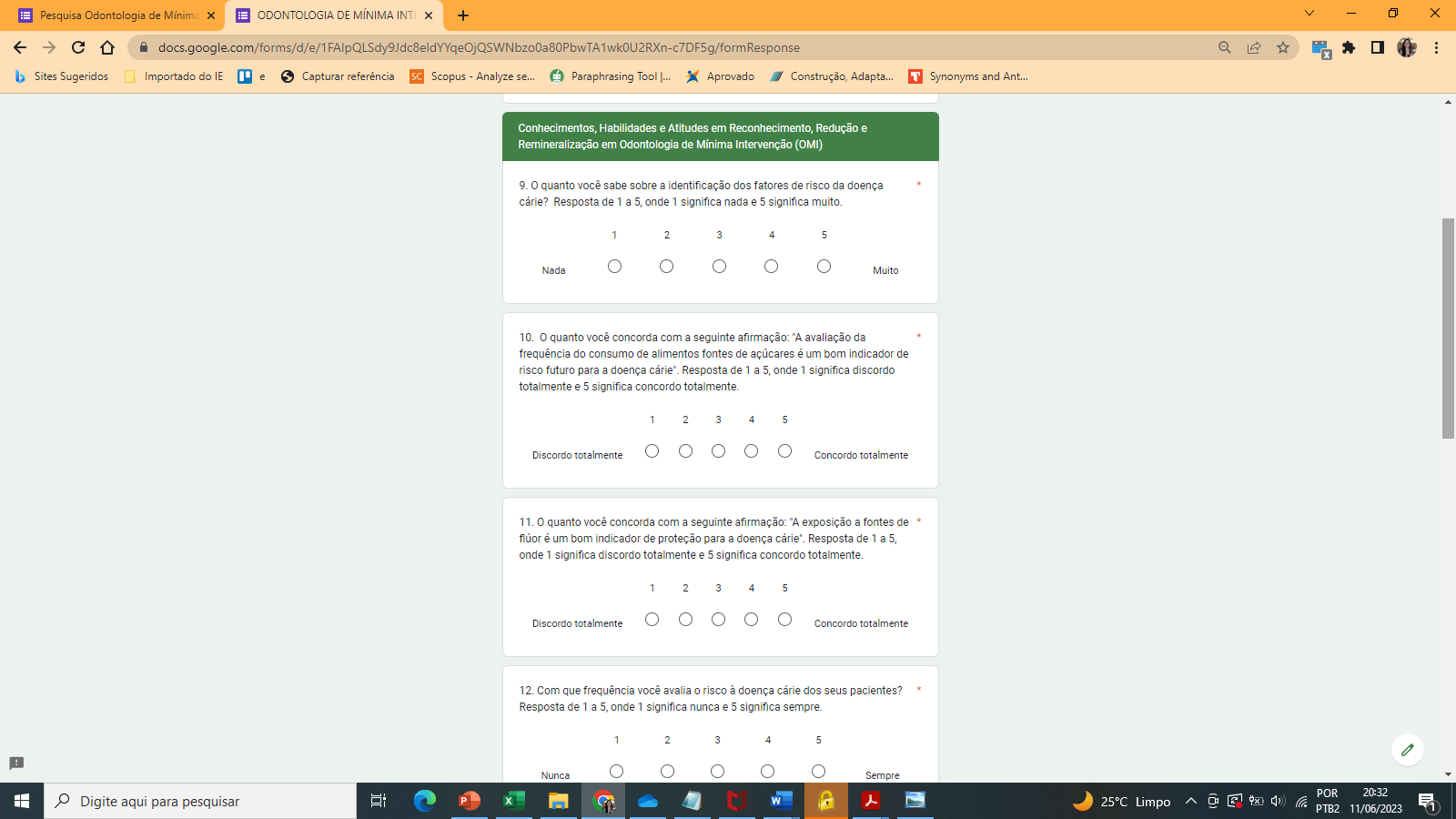


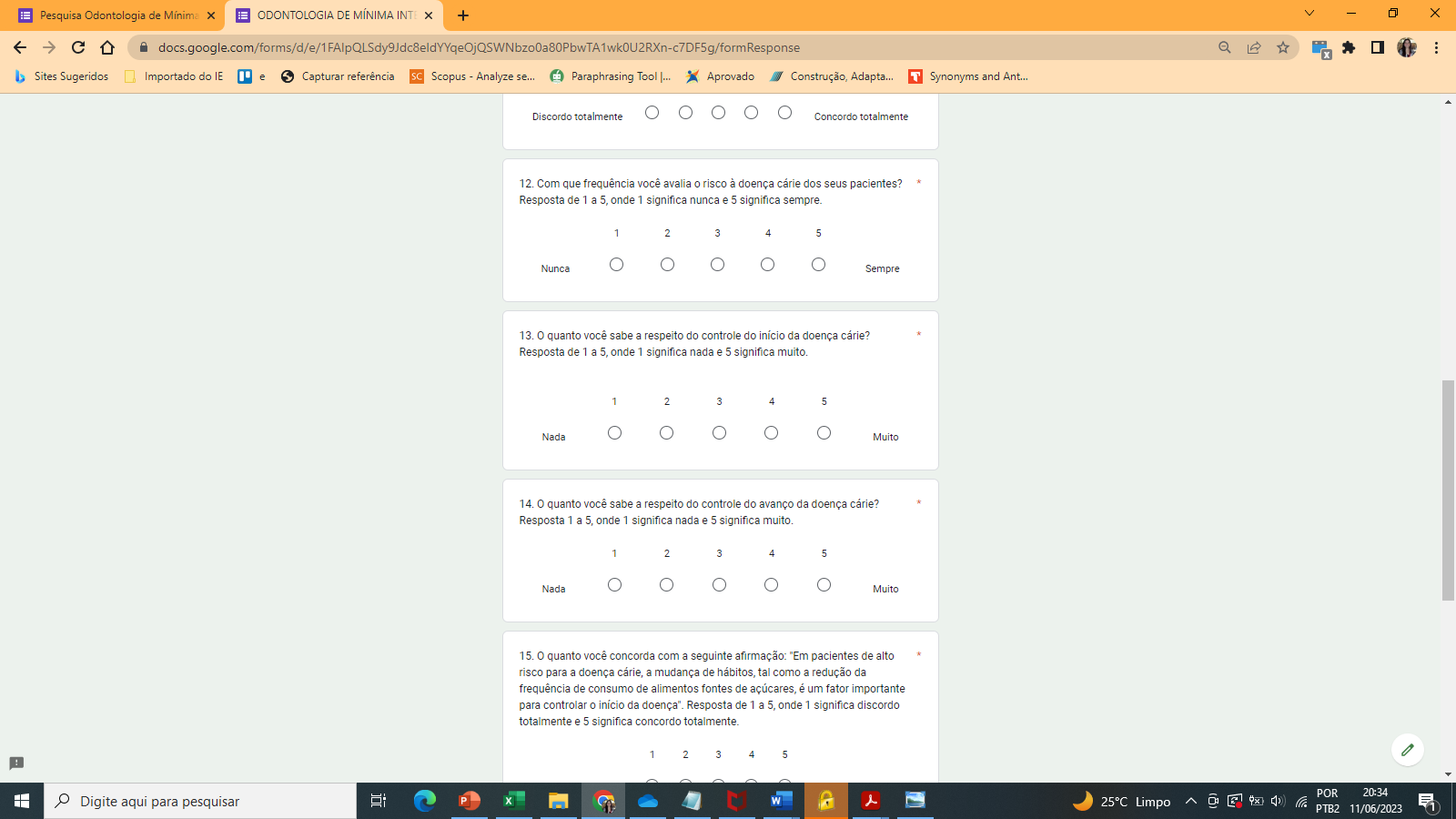


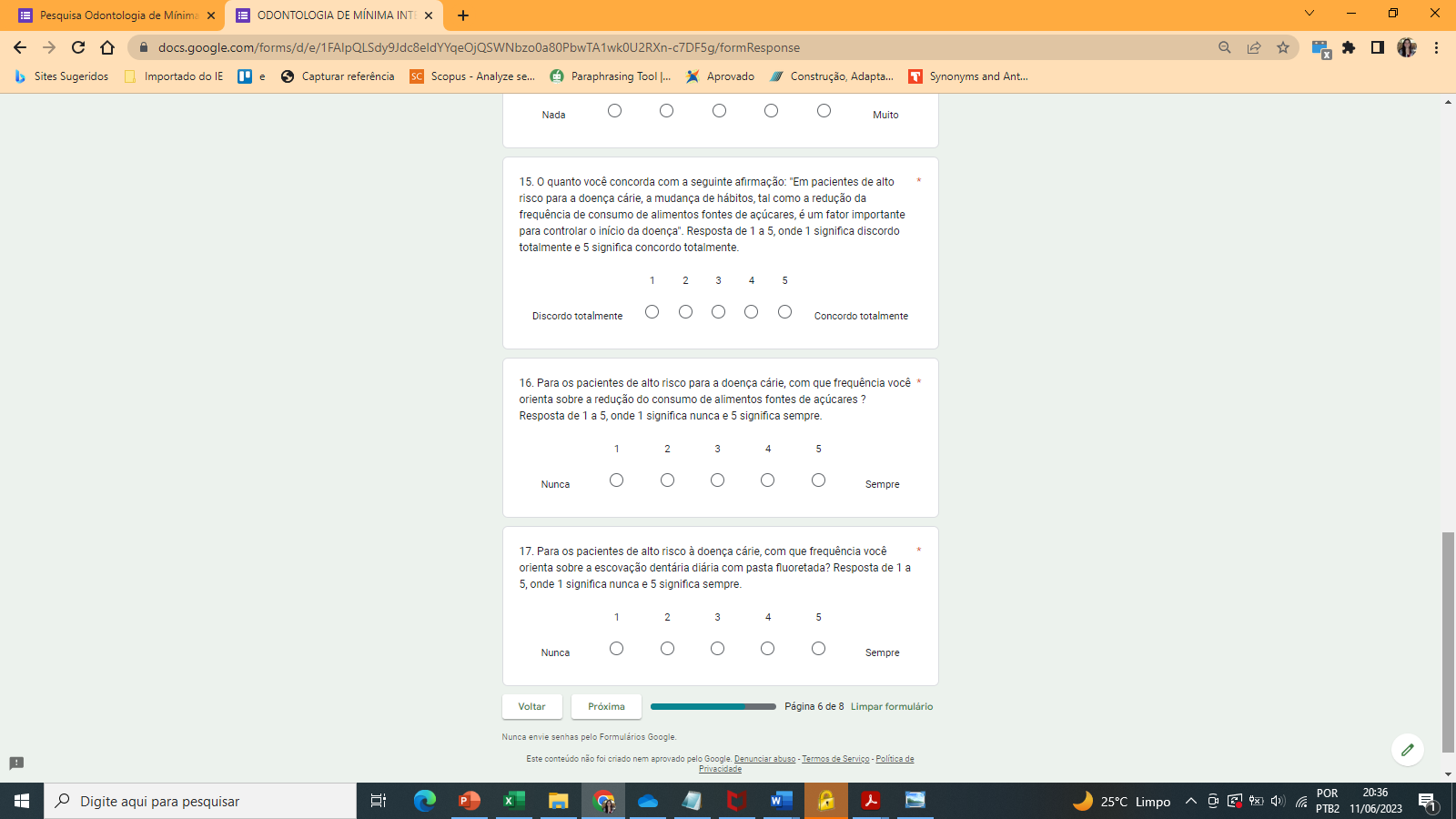


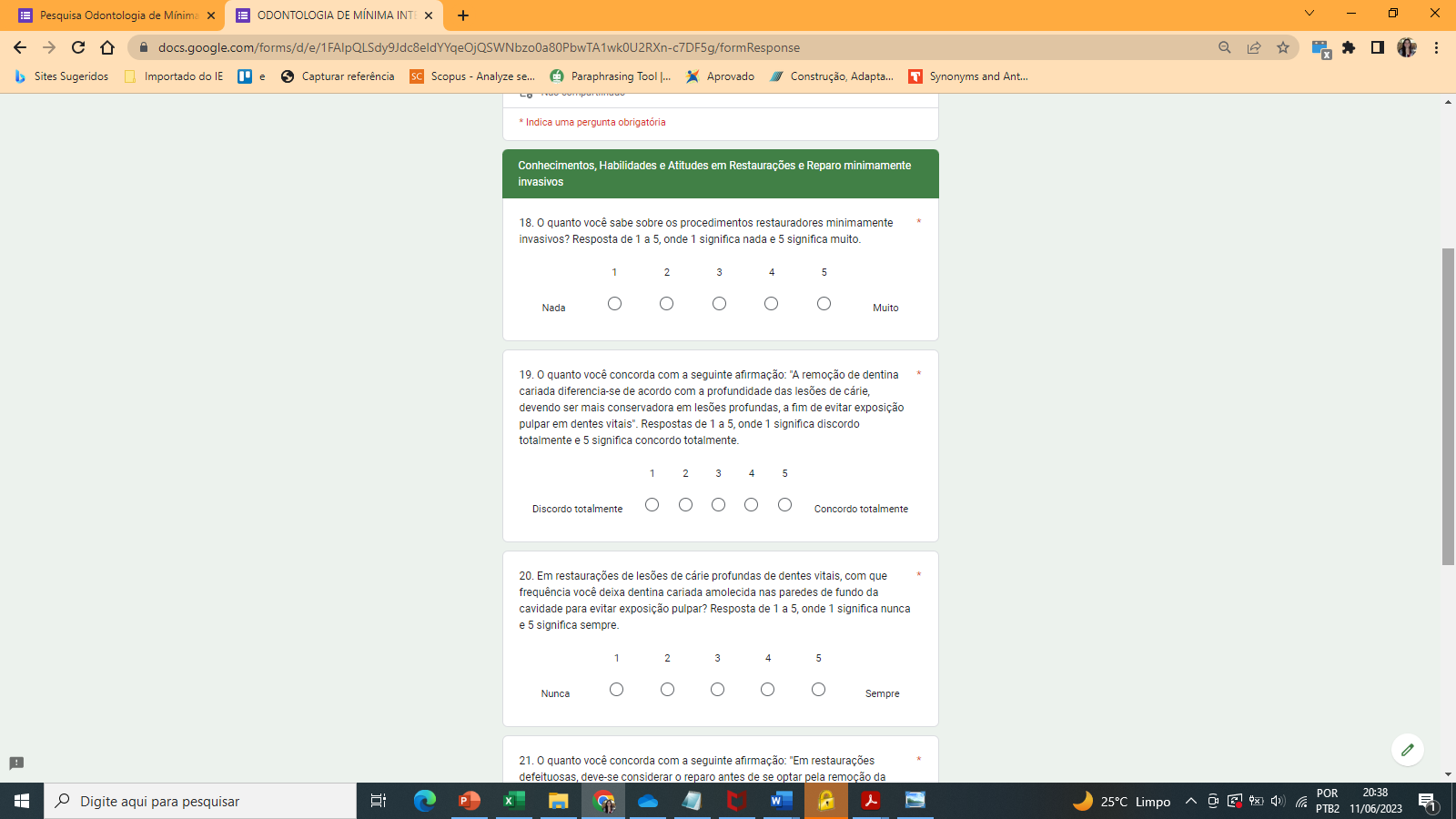


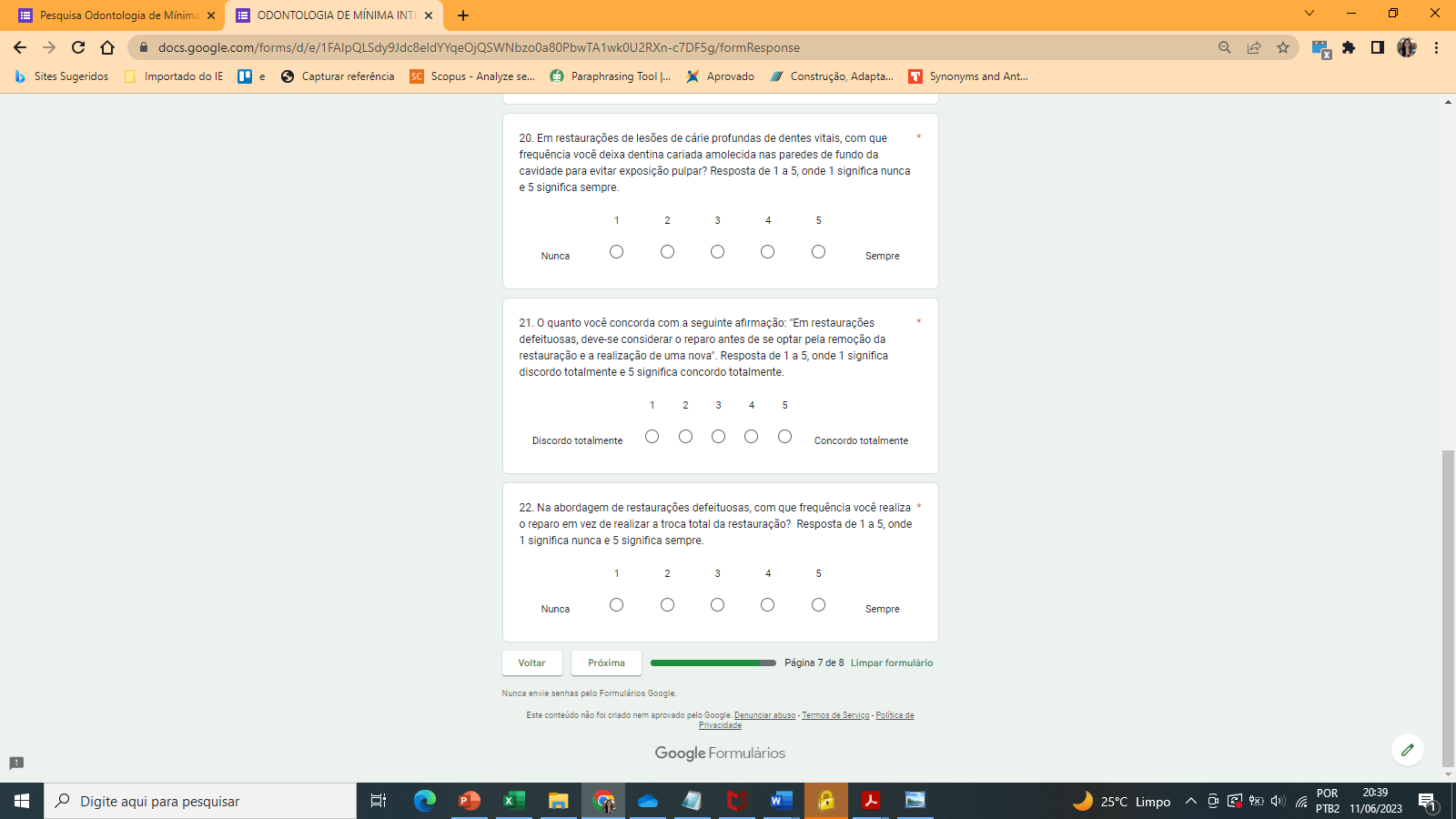


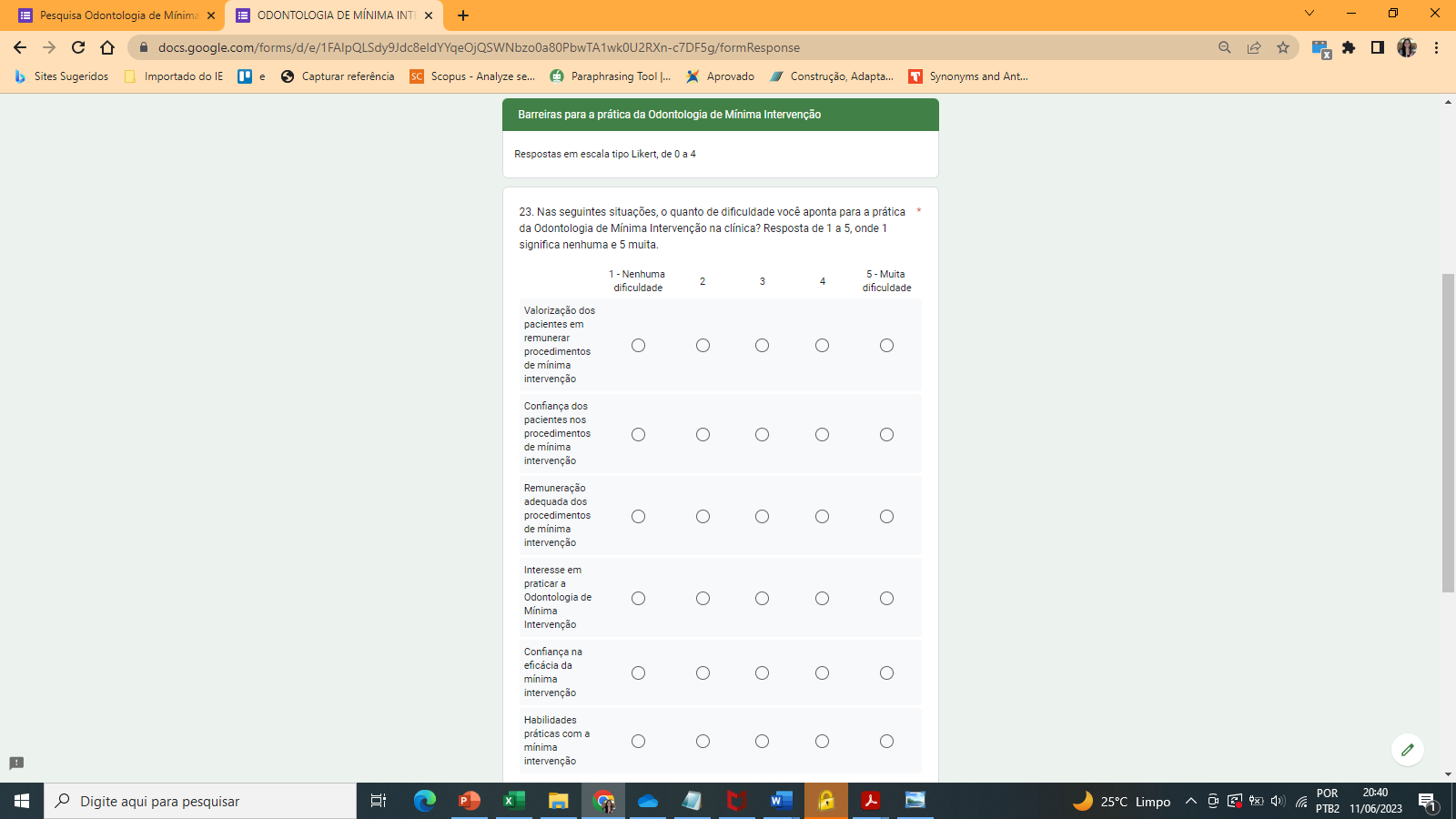


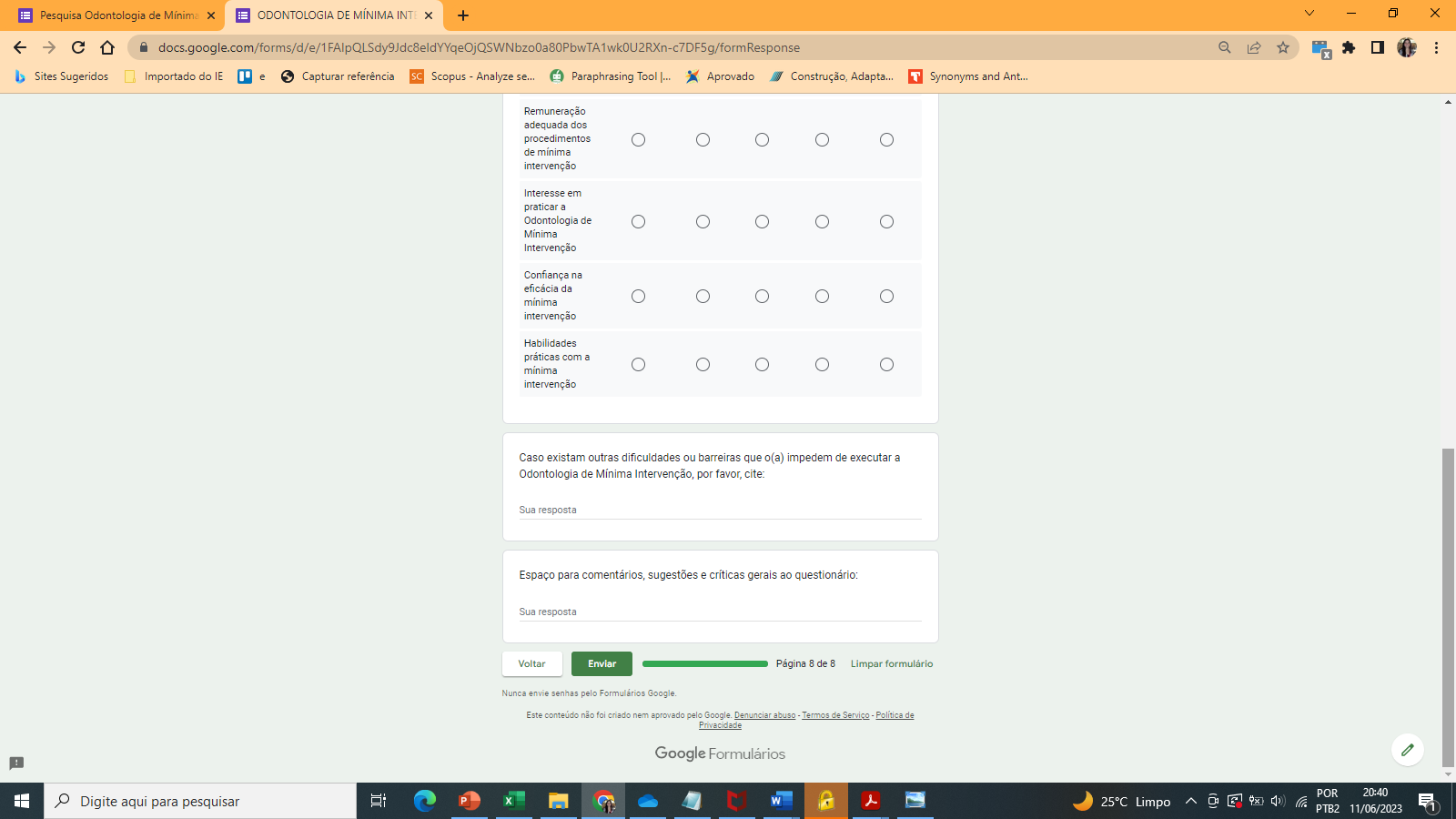

Supplement: supplementary material [file 1807-3107-bor-40-e041-S1.docx]
