# Supplementary material for: Dentists' knowledge, skills, attitudes and barriers towards minimal intervention dentistry
Source: Braz Oral Res. 2026 Jul 24;40:e041. doi: 10.1590/1807-3107bor-2026.vol40.041 (PMC13399977; doi:10.1590/1807-3107bor-2026.vol40.041)
Supplement: Supplementary file 2 [file 1807-3107-bor-40-e041-S2.docx]

## **APPENDIX S2 –**Description of the full statistical analysis

## **Frame 1:** Determination of variables

| **Name** | **Variable type** | **Variable treatment** |
| --- | --- | --- |
| Knowledge in MID | Continuous  (5 structured items, Likert scale from 1 to 5, ranging from 5 to 25 points; higher scores indicate higher assessed competence) | Dependent variable (analysis 1) |
| Skills and Attitude in MID | Continuous  (10 structured items, Likert scale from 1 to 5, ranging from 10 to 50 points; higher scores indicate higher assessed competence) | Dependent variable (analysis 2) |
| Barrier for knowledge in MID | Continuous (5 structured items, Likert scale from 1 to 5, ranging from 5 to 25 points; higher scores indicate lower assessed competence) | Dependent variable (analysis 3) |
| Barrier for practice in MID | Continuous (6 structured items, Likert scale from 1 to 5, ranging from 6 to 30 points; higher scores indicate lower assessed competence) | Dependent variable (analysis 4) |
| Gender | Continuous | Independent variable |
| Administrative Region of Residence | Categorical (Plano Piloto; Other regions) | Independent variable |
| Years of experience | Continuous | Independent variable |
| Graduation Institution | Categorical (Public; Private (with or without scholarship) | Independent variable |
| Highest Academic Qualification | Categorical (Undergraduate; Postgraduate (specialization, master's, doctorate, post-doctorate) | Independent variable |
| Professional Role | Categorical (Teaching (undergraduate, postgraduate, research); Others) | Independent variable |
| Heard of MID | Categorical (Yes; No) | Adjustment variable |
| Information Seeking | Categorical (Yes (in any source); No) | Independent variable |
| Received training on MID | Categorical (Yes; No) | Adjustment variable |

MID=Minimal Intervention Dentistry

**Multiple linear regression to assess the factors that may influence knowledge, skills, and attitudes, as well as barriers to MID among dentists in the Federal District**

**Step 1:** Normality of dependent variables (knowledge, skills and attitudes, and barriers to knowledge, practice) and independent variables: none assumed normal distribution (p<0,001) (Shapiro-Wilk Test). Multiple linear regression was retained because the residuals of the multiple regression models with these variables showed a normal distribution through graphical analysis (FIELD, 2009). Additionally, there is no violation of other assumptions of multiple linear regression (WILLIAMS *et al.*, 2013). An assessment of how the variables are related was conducted through Spearman’s linear correlation.

**Step 2**: Simple linear regression.

**Step 3:** Multiple linear regression where all variables with a p-value ≤ 0.20 in simple regression were added to the multiple regression model, in addition to the adjustment variables “Heard of MID” and “Received training on MID”.

**Table 1:** Linear correlation between the average knowledge and skills/attitudes scores with independent variables (n=404)

| **Variables** | **Knowledge** | | **Skills and Attitudes** | |
| --- | --- | --- | --- | --- |
|  | **ρ** | **p valor** | **ρ** | **p valor** |
| Gender | 0.13 | **0.004** | 0.11 | **0.01** |
| Age (years) | -0.12 | **0.006** | 0.11 | **0.01** |
| Administrative Region of Residence | 0.04 | 0.17 | 0.13 | **0.003** |
| Years of experience | -0.14 | **0.002** | 0.10 | **0.02** |
| Graduation Institution | 0.11 | **0.01** | 0.22 | **<0.001** |
| Highest Academic Qualification | -0.05 | 0.15 | 0.007 | 0.44 |
| Professional role | 0.16 | **0.001** | 0.09 | **0.02** |
| Heard of MID | 0.23 | **<0.001** | 0.15 | **0.001** |
| Information seeking | 0.43 | **<0.001** | 0.36 | **<0.001** |
| Received training on MID | 0.52 | **<0.001** | 0.33 | **<0.001** |

Spearman’s linear correlation. MID=Minimal Intervention Dentistry

**Table 2:** Multiple linear regression for the association between knowledge in Minimal Intervention Dentistry (MID) and predictive variables (n=404).

| **Variable** | **Knowledge in MID** | | | |
| --- | --- | --- | --- | --- |
|  | **β IC (95%)**  **Unadjusted** | **p-value** | **β IC (95%)**  **Adjusted** | **p-value** |
| Gender |  |  |  |  |
| Female | 1.02 (0.28; 1.76) | **0.007** | 0.56 (-0.04; 1.16) | 0.06 |
| Male |  |  |  |  |
| Age (Years) | -0.04 (-0.07;-0.009) | **0.01** | 0.04 (-0.02; 0.11) | 0.22 |
| *Administrative Region of Residence* |  |  |  |  |
| Plano Piloto | 0.34 (-0.39; 1.07) | 0.35 |  |  |
| Other regions |  |  |  |  |
| Years of experience | -0.04 (-0.07; -0.01) | **0.003** | -0.06 (-0.13; 0.01) | 0.09 |
| *Graduation Institution* |  |  |  |  |
| Public | 0.74 (0.08; 1.40) | **0.02** | 0.56 (0.03; 1.09) | **0.03** |
| Private |  |  |  |  |
| *Highest Qualification* |  |  |  |  |
| Postgraduate | -0.49 (-1.42; 0.44) | 0.30 |  |  |
| Graduate |  |  |  |  |
| *Professional Activity* |  |  |  |  |
| Teaching/Research | 2.01 (0.81; 3.20) | **0.001** | 1.24 (0.27; 2.21) | **0.01** |
| Other |  |  |  |  |
| Heard of MID |  |  |  |  |
| Yes | 5.06 (3.01; 7.11) | **<0.001** | 1.00 (-0.78; 2.79) | 0.27 |
| No |  |  |  |  |
| Information seeking |  |  |  |  |
| Yes | 3.97 (3.17; 4.76) | **<0.001** | 2.35 (1.56; 3.14) | **<0.001** |
| No |  |  |  |  |
| Received training on MID |  |  |  |  |
| Yes | 3.89 (3.27; 4.51) | **<0.001** | 2.95 (2.31; 3.59) | **<0.001** |
| No |  |  |  |  |

R2: 0.38; Adjusted R2: 0.36. Bold indicates statistical significance. All independent variables with p ≤ 0.20 in simple regression were included in the adjusted model. "Heard of MID" and "Received training on MID" were considered adjustment variables. MID = Minimal Intervention Dentistry.

The analysis resulted in a statistically significant model [F (8, 395) = 30.24; p < 0.001; R2 0.38]. Graduation institution (standardized β = 0.08; t = 2.09; p = 0.03); professional activity (standardized β = 0.10; t = 2.51; p = 0.01); information seeking (standardized β = 0.26; t = 5.85; p < 0.001) and receiving training on MID (standardized β = 0.39; t = 9.09; p < 0.001) are predictors of knowledge on MID.

It is expected that there is a higher average of knowledge on MID among dentists in the Federal District who studied in public institutions by 0.56 points compared to those who studied in private institutions (95% CI 0.03; 1.09; p = 0.03). Additionally, dentists working in teaching/research showed, on average, 1.24 points higher knowledge on MID (95% CI 0.27; 2.21; p = 0.01) compared to those working in private clinics, public clinics, and administration. Furthermore, dentists actively seeking information and those who received training on MID showed, on average, higher knowledge on MID (2.35; 95% CI 1.56; 3.14; p < 0.001 and 2.95; 95% CI 2.31; 3.59; p < 0.001, respectively). The results indicate that this model can explain 36% of the variability of knowledge on MID.

Parameters of multiple linear regression:

- Independence of Residuals: Durbin-Watson test equals 1.92 (acceptable between 1.5 and 2.5); thus, the residuals are independent.
- Absence of Multicollinearity:

- Variance Inflation Factor (VIF): gender 1.03; age 8.07; years of experience 8.69; graduation institution 1.02; professional activity 1.01; heard of MID 1.13; information seeking 1.26; received training on MID 1.21 (VIF > 10 can cause multicollinearity problems (high correlations between explanatory variables)) (GUJARATI, 2011)); thus, there is no multicollinearity.

- Tolerance: gender 0.96; age 0.11; years of experience 0.11; graduation institution 0.98; professional activity 0.98; heard of MID 0.88; information seeking 0.79; received MID training 0.82 (Tolerance value > 0.10 indicates the absence of multicollinearity); thus, there is no multicollinearity.

- Absence of Outliers: Standardized predicted values (-2.95; 1.54) and standardized residuals (-3.06; 2.49) (reference values between -3.00 and 3.00); thus, there are no outliers. Additionally, Cook's Distance (0.00; 0.03), values greater than 1 indicate significant influence.
- Normality of Residuals: Graphical analysis (Figure 1).


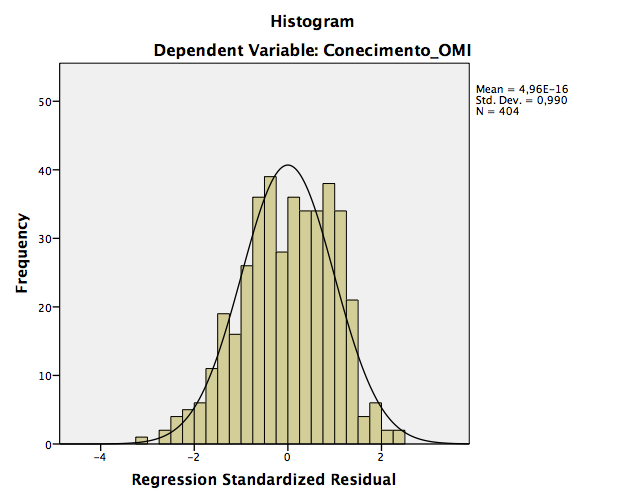


A


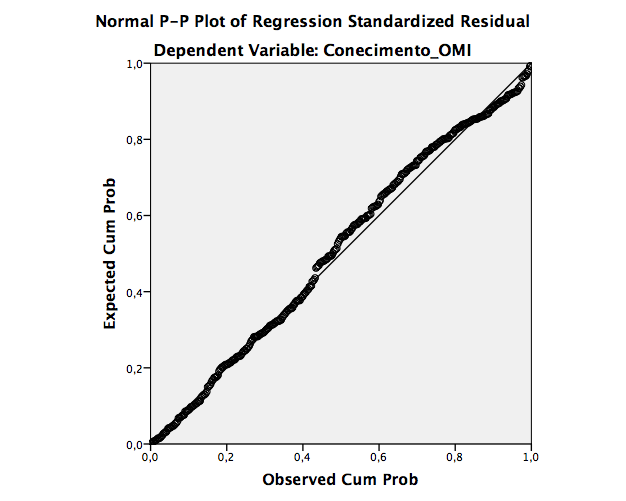


B

Figure 1 A and B. "Normality of the residuals in the multiple linear regression between knowledge on Minimal Intervention Dentistry (MID) and independent variables.

Homoscedasticity: Equal variances of the residuals; graphical analysis (Figure 2).


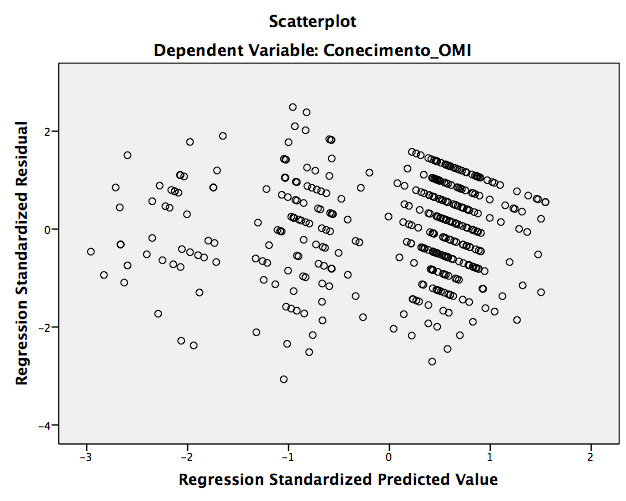


Figure 2. Homoscedasticity of the residuals from the multiple linear regression between knowledge in MID and independent variables.

Table 3: Multiple linear regression for the association between skill and attitude in MID and the predictor variables (n=404).

| **Variable** | **Skill and Attitude in MID** | | | |
| --- | --- | --- | --- | --- |
|  | **β CI (95%)**  **Unadjusted** | **p-value** | **β CI (95%)**  **Adjusted** | **p-value** |
| Gender |  |  |  |  |
| Female | 1.43 (0.16; 2.70) | **0.02** | 1.22 (0.07; 2.37) | **0.03** |
| Male |  |  |  |  |
| Age (years) | 0.06 (0.01; 0.11) | **0.02** | 0.12 (-0.01; 0.26) | 0.08 |
| Administrative Region of Residence |  |  |  |  |
| Plano Piloto | 1.72 (0.48; 2.97) | **0.007** | 0.59 (-0.56; 1.76) | 0.31 |
| Other Regions |  |  |  |  |
| Years of experience | 0.05 (0.001; 0.10) | **0.04** | -0.04 (-0.17; 0.09) | 0.55 |
| Undergraduate Institution |  |  |  |  |
| Public | 2.57 (1.47; 3.68) | **<0.001** | 2.17 (1.15; 3.18) | **<0.001** |
| Private |  |  |  |  |
| Highest Degree |  |  |  |  |
| Postgraduate | 0.12 (-1.47; 1.71) | 0.88 |  |  |
| Undergraduate |  |  |  |  |
| Professional Activity |  |  |  |  |
| Teaching/Research | 1.99 (-0.07; 4.05) | **0.05** | 0.86 (-0.99; 2.72) | 0.36 |
| Others |  |  |  |  |
| Heard abou MID |  |  |  |  |
| Yes | 5.73 (2.17; 9.29) | **0.002** | 0.83 (-2.55; 4.21) | 0.62 |
| No |  |  |  |  |
| Information Seeking |  |  |  |  |
| Yes | 3.13 (3.70; 6.56) | **<0.001** | 3.06 (1.55; 4.56) | **<0.001** |
| No |  |  |  |  |
| Received MID Training |  |  |  |  |
| Yes | 4.19 (3.02; 5.37) | **<0.001** | 3.40 (2.18; 4.62) | **<0.001** |
| No |  |  |  |  |

R² 0.23; Adjusted R² 0.22. Bold indicates statistical significance. All independent variables with p ≤ 0.20 in simple regression were included in the adjusted model. The variables "Heard about MID" and "Received MID Training" were considered adjustment variables. MID= Minimum Intervention Dentistry.

The analysis resulted in a statistically significant model [F (9, 394) = 13.74; p < 0.001; R² 0.23]. Gender (standardized β = 0.09; t = 11.88; p = 0.03); undergraduate institution (standardized β = 0.18; t = 4.22; p < 0.001); information seeking (standardized β = 0.19; t = 4.00; p < 0.001), and receiving MID training (standardized β = 0.26; t = 5.49; p < 0.001) are predictors of skills and attitudes in MID.

It is expected that there is a higher mean in the score of skills and attitudes in MID among female dentists in the Federal District (1.22; 95% CI 0.07; 2.37; p = 0.03), as well as among those who studied in public institutions (2.17; 95% CI 1.15; 3.18; p < 0.001). Additionally, dentists who seek information and those who received MID training presented, on average, higher results in skills and attitudes in MID (3.06; 95% CI 1.55; 4.56; p < 0.001 and 3.40; 95% CI 2.18; 4.62; p < 0.001; respectively). The results indicate that this model can explain 22% of the variability of skills and attitudes in MID.

Multiple linear regression parameters:

• Residual Independence: Durbin-Watson test equal to 2.01 (acceptable between 1.5 and 2.5); therefore, the residuals are independent.

• Absence of Multicollinearity:

- Variance Inflation Factor (VIF): gender 1.03; age 8.77; residential region 1.10; years of experience 8.74; undergraduate institution 1.02; professional activity 1.03; heard about MID 1.13; information seeking 1.26; received MID training 1.23 (VIF > 10 can cause multicollinearity problems (high correlations between explanatory variables)) (GUJARATI, 2011); therefore, there is no multicollinearity.

- Tolerance: gender 0.96; age 0.11; residential region 0.90; years of experience 0.11; undergraduate institution 0.95; professional activity 0.96; heard about MID 0.88; information seeking 0.79; received MID training 0.80 (Tolerance value > 0.10 indicates absence of multicollinearity); therefore, there is no multicollinearity.

- Absence of outliers: Standardized predicted values (-3.04; 2.40) and standardized residuals (-4.50; 2.46) (reference values between -3.00 and 3.00); therefore, there are outliers. Additionally, Cook's Distance (0.00; 0.74), values greater than 1 indicate significant influence.
- Normality of residuals: Graphical analysis (Figure 3).


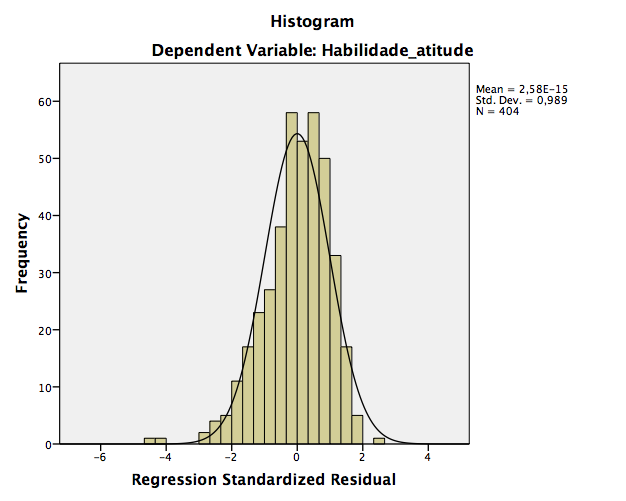


A


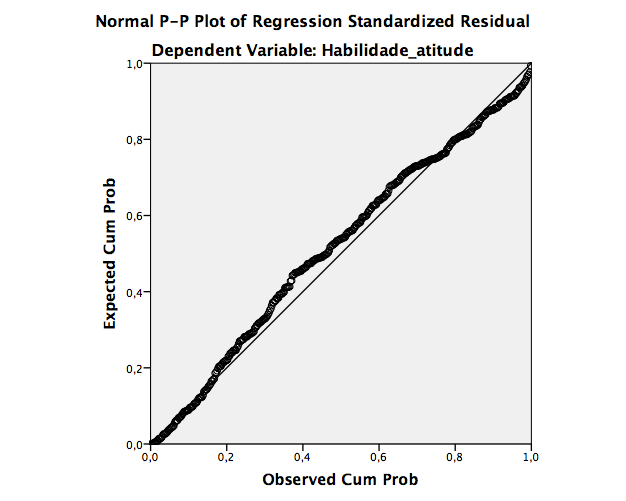


B

Figure 3A and 3B. Normality of the residuals from the multiple linear regression between skills and attitudes in OMI and independent variables.

- Homoscedasticity: Equal variances of the residuals; graphical analysis (Figure 4).4).


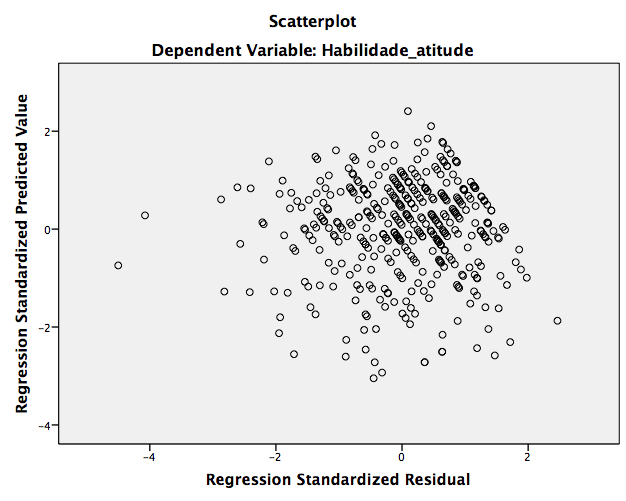


Figure 4. Homoscedasticity of the residuals from the multiple linear regression between skills and attitudes in OMI and independent variables.

**Table 4:** Linear correlation between mean barriers to knowledge and mean barriers to practice in MID with independent variables (n=404).

| **Variables** | **Barriers**  **Knowledge** | | **Barriers**  **Practice** | |
| --- | --- | --- | --- | --- |
|  | **ρ** | **p valor** | **ρ** | **p valor** |
| Gender | 0.06 | 0.08 | -0.02 | 0.31 |
| Age (Years) | 0.03 | 0.25 | -0.13 | **0.003** |
| Administrative Region of Residence | -0.01 | 0.40 | -0.09 | **0.03** |
| Years of experience | 0.01 | 0.35 | -0.15 | **0.001** |
| Undergraduate institution | -0.13 | **0.003** | -0.04 | 0.21 |
| Highest degree | -0.10 | **0.01** | -0.09 | **0.02** |
| Professional Activity | -0.19 | **<0.001** | -0.07 | 0.08 |
| Heard abou MID | 0.001 | 0.49 | -0.02 | 0.33 |
| Information seeking | -0.03 | 0.25 | -0.09 | **0.02** |
| Received MID training | -0.16 | **<0.001** | -0.12 | **0.005** |

Linear correlation of Spearman. MID = Minimum Intervention Dentistry.

**Table 5:** Multiple linear regression for the association between barriers to knowledge in MID and predictive variables (n=404).

| **Variable** | **Barriers to Knowledge in MID** | | | |
| --- | --- | --- | --- | --- |
|  | **β CI (95%)**  **Unadjusted** | **p-value** | **Β CI (95%)**  **Ajusted** | **p-value** |
| Gender |  |  |  |  |
| Female | 0.59 (-0.25; 1.44) | 0.16 | 0.73 (-0.08; -1.56) | 0.08 |
| Male |  |  |  |  |
| Age (years) | 0.01 (-0,02;0,04) | 0.51 |  |  |
| Administrative Region of Residence |  |  |  |  |
| Plano Piloto | -0.10 (-0,93; 0,72) | 0.08 | 0.31 (-0.53; 1.15) | 0.46 |
| Other Regions |  |  |  |  |
| Years of experience | 0.007 (-0.02; 0.04) | 0.07 | 0.01 (-0.02; 0.04) | 0.54 |
| Undergraduate institution |  |  |  |  |
| Public | -1.05 (-1.80; -0.31) | **0.005** | -0.88 (-1.61; -0.15) | **0.01** |
| Private |  |  |  |  |
| Maior titulação |  |  |  |  |
| Postgraduate | -1.11 (-2.17; -0.06) | **0.03** | -1.11 (-2.22; -0.003) | **0.04** |
| Undergraduate |  |  |  |  |
| Professional Activity |  |  |  |  |
| Teaching/Research | -2.71 (-4.06; -1.35) | **<0.001** | -2.56 (-3.90; -1.23) | **<0.001** |
| Others |  |  |  |  |
| Heard about OMI |  |  |  |  |
| Yes | 0.01 (-2.38; 2.41) | 0.99 | 1.10 (-1.25;1.45) | 0.36 |
| No |  |  |  |  |
| Information Seeking |  |  |  |  |
| Yes | -0.34 (-1.34; 0.66) | 0.50 |  |  |
| No |  |  |  |  |
| Received OMI Training |  |  |  |  |
| Yes | -1.50 (-2.32; -0.69) | **<0.001** | -1.52 (-2.35; -0.68) | **<0.001** |
| No |  |  |  |  |

R² 0.10; Adjusted R² 0.08. Bold indicates statistical significance. All independent variables with p ≤ 0.20 in simple regression were included in the adjusted model. The variables "Heard about MID" and "Received MID Training" were considered adjustment variables. MID = Minimum Intervention Dentistry.

The analysis resulted in a statistically significant model [F (8, 395) = 5.49; p < 0.001; R² 0.08]. Undergraduate institution (standardized β = 0.11; t = -2.38; p = 0.01); highest degree (standardized β = 0.10; t = -1.97; p = 0.04); professional activity (standardized β = -0.18; t = -3.78; p < 0.001), and receiving MID training (standardized β = -0.18; t = -3.58; p < 0.001) are predictors of barriers to knowledge in MID.

Dentists from the Federal District who studied in public institutions have, on average, fewer barriers to knowledge in the questionnaire scores compared to those who studied in private institutions (-0.88, 95% CI -1.61; -0.15; p = 0.01). The same is true for dentists who have postgraduate education as their highest degree, work in teaching/research, or have received MID training (-1.11, 95% CI -2.22; -0.003; p = 0.04; -2.56, 95% CI -3.90; -1.23; p < 0.001 and -1.52, 95% CI -2.35; -0.68; p < 0.001 respectively). The results indicate that this model can explain 8% of the variability in barriers to knowledge in MID.

Multiple linear regression parameters:

- Residual Independence: Durbin-Watson test equal to 1.84 (acceptable between 1.5 and 2.5); therefore, the residuals are independent.
- Absence of Multicollinearity:
- Variance Inflation Factor (VIF): gender 1.03; residential region 1.10; years of experience 1.30; undergraduate institution 1.03; highest degree 1.19; professional activity 1.02; heard about OMI 1.05; received OMI training 1.11 (VIF > 10 can cause multicollinearity problems (high correlations between explanatory variables)) (GUJARATI, 2011); therefore, there is no multicollinearity.
- Tolerance: gender 0.97; residential region 0.90; years of experience 0.76; undergraduate institution 0.96; highest degree 0.83; professional activity 0.97; heard about OMI 0.94; received OMI training 0.89 (Tolerance value > 0.10 indicates absence of multicollinearity); therefore, there is no multicollinearity.
- Absence of outliers: Standardized predicted values (-2.87; 2.20) and standardized residuals (-1.95; 3.40) (reference values between -3.00 and 3.00); therefore, there are outliers. Additionally, Cook's Distance (0.00; 0.03), values greater than 1 indicate significant influence.
- Normality of residuals: Graphical analysis (Figure 5).


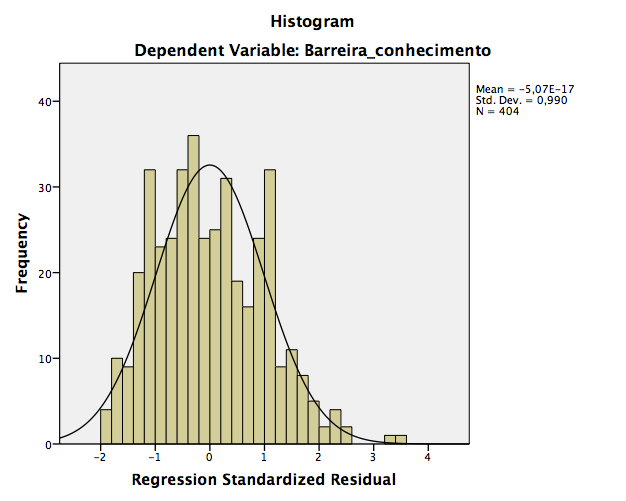


A


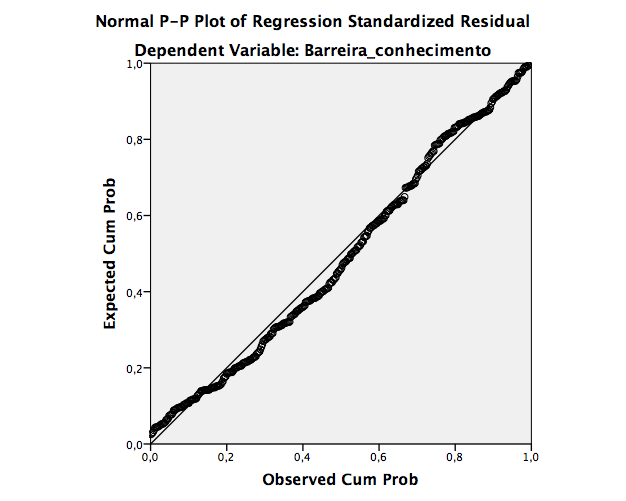


B

Figure 5 A and B. Normality of residuals of the multiple linear regression between knowledge in MID and independent variables.

- Homoscedasticity: Equal variances of residuals; graphical analysis (Figure 6).
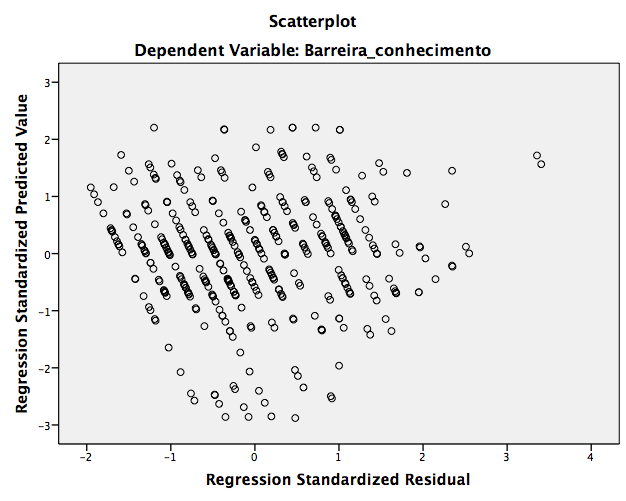


Figure 6. Homoscedasticity of the residuals of the multiple linear regression between knowledge in MID and independent variables.

**Table 6**: Multiple linear regression for the association between barriers to practice in MID and the predictive variables (n=404).

| **Variable** | **Barriers to Practice in MID** | | | |
| --- | --- | --- | --- | --- |
|  | **β IC (95%)**  **Unadjusted** | **p-value** | **β IC (95%)**  **Adjusted** | **p-value** |
| Gender |  |  |  |  |
| Female | -0.32 (-1.64; 0.99) | 0.63 |  |  |
| Male |  |  |  |  |
| Age (years) | -0.07 (-0.13;-0.02) | **0.006** | -0.07 (-0.14; 0.18) | **0.01** |
| Residential Administrative Region |  |  |  |  |
| Plano Piloto | -1.24 (-2.53; 0.05) | 0.06 | -0.49 (-1.82; 0.83) | 0.46 |
| Other regions |  |  |  |  |
| Years of Experience (years) | -0.08 (-0.13; -0.02) | **0.003** |  |  |
| Undergraduate Institution |  |  |  |  |
| Public | -0.47 (-1.64; 0.69) | 0.42 |  |  |
| Private |  |  |  |  |
| Highest Degree |  |  |  |  |
| Postgraduate | -1.63 (-3.27; 0.01) | **0.05** | -0.85 (-2.57; 0.87) | 0.33 |
| Undergraduate |  |  |  |  |
| Professional Activity |  |  |  |  |
| Teaching/Research | -1.52 (-3.66; 0.61) | 0.16 | -1,17 (-3.31; 0.96) | 0.28 |
| Others |  |  |  |  |
| Heard about MID |  |  |  |  |
| Yes | -0.81 (-4.53; 2.91) | 0.66 | 0.68 (-3.20; 4.57) | 0.72 |
| No |  |  |  |  |
| Information Seeking |  |  |  |  |
| Yes | -1.53 (-3.09; 0.02) | **0.05** | -0.67 (-2.40; 1.04) | 0.43 |
| No |  |  |  |  |
| Received MID Training |  |  |  |  |
| Yes | -1.67 (-2.94; -0.39) | **0.01** | -1.84 (-3.25; -0.44) | **0.01** |
| No |  |  |  |  |

R^2^: 0.05; Adjusted R^2^: 0.03. Statistically significant variables are shown in bold. All independent variables with p ≤ 0.20 in simple regression were included in the adjusted model. The variables "Heard about MId" and "Received MID Training" were considered adjustment variables. MID = Minimal Intervention Dentistry.

The analysis resulted in a statistically significant model [F (8, 395) = 2.91; p <0.001; R2 0.03]. Age (standardized β = -0.14; t = -2.56; p = 0.01) and receiving training in MI (standardized β = -0.14; t = -2.58; p = 0.01) are predictors of barriers to practice in MI.

The results indicate that the younger the dentist, the higher the average scores on barriers to practice (-0.07, 95% CI -0.14; 0.18; p = 0.01). Dentists from the DF who have already received MI training have, on average, fewer barriers to practice in the questionnaire (-1.84, 95% CI -3.25; -0.44; p = 0.01). The results indicate that this model can explain 3% of the variability of barriers to practice in MI (the model is not very explanatory).

Multiple linear regression parameters:

- Independence of residuals: Durbin-Watson test equal to 1.93 (acceptable between 1.5 and 2.5); hence, the residuals are independent.
- Absence of Multicollinearity:

- Variance Inflation Factor (VIF): age 1.24; housing region 1.09; highest degree 1.12; professional activity 1.03; heard of MI 1.13, information seeking 1.25, and received MI training 1.23 (VIF >10 can cause multicollinearity problems); hence, there is no multicollinearity.

- Tolerance: age 0.80; housing region 0.91; highest degree 0.88; professional activity 0.96; heard of MI 0.88, information seeking 0.79, and received MI training 0.80 (Tolerance value > 0.10 indicates absence of multicollinearity); hence, there is no multicollinearity. Experience time had a tolerance of 0.10 and was removed from the model.

- Absence of outliers: Standardized predicted values (-3.13; 3.02) and standardized residuals (-1.99; 2.52) (reference values between -3.00 and 3.00); hence there are outliers. In addition, Cook's Distance (0.00; 0.06), values greater than 1 indicate significant influence.
- Normality of residuals: Graphical analysis (Figure 7).


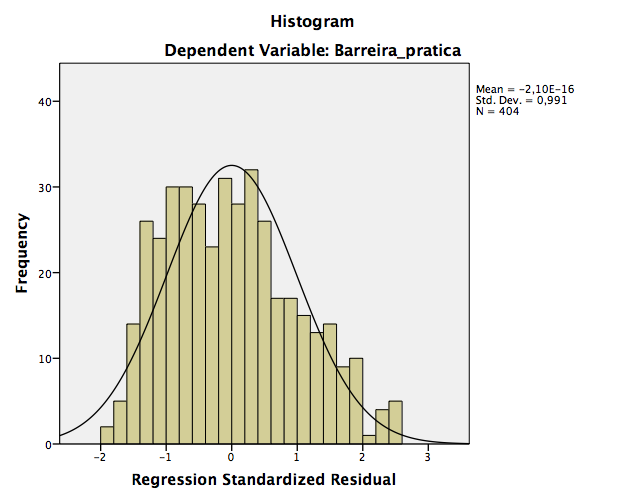


A


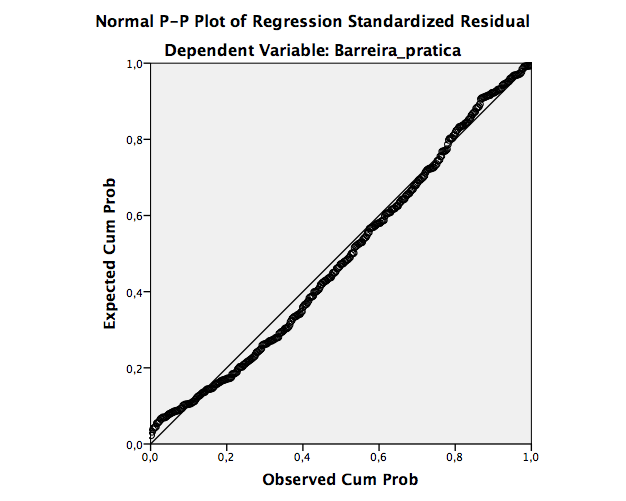


B

Figure 7 A and B. Normality of residuals from multiple linear regression between MI knowledge and independent variables.

• Homoscedasticity: Equal variances of residuals; graphical analysis (Figure 8).
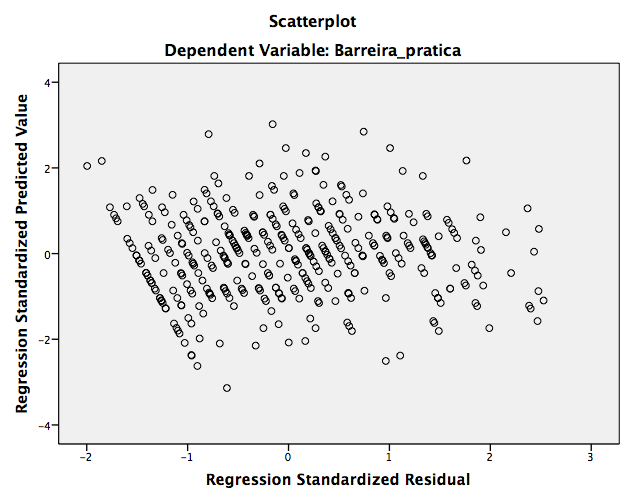


Figure 8. Homoscedasticity of residuals from multiple linear regression between MID knowledge and independent variables.

**REFERENCES FOR THE STATISTICAL ANALYSIS:**

Field, A. Descobrindo a estatística usando o SPSS-5. Penso Editora, 2009.

Gujarati, DN; Porter, DC. Econometria Básica-5. Amgh Editora, 2011.

Williams, MN; Grajales, CAG; Kurkiewicz, D. Assumptions of multiple regression: Correcting two misconceptions. 2013. ISSN 1531-7714.
