# Supplementary material for: Dentists' knowledge, skills, attitudes and barriers towards minimal intervention dentistry
Source: Braz Oral Res. 2026 Jul 24;40:e041. doi: 10.1590/1807-3107bor-2026.vol40.041 (PMC13399977; doi:10.1590/1807-3107bor-2026.vol40.041)
Supplement: Supplementary file 3 [file 1807-3107-bor-40-e041-S3.docx]

**Appendix S3** **–** Database Search Strategy.

| **Database** | **Search** |
| --- | --- |
| **Scopus** | TITLE-ABS-KEY ( ( "Minimally Invasive Dentistry"  OR  "Minimal Intervention Dentistry"  OR  "Evidence-Based Dentistry"  OR  "Dental Caries" )  AND  ( "Dentists"  OR  "Dentist"  OR  "Oral Medicine"  OR  "Dental Practitioners"  OR  "Dental Practitioner"  OR  "Oral Health-Care Worker"  OR  "Oral Health-Care Professionals" )  AND  ( "Health Knowledge, Attitudes, Practice"  OR  "Knowledge, Attitudes, Practice"  OR  "Health Behavior"  OR  "Health Behaviors"  OR  "Health-Related Behavior"  OR  "Health-Related Behaviors"  OR  "Attitude Of Health Personnel"  OR  "Health Personnel Attitude"  OR  "Health Personnel Attitudes"  OR  "Staff Attitude"  OR  "Staff Attitudes"  OR  "Attitude To Health"  OR  "Health Attitude"  OR  "Health Attitudes"  OR  "Beliefs"  OR  "Belief"  OR  "Evidence-practice gap"  OR  "Knowledge, Attitude, Skills"  OR  "Knowledge" ) )  AND  ( LIMIT-TO ( DOCTYPE ,  "ar" ) )  AND  ( LIMIT-TO ( SUBJAREA ,  "DENT" ) ) |
