# Supplementary material for: Dentists' knowledge, skills, attitudes and barriers towards minimal intervention dentistry
Source: Braz Oral Res. 2026 Jul 24;40:e041. doi: 10.1590/1807-3107bor-2026.vol40.041 (PMC13399977; doi:10.1590/1807-3107bor-2026.vol40.041)
Supplement: Supplementary file 4 [file 1807-3107-bor-40-e041-suppl.docx]

**UNIVERSIDADE DE BRASÍLIA**

**Faculdade de Ciências da Saúde**

**Programa de Pós-Graduação em Odontologia**

**
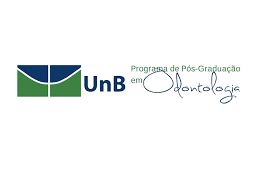
**

**DISSERTAÇÃO DE MESTRADO**

**COMPETÊNCIAS E BARREIRAS DOS CIRURGIÕES-DENTISTAS**

**ACERCA DA ODONTOLOGIA DE MINIMA INTERVENÇÃO**:

**REVISÃO SISTEMÁTICA E ESTUDO TRANSVERSAL**

**REGINA CARDOSO DE MOURA**

**BRASÍLIA**

**2023**

**REGINA CARDOSO DE MOURA**

**COMPETÊNCIAS E BARREIRAS DOS CIRURGIÕES-DENTISTAS**

**ACERCA DA ODONTOLOGIA DE MINIMA INTERVENÇÃO: REVISÃO SISTEMÁTICA E ESTUDO TRANSVERSAL**

Dissertação apresentada ao Programa de Pós-Graduação em Odontologia da Faculdade de Ciências da Saúde da Universidade de Brasília, como requisito parcial à obtenção do título de Mestre em Odontologia

**Orientador: Carla Massignan.**

**BRASÍLIA**

**2023**

**REGINA CARDOSO DE MOURA**

**COMPETÊNCIAS E BARREIRAS DOS CIRURGIÕES-DENTISTAS**

**ACERCA DA ODONTOLOGIA DE MINIMA INTERVENÇÃO:**

**REVISÃO SISTEMÁTICA E ESTUDO TRANSVERSAL**

Dissertação aprovada, como requisito parcial para obtenção do grau de Mestre em Odontologia, Programa de Pós-Graduação em Odontologia da Faculdade de Ciências da Saúde da Universidade de Brasília.

Data da defesa: 30/06/2023

Banca examinadora:

Prof. Dr. Carla Massignan (Presidente)

___________________________________________________________________

Prof. Dr. Aline de Almeida Neves

___________________________________________________________________

Prof. Dr. Daniela Raggio

*“Tua é, ó Senhor, a grandeza, e o poder, e a glória, e a vitória, e a majestade, porque teu é tudo quanto há no céu e na terra; teu é, ó Senhor, o reino, e tu te exaltaste como chefe sobre todos”.*

1 Crônicas 29:11

**RESUMO**

Os objetivos desse estudo foram: 1) avaliar os conhecimentos, atitudes e práticas (CAP) dos cirurgiões-dentistas sobre a Odontologia de Mínima Intervenção (OMI) por meio de uma revisão sistemática (RS) 2) construir e avaliar as propriedades psicométricas de um questionário para mensurar os conhecimentos, habilidades e atitudes dos cirurgiões-dentistas 3) avaliar os conhecimentos, habilidades, atitudes e barreiras dos cirurgiões-dentistas (CDs) do Distrito Federal (DF), sobre a Odontologia de Mínima Intervenção. 1) Foram incluídos estudos transversais que analisaram os resultados de CAP sobre os princípios de OMI. As buscas foram realizadas em janeiro de 2022. Dois revisores independentes selecionaram os estudos incluídos. A qualidade metodológica dos estudos foi avaliada por meio do Checklist de Avaliação Crítica do Instituto Joanna Briggs para Estudos de Prevalência. Metanálise de proporção foi realizada. O teste Q de Cochran foi usado para avaliar a heterogeneidade e estatística I^2^. Dos 2.079 estudos identificados, doze foram incluídos. A proporção combinada de conhecimento sobre OMI foi de 75,66% e de atitudes e prática foi de 47,95%. Os achados sugerem que o conhecimento dos dentistas sobre tópicos da OMI é aceitável, e as atitudes e as práticas precisam de melhorias. Os estudos carecem de uniformidade nos métodos e ainda há necessidade de mais estudos para elucidar o CAP dos cirurgiões-dentistas. 2) O questionário elaborado passou pela análise de uma equipe de brasileiros experts no tema. Foram realizados dois grupos focais e um pré-teste para averiguar a redação, sequência das perguntas e entendimento das questões. Os ajustes necessários foram realizados. Após aplicado, foi realizada Análise Fatorial Exploratória para avaliar a estrutura fatorial do questionário. Utilizou-se Análise Paralela com permutação aleatória dos dados. A Análise Paralela sugeriu que os itens do questionário se dividem em dois fatores. As cargas fatoriais dos itens foram adequadas em seus respectivos fatores. O questionário apresentou bons atributos na Análise Fatorial Exploratória e os achados sugerem evidência psicométrica do instrumento. Novos estudos são indicados para que sejam realizados novos testes, de forma a aumentar as evidências de validade. 3) Realizou-se um estudo transversal, baseado em questionário online autorreferido. Os dados foram analisados no SPSS, apresentados com estatística descritiva e inferencial, e foi realizada regressão linear múltipla. Uma amostra de 404 participantes foi obtida. A maioria dos respondentes, 74%, era do sexo feminino e um tempo de experiência médio de 12 anos (±10,9). O melhor escore foi obtido no item de prática acerca da orientação sobre a escovação dentária diária com pasta fluoretada em que 95,6% relataram realizá-la; o pior no item sobre o conhecimento acerca de restaurações minimamente invasivas, com 38,6%. Em relação as barreiras, o item sobre remuneração adequada dos procedimentos de mínima intervenção apresentou maior percentual de CDs que o avaliaram de forma desfavorável (38,3%). Este estudo mostrou uma proporção geral de conhecimentos, habilidades e atitudes dos CDs adequada, com exceção de atitudes em remoção seletiva de dentina cariada. Conclui-se que, em geral, os CDs do DF possuem competências adequadas em OMI, porém há dificuldades a serem enfrentadas para a prática da mínima intervenção.

**Palavras-chave:** Cárie Dentária. Odontologia Baseada em Evidências. Conhecimentos, Atitudes e Prática em Saúde. Odontologia de Mínima Intervenção.

**ABSTRACT**

The objectives of this study were: 1) to assess the knowledge, attitudes, and practices (KAP) of dentists regarding Minimal Intervention Dentistry (MID) with a systematic review (SR); 2) to build and evaluate the psychometric properties of a questionnaire to measure the knowledge, ability, and attitudes of dentists 3) to evaluate the knowledge, ability, attitudes, and barriers of dentists in the Federal District about Minimal Intervention Dentistry. Cross-sectional studies that analyzed KAP outcomes on MID principles were included. Searches were conducted in January 2022. Two independent reviewers selected the included studies. The methodological quality of studies was assessed by using the Joanna Briggs Institute Critical Assessment Checklist for Prevalence Studies. A proportion meta-analysis was performed. Cochran's Q test was used to assess heterogeneity and I^2^ statistics. Of the 2079 identified studies, twelve were included. The comparison of knowledge about MID was 75.66% and attitudes and practice were 47.95%. The findings suggest that the knowledge of dentists on MID topics is acceptable, and the attitudes and practices need improvements. The studies lacked uniformity in methods and there is still a need for more studies to elucidate the KAP of dentists worldwide. 2) The developed questionnaire was analyzed by a team of Brazilian specialists in the subject. Two focus groups and a pre-test were carried out to verify the writing, the sequence, and the understanding of questions. Any necessary adjustments were made. After application, Exploratory Factor Analysis was performed to assess the factorial structure of the questionnaire. Parallel analysis was used with a random permutation of the data. The Parallel Analysis suggested that the questionnaire items were divided into two factors. The factor loadings of the items were adequate in their respective factors. The questionnaire showed good attributes in the Exploratory Factor Analysis and the findings suggest psychometric evidence of the instrument. New studies are recommended to perform new tests and to increase the evidence of validity. 3) A cross-sectional study was carried out, based on a self-reported online questionnaire. Data were analyzed using SPSS, presented with descriptive and inferential statistics, and multiple linear regression was performed. A sample of 404 participants was obtained. Most respondents were female (74%) and had an average experience of 12 years (±10,9). The best score was obtained in the practice item regarding guidance on daily toothbrushing with fluoride toothpaste, in which 95.6% reported performing it; the worst was in the item of knowledge about minimally invasive restorations, with 38.6%. Regarding the barriers, the item of adequate remuneration for minimal intervention procedures had a higher percentage of professionals who evaluated it unfavorably (38.3%). This study showed an overall proportion of knowledge, ability, and attitudes of dentists adequate, with the exception of attitudes in selective removal of decayed dentin. It is concluded that, in general, CDs from the DF have adequate skills in OMI, but there are difficulties to be faced in the practice of minimal intervention.

**Keywords:** Dental caries; Evidence-based dentistry; Health knowledge; Aptitude; Attitude of Health Personnel; Minimum Intervention Dentistry.

**LISTA DE ABREVIATURAS**

RS – Revisão Sistemática

DF – Distrito Federal

CDs – Cirurgiões-Dentistas

OMI – Odontologia de Mínima Intervenção

UnB – Universidade de Brasília

CRO-DF - Conselho Regional de Odontologia do Distrito Federal

CEP/FS - Comitê de Ética em Pesquisa da Faculdade de Ciências da Saúde

TCLE - Termo de Consentimento Livre e Esclarecido

**SUMÁRIO**

[1 INTRODUÇÃO 11](#_Toc141634265)

[1.2. JUSTIFICATIVA E RELEVÂNCIA DO ESTUDO 13](#_Toc141634266)

[1.3. OBJETIVOS 14](#_Toc141634267)

[**1.3.1 Objetivo Geral** 14](#_Toc141634268)

[**1.3.2 Objetivos Específicos** 14](#_Toc141634269)

[1.4 HIPÓTESE (S) 14](#_Toc141634270)

[2. KNOWLEDGE, ATTITUDES, AND PRACTICE OF DENTISTS ON MINIMAL INTERVENTION DENTISTRY: A SYSTEMATIC REVIEW AND META-ANALYSIS. 18](#_Toc141634271)

[2.1 INTRODUCTION 18](#_Toc141634272)

[2.2 METHODS 19](#_Toc141634273)

[**2.2.1 Protocol and Registration** 19](#_Toc141634274)

[**2.2.2 Eligibility Criteria** 19](#_Toc141634275)

[**2.2.3 Information Sources, Search Strategy, and Selection Process** 20](#_Toc141634276)

[**2.2.4 Data Items and Effect Measures** 20](#_Toc141634277)

[**2.2.5 Methodological Quality** 21](#_Toc141634278)

[**2.2.6 Synthesis Of Results** 21](#_Toc141634279)

[2. 3 RESULTS 23](#_Toc141634280)

[**2.3.1 Study Selection and Characteristics** 23](#_Toc141634281)

[**2.3.2 Methodological Quality** 34](#_Toc141634282)

[**2.3.3 Data Analysis** 34](#_Toc141634283)

[2.4 DISCUSSION 38](#_Toc141634284)

[2.5 CONCLUSION 42](#_Toc141634285)

[2.6 DECLARATION OF COMPETING INTEREST 42](#_Toc141634286)

[2.7 ACKNOWLEDGMENTS 42](#_Toc141634287)

[**REFERENCES** 43](#_Toc141634288)

[3. CONSTRUÇÃO, ADAPTAÇÃO E PROPRIEDADES PSICOMÉTRICAS: um estudo de CONHECIMENTOS, HABILIDADES E ATITUDES DOS CIRURGIÕES-DENTISTAS SOBRE A ODONTOLOGIA DE MÍNIMA INTERVENÇÃO. 48](#_Toc141634289)

[3.1 INTRODUÇÃO 48](#_Toc141634290)

[3.2 METODOLOGIA 50](#_Toc141634291)

[**3.2.1 População, Contexto e Período de coleta** 50](#_Toc141634292)

[**3.2.2 Construção e Adaptação do questionário** 51](#_Toc141634293)

[**3.2.3 Avaliação de Evidências Psicométricas** 55](#_Toc141634294)

[**3.2.4 Medidas** 56](#_Toc141634295)

[**3.2.5 Plano de análise de dados** 57](#_Toc141634296)

[3.3 RESULTADOS 57](#_Toc141634297)

[3.4 DISCUSSÃO 60](#_Toc141634298)

[3.5 CONCLUSÃO 62](#_Toc141634299)

[4. Conhecimentos, habilidades, atitudes E BARREIRAS DOS CIRURGIÕES DENTISTAS do distrito federal SOBRE A ODONTOLOGIA DE MÍNIMA INTERVENÇÃO: ESTUDO TRANSVERSAL 66](#_Toc141634300)

[4.1 INTRODUÇÃO 66](#_Toc141634301)

[4.2. METODOLOGIA 68](#_Toc141634302)

[**4.2.1 Caracterização da pesquisa** 68](#_Toc141634303)

[**4.2.2 População e Seleção da amostra** 68](#_Toc141634304)

[**4.2.3 Instrumento e Coleta dos dados** 68](#_Toc141634305)

[**4.2.4 Metodologia de análise e interpretação dos dados** 70](#_Toc141634306)

[4.3 RESULTADOS 71](#_Toc141634307)

[**4.3.1 Participantes e Características** 72](#_Toc141634308)

[4.4 DISCUSSÃO 85](#_Toc141634309)

[**REFERÊNCIAS** 89](#_Toc141634310)

[5. DISCUSSÃO GERAL E CONSIDERAÇÕES FINAIS 92](#_Toc141634311)

[5.1 DISCUSSÃO GERAL 92](#_Toc141634312)

[5.2 CONSIDERAÇÕES FINAIS 94](#_Toc141634313)

[APÊNDICE 100](#_Toc141634314)

[**APÊNDICE 1** – Artigo 1 publicado 100](#_Toc141634315)

[**APÊNDICE 2** – Quadro da estratégia de busca das bases de dados 110](#_Toc141634316)

[**APÊNDICE 3** – Quadro dos artigos excluídos e razão para exclusão 112](#_Toc141634317)

[**APÊNDICE 4** – Tabela de desfechos dos estudos incluídos 114](#_Toc141634318)

[**APÊNDICE 5** – Tabela de Metanálise de proporção de conhecimentos em Odontologia de Mínima Intervenção 129](#_Toc141634319)

[**APÊNDICE 6** – Tabela de Metanálise de proporção de atitudes e prática em Odontologia de Mínima Intervenção 131](#_Toc141634320)

[**APÊNDICE 7 –** Questionário de pesquisa – estudo transversal 133](#_Toc141634321)

[**APÊNDICE 8** – Quadro com termos de busca de experts no assunto competências em Odontologia de Mínima Intervenção para base SCOPUS 150](#_Toc141634322)

[**APÊNDICE 9** – Checklist STROBE para o estudo “Conhecimentos, habilidades, atitudes e barreiras dos cirurgiões-dentistas do Distrito Federal sobre a Odontologia de Mínima Intervenção: estudo transversal” 151](#_Toc141634323)

[**APÊNDICE 10 –** Quadro com questões de conhecimentos, habilidades, atitudes e barreiras sobre OMI 153](#_Toc141634324)

[**APÊNDICE 11 –** Descritivo da análise estatística completa 159](#_Toc141634325)

[ANEXO 175](#_Toc141634336)

[**ANEXO 1 –** Parecer Consubstanciado do Comitê De Ética em Pesquisa (CEP/FS/UnB) 175](#_Toc141634337)

# 1 INTRODUÇÃO

As doenças bucais afetam mais de 3,5 bilhões de pessoas em todo o mundo, sendo a cárie dentária não tratada a condição mais prevalente, segundo o estudo de Carga Global de Doenças (Global Burden of Disease - GBD).(1) Em 2017, ela afetava 2,3 bilhões de dentes permanentes e 532 milhões em dentes decíduos, com crescente aumento em países de baixa e média renda.(2) Já nos países de renda média-alta, como é o caso do Brasil, há uma tendência a diminuição dessa prevalência.(3) A prevalência brasileira de cárie não-tratada em dentes permanentes em 2017 foi de 37,46%. Porém, mesmo havendo redução em comparação ao estudo de 1990, que era de 38.17%, a prevalência atual ainda é considerada alta.(3)

A cárie dentária é entendida como uma doença crônica não-transmissível (DCNT), e se desenvolve devido a uma combinação de fatores genéticos, psicológicos, ambientais e comportamentais.(4, 5) Ela é uma consequência da alteração na microbiota oral normal, devido a uma mudança no estilo de vida e ambiente oral.(5) Com um melhor entendimento da etiopatogenia da doença, dependente de biofilme mas mediada por comportamento, os dentistas tem uma maior amplitude de opções de tratamento com evidências disponíveis para isso.(6)

O processo mecânico, tradicional, de preparo e restauração de cavidades não cura a doença cárie.(7) O reparo dos danos teciduais causados pelo processo de cárie não deve ser o foco do cuidado,(8) o controle da doença deve ser realizado por meio de ações preventivas de longo prazo, com o comprometimento e mudança de comportamento do paciente, assistido pelos profissionais.(7) Há uma mudança em direção a alternativas de tratamento individualizadas, com intervenções mínimas, baseadas em evidência científica e focada na promoção e manutenção da saúde bucal.(6)

A “Odontologia de Mínima Intervenção” (OMI) concretizou-se como uma resposta a Odontologia Operatória baseada em conceitos de mais de um século atrás.(9) Ela se ampara no respeito sistemático aos tecidos dentários naturais(10) e busca a manutenção dos dentes funcionais por toda a vida(9). Para alcançar o objetivo de manter os dentes funcionais por toda a vida devem ser tomadas medidas para manter os dentes livres de lesões de cárie.(9) Entre essas medidas estão a detecção precoce e avaliação de risco à cárie; remineralização de esmalte e dentina desmineralizados; medidas preventivas ótimas; intervenções operatórias minimamente invasivas e reparar em lugar de trocar restaurações.(9, 10). Há também o conceito do cuidado oral em “Mínima Intervenção”, que é a abordagem integral de cuidado em equipe, para que se mantenha a saúde bucal do indivíduo a longo prazo com plano de cuidado preventivo, concentrado no paciente e baseado no comportamento, respeitando-se suas necessidades, desejos e expectativas.(8)

A evidência científica crescente e consistente apoia o tratamento de cárie dentária com estratégias menos invasivas.(11) Há uma forte base de evidência científica para apoiar as técnicas da OMI e há outras em fase de teste.(6, 12) Cabe ressaltar ainda que a Federação Dentária Internacional (FDI) apoia a OMI como o modo contemporâneo de tratar a cárie dentária.(13) Apesar da crescente evidência em torno da mínima intervenção ainda persiste o desafio em traduzir esses conceitos para a prática clínica.(14) O modo como os profissionais tem lidado com a cárie dentária tem se tornado o tema central para reduzir sua carga global.(12)

Os dentistas continuam a ser treinados para intervir de forma reativa e cirúrgica, em lugar de agir de forma proativa e preventiva.(15) Sem repensar a educação odontológica em linha com a mudança de paradigma para o uso rotineiro de estratégias menos invasivas no manejo das lesões de cárie, é improvável que esse novo paradigma seja implementado na prática.(15) Um revisão sistemática encontrou que uma grande proporção de profissionais ainda usa a abordagem cirúrgica para lesões em esmalte e lesões proximais na junção cemento-esmalte.(16) Um estudo prévio que avaliou a tendência mundial de se utilizar estratégias menos invasivas em dentes decíduos encontrou que os dentistas tendem a intervir de forma operatória em estágios iniciais da lesão de cárie, embora existam variações entre os países.(17) Os resultados ao longo dos anos sugere que há uma lacuna entre a visão científica e a prática clínica no tratamento da doença cárie.(17)

As explicações para a lacuna entre a pesquisa científica e a prática clínica da OMI são complexas.(7, 18) A pesquisa translacional tem mostrado que a maioria das barreiras para adotar evidência científica em prática clínica dá-se em três áreas, resumidas no desconhecimento, dificuldade em executar ou mesmo a dificuldade em mudar as práticas.(7) Algumas causas encontradas no caso da OMI são as inconsistências nos guias de prática clínica, diferenças na educação odontológica, políticas nacionais de saúde, sistemas de remuneração(7), além de fatores humanos.(18) Também são citados outros fatores para essa variação, como o acesso à internet e literatura odontológica impressa, além da disponibilidade de equipamentos e materiais odontológicos.(18)

Há a necessidade de uma nova agenda de saúde bucal para incentivar pesquisas que busquem entender as barreiras e facilitadores para incorporação sistemática de protocolos de OMI.(15) Entre algumas barreiras citadas estão problemas com regulação, conhecimento clínico e treinamento dos profissionais.(15) Recomenda-se a realização de ensaios clínicos para confirmar a efetividade e custo-efetividade das intervenções, além de enfrentar os desafios para se implementar intervenções efetivas no mundo real. (15)

1.2. JUSTIFICATIVA E RELEVÂNCIA DO ESTUDO

Com base nos achados apresentados, verifica-se que há a necessidade de realizar um estudo amplo que busque o melhor entendimento das competências e fatores dificultadores dos dentistas acerca da OMI. Justifica-se a necessidade de revisar a literatura científica atual para entender as competências e barreiras dos cirurgiões-dentistas em nível mundial. A partir do panorama mundial sobre o tema, deve ser realizado um estudo transversal para entender como é a realidade de competências e barreiras dos cirurgiões-dentistas no atual contexto regional brasileiro.

Com esse estudo será possível identificar quais áreas temáticas os profissionais possuem competências, em quais possuem dificuldades, além de possíveis fatores que limitam o desenvolvimento das competências. O manejo da cárie dentária com a mínima intervenção tem como maior beneficiado os pacientes assistidos, sendo de suma importância a prática baseada na melhor evidência disponível. Com o conhecimento gerado a partir desse estudo, visa-se a sensibilização dos cirurgiões-dentistas sobre a importância da compreensão e prática da OMI. O panorama encontrado nesse estudo possibilitará que cursos de odontologia, tanto a nível de graduação e pós-graduação, utilizem o conhecimento gerado para analisarem o ensino da mínima intervenção e as necessidades de ajustes nos seus currículos. Além disso, gestores e profissionais que lidam com a organização do cuidado em saúde bucal podem utilizar-se do conhecimento gerado para programar capacitações e reorientação de seus serviços.

## 1.3. OBJETIVOS

### **1.3.1 Objetivo Geral**

O objetivo do presente é avaliar as competências e barreiras dos cirurgiões-dentistas acerca da Odontologia de Mínima Intervenção.

### **1.3.2 Objetivos Específicos**

A dissertação foi dividida em três etapas, resultando em três artigos independentes que seguem os seguintes objetivos: (1) Realizar uma revisão sistemática para avaliar os conhecimentos, atitudes e práticas dos cirurgiões-dentistas sobre a Odontologia de Mínima Intervenção; (2) Elaborar e avaliar as propriedades psicométricas de um questionário para mensurar os conhecimentos, habilidades e atitudes dos cirurgiões-dentistas acerca da Odontologia de Mínima Intervenção (OMI); (3) Analisar os conhecimentos, habilidades e atitudes e barreiras autorreferidas pelos cirurgiões-dentistas acerca dos princípios da Odontologia de Mínima Intervenção; verificar a associação do perfil, formação e experiência profissional dos cirurgiões-dentistas com as competências e barreiras apresentadas.

## 1.4 HIPÓTESE (S)

Espera-se que o presente estudo possa descrever as competências dos cirurgiões-dentistas sobre o conhecimento e prática da Odontologia de Mínima Intervenção e investigar eventuais falhas e possíveis fatores explicativos para as falhas apresentadas. A hipótese será considerada nula caso os cirurgiões-dentistas não apresentem falhas nas competências investigadas ou não apresentem barreiras que dificultam a prática da Odontologia de Mínima Intervenção.

H0: Os cirurgiões-dentistas não possuem fatores limitantes na teoria e prática da Odontologia de Mínima Intervenção para o manejo de cárie dentária.

H1: Os cirurgiões-dentistas possuem fatores limitantes na teoria e prática da Odontologia de Mínima Intervenção para o manejo de cárie dentária.

**REFERÊNCIAS**

1. Peres MA, Macpherson LMD, Weyant RJ, Daly B, Venturelli R, Mathur MR, et al. Oral diseases: a global public health challenge. Lancet (London, England). 2019;394(10194):249-60.

2. Bernabe E, Marcenes W, Hernandez C, Bailey J, Abreu L, Alipour V, et al. Global, Regional, and National Levels and Trends in Burden of Oral Conditions from 1990 to 2017: A Systematic Analysis for the Global Burden of Disease 2017 Study. Journal of dental research. 2020;99(4):362-73.

3. Crescente LG, Gehrke GH, Santos CM. Mudanças da prevalência de dentes permanentes cariados no Brasil e em países de renda média-alta nos anos 1990 e 2017. Ciência & Saúde Coletiva. 2023;27:1181-90.

4. WHO. Noncommunicable diseases: World Health Organization (WHO); 2022 [Available from: <https://www.who.int/news-room/fact-sheets/detail/noncommunicable-diseases>.

5. Pitts NB, Zero DT, Marsh PD, Ekstrand K, Weintraub JA, Ramos-Gomez F, et al. Dental caries. Nature reviews Disease primers. 2017;3:17030.

6. Innes NPT, Chu CH, Fontana M, Lo ECM, Thomson WM, Uribe S, et al. A century of change towards prevention and minimal intervention in cariology. Journal of dental research. 2019;98(6):611-7.

7. Banerjee A. MI'opia or 20/20 vision? Br Dent J. 2013;214(3):101-5.

8. Banerjee A. 'Minimum intervention' – MI inspiring future oral healthcare? Br Dent J. 2017;223(3):133-5.

9. Frencken JE, Peters MC, Manton DJ, Leal SC, Gordan VV, Eden E. Minimal intervention dentistry for managing dental caries–a review: report of a FDI task group. Int Dent J. 2012;62(5):223-43.

10. Ericson D. The concept of minimally invasive dentistry. Dental update. 2007;34(1):9-10, 2-4, 7-8.

11. Innes NP, Frencken JE, Schwendicke F. Don't Know, Can't Do, Won't Change: Barriers to Moving Knowledge to Action in Managing the Carious Lesion. Journal of dental research. 2016;95(5):485-6.

12. Banerjee A, Frencken JE, Schwendicke F, Innes NPT. Contemporary operative caries management: consensus recommendations on minimally invasive caries removal. Br Dent J. 2017;223(3):215–22.

13. FDI. FDI policy statement on Evidence-based dentistry Adopted by the FDI General Assembly, September 2016, Poznan, Poland. Int Dent J. 2017;67(1):12-3.

14. Elouafkaoui P, Bonetti D, Clarkson J, Stirling D, Young L, Cassie H. Is further intervention required to translate caries prevention and management recommendations into practice? British Dental Journal. 2015;218(1):20-1.

15. Bernabé E, Marcenes W. Can minimal intervention dentistry help in tackling the global burden of untreated dental caries? British dental journal. 2020;229(7):487-91.

16. Innes NPT, Schwendicke F. Restorative Thresholds for Carious Lesions: Systematic Review and Meta-analysis. J Dent Res. 2017;96(5):501-8.

17. Laske M, Opdam NJM, Bronkhorst EM, Braspenning JCC, van der Sanden WJM, Huysmans MCDNJM, et al. Minimally Invasive Intervention for Primary Caries Lesions: Are Dentists Implementing This Concept? Caries research. 2019;53(2):204-16.

18. Grol R, Grimshaw J. From best evidence to best practice: effective implementation of change in patients' care. Lancet (London, England). 2003;362(9391):1225-30.

# 2. KNOWLEDGE, ATTITUDES, AND PRACTICE OF DENTISTS ON MINIMAL INTERVENTION DENTISTRY: A SYSTEMATIC REVIEW AND META-ANALYSIS.^[[1]](#footnote-1)^

## 2.1 INTRODUCTION

Minimal Intervention Dentistry (MID) is evidence-based oral health care that aims to keep teeth functional for life and in a caries-free state.(1, 2) The goals of MID are achieved by following important strategies for optimal disease control.(3) The main strategies included early detection, risk assessment, and care planning; disease control and preventive measures; minimally invasive operative management and repair instead of replacing restorations.(1-3)

The philosophy of MID has been supported by scientific literature around the world in the last 20 years.(4) The cognizance of MID is continuously increasing, along with growing scientific evidence.(5) All collaborators of oral health care must cooperate to execute the MID approach and make it the rule.(5) Besides, FDI World Dental Federation defends MID as the up-to-date way to control dental caries.(6) The FDI Policy Statement on MID for managing dental caries is established on the current best evidence available.(6)

The contemporary understanding of dental caries management supports nonoperative treatment whenever possible.(7) The invasive operative treatment should be limited to situations where oral health balance is missing and a cavity has happened.(3) However, the spotlight on dental restorative care is still driving the approach to dental caries management around the world.(8) Research indicates, for example, that dentists who do not regularly provide Caries Risk Assessment (CRA) or diet counseling are more likely to take an invasive approach.(9) A recent systematic review and meta-analysis indicated that a high proportion of dentists intervene too invasively on caries lesions, so less invasive therapies should be recommended for a more conservative clinical practice.(10)

Considering the global and economic burden of dental caries, it is of great relevance to understanding the way oral healthcare professionals are treating dental caries.(11, 12) As far as we are concerned, there is no preview systematic review analyzing the cognizance and practice of MID between dentists. Thus, the present systematic review aims to determine the knowledge, attitudes, and practice of dentists on the MID approach and its main principles.

## 2.2 METHODS

### **2.2.1 Protocol and Registration**

The present systematic review was reported following the Preferred Reporting Items for Systematic Reviews and Meta-analyses (PRISMA)(13). A protocol was previously registered in the International Prospective Register of Systematic Reviews (PROSPERO) under the code CRD42021257518, in the following link: <https://www.crd.york.ac.uk/prospero/display_record.php?RecordID=257518>

### **2.2.2 Eligibility Criteria**

The research question was formulated following the CoCoPop(14) (Condition, Context, and Population) framework: “Among dentists, what is the proportion of knowledge, attitude, and practice on MID principles?” Where Condition was considered the knowledge, attitude, and practice on MID principles; Context was the proportion or prevalence, and Population was dentists. Studies assessing dentists’ knowledge, attitudes, and practice on MID, and their proportions were evaluated.

Cross-sectional studies that analyzed the knowledge, attitude, and practice of dentists on MID main principles were the inclusion criteria of the present review. The following exclusion criteria were applied: 1) Studies in which participants were undergraduate students; 2) Studies that evaluated only caries treatment decision; 3) Studies that evaluated only caries diagnosis; and 4) Studies with a lack of information about the outcomes, even after contacting the corresponding authors.

### **2.2.3 Information Sources, Search Strategy, and Selection Process**

Individual search strategies were developed for the following databases: Cochrane Library, Dentistry and Oral Sciences Source - DOSS (EBSCO), Embase, Latin American and Caribbean Health Sciences (LILACS), PsycINFO, PubMed, Scopus, and Web of Science. Additionally, a grey literature search was conducted in the databases Google Scholar, ProQuest Dissertations and Theses, and System for information on the grey literature in Europe (OpenGrey). All searches were performed on January 8^th^, 2022. The search strategy was initially developed for PubMed using keywords and MeSH terms and adapted for other databases. All the search strategies can be found in the online supplementary material (Apêndice 2). Reference lists of the eligible studies were checked to identify potentially relevant studies. No publication period or language restrictions were applied. Studies were managed on EndNote (EndNoteTMX9 Clarivate Analytics, USA) and Rayyan reference management software. Duplicate studies were removed on both software.

Four reviewers (RCM, PSS, PMSM, and FCV) conducted the search. In all phases, the reviewers were trained, and a pilot test was conducted until all of them understood the eligibility criteria. In phase one (titles and abstracts reading) and phase two (full-text reading), the studies were assessed for the eligibility criteria by three independent reviewers (RCM, PSS, PMSM). Disagreements were solved in a consensus meeting, and if any disagreement persisted, the fourth reviewer (FCV) was involved to steer the decision. In the data extraction phase, two reviewers (RCM, PSS) independently performed data collection in a self-designed excel worksheet, after piloting four studies.

### **2.2.4 Data Items and Effect Measures**

The following data was extracted from included studies: authorship, year and country of publication, study design, the main purpose of the study, sampling strategy, and characteristics of the sample (sample size, gender, age, professional experience, workplace, post-graduate training, continuing education on MID), details of the applied questionnaire (questionnaire administration, number of questions, validity, and reliability of questionnaire), categorization of skills levels, scores on MID with statements and percentages, associated factors, and conclusions. After data collection, the two reviewers (RM, PSS) settled disagreements in a consensus meeting and consulted the published records of the included studies. When necessary, for absent or incomplete data, the correspondence author was contacted via email. Two attempts were made to contact the corresponding author. In the absence of answers, the information was not included.

### **2.2.5 Methodological Quality**

The Joanna Briggs Institute Critical Appraisal Checklist for Studies Reporting Prevalence Data (15) was applied to assess the methodological quality of the individual studies. This tool comprises nine questions. These questions assess the methodological quality of studies considering sample characteristics, sampling method, sample size, participants’ description, statistical analysis, validity, reliability of condition under study, and response rates. All questions can be answered as “yes”, “no”, “unclear” or “not applicable”. Two reviewers (RM and PSS) were previously trained and calibrated to use this tool, discussing each predetermined question. In case of disagreements, a third reviewer (FCV) was involved to steer the decision. The methodological quality assessment plot was created by using the robvis tool.(16)

### **2.2.6 Synthesis Of Results**

The prevalence of positive knowledge, attitude, and practice was extracted from cross-sectional included studies, transformed from relative frequencies when necessary, and expressed in absolute frequencies. To synthesize the analysis, the following criteria previously reported(16) were adopted: a frequency of up to 25% was considered as ‘insufficient’ skill, between 26% and 50% as ‘reasonable’, between 51 and 75% as ‘good’, and between 76 and 100% as ‘excellent’.

Statements with similar responses and homogeneity in their categorization were pooled. Likert-type response scales were re-scored to dichotomous measurement, as previously described.(17) In the 5-point Likert-type scale, responses 4 and 5 were collapsed into an agreement category, responses 1 and 2 into a disagreement category, and 3 into a neutral category. In the 4-point Likert-type scale, responses 3 and 4 were collapsed into an agreement category and responses 1 and 2 into a disagreement category. Dichotomous responses remained the same. The categories in agreement with the last evidence on MID were considered for the synthesis. The statements described as attitude or as practice on MID were pooled together like was presented in the studies.

A proportion meta-analysis was performed to assess the prevalence of knowledge, attitude, and practice (KAP) on MID. The meta-analysis was performed with the R program, version 3.5.2 with RStudio (R Core Team, Vienna, Austria, 2018 with the meta package(18). The random effect model was applied due to the expected variability among the studies(19). The pooled prevalence estimated of KAP (95% confidence intervals) was presented as a percentage and the variance was stabilized using the Freeman-Tukey double arcsine method.

The Cochran’s Q test (20) was used to assess the heterogeneity and the I² statistic for evaluation of true variation due to heterogeneity (21). The I² is expected to be high in a meta-analysis of proportion, and this can be due to little variance, and true heterogeneity due to differences in the time and place of included studies.(22)

The prediction intervals were graphically presented in each meta-analysis. In meta-analyses with few studies, if the studies are small and if significant heterogeneity is present, the prediction interval tends to be wider than the range of study results.(23)

Publication bias was evaluated using a funnel plot approach, and Begg’s and Egger’s correlation test was performed to identify funnel plot asymmetry. A p-value of 0.05 or less was considered statistically significant in each analysis. If any asymmetry was identified, the included studies were checked, assessing whether the asymmetry is due to publication bias or other reasons, such as the presence of methodological heterogeneity.

## 2. 3 RESULTS

### **2.3.1** Study Selection and Characteristics

The literature search identified 2,470 records from the main databases and 239 from the grey literature. After removing duplicates, 1,848 records remained for the title and abstract reading (phase 1). The full text of 21 records was selected for reading (phase 2) and 13 records from 11 studies(24-36) met the inclusion criteria of the review. One additional study(37) was selected from the reference list of one included study, totaling 12 studies and 14 reports.

The flow diagram **(Figure 1**) shows the results of the search and selection process. The excluded studies are shown in Appendix S2 (Apêndice 3) with reasons for exclusion (online supplementary material).

The studies were published between 2010 and 2021. The sample size of the included studies ranged from 70[26] to 465[25] participants with a higher number of male dentists in the majority of studies. [25, 27, 30, 31, 32, 34, 35, 37]

The studies showed a wide variety of questions and statements about knowledge, attitude, and practice on MID. A great part of outcomes could not be grouped due to the heterogeneity of statements and varied response categories. Ordinal responses, with a 5-point [25, 26, 30, 31, 32, 33, 35, 36, 37, 38] and 4-point Likert-type scale[25, 26, 33, 37] were the most frequent. Dichotomous responses were used often, with the following options: yes or no[29, 31, 35, 37] ; agreement or disagreement(29); aware or not aware(35); consistent or inconsistent with evidence[27]; correct or incorrect(37). Multiple-choice responses were also present in some studies.[29, 32, 38]

The main characteristics of included studies are provided in **Table 1.** All outcomes collected from included studies can be accessed in the supplementary material (Apêndice 4)**.**

The most prevalent topic was minimally invasive treatment on MID followed by noninvasive treatment and diagnosis. The least researched topics on MID were repairment, microinvasive treatment, and general aspects of MID.

**Figure 1 -** PRISMA flow diagram.

**Table 1-** Main characteristics of included studies

| **First Author; Year; Country;**  **Study design** | **Sample Size** | **Sex (Female and male, n/%)** | **Mean age (years ± SD) or Age range (years) or Percentage by age groups** | **Professional experience in years (Mean ± SD) or (Median IQR) or (percentage by age groups)** | **Workplace (n/%)** | **Specialty practice (n/%)** | **Training on MID (n/%)  Source of training MID (n/%)** | **Type of assessment** | **Categorization of Skills Levels** |
| --- | --- | --- | --- | --- | --- | --- | --- | --- | --- |
| Gaskin  (2010); USA Cross-sectional | 465 | Female (88/18.9%)  Male (377/81.1%) | 45.6 y  26- 75 y | 17.2 y | Civilian (327/70.3%)  Federal (138/29.7%) | Postgraduate training (270/58.1%) | NM | KAP | Likert-type scale -5-point to Knowledge.  Likert-type scale -4 point to Attitudes and Practice. |
| Haider  (2021); Pakistan  Cross-sectional | 70 | NM | NM | 0-2 y (19/27.1%) 2-5 y (17/24.3%) 5-10 y (17/24.3%)  >10 y (17/24.3%) | NM | NM | Thoroughly (3/4.3%)  To some extent (37/52.9%) Not at all (30/42.9%)  Source of training  During BDS (51/72.9%) During their internship time (19/27.1%) | KP | Likert-type scale-4-point to Knowledge.  Likert-type scale-5-point to Practice. |
| Kakudate (2020); Japan Cross-sectional  Kakudate  (2021); Japan. Interventional | 206 | Female (44/21.4%)  Male (162/78.6%) | NM | 20 ± 12 | Private (206/100%) | Specialty practice - Operative dentistry (52/25.2%)  Specialty Practice - Non-Operative dentistry (154/74.8%) | NM | P | Response options classified as consistent and  inconsistent with evidence |
| Katz  (2013); Brazil Cross-sectional | 123 | Female (76/61.8%) Male (47/38.2%) | NM | >10 y (74/68.5%) <10 y (34/31.5%) | Public and Private (53/43.1%)  Only Public (47/38.2%) Only Private (23/18.7%) | Yes (74/60.2%)  Specialists in pediatric dentistry, special care dentistry, public health and restorative dentistry (34/27.7%) Specialists in the areas of surgery, endodontics, implants, prosthodontics and legal dentistry (40/32.5%) | Training on MID Yes (14/11.4%) No (109/88.6%) | KP | Yes or No questions. Multiple choice questions. |
| Khan  (2019); Pakistan Cross-sectional | 119 | Female (55/46.2%) Male (64/53.8%) | 21-25 y (35/29.4%) 26-30 y (57/47.9%) 31-35 y (18/15.1%) >35 y (9/7.6%) | < 5 y (56/47.1%) 5-10 y (49/41.2%) >10 y (14/11.8%) | NM | General Dental Practitioners (119/100%) | Training on MID Yes (80/67.2%)  No (39/32.8%)  Source of training  Lectures only (15/18.8%) Clinical Only (3/3.8%) Lectures and clinical both (43/53.8%) Conferences and courses (19/23.8%) | KA | Responses in Agreement (Always/Mostly or Very effective/Effective) and Disagreement (Sometimes/Rarely/Never or Ineffective/Very Ineffective) were calculated together.  Likert-type scale 5-point. |
| Kumar  (2021); India  Cross-sectional | 285 | Female (133/46.7%) Male (152/53.3%) | <30 y (95/33.3%)  35-55 y (99/34.7%) >55 y (91/31,9%) | < 5 y (120/42.1%) 5-10 y (97/34.0%) >10 y (68/23,9%) | NM | Yes (137/48.1%) Community Dentistry (10/7.6%) Pediatric Dentistry (33/25.1%)  Oral Surgery (15/11.4%) Endodontics (26/19.8%) Orthodontics (17/13.0%) Periodontics (12/9.1%)  Oral medicine and radiology (9/6.9%) Prosthodontics (8/6.1%)  Oral pathology (1/0.8%) | Training/Certification/CDE  Yes (131/46.0%)  No (154/54.0%)  Source of Training  Didatic (65/49.6%)  Hands-on (8/6.1%)  Both (42/32.0%)  Others (16/12.2%) | KP | Yes or No questions.  Lkert-type scale 5-point for questions related to the use of various MID techniques. |
| Mirsiaghi  (2018); UK Cross-sectional | 149 | Female (60/40.3%) Male (89/59.7% | 27 y 24-41 y | 1.39 y (0.9 -14.6) | Exclusively NHS (35/23.5%) Mostly NHS (76/51.0%); Mixed 50/50 (17/11.4%); Mostly private (11/7.4%); Exclusively private (10/6.7%). | General Dental Practitioners -(149/100%). | Postgraduate Training in MID (19/12.7% ) | KP | Multiple choice questions.  Likert-type scale 5-point  for Knowledge  Likert-type scale 4-point for Knowledge on studied scenario. |
| Natarajan  (2019); India  Cross-sectional | 119 | NM | <30 y (14/11.8%) 30–40 y (62/52.1%) 40–50 y (41/34.4%)  >50 y (2/1.7%) | 0–2 y (9/7.6%)  2–5 y (31/26.0%) 5–10 y (50/42.0%)  >10 (29/24.4%) | NM | NM | Training on MID Thoroughly (16/13.4%)  To a certain extent (67/56.3%)  Not at all (24/20.1%) | KAP | Likert-type scale - 4-point to Knowledge and Attitudes Likert-type scale- 5 point to Practice. |
| Oliveira. (2011); USA  Cross-sectional  Oliveira  (2016); USA  Cross-sectional. | 136 | Female (64/47.1%) Male (72/52.9%) | 46.8 y  27–74 y | NM | Federal Service dentists (88/64.7%) | Yes (61/46.2%) Dental Public Health or General Public Health (22/16.5%) GPR training (15/11.4%)  AEGD (10/7.4% )  Pediatric dentistry (9/6.6%)  Oral surgery (2/1.5%) Endodontic (1/0.7%)  Orthodontic (1/0.7%)  Periodontics (1/0.7% ) | Training on MID Yes (63/46.6%) No (72/53.3%) Source of training Dental School (23/24.2%)  Continuing dental education courses (8/8.4%)  Continuing dental education courses (53/55.8%) Others (11/11.6%) | K | Yes or No questions  Likert-type scale- 5 point |
| Rayapudi  (2018); India  Cross-sectional | 126 | Female (65/51.9%) Male (61/48.1%) | < 30 y (62/49.6%) 30‑45 y (54/42.7%) 46‑60 y (10/7.6%) | 0‑2 y (42/33.6%) 2‑5 y (32/25.2%) 5‑10 y (21/16.8%) >10 y (31/24.4%) | NM | NM | Training on MID Thoroughly (11/8.7%)  To a certain extent (95/75.4%)  Not at all (20/15.9%)  Source of training  During BDS (46/43.5%)  During Internship (12/11.5%)  During PG (17/16.0%)  Continuing dental education courses (18/16.8%)  Others (13/12.2%) | KA | Dichotomous questions - Aware or Not aware  Likert-type scale - 5 -point |
| Shah  (2016); Saudi Arabia  Cross-sectional | 161 | Female (74/46.0%)  Male (87/54.0%) | 20-30 y (67/41.6%)  31-40 y (79/49.1%)  41-50 (11/6.8%)  >50 y(4/2.5%) | <5 y (35/21.7%) 5-10 y (81/50.3%) 11-15 y (18/11.2%)  >15 y (27/16.8%) | NM | General Dental Practitioners (161/100%) | Training on MID Yes (66/41.0%)  No (95/59.0%)  Source of training Lectures only (20/30.3%)  Clinical only (16/24.2%)  Both (26/39.4%)  Conferences/Courses (4/6.0%) | KA | Yes or No questions  Likert-type scale 5 -point |
| Suma  (2017); India  Cross-sectional | 100 | NM | NM | NM | NM | NM | NM | KAP | Multiple choice questions for Knowledge. Dichotomous questions - correct or incorrect for clinical decision behaviors.  Likert-type scale 5-point. |

SD, Standard Deviation; IQR, Interquartile Range; MID, Minimal Intervention Dentistry; NM, Not mentioned; KAP, Knowledge, Attitudes and Practice; KP, Knowledge and Practice; P, Practice; KA, Knowledge and Attitudes; K, Knowledge.

### **2.3.2 Methodological Quality**

The methodological quality assessment of primary studies is presented in **Figure 2.** None of the included studies reached a positive score in all domains from the JBI critical appraisal tool. Moreover, regarding the domain of sample size adequacy, three studies rated a positive judgment (29, 32, 35) as they reported some type of sample size estimation, eight rated negative, and one uncertain judgment. All the studies had an adequate response rate (more than 70% of the selected sample). Nine studies presented a negative judgment in the domain related to the sampling method due to convenience samples and only three studies reported random sampling and rated positively.[25, 33, 38] The domain of instrument validation rated also unfavorable judgment, with nine uncertain [25, 27, 29, 30, 32, 33, 35-38] and two negative(25) due to the lack of utilization of validated questionnaires. Only the domain about the reliability of the condition under study rated positively in all studies, because the same closed-ended questionary was applied to all participants.

### **2.3.3 Data Analysis**

A total of ten studies and 24 statements were included in the meta-analyses as they were repeated in two or more studies and showed similarity in question stems. Results of the random-effect model meta-analysis, namely the proportion of knowledge, attitude, and practice on MID are shown in **Figure 3** and **Figure 4. Table S2** and **Table S3** of the MA can be seen in the supplementary material (Apêndice 5 e 6). The pooled proportion of Knowledge on Minimal Intervention Dentistry was 75.66% (95% CI: 69.33 – 81.48; p<0.01; I²: 97%; Tau^2^: 0.0456) and of attitudes and practice were 47.95% (95% CI: 38.55 – 57.43; p<0.01; I²: 98%; Tau^2^: 0.0743).

The pooled proportion of knowledge, attitudes, and practice was 63.13% (95% CI: 56.55 – 69.49; p<0.01; I²: 98%; Tau^2^: 0.0790). The most positive statements results were about knowledge, seven of them were rated as “excellent”. Three statements about practice and attitude were rated as excellent. Four statements about knowledge and two about attitude and practice were rated as “good”. One statement about knowledge was rated as “reasonable”. The seven other statements about attitude and practice were also rated as “reasonable” or “insufficient”.

Visual inspection of the funnel plot reveals asymmetry and Begg’s and Egger’s correlation test provided evidence of suspected publication bias (p<0.001) for one statement of attitude and practice, “Remineralization with Casein Phosphopeptides-Amorphous (CPP-ACP)”.

**Figure 2-** Methodological quality assessment based on the Joanna Briggs Institute critical appraisal checklist for studies reporting prevalence data. Questions, Q1 = Was the sample frame appropriate to address the target population?; Q2 = Were study participants recruited in an appropriate way?; Q3 = Was the sample size adequate?; Q4 = Were the study subjects and setting described in detail?; Q5 = Was data analysis conducted with sufficient coverage of the identified sample?; Q6 = Were valid methods used for the identification of the condition?; Q7 = Was the condition measured in a standard, reliable way for all participants?; Q8 = Was there appropriate statistical analysis?; Q9 = Was the response rate adequate, and if not, was the low response rate managed appropriately?.

**Fig. 3 -** Meta-analysis of proportion of knowledge on Minimal Intervention Dentistry :


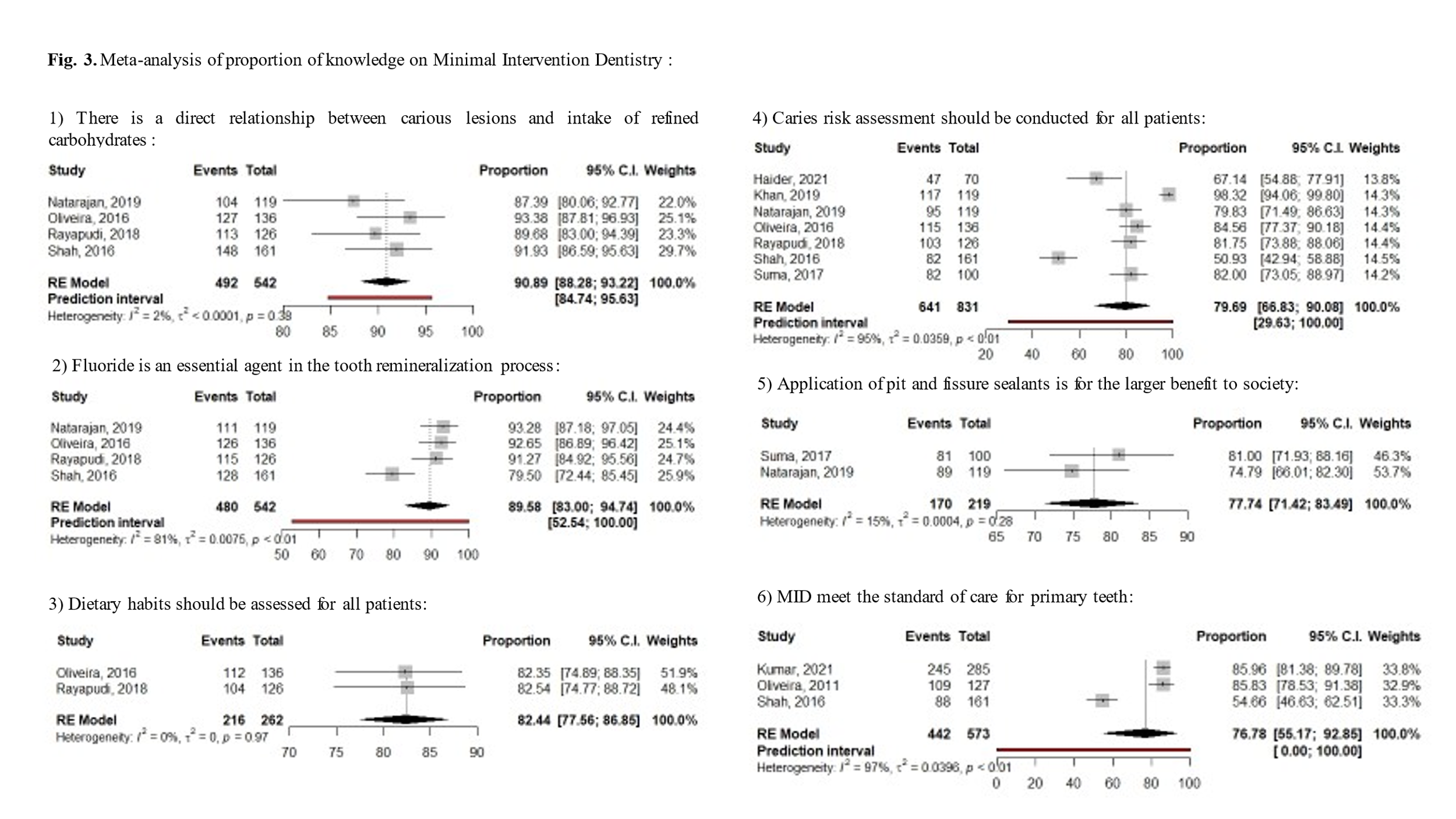


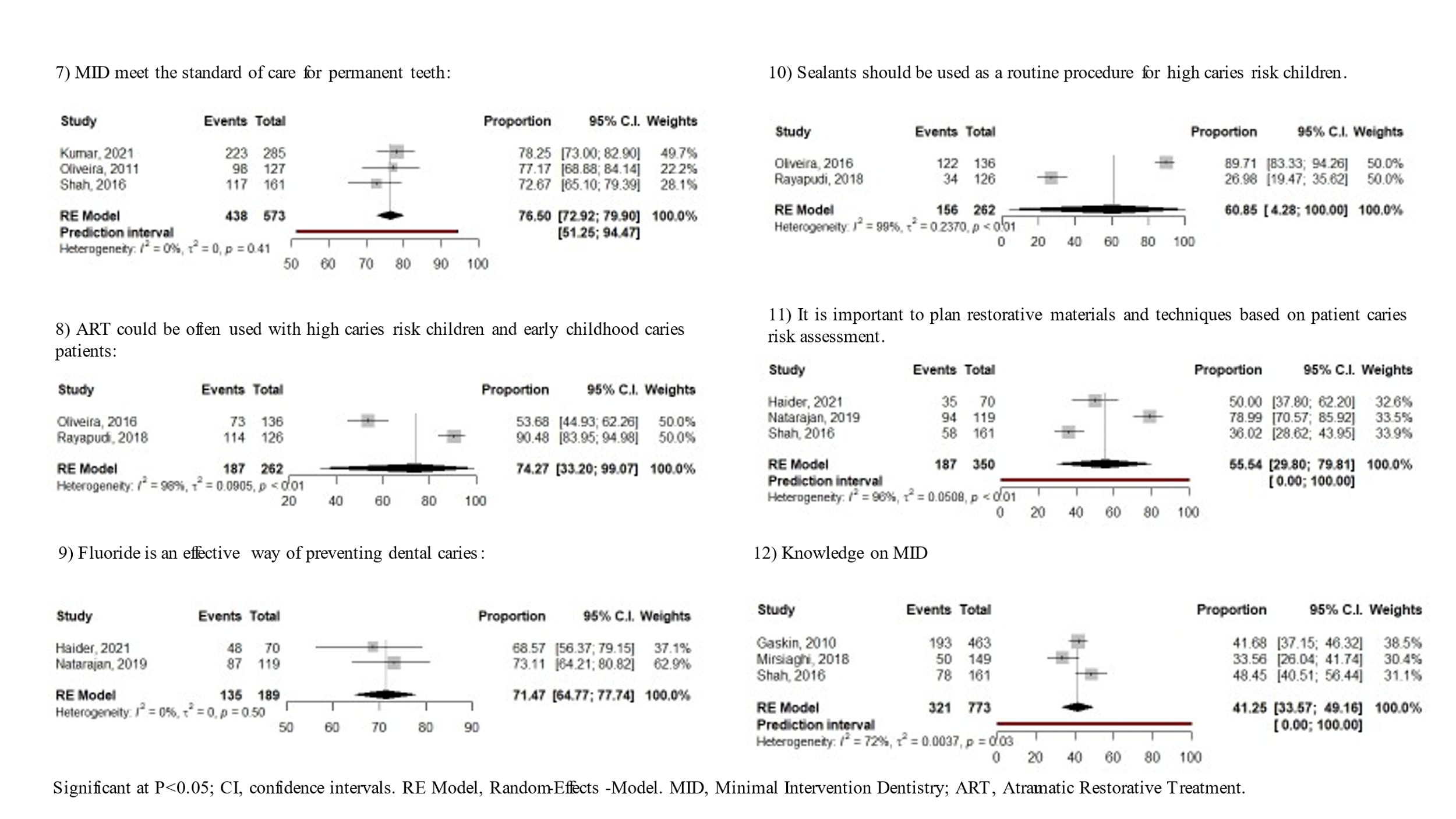


## 2.4 DISCUSSION

This systematic review showed that dentists had overall positive skills on MID topics, and minimally invasive treatment on MID was the topic most studied. Most of the higher rate was about knowledge and the results of the meta-analyses showed that knowledge of MID was rated as excellent in most of the statements. Attitude and practice were rated as reasonable in most of them.

The best prevalence in the meta-analyses, rating excellent and good, was about the detection and prevention/control of MID. It was the case of knowledge on aspects of dental caries etiology and diagnosis, the indication of caries risk assessment, fluoride effectiveness for caries prevention, use of fluoride for tooth remineralization, and pit and fissure sealant benefits. Also, attitudes and practice of topical fluorides’ effectiveness, and diagnosis by using radiographs. The literature shows that risk-based prevention is one of the cornerstones of contemporary caries management and a crucial element of minimal intervention (38). The best available evidence is recommended to support this risk-based, patient-centered decision-making for effective prevention, control, and management of dental caries (39).

Most of the lower prevalence was about minimally invasive restoration, rating reasonable or insufficient in the meta-analyses. It involves attitudes and practice of preventive resin restoration, slot and tunnel preparations, ART, and repairing defective restorations instead of replacement. This lack of practice can be understood. For preventive resin restoration, and slot and tunnel preparations, there are few studies evaluating their effectiveness (3). ART and repair of direct restorations do not appear to differ from conventional restorations in permanent teeth, but the evidence is not high (40, 41). There is a need for more clinical research on these techniques for better guidance in the practice.

MID is not a new approach as its beginning dates to the nineties (42). Since then, there is growing evidence about less invasive and more contemporaneous management of carious lesions (43). This could explain also the excellent rate of knowledge about MID topics in the present study. However, some concerns with attitudes and practice with MID were found, as previously presented.

**Figure 4 -** Meta-analysis of proportion of attitudes and practice on Minimal Intervention Dentistry (MID).


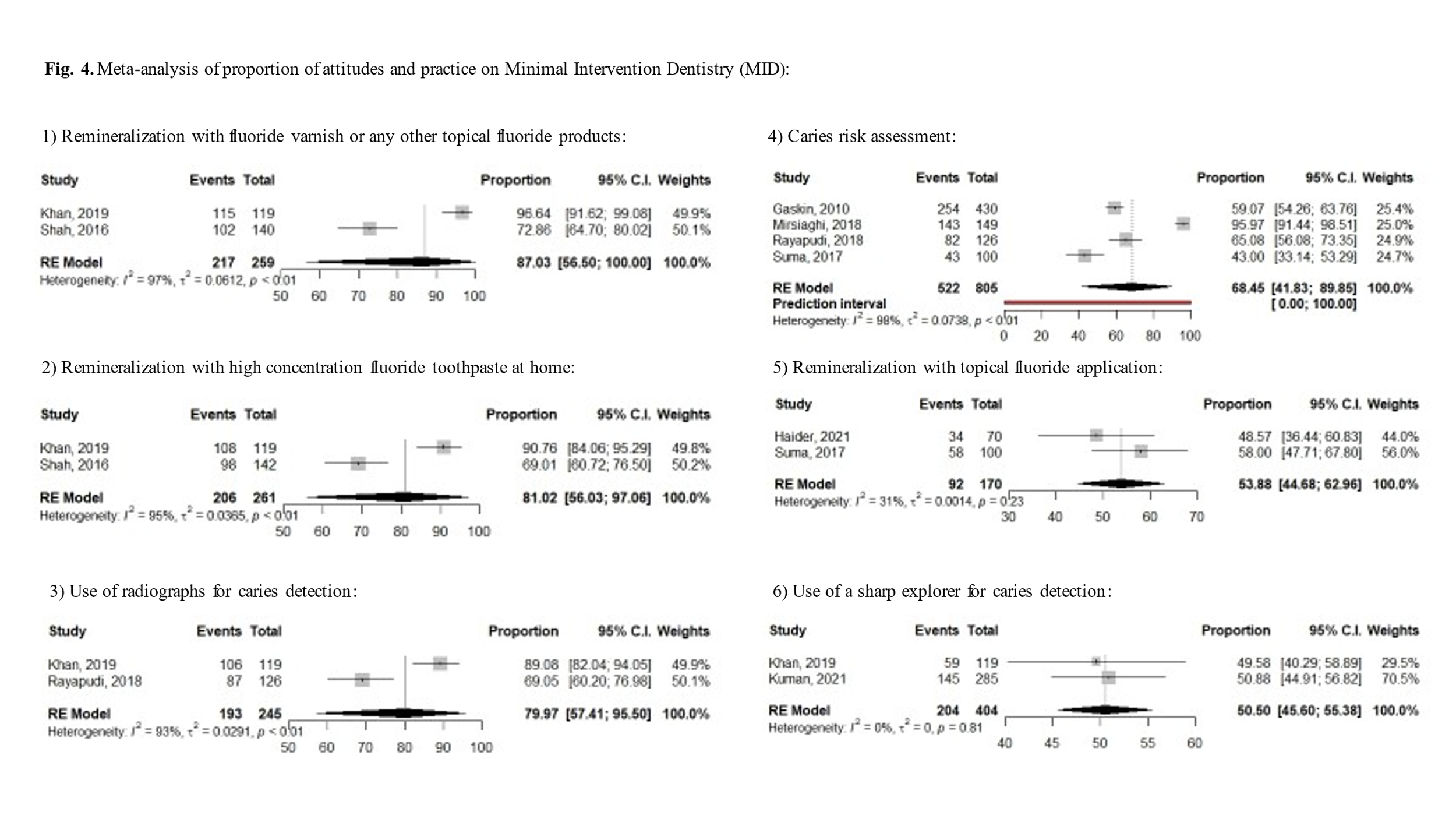


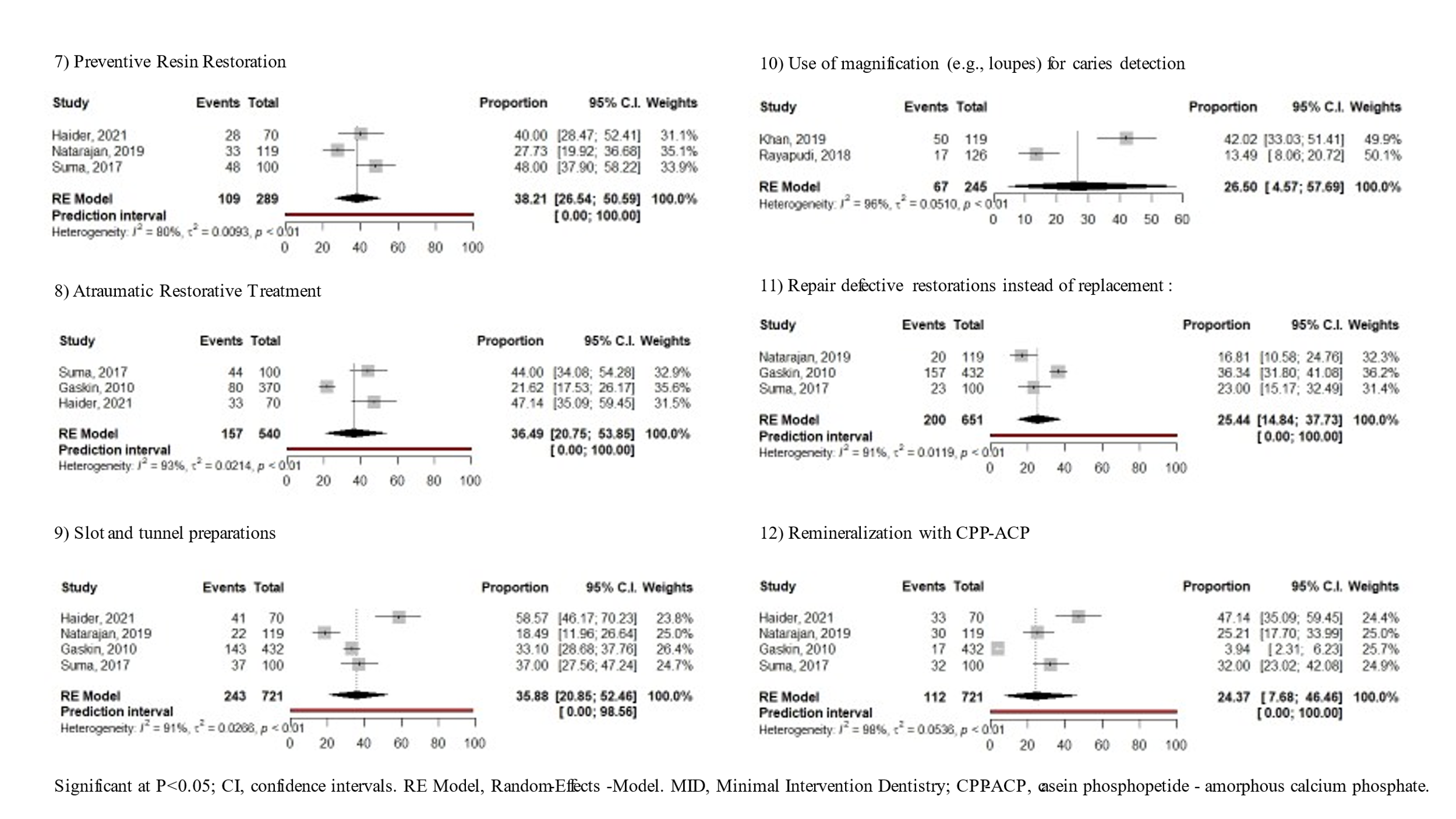


Making evidence is only the first part of the process and the next challenge is implementing that evidence in clinical practice (43). Even if the new evidence is strong, it cannot be assumed that clinical practice will change significantly right away (44). It can take time and there may be gaps when novel, evidence-based treatments are introduced from scientific literature into general clinical practice (44). Nevertheless, to expand the MID philosophy in dental practice, investments in dental education are necessary (45). It is mandatory to incorporate new concepts of dental caries and its management into dentistry schools’ curricula worldwide (45).

From visual inspection of funnel plots, the outliers were from two studies with bigger and smaller sample sizes, compared to other studies included in the plots. It seems to be a trend to lower prevalence in bigger sample size studies and bigger prevalence in the smaller sample size. Although reporting bias may not be discarded, the asymmetry is possibly related to the heterogeneous sample size between studies and the absence of reporting of sampling techniques in most of the studies.

This systematic review had limitations from the primary studies, as follows: (1) there was a lack of information about sampling techniques and the use of convenience samples; (2) the lack of information about questionnaire validation may put the outcomes in suspicion; (3) the high variability in questionnaire characteristics, as the statements about MID topics varied a lot between studies; (4) it was difficult to interpret knowledge, attitudes, and practice scores in primary studies. Many studies did not always provide information about the meaning of the outcomes based on recent scientific evidence and this systematic review spared no efforts to find and compare each outcome with the best and newer evidence.

Although every attempt has been made to address issues with primary studies, the results of the present study should be interpreted with caution. Future main studies in this field must address these limitations, with standard, validated, evidence-based questionnaires, using appropriate sampling methods. There is a need for further investigation on this topic in more countries to better understand the knowledge, attitudes, and practice of MID among dentists worldwide.

## 2.5 CONCLUSION

The pooled prevalence of Knowledge on Minimal Intervention Dentistry was 75.66% and of Attitudes and Practice was 47.95%. The findings suggest that the knowledge of dentists on Minimal Intervention Dentistry topics is adequate, and the attitudes and practices are average. Understanding the cognizance and the way oral healthcare professionals are treating dental caries is the first step to expanding the minimal intervention evidence into a dental practice. Efforts are required to include the new concept of dental caries management in dentistry schools’ curricula. The final goal is to change the practice and make MID the standard of care worldwide. As the studies lacked uniformity in methods, there is still a need for more studies with dentists on this topic worldwide to better understand the knowledge, attitudes, and practice about Minimal Intervention Dentistry.

## 2.6 DECLARATION OF COMPETING INTEREST

The authors declare no conflict of interest.

## 2.7 ACKNOWLEDGMENTS

The authors wish to thank all the corresponding authors of the studies who assisted us with our clarifications on queries regarding their studies.

## **REFERENCES**

1. Tyas MJ, Anusavice KJ, Frencken JE, Mount GJ. Minimal intervention dentistry—a review* FDI Commission Project 1–97. International dental journal. 2000;50(1):1-12.

2. Dawett B, Atkins B, Banerjee A. A guide to building 'MI' oral healthcare practice. Br Dent J. 2017;223(3):223-7.

3. Frencken JE, Peters MC, Manton DJ, Leal SC, Gordan VV, Eden E. Minimal intervention dentistry for managing dental caries - a review: report of a FDI task group. Int Dent J. 2012;62(5):223-43.

4. Walsh LJ, Brostek AM. Minimum intervention dentistry principles and objectives. Aust Dent J. 2013;58:3-16.

5. Banerjee A. 'Minimum intervention' – MI inspiring future oral healthcare? Br Dent J. 2017;223(3):133-5.

6. World Dental Federation FDI. FDI policy statement on Minimal Intervention Dentistry (MID) for managing dental caries: Adopted by the General Assembly: September 2016, Poznan, Poland. Int Dent J. 2017;67(1):6-7.

7. Machiulskiene V, Carvalho JC. Clinical Diagnosis of Dental Caries in the 21st Century: Introductory Paper - ORCA Saturday Afternoon Symposium, 2016. Caries Res. 2018;52(5):387-91.

8. Ismail AI, Tellez M, Pitts NB, Ekstrand KR, Ricketts D, Longbottom C, et al. Caries management pathways preserve dental tissues and promote oral health. Community Dent Oral Epidemiol. 2013;41(1):e12-40.

9. Kakudate N, Sumida F, Matsumoto Y, Manabe K, Yokoyama Y, Gilbert GH, et al. Restorative treatment thresholds for proximal caries in dental PBRN. J Dent Res. 2012;91(12):1202–8.

10. Innes NPT, Schwendicke F. Restorative Thresholds for Carious Lesions: Systematic Review and Meta-analysis. J Dent Res. 2017;96(5):501-8.

11. Banerjee A, Frencken JE, Schwendicke F, Innes NPT. Contemporary operative caries management: consensus recommendations on minimally invasive caries removal. Br Dent J. 2017;223(3):215–22.

12. Kassebaum NJ, Bernabé E, Dahiya M, Bhandari B, Murray CJ, Marcenes W. Global burden of untreated caries: a systematic review and metaregression. J Dent Res. 2015;94(5):650-8.

13. Page MJ, Moher D, Bossuyt PM, Boutron I, Hoffmann TC, Mulrow CD, et al. PRISMA 2020 explanation and elaboration: updated guidance and exemplars for reporting systematic reviews. BMJ (Clinical research ed). 2021;372:160.

14. Munn Z, Stern C, Aromataris E, Lockwood C, Jordan Z. What kind of systematic review should I conduct? A proposed typology and guidance for systematic reviewers in the medical and health sciences. BMC Med Res Methodol. 2018;18(1):5.

15. Munn Z, Moola S, Lisy K, Riitano D, Tufanaru C. Methodological guidance for systematic reviews of observational epidemiological studies reporting prevalence and cumulative incidence data. Int J Evid Based Healthc. 2015;13(3):147-53.

16. Tewari N, Sultan F, Mathur VP, Rahul M, Goel S, Bansal K, et al. Global status of knowledge for prevention and emergency management of traumatic dental injuries in dental professionals: Systematic review and meta-analysis. Dent Traumatol. 2021;37(2):161-76.

17. Matell MS, Jacoby J. Is There an Optimal Number of Alternatives for Likert Scale Items? Study I: Reliability and Validity:. Educational and Psychological Measurement. 1971;31(3):657-74.

18. Schwarzer G, Carpenter JR, Rücker G. Meta-analysis with R: Springer; 2015. 264 p.

19. Borenstein M, Hedges LV, Higgins JP, Rothstein HR. A basic introduction to fixed-effect and random-effects models for meta-analysis. Res Synth Methods. 2010;1(2):97-111.

20. Cochran WG. The combination of estimates from different experiments. Biometrics. 1954;10(1):101-29.

21. Borenstein M, Higgins JP, Hedges LV, Rothstein HR. Basics of meta-analysis: I2 is not an absolute measure of heterogeneity. Res Synth Methods. 2017;8(1):5-18.

22. Barker TH, Migliavaca CB, Stein C, Colpani V, Falavigna M, Aromataris E, et al. Conducting proportional meta-analysis in different types of systematic reviews: a guide for synthesisers of evidence. BMC Med Res Methodol. 2021;21(1):1-9.

23. IntHout J, Ioannidis JP, Rovers MM, Goeman JJ. Plea for routinely presenting prediction intervals in meta-analysis. BMJ open. 2016;6(7):e010247.

24. Gaskin EB, Levy S, Guzman-Armstrong S, Dawson D, Chalmers J. Knowledge, attitudes, and behaviors of federal service and civilian dentists concerning minimal intervention dentistry. Mil Med. 2010;175(2):115-21.

25. Haider SA, Irfan M, Zahid N, Butt A, Arslan M, A Tariq M. Evidence based practice of minimal invasive dentistry among dentists. Pak J Sci. 2021;73(1):40-4.

26. Kakudate N, Yokoyama Y, Sumida F, Matsumoto Y, Yamazaki H, Touge T, et al. Evidence-practice gap in minimal intervention dentistry: Findings from a dental practice-based research network. J Dent. 2020;102:103469.

27. Kakudate N, Yokoyama Y, Sumida F, Matsumoto Y, Takata T, Gordan VV, et al. Web-based intervention to improve the evidence-practice gap in minimal intervention dentistry: Findings from a dental practice-based research network. J Dent. 2021;115:103854.

28. Katz CRT, De Andrade MDRB, Lira SS, Ramos Vieira EL, Heimer MV. The concepts of minimally invasive dentistry and its impact on clinical practice: A survey with a group of Brazilian professionals. Int Dent J. 2013;63(2):85-90.

29. Khan SI, Asghar S, Abid A, Aftab F. Awareness Regarding Minimally Invasive Dentistry among Dentists of Karachi. Journal of Bahria University Medical and Dental College. 2019;9(4):294-8.

30. Kumar S, Mala N, Rana KS, Namazi N, Rela R, Kumar K. Cognizance and use of minimally invasive dentistry approach by general dentists: An overlooked companion. Journal of Pharmacy And Bioallied Sciences. 2021;13(5):199.

31. Mirsiaghi F, Leung A, Fine P, Blizard R, Louca C. An investigation of general dental practitioners' understanding and perceptions of minimally invasive dentistry. British dental journal. 2018;225(5):420-4.

32. Natarajan K, Prabakar J. Knowledge, attitude, and practice on minimally invasive dentistry among dental professionals in Chennai. Drug Invention Today. 2019;11(8):1768-72.

33. Oliveira DC. Minimally Invasive Dentistry approach in Dental Public Health [M.S.]. Ann Arbor: The University of Iowa; 2011.

34. Oliveira DC, Warren JJ, Levy SM, Kolker J, Qian F, Carey C. Acceptance of minimally invasive dentistry among US dentists in public health practices. Oral Health and Preventive Dentistry. 2016;14(6):501-8.

35. Rayapudi J, Usha C. Knowledge, attitude and skills of dental practitioners of Puducherry on minimally invasive dentistry concepts: A questionnaire survey. Journal of Conservative Dentistry. 2018;21(3):257-62.

36. Shah AH, Sheddi FM, Alharqan MS, Khawja SG, Vohra FM, Akram Z, et al. Knowledge and attitude among general dental practitioners towards minimally invasive dentistry in Riyadh and AlKharj. Journal of Clinical and Diagnostic Research. 2016;10(7):90-4.

37. Suma G, Salman Y, Devadoss E. Knowledge, Attitude, Behavior, and Practice toward Minimal Intervention Dentistry among Dental Professionals in Bengaluru City, India. Journal of Health Sciences & Research,. 2017;8(1):20-4.

38. Fontana M, Zero D. Assessing patients' caries risk. Journal of the American Dental Association. 2006;137(9):1231-9.

39. Fontana M, Gonzalez-Cabezas C. Minimal intervention dentistry: part 2. Caries risk assessment in adults. British dental journal. 2012;213(9):447-51.

40. Mickenautsch S. High-viscosity glass-ionomer cements for direct posterior tooth restorations in permanent teeth: The evidence in brief. Journal of dentistry. 2016;55:121-3.

41. Mendes L, Pedrotti D, Casagrande L, Lenzi T. Risk of failure of repaired versus replaced defective direct restorations in permanent teeth: a systematic review and meta-analysis. Clinical oral investigations. 2022;26(7):4917-27.

42. Dawson AS, Makinson OF. Dental treatment and dental health. Part 2. An alternative philosophy and some new treatment modalities in operative dentistry. Australian dental journal. 1992;37(3):205-10.

43. Elouafkaoui P, Bonetti D, Clarkson J, Stirling D, Young L, Cassie H. Is further intervention required to translate caries prevention and management recommendations into practice? British dental journal. 2015;218(1):E1.

44. Schwendicke F, Doméjean S, Ricketts D, Peters M. Managing caries: the need to close the gap between the evidence base and current practice. British dental journal. 2015;219(9):433-8.

45. Fernández CE, González-Cabezas C, Fontana M. Minimum intervention dentistry in the US: an update from a cariology perspective. British dental journal. 2020;229(7):483-6.

**3. CONSTRUÇÃO, ADAPTAÇÃO E PROPRIEDADES PSICOMÉTRICAS: um estudo de CONHECIMENTOS, HABILIDADES E ATITUDES DOS CIRURGIÕES-DENTISTAS SOBRE A ODONTOLOGIA DE MÍNIMA INTERVENÇÃO.**

Autores: Regina Cardoso de Moura, Matheus França Perazzo, Soraya Coelho Leal, Lucianne Cople Maia de Faria, Carla Massignan.

RESUMO

**Objetivos:** Construir e avaliar as propriedades psicométricas de um questionário para mensurar os conhecimentos, habilidades e atitudes dos cirurgiões-dentistas acerca da Odontologia de Mínima Intervenção (OMI). **Métodos:** O questionário elaborado passou pela análise de uma equipe de brasileiros experts no tema. Foram realizados dois grupos focais e um pré-teste para averiguar a redação, sequência das perguntas e entendimento das questões. Os ajustes necessários foram realizados. Após aplicado, foi realizado Análise Fatorial Exploratória para avaliar a estrutura fatorial do questionário. Utilizou-se Análise Paralela com permutação aleatória dos dados. **Resultados:** Um total de 404 cirurgiões-dentistas compuseram a amostra final para os testes psicométricos da escala de conhecimentos, habilidades e atitudes em OMI. A análise paralela sugeriu que os itens do questionário se dividem em dois fatores. As cargas fatoriais dos itens foram adequadas em seus respectivos fatores. O questionário apresentou bons atributos na Análise Fatorial Exploratória e os achados sugerem evidência psicométrica do instrumento. **Conclusão:** Os achados foram positivos, com evidência de validade do questionário, e novos estudos são indicados para que sejam realizados novos testes, de forma a aumentar as evidências de validade.

**Palavras-chave:** Estudo de Validação. Análise Fatorial. Odontologia Baseada em Evidências. Cárie Dentária. Conhecimentos, Atitudes e Prática em Saúde.

3.1 INTRODUÇÃO

Para se obter um instrumento de medida com características psicométricas adequadas, um conjunto de etapas devem ser realizadas.(1) Vários autores concordam que os passos incluem conceitualizar o teste, revisar a literatura sobre o construto a ser estudado, criar os itens, aplicar os itens em uma amostra, analisar os itens, e revisar o teste.(2)

A primeira etapa inclui o desenvolvimento dos itens, e estão ligados à validade do instrumento, que são: a revisão da literatura, da teoria relacionada ao novo teste; processos complementares, como entrevistas e consultas a juízes; definição operacional e elaboração dos itens.(1) A partir da primeira etapa chega-se à versão preliminar do instrumento.(1) A segunda etapa relaciona-se à coleta de dados, que inclui: a aplicação da versão preliminar a grupos focais, à amostra-piloto e à amostra-alvo, que é a amostra a ser testada pelo instrumento construído.(1) Já a terceira etapa relaciona-se às análises estatísticas e análise do teste, bem como escrita de uma versão final.(1)

As propriedades psicométricas são variáveis que determinam a qualidade de um instrumento de avaliação e dentre elas estão a confiabilidade e a validade.(3) A capacidade com que um instrumento mede fielmente um acontecimento chama-se confiabilidade.(3) Já a capacidade com que um instrumento mede precisamente um acontecimento estudado chama-se validade.(3) A análise fatorial exploratória é usada para confirmar se o instrumento possui estrutura apropriada, a validade de construção.(4) Nesse caso, a validade estrutural é uma propriedade necessária na validade de construto, de forma a constituir a validade de um instrumento de medida.(5)

A análise fatorial exploratória é definida como uma reunião de técnicas multivariadas, que busca encontrar a configuração contida em uma matriz de dados e definir o número e origem das variáveis latentes, que são os fatores, que representam melhor um conjunto de variáveis.(6) Quando as variáveis observadas apresentam uma variância em comum, ou seja, são influenciadas pelo mesmo construto, elas pertencem a um mesmo fator.(6) O fator é definido como uma variável latente que influencia mais de uma variável observada, e representa a covariância entre elas.(7)

A análise fatorial exploratória é muito utilizada para a construção, avaliação e refinamento de instrumentos psicológicos(7). Ela é particularmente útil em instrumentos para medir personalidade, psicopatologias, atitudes, estilos comportamentais, esquema cognitivo, e outros construtos multidimensionais.(8) Na educação em saúde o conceito de competências profissionais tem caráter multidimensional.(9) Ele inclui as dimensões cognitiva, técnica, integrativa, contextual, interrelacional, afetiva/moral e dos hábitos mentais.(9) Assim, a competência decorre do desempenho em situações reais, resultado de uma aprendizagem complexa, combinando habilidades cognitivas, atitudinais e psicomotoras.(10-14)

Até o presente momento não foi verificado um instrumento com validade estrutural para avaliar competências dos cirurgiões-dentistas nos princípios da Odontologia de Mínima Intervenção. Esse estudo objetiva construir e avaliar as propriedades psicométricas de um questionário para mensurar os conhecimentos, habilidades e atitudes dos cirurgiões-dentistas brasileiros acerca da Odontologia de Mínima Intervenção (OMI).

3.2 METODOLOGIA

**3.2.1 População, Contexto e Período de coleta**

Um estudo transversal foi realizado a fim de validar o questionário criado para mensurar os conhecimentos, habilidades e atitudes em Odontologia de Mínima Intervenção.

O questionário foi idealizado na plataforma Google Forms, e a coleta de dados realizada online. Os participantes selecionados eram cirurgiões-dentistas que atuavam no Distrito Federal (DF) no momento da pesquisa. Aqueles profissionais que não tinham registro no Conselho Regional de Odontologia do DF (CRO-DF) foram excluídos do presente estudo.

Antes de passar pela avaliação psicométrica propriamente dita, o questionário foi avaliado por um painel de especialistas, os cirurgiões-dentistas que mais publicam no assunto de OMI, e dois grupos focais também composto por cirurgiões-dentistas. Os dados dessas etapas não foram utilizados para a avaliação psicométrica. O estudo foi realizado entre novembro de 2021 e março de 2023. Este estudo seguiu a Declaração de Helsinki e resolução 466/2012 do Conselho Nacional de Saúde Brasileiro, e foi submetido ao Comitê de Ética em Pesquisa da Faculdade de Ciências da Saúde (CEP/FS) da Universidade de Brasília (UnB), parecer CAAE: 47639021.8.0000.0030 (Anexo 1).

**3.2.2 Construção e Adaptação do questionário**

A construção do questionário foi baseada em estudos prévio de competências em OMI (15) e evidência disponível sobre a OMI.(16-18) O questionário proposto dividiu-se em 7 seções, disponíveis no Apêndice 7. As seções de 1 a 4 continham o Termo de Consentimento Livre e Esclarecido (TCLE), duas questões para seleção dos participantes, e questões para avaliar as características sociodemográficos dos participantes, formação e experiência prévia em OMI. As seções 5 a 7, tinham as questões para acessar os conhecimentos, habilidades e atitudes dos profissionais acerca da Odontologia de Mínima Intervenção, a serem respondidas por meio de escala Likert (1 a 5). As seções 3 e 8 continham duas questões para avaliar as barreiras para o conhecimento e a prática da OMI, também em escala Likert, e uma questão aberta, para sugestões e comentários adicionais dos participantes.

As seções sobre conhecimentos, habilidades e atitudes foram criadas com base no conceito de competências de Zarifian (19), desdobrando-se em três dimensões: do conhecimento, como a dimensão do saber; da habilidade como o saber-fazer e da atitude como a dimensão do querer-saber-fazer. Assim, nessas sessões, serão mensurados os conhecimentos, habilidades e atitudes dos cirurgiões-dentistas acerca da OMI. As perguntas para a avaliação das competências foram divididas em áreas temáticas baseadas nos quatro conceitos principais da Mínima Intervenção propostos por Walsh(18), reconhecimento, redução, regeneração e reparo (Quadro 1).

**Quadro 1 -** Questionário de conhecimentos, habilidades e atitudes sobre OMI

|  | **Competências avaliadas** | | |
| --- | --- | --- | --- |
| **Áreas Temáticas** | **Conhecimento** | **Habilidade** | **Atitude** |
| **OMI (Geral)** | **2.** O quanto você sabe sobre a Odontologia de Mínima Intervenção para o manejo da cárie dentária? | **7.** O quanto você concorda com a seguinte afirmação: O objetivo da Odontologia de Mínima Intervenção é manter os dentes saudáveis ​​e funcionais por toda a vida, e envolve a implementação de estratégias importantes para manter os dentes livres de lesões de cárie. Essas estratégias são a detecção precoce de cárie e avaliação de risco; remineralização do esmalte e dentina desmineralizados; medidas ótimas de prevenção de cárie; intervenções operatórias minimamente invasivas e reparo ao invés de substituição de restaurações". | **8.** Com que frequência você aplica a Odontologia de Mínima Intervenção no manejo da cárie dentária na sua prática clínica diária? |
| **Continua** | | | |
|  | | | |
|  | | | |
|  | | | |
|  | | | |
|  | | | |
| **Quadro 1 -** Questionário de conhecimentos, habilidades e atitudes sobre OMI | | | |
| **Reconhecimento, Redução e Remineralização** | **9.** O quanto você sabe sobre a identificação dos fatores de risco da doença cárie?  **13.** O quanto você sabe a respeito do controle do início da doença cárie?  **14.** O quanto você sabe a respeito do controle do avanço da doença cárie? | **10.** O quanto você concorda com a seguinte afirmação: "A avaliação da frequência do consumo de alimentos fontes de açúcares é um bom indicador de risco futuro para a doença cárie".  **11.** O quanto você concorda com a seguinte afirmação: "A exposição a fontes de flúor é um bom indicador de proteção para a doença cárie".  **15.** O quanto você concorda com a seguinte afirmação: "Em pacientes de alto risco para a doença cárie, a mudança de hábitos, tal como a redução da frequência de consumo de alimentos fontes de açúcares, é um fator importante para controlar o início da doença" | **12.** Com que frequência você avalia o risco à doença cárie dos seus pacientes?   **16.** Para os pacientes de alto risco para a doença cárie, com que frequência você orienta sobre a redução do consumo de alimentos fontes de açúcares ?   **17.** Para os pacientes de alto risco à doença cárie, com que frequência você orienta sobre a escovação dentária diária com pasta fluoretada? |
| **Restaurações minimamente invasivas e Reparo de restaurações** | **18.** O quanto você sabe sobre os procedimentos restauradores minimamente invasivos? | **19.** O quanto você concorda com a seguinte afirmação: "A remoção de dentina cariada diferencia-se de acordo com a profundidade das lesões de cárie, devendo ser mais conservadora em lesões profundas, a fim de evitar exposição pulpar em dentes vitais".   **21**. O quanto você concorda com a seguinte afirmação: "Em restaurações defeituosas, deve-se considerar o reparo antes de se optar pela remoção da restauração e a realização de uma nova". | **20.** Em restaurações de lesões de cárie profundas de dentes vitais, com que frequência você deixa dentina cariada amolecida nas paredes de fundo da cavidade para evitar exposição pulpar?  **22.** Na abordagem de restaurações defeituosas, com que frequência você realiza o reparo em vez de realizar a troca total da restauração? |

Incialmente o questionário foi avaliado por um painel de especialistas brasileiros no assunto. Foram considerados especialistas os pesquisadores brasileiros que mais produziram em OMI, no período de 1996 até 2021. Os pesquisadores foram selecionados por meio de busca na base de dados SCOPUS. A estratégia de busca foi feita com os seguintes descritores em ciências da saúde *DeCS/Mesh* associados: (*"Minimal Intervention Dentistry"*) AND (*"Dentists*") AND (*“Health Knowledge, Attitudes, Practice*") mais termos correlatos, conforme o Apêndice 8. Foi feito contato com esses pesquisadores por meio de endereço eletrônico disponível em suas publicações recentes e enviado o questionário do presente estudo, com convite solicitando participação voluntária e anônima, para avaliação quanto ao conteúdo técnico-científico, redação e entendimento dos itens. Os especialistas que concordaram em participar após leitura do Termo de Consentimento Livre e Esclarecido, foram encaminhados para as seções seguintes do questionário e foram solicitados a responder as perguntas do presente estudo. As considerações dos especialistas foram feitas dentro de cada item em um campo de respostas em escala Likert (1-4), sendo 1= não relevante, 2=pouco relevante, 3=relevante, 4=muito relevante. Também havia um campo para preenchimento de sugestões em cada item estudado. Os itens pontuados com 1 ou 2 por 10% ou mais dos especialistas foram reformulados,(20) considerando as sugestões enviadas, até se atingir o consenso da equipe de pesquisa.(20)

Após as adaptações necessárias, este questionário foi testado em dois grupos focais para avaliar o entendimento dos itens pela população-alvo. Esses grupos foram compostos por três e dois profissionais respectivamente, de especialidades distintas da odontologia, e estes não participaram das demais etapas da pesquisa. Os encontros para leitura e discussão dos itens foram realizados online, em uma plataforma de reuniões, com o envio prévio do questionário aos participantes, bem como projeção do questionário no momento da reunião. Todos os itens foram lidos de forma pausada, e a cada item, os participantes eram interrogados sobre o entendimento do item e se havia algum comentário adicional. Todas as sugestões foram anotadas. Após, as mudanças consensuais foram introduzidas pelos autores ao instrumento, de forma a melhorar o entendimento e legibilidade dos itens.

**3.2.3 Avaliação de Evidências Psicométricas**

Um total de 404 cirurgiões-dentistas do Distrito Federal completaram as respostas de pesquisa e foram incluídos no presente estudo.

A avaliação de confiabilidade e validade foi feita de acordo com a lista de verificação COSMIN, padrão baseado em consenso para a seleção de instrumentos de medição em saúde.(21)

Antes da coleta de dados propriamente dita, o questionário passou por um pré-teste com 30 cirurgiões-dentistas, que seriam os juízes-avaliadores, para averiguar a clareza, adequação e compreensão dos itens. Nessa etapa, utilizou-se o Coeficiente de Validade de Conteúdo (CVC) para verificar se os itens estavam adequados para a pesquisa.(22) O questionário foi enviado em formulário online com todos os itens identificados na versão preliminar, na qual cada juiz-avaliador analisou cada item do questionário a partir de três critérios: a clareza: “a linguagem está clara?”; adequação: “a linguagem é adequada para cirurgiões-dentistas” e compreensão: “a pergunta é compreensível?”. A avaliação dos itens foi feita com base numa escala do tipo Likert que variou de um a cinco, sendo que um representava “discordo totalmente”; e cinco, “concordo totalmente”. Após cada juiz-avaliador responder ao questionário, efetuou-se o cálculo do CVC.(22) Calcula-se o CVC com base nas notas dos juízes (1 a 5), obtendo-se a média das notas de cada item (Mx).(23) Utilizando-se essa média (Mx), calcula-se então o CVC inicial para cada item (CVCi), dividindo-se pelo maior valor que a questão poderia receber de clareza, adequação ou compreensão.(23) Em seguida calcula-se o erro (Pei), de forma a descontar algum viés dos juízes avaliadores em cada questão.(23) Para tal, divide-se um (1) pelo número de juízes avaliadores, elevado pelo mesmo número de avaliadores.(23) Assim, o CVC final de cada item (CVCc) poderá ser calculado a partir da subtração do CVCi pelo Pei.(23) Ao final, calcula-se o CVC total do questionário (CVCt), para cada uma das características, com a subtração da média do CVCi (MCVCi) pela média do Pei (MPei).(23) Consideraram-se aceitáveis os itens do questionário que obtiveram CVC> 0,8.(22) Poucas correções foram realizadas, e todos os itens obtiveram CVC>0,8, sendo o CVC geral de 0,93. Com a finalização dessa etapa, as respostas dos voluntários foram desconsideradas.

**3.2.4 Medidas**

3.2.4.1 Conhecimentos, habilidade e atitudes sobre OMI

Essa seção contava com 17 itens estruturados, respondidos por meio de escala Likert (1 a 5), para acessar os conhecimentos, habilidades e atitudes acerca da OMI. Quanto maior o escore, maior a competência avaliada pelo item.

3.2.4.2 Barreiras para a OMI

Essa seção era composta por dois itens estruturados respondidos por meio de escala Likert (1 a 5) para acessar as barreiras para o conhecimento e prática da OMI. Quanto maior o escore, pior o item era avaliado, o que verificaria a presença da dificuldade avaliada.

3.2.4.3 Dados sociodemográficos, perfil e experiência em OMI

Nessa seção foram avaliados: sexo, idade, região de moradia, tipo de instituição de graduação, tempo de experiência, maior titulação, especialidade de pós-graduação, tempo desde a última pós-graduação, atuação profissional principal, treinamento prévio em OMI, local de treinamento, fonte de informação sobre OMI.

**3.2.5 Plano de análise de dados**

Para realizar o tratamento de dados e análise estatística foram utilizados o SPSS, v. 23.0 e o programa Factor 11.05. A Análise Fatorial Exploratória (AFE) foi realizada para avaliar a estrutura fatorial da escala de conhecimentos, habilidades e atitudes em Odontologia de Mínima Intervenção. A análise foi realizada com matriz policórica e com o método de extração *Robust Diagonally Weighted Least Squares* (RDWLS).(24) Para decisão sobre a quantidade de fatores a serem retidos, foi realizada a Análise Paralela com permutação aleatória dos dados (25) e rotação com a *Robusti Promin*.(26) A fidedignidade composta foi calculada com base nas cargas fatoriais padronizadas e variância de erro.(27)

A avaliação do modelo foi realizada com os índices de ajuste *Root Mean* *Square Error of Aproximation* (RMSEA), *Comparative Fit Index* (CFI) e *Tucker-Lewis Index* (TLI).(28) Adotou-se os seguintes limites para julgar o ajuste do modelo: RMSEA<0,06; CFI>0,90; TLI>0,90 para um ajuste adequado.(28) Já a unidimensionalidade do construto foi confirmada com os índices a seguir: *Unidimensional Congruence* (ÚNICO) >0,95; *Explained Commom Variance* (ECV)>0,80 *e Mean of Item Residual Absloute Loadings* (MIREAL)>0,30.

3.3 RESULTADOS

Os dois grupos focais realizados foram compostos por três e dois profissionais respectivamente, todos do sexo feminino e com uma média de idade de 44 anos (IC 33-52). Todos os CDs tinham especialização como maior titulação, e alguns possuíam 2 ou mais especialidades. As especialidades eram prótese dentária (40%), implantodontia (20), ortodontia (120%), dentística (120%), odontologia do trabalho (20%), odontopediatria (20%) e endodontia (20%).

Já no pré-teste, participaram 30 CDs, sendo 73,3% (n=22) do sexo feminino, 23,2% (n=7) do sexo masculino e 1 participante preferiu não responder. A média de idade foi de 35 anos (IC 24-49). A maioria dos CDS possuía especialização como maior titulação (53,3%), seguida por mestrado (20%). Alguns profissionais possuíam 2 ou mais especialidades. As especialidades eram odontopediatria (26,7%), ortodontia (23,3%), prótese dentária (20%), dentística (10%), disfunção temporomandibular e dor orofacial (10%), harmonização orofacial (10%), endodontia (6,7%), odontogeriatria (6,7%), ortopedia facial (6,7%), implantodontia (3,3%), odontologia em saúde coletiva (3,3%), odontologia para pacientes especiais (3,3%), radiologia odontológica (3,3%).

Dos 454 CDs que acessaram o questionário, excluindo-se os profissionais que acessaram e não concordaram em participar ou acessaram e não eram elegíveis para o estudo (n=50; 11,0%), um total de 404 cirurgiões-dentistas compuseram a amostra final para os testes psicométricos da escala de conhecimentos, habilidades e atitudes em OMI. Das características dos participantes, destaca-se sexo predominante o feminino (74%), atuação em clínica privada própria ou compartilhada (66,6%) e especialização como maior titulação (61,1%).

O resultado dos testes de esfericidade de Barlett (2481,7, df =120, p<0,01) e KMO (0,83) apontaram para interpretabilidade da matriz de correlação de itens. A Análise Paralela sugeriu dois fatores como os mais representativos para os dados, já que dois fatores dos dados reais apresentam porcentagem de variância explicada maior do que os dados aleatórios, conforme pode ser visto na Tabela 1.

**Tabela 1.** Resultado da Análise Paralela

| Fatores | Percentual de variância explicada dos dados reais | Percentual de variância dos dados da média aleatória | Percentual de variância explicada dos dados aleatórios (95% IC) |
| --- | --- | --- | --- |
| 1  2  3  4  5  6  7  8  9  10  11  12  13  14  15 | 38.2726**  12.7368*  9.5311  8.1820  5.8625  5.1317  4.4570  3.7767  3.3394  2.3102  2.0638  1.6757  1.4511  0.9662  0.2431 | 12.6969  11.6750  10.7500  9.8921  9.0254  8.1825  7.3816  6.5856  5.7393  4.9927  4.2299  3.4514  2.6278  1.8231  0.9468 | 14.4680  13.0629  11.9948  10.8499  9.9117  8.9531  8.1195  7.3359  6.5245  5.8941  5.1903  4.3903  3.6245  2.7580  1.8121 |

Numa análise preliminar houve padrão de cargas cruzadas*, ou crossloading,* em um item (“Com que frequência você aplica a Odontologia de Mínima Intervenção no manejo da cárie dentária na sua prática clínica diária?”) com cargas fatoriais de 0.383 e 0.325 nos respectivos fatores 1 e 2. Optou-se por remover o item de *crossloading* e realizar nova análise. Após, mantiveram-se os dois fatores e a estrutura contemplou razoavelmente bem a divisão teórica dos itens, sendo o fator 1 (F1) atribuído à dimensão do Conhecimentos e o fator 2 (F2) à dimensão das Habilidade e Atitudes. Apenas o item 6, “Com que frequência você avalia o risco a doença cárie dos seus pacientes” não foi contemplado na dimensão prevista teoricamente, de Habilidades e Atitudes. As cargas fatoriais dos itens e os índices de Fidedignidade Composta podem ser vistos na Tabela 2.

**Tabela 2.** Estrutura Fatorial de Competências em OMI

|  | Fator 1 | Fator 2 |
| --- | --- | --- |
| Itens | Conhecimentos em OMI | Habilidade e Atitude em OMI |
| Item 1 | **0.498** | 0.285 |
| Item 2 | -0.002 | **0.626** |
| Item 3 | **0.709** | -0.104 |
| Item 4 | -0.108 | **0.589** |
| Item 5 | 0.026 | **0.377** |
| Item 6 | **0.456** | 0.078 |
| Item 7 | **0.880** | -0.045 |
| Item 8 | **1.008** | -0.162 |
| Item 9 | -0.050 | **0.598** |
| Item 10 | 0.007 | **0.499** |
| Item 11 | 0.141 | **0.395** |
| Item 12 | **0.599** | 0.205 |
| Item 13 | 0.110 | **0.452** |
| Item 14 | 0.154 | **0.403** |
| Item 15 | -0.099 | **0.718** |
| Item 16 | -0.023 | **0.714** |
| Fidedignidade Composta | 0.856 | 0.806 |

*OMI=Odontologia de Mínima Intervenção

A maioria dos itens tiveram cargas fatoriais adequadas e elevadas em seus fatores. Nessa segunda análise não foram encontrados padrão de cargas cruzadas, ou *crossloading.* Com relação a fidedignidade composta, essa também se mostrou adequada para todos os fatores, já que todas foram acima de 0,70. Já a estrutura fatorial apresentou índices de ajuste adequados: χ^2^=235,09, *gl*=89; p>0,001; CFI=0.934; TLI=0.91, exceto para o RMSEA (RMSEA=0.092), o qual foi considerado medíocre. A unidimensionsalidade foi rejeitada, já que os valores de UNICO= 0.939 ( 0.907- 0.975) e ECV= 0.762 (0.719-0.830) foram abaixo do necessário, e apenas MIREAL=0.288 (0.254-0.323) atingiu o necessário.

3.4 DISCUSSÃO

O objetivo do presente estudo foi elaborar e avaliar as propriedades psicométricas de um questionário para mensurar os conhecimentos, habilidades e atitudes dos cirurgiões-dentistas brasileiros acerca da Odontologia de Mínima Intervenção (OMI). O estudo apresentou os resultados das etapas de construção e análises fatoriais exploratórias realizadas.

A unidimensionalidade foi rejeitada e na multidimensionalidade testada, a estrutura interna foi apresentada com um modelo de dois fatores, confirmado na análise teórica realizada pelos pesquisadores. Ressalta-se que a multidimensionalidade é inerente aos estudos de competências, o que suporta o modelo teórico criado e o modelo fatorial encontrado na análise fatorial exploratória.

As cargas fatoriais apresentadas na extração tiveram valores positivos, já que a carga fatorial mínima é de 0,3 para ser aceitável, apesar de poderem ocorrer variações a depender do tipo de estudo.(29) Os itens se ajustaram bem aos fatores, com exceção para o item seis, que apesar de ter carga fatorial positiva para a dimensão do conhecimento, no processo de validação de conteúdo esse item apresentou significância conceitual para a outra dimensão, de habilidades e atitudes.

Na fidedignidade composta, os valores encontrados para os dois fatores foram adequados. Ressalta-se que esse modelo foi escolhido como estimador de confiabilidade composta por não gerar subestimação, como é o caso do coeficiente de Cronbach.(27)

Houve uma tendência de respostas positivas para os itens avaliados. Recomenda-se que o estudo seja replicado em contextos diferentes para que se confirme se a solução fatorial acontecerá de forma semelhante. Em razão do item de atitude “Com que frequência você aplica a Odontologia de Mínima Intervenção no manejo da cárie dentária na sua prática clínica diária?” ter apresentado carga fatorial acima de 0,3 nos dois fatores, e do item também de atitude “Com que frequência você avalia o risco à doença cárie dos seus pacientes?” não terem tido uma boa disposição, seria oportuno realizar novas análises em estudos futuros com amostras diferentes.

3.5 CONCLUSÃO

O instrumento construído para avaliar os conhecimentos, habilidades e atitudes dos cirurgiões-dentistas em Odontologia de Mínima Intervenção apresentou bons atributos na Análise Fatorial Exploratória e os achados sugerem evidência psicométrica do instrumento. Novos estudos são indicados para que sejam realizados novos testes, de forma a aumentar as evidências de validade.

**REFERÊNCIAS**

1. Hutz CS, Bandeira DR, Trentini CM. Psicometria. Porto Alegre: Artmed; 2015. 192 p.

2. Cohen RJ, Swerdlik ME, Sturman ED. Testagem e Avaliação Psicológica-: Introdução a Testes e Medidas. 8. ed. ed. Porto Alegre: Amgh Editora; 2014. 756 p.

3. Pilatti LA, Pedroso B, Gutierrez GL. Propriedades Psicométricas de Instrumentos de Avaliação: Um debate necessário. RBECT. 2010;3(1):81-91.

4. Fayers P, Hand D. Factor analysis, causal indicators and quality of life. Qual Life Res. 1997;6(2):139-50.

5. Mokkink L, Terwee C, Patrick D, Alonso J, Stratford P, Knol D, et al. International consensus on taxonomy, terminology, and definitions of measurement properties for health-related patient-reported outcomes: results of the COSMIN study. J Clin Epidemiol. 2010;63(7373):745.

6. Brown TA. Confirmatory factor analysis for applied research. New York: The Guilford Press; 2006.

7. Damásio BF. Uso da análise fatorial exploratória em psicologia. Avaliaçao Psicologica: Interamerican Journal of Psychological Assessment. 2012;11(2):213-28.

8. Floyd FJ, Widaman KF. Factor analysis in the development and refinement of clinical assessment instruments. Psychological Assessment. 1995;7(3):286-99.

9. Hojat M, Veloski J, J NT, Erdmann JB, Gonnella JS. Assessing physicians' orientation toward lifelong learning. Journal of general internal medicine. 2006;21(9):931–6.

10. Perrenoud P. Construindo as competências desde a escola. Porto Alegre: Artmed; 1999. 90 p.

11. Morin E. Os Sete Saberes Necessarios a Educacao do Futuro. Sustinere-Revista de Saude e Educacao. 2016;4(1):161-2.

12. Lucchese R, Barros S. Pedagogia das competências um referencial para a transição paradigmática. no ensino de enfermagem: uma revisão da literatura. Acta Paulista de Enfermagem. 2006;19:92-9.

13. Van Merriënboer JJ, Kirschner PA. Ten steps to complex learning: A systematic approach to four-component instructional design. United Kingdom.: Routledge; 2017.

14. Educação CNd. Parecer CNE/CES nº 116/2014. Diretrizes curriculares nacionais do curso de graduação em medicina. Diário Oficial da União. 2014.

15. Kakudate N, Yokoyama Y, Sumida F, Matsumoto Y, Yamazaki H, Touge T, et al. Evidence-practice gap in minimal intervention dentistry: Findings from a dental practice-based research network. J Dent. 2020;102:103469.

16. Frencken JE, Peters MC, Manton DJ, Leal SC, Gordan VV, Eden E. Minimal intervention dentistry for managing dental caries–a review: report of a FDI task group. Int Dent J. 2012;62(5):223-43.

17. Innes N, Frencken J, Bjørndal L, Maltz M, Manton D, Ricketts D, et al. Managing Carious Lesions: Consensus Recommendations on Terminology. Advances in dental research. 2016;28(2):49-57.

18. Walsh LJ, Brostek AM. Minimum intervention dentistry principles and objectives. Aust Dent J. 2013;58:3-16.

19. Zarifian P. Objetivo competência: por uma nova lógica; tradução.2001. 197 p.

20. Polit DF, Beck CT. The content validity index: are you sure you know what's being reported? Critique and recommendations. Research in nursing & health. 2006;29(5):489-97.

21. Terwee CB. COSMIN checklist with 4-point scale. Amsterdam: Cosmin; 2011.

22. Pasquali L. Instrumentação psicológica: fundamentos e práticas. Porto Alegre: Artmed; 2010. 560 p.

23. Silveira MB, Saldanha RP, Leite JCdC, Silva TOFd, Silva T, Filippin LI. Construção e validade de conteúdo de um instrumento para avaliação de quedas em idosos. Einstein (São Paulo). 2018;16:1-8.

24. Asparouhov T, Muthén B. Simple second order chi-square correction. Mplus technical appendix. 2010:1-8.

25. Timmerman M, Lorenzo-Seva U. Evaluación de la dimensionalidad de elementos politómicos ordenados con análisis paralelo. Psychol Methods. 2011;16(2):209-20.

26. Lorenzo-Seva U, Ferrando PJ. Robust Promin: a method for diagonally weighted factor rotation. LIBERABIT Revista Peruana de Psicología. 2019;25(1):99-106.

27. Raykov T. Estimation of Composite Reliability for Congeneric Measures. Applied Psychological Measurement. 1997;21(2):173–84.

28. Hu L, Bentler PM. Cutoff criteria for fit indexes in covariance structure analysis: Conventional criteria versus new alternatives. Structural equation modeling: a multidisciplinary journal. 1999;6(1):1-55.

29. Pett MA, Lackey NR, Sullivan JJ. Making Sense of Factor Analysis: the use of fator analysis for instrument development in health care research. California: Sage; 2003 2023-06-07. 368 p.

**4.** **Conhecimentos, habilidades, atitudes E BARREIRAS DOS CIRURGIÕES DENTISTAS do distrito federal SOBRE A ODONTOLOGIA DE MÍNIMA INTERVENÇÃO: ESTUDO TRANSVERSAL**

Autores: Regina Cardoso de Moura, Matheus França Perazzo, Soraya Coelho Leal, Lucianne Cople Maia de Faria, Carla Massignan.

RESUMO

**Objetivos:** Avaliar os conhecimentos, habilidades, atitudes e barreiras dos cirurgiões-dentistas (CDs) do Distrito Federal (DF), sobre a Odontologia de Mínima Intervenção, e testar associações com perfil e formação desses profissionais. **Métodos:** Realizou-se um estudo transversal, quantitativo, baseado em questionário online autorreferido. O questionário, previamente validado, foi enviado pelas mídias sociais aos CDs. Os dados foram analisados no SPSS, apresentados com estatística descritiva e inferencial, e foi realizada regressão linear múltipla. O nível de significância adotado foi de 5% (p<0,05). **Resultados:** Um total de 454 cirurgiões-dentistas acessaram o questionário, e excluindo-se os profissionais que acessaram e não concordaram em participar ou acessaram e não eram elegíveis para o estudo (n=50; 11,0%), uma amostra de 404 participantes foi obtida. A maioria dos respondentes, 74%, era do sexo feminino e um tempo de experiência médio de 12 (±10,9) anos. O item com maior porcentagem de avaliações positivas foi obtido no item de prática acerca da orientação sobre a escovação dentária diária com pasta fluoretada em que 95,6% relataram realizá-la; a pior porcentagem foi no item sobre as atitudes acerca da remoção seletiva de dentina cariada em cavidades muito profundas, com 38,6%. Em relação as barreiras, o item sobre remuneração adequada dos procedimentos de mínima intervenção apresentou maior percentual de profissionais que o avaliaram de forma desfavorável, com 38,3%. **Conclusão:** Este estudo mostrou uma proporção geral de conhecimentos, habilidades e atitudes dos profissionais adequada, com exceção de atitudes em remoção seletiva de dentina cariada. Conclui-se que, em geral, os CDs do DF possuem competências adequadas em OMI, porém há dificuldades a serem enfrentadas para a prática da mínima intervenção.

**Palavras-chave:** Cárie Dentária; Odontologia Baseada em Evidências; Conhecimento; Aptidão; Atitude do Pessoal de Saúde; Lacunas da Prática Profissional.

4.1 INTRODUÇÃO

A Odontologia de Mínima Intervenção (OMI) é a abordagem moderna em saúde bucal baseada em evidência que objetiva a preservação de estrutura dental e longevidade dentária, aumentando também a saúde bucal com um todo e bem-estar do indivíduo.(1) Os seus princípios podem ser divididos como segue: 1) Reconhecimento, envolvendo a identificação e avaliação precoce de fatores ao risco de cárie ; 2) Redução, com eliminação ou minimização dos fatores de risco de cárie identificados; 3) Regeneração, com a interrupção, reversão e recuperação de lesões com agentes tópicos apropriados; 4) Reparo, quando existe uma cavidade, com a intervenção cirúrgica preservando ao máximo a estrutura dentária.(2)

A lacuna entre a evidência científica mais atual e a prática clínica realizada sempre existiu, o que é chamado de lacuna entre evidência e prática.(3) Isso pode ser visto também na OMI, já que, apesar da evidência atual recomendar tratamentos não invasivos e não restauradores em lesões de cárie incipientes, uma significante parcela de dentistas ainda parece atuar de forma muito invasiva.(3) Uma revisão sistemática sobre o manejo dos cirurgiões-dentistas para lesões de cárie profundas em dentes permanentes revelou que em quase metade dos estudos os dentistas não adotam estratégias baseadas em evidência.(3)

A forma como os profissionais de saúde bucal manejam a doença cárie vem se tornando um dos focos principais para a redução do seu dano global.(4) Diversas pesquisas ao redor do mundo vêm estudando as competências dos cirurgiões-dentistas englobando os princípios gerais da OMI, porém grande parte destes estudos tiveram problemas metodológicos.(5) Uma revisão sistemática prévia sobre os conhecimentos, atitudes e práticas dos cirurgiões-dentistas sobre a mínima intervenção revelou que apesar do conhecimento apresentado, os profissionais ainda possuem limitações na sua prática.(5)

Um estudo brasileiro avaliou as competências sobre os princípios gerais da OMI, explorando de forma breve as barreiras, e contou com uma amostra pequena.^{KATZ, 2013, The concepts of minimally invasive dentistry and its impact on clinical practice: a survey with a group of Brazilian professionals}{KATZ, 2013, The concepts of minimally invasive dentistry and its impact on clinical practice: a survey with a group of Brazilian professionals}^(6) Portanto, ressalta-se a importância de melhor explorar as competências e as barreiras da mínima intervenção no contexto brasileiro e regional. O presente estudo objetivou avaliar os conhecimentos, habilidades, atitudes e barreiras dos cirurgiões-dentistas (CDs) do Distrito Federal (DF), sobre a Odontologia de Mínima Intervenção, e suas associações com o perfil e formação desses profissionais.

4.2. METODOLOGIA

**4.2.1 Caracterização da pesquisa**

Trata-se de um estudo transversal, quantitativo, baseado em questionário online autorreferido. Este estudo foi submetido ao Comitê de Ética em Pesquisa da Faculdade de Ciências da Saúde (CEP/FS) da Universidade de Brasília (UnB), parecer CAAE: 47639021.8.0000.0030 (ANEXO 1), e seguiu a Declaração de Helsinki e resolução 466/2012 do Conselho Nacional de Saúde Brasileiro. O estudo está reportando seguindo as recomendações das diretrizes do STROBE (Strengthening the Reporting of Observational Studies in Epidemiology) (Apêndice 9).(7)

**4.2.2 População e Seleção da amostra**

Este estudo teve como população-alvo os cirurgiões-dentistas atuantes no Distrito Federal (DF). O estudo foi aplicado online por meio de um questionário semiestruturado, via Google Forms, entre agosto de 2022 e março de 2023. Para o presente estudo, foram critérios de inclusão ser cirurgião-dentista e atuar profissionalmente no Distrito Federal. Foram excluídos do presente estudo os cirurgiões-dentistas sem inscrição ativa no Conselho Regional de Odontologia do Distrito Federal no momento da pesquisa.

**4.2.3 Instrumento e Coleta dos dados**

A construção do questionário foi baseada em estudos prévios(8) e evidências disponíveis sobre a OMI.(2, 9, 10) O questionário dividiu-se em 7 seções (Apêndice 7). As seções 1, 2, 3 e 4 continham o Termo de Consentimento Livre e Esclarecido (TCLE), confirmação do interesse em participar da pesquisa, registro no conselho local, e questões para acessar as características sociodemográficos dos participantes, formação e experiência prévia em OMI. As seções 5, 6 e 7, continham as questões para acessar os conhecimentos, habilidades e atitudes dos profissionais acerca da OMI, a serem respondidas por meio de escala Likert de 5 pontos. As seções 3 e 8 continham duas questões estruturadas de múltipla escolha para acessar as barreiras para o conhecimento e a prática da OMI, e uma questão subjetiva, para sugestões e comentários adicionais dos participantes.

As seções 5, 6 e 7 foram criadas com base no conceito de competências de Zarifian(11), desdobrando-se em três dimensões: do conhecimento, como a dimensão do saber; da habilidade como o saber-fazer e da atitude como a dimensão do querer-saber-fazer. Assim, nessas sessões, foram mensurados os conhecimentos, habilidades e atitudes dos cirurgiões-dentistas acerca da OMI. As perguntas para a avaliação das competências foram divididas em áreas temáticas baseadas nos quatro conceitos principais da Mínima Intervenção propostos por Walsh(2), reconhecimento, redução, regeneração e reparo. As perguntas específicas sobre cada área temática foram então elaboradas a partir de adaptações da literatura da Mínima Intervenção(2, 9, 10) e de uma pesquisa prévia que estudou competências em Mínima Intervenção(12), conforme o quadro no Apêndice 10.

Antes de ser aplicado esse questionário passou por um processo de adaptação e avaliação psicométrica, previamente relatado, com avaliação de 12 especialistas brasileiros, dois grupos focais, um pré-teste com 30 cirurgiões-dentistas, e análise fatorial exploratória para as perguntas de competências.

Após a finalização das adaptações do questionário no Google Forms, foram criados um link e QR code para compartilhamento e resposta dos participantes. Tendo em vista a impossibilidade de acessar os dados de todos os cirurgiões-dentistas do DF para realizar uma amostragem aleatória, foram então utilizadas estratégias para se alcançar a população-alvo, com o objetivo de aumentar o alcance de participantes(13) e reduzir o viés de seleção. Enviou-se o questionário por link e QR code das seguintes maneiras: 1) por e-mail, em uma lista de transmissão de uma Clínica Radiológica parceira e do Sindicato dos Odontologistas do DF; 2) pelas mídias sociais, em grupos e contas individuais dos profissionais (Whatsapp, Facebok e Instagram); 3) por meio de folders, entregues nas clínicas odontológicas do Distrito Federal. Para todos os profissionais que eram convidados a participar, era solicitado que encaminhasse o convite para um ou mais colegas que preenchessem os critérios de inclusão, de forma a aumentar a quantidade de profissionais que receberiam o convite para participar de pesquisa, método esse conhecido como bola de neve.(14)

A quantidade total de cirurgiões-dentistas com inscrição ativa no Conselho Regional de Odontologia (CRO) no DF era de 8944 cirurgiões-dentistas em junho de 2022. Conforme a revisão sistemática prévia realizada(5), os profissionais têm conhecimento em OMI mas ainda possuem limitações na sua prática. A partir do estudo piloto realizado, dentre os profissionais que tinham conhecimento em OMI, aqueles que apresentaram alguma dificuldade para sua prática foram 40% (n=6). Dessa forma, foi realizado o cálculo amostral (http://calculoamostral.bauru.usp.br/calculoamostral/) considerando um intervalo de confiança de 95%, erro amostral de 5%, efeito do desenho de 1,2 para aumentar a precisão,(15) e 10% de perda de participantes, totalizando uma amostra necessária de 473, e retirando as perdas, 426 cirurgiões-dentistas. As perdas referiam-se aos casos dos participantes que acessaram o questionário mas não concordaram em participar ou que não eram elegíveis para o estudo

**4.2.4 Metodologia de análise e interpretação dos dados**

Os dados com todas as respostas dos participantes foram consolidados em planilha Excel® e exportados para o programa Statistical Package for Social Sciences (SPSS para Windows, versão 21.0, SPSS Inc. Chicago, IL, EUA).

A análise descritiva e inferencial foi realizada para apresentação do perfil sociodemográfico, formação, experiência dos cirurgiões-dentistas, conhecimentos, habilidades, atitudes e barreiras, os quais foram expressos em média e desvio-padrão para as variáveis quantitativas ou em frequência absoluta e relativa para as variáveis qualitativas nominais e ordinais.

O resultado da avaliação das competências e barreiras dos profissionais foi apresentado sob a forma de frequência absoluta e relativa. Ainda, para descrever as competências pelo nível de aferição, a fim de sintetizar os achados, os níveis 4 e 5 da escala Likert, níveis mais altos das escalas, foram consideradas como cumprimento ao item em análise e expressos como frequência absoluta e relativa. Para as barreiras, os níveis 4 e 5 somados, expressam o não cumprimento ao item em análise, o que significa a existência de fato de uma dificuldade com aquele item.

Para sintetizar a análise, foram adotados os seguintes critérios previamente relatados (16): frequência de até 25% foi considerada como competência 'insuficiente', entre 26% e 50% como 'razoável', entre 51 e 75% como 'boa ', e entre 76 e 100% como 'excelente'.

As variáveis independentes (conhecimentos, habilidades, atitudes, barreiras para conhecimento, barreiras para prática) foram categorizadas, conforme disposto no Quadro 1 do Apêndice 11. Em seguida, foi realizado teste de normalidade utilizando o Teste de Shapiro-Wilk. Não houve normalidade da distribuição. Realizou-se análise gráfica dos resíduos dos modelos de regressão múltipla e estes apresentaram distribuição normal, então a regressão linear múltipla foi mantida (17) e não houve violação dos outros pressupostos da regressão linear múltipla.(18) Foi realizada Correlação linear de Spearman e regressão linear simples. Todas as variáveis com valor de p ≤ 0,20 na regressão simples foram adicionadas no modelo de regressão múltipla além das variáveis de ajuste, sendo elas “Já ouviu falar em OMI” e “Receber treinamento em OMI”.

4.3 RESULTADOS

**4.3.1 Participantes e Características**

Um total de 454 cirurgiões-dentistas acessaram o questionário, e excluindo-se os profissionais que acessaram e não concordaram em participar ou acessaram e não eram elegíveis para o estudo (n=50; 11,0%), um total de 404 cirurgiões-dentistas do Distrito Federal completaram as respostas de pesquisa e foram incluídos no presente estudo.

Os resultados do questionário socioeconômico sobre o perfil e formação profissional dos participantes estão descritos nas Tabelas 1 e 2.

Ressalta-se que a maioria de participantes era do sexo feminino (n=299; 74%), atuava em clínica privada própria ou compartilhada (n=269; 66,6%), possuía especialização como maior titulação (n=247; 61,1%). Dentre as especialidades as mais frequentes eram odontopediatria (n=79; 12,4%), implantodontia (n=70; 11,0%), prótese dentária (n=65; 10,2%), dentística (n=56; 8,8%) e endodontia (n=51; 8,0%), e cabe mencionar que havia profissionais com mais de uma especialidade. Na pergunta de pós-graduações em outras áreas, as mais frequentes na área de saúde foram em saúde pública/coletiva/família (n= 11; 22,9%), odontologia hospitalar (n=6; 12,5%), ciências da saúde (n=3, 6,25%), e em áreas distintas à saúde como gestão e administração (n=11; 22,9%), direito (n=4; 8,3%) e educação (n=3; 6,2%).

Com relação a formação em OMI (Tabela 3), a grande maioria dos profissionais relatou já ter recebido treinamento (n=290, 71,8%), e dentre os treinamentos as fontes mais frequentes foram na graduação (n=121; 41,7%) e em cursos livres, como cursos de aperfeiçoamento, atualização, extensão (n=101; 33,2%). Já com relação a fonte de informações mais buscadas acerca da OMI, as mais frequentes relatadas foram cursos ou palestras (n=134; 33,2%), seguido por artigos científicos em inglês e outros idiomas (n=95; 23,5), ressaltando-se também que 16,3% (n=66) dos profissionais relataram não buscar informações sobre OMI.

**Tabela 1** -Perfil e formação dos cirurgiões-dentistas do Distrito Federal em 2023.

| Variáveis |  |  |
| --- | --- | --- |
| 1. Gênero (n=404). |  | n (%) |
|  | Feminino | 299 (74) |
|  | Masculino | 105 (26) |
| 2. Idade (anos) (n=404). |  | média (±DP) |
|  |  | 38 (10,4) |
| 3. Região Administrativa de Moradia (n=389). |  | n (%) |
|  | Águas Claras (RA XX) | 60 (15,4) |
|  | Arniqueira (RA XXXIII) | 5 (1,3) |
|  | Brazlândia (RA IV) | 1 (0,3) |
|  | Ceilândia (RA IX) | 7 (1,8) |
|  | Cruzeiro (RA XI) | 4 (1,0) |
|  | Fercal (RA XXXI) | 1 (0,3) |
|  | Gama (RA II) | 16 (4,1) |
|  | Guará (RA X) | 32 (8,2) |
|  | Jardim Botânico (RA XXVII) | 14 (3,6) |
|  | Lago Norte (RA XVIII) | 13 (3,3) |
|  | Lago Sul (RA XVI) | 13 (3,3) |
|  | Núcleo Bandeirante (RA VIII) | 4 (1,0) |
|  | Park Way (RA XXIV) | 6 (1,5) |
|  | Planaltina (RA VI) | 5 (1,3) |
|  | Plano Piloto (RA I) | 111 (28,5) |
|  | Recanto das Emas (XV) | 1 (0,3) |
|  | Riacho Fundo (RA XVII) | 3 (0,8) |
|  | Riacho Fundo II (RA XXI) | 2 (0,5) |
|  | Samambaia (RA XII) | 10 (2,6) |
|  | Santa Maria (RA XIII) | 2 (0,5) |
|  | São Sebastião (RA XIV) | 2 (0,5) |
|  | Sobradinho (RA V) | 15 (3,9) |
|  | Sobradinho II (RA XXVI) | 3 (0,8) |
|  | Sudoeste/Octogonal (RA XXII) | 25 (6,4) |
|  | Taguatinga (RA III) | 20 (5,1) |
|  | Varjão (RA XXIII) | 1 (0,3) |
|  | Vicente Pires (RA XXX) | 13 (3,3) |

*DP = Desvio Padrão.

**Tabela 2 -** Formação e experiência dos cirurgiões-dentistas do Distrito Federal em 2023 (n=404).

| Variáveis |  |  |
| --- | --- | --- |
| 1. Tempo de experiência (anos) |  | média (±DP) |
|  |  | 12 (10,9) |
| 2. Atuação profissional |  | n (%) |
|  | Docência de graduação | 25 (6,2) |
|  | Docência de pós-graduação | 2 (0,5) |
|  | Clínica do serviço público | 80 (19,8) |
|  | Clínica própria do serviço privado | 136 (33,7) |
|  | Clínica compartilhada do serviço privado | 133 (32,9) |
|  | Clínica Filantrópica | 15 (3,7) |
|  | Pesquisa | 5 (1,2) |
|  | Perícia | 1 (0,2) |
|  | Hospital privado | 1 (0,2) |
|  | Hospital das Forças | 1 (0,2) |
|  | Gestão | 2 (0,2) |
|  | Não atuo como CD | 3 (0,7) |
| 3. Instituição de graduação |  | n (%) |
|  | Pública | 173 (42,8) |
|  | Privada sem bolsa/auxílios | 160 (39,6) |
|  | Privada com bolsa/auxílios | 71 (17,6) |
| 4. Maior titulação |  | n (%) |
|  | Graduação | 58 (14,4) |
|  | Especialização | 247 (61,1) |
|  | Mestrado | 70 (17,3) |
|  | Doutorado | 26 (6,4) |
|  | Pós-doutorado | 3 (0,7) |
|  |  |  |
| 5. Especialidade |  | n (%) |
|  | Não possuo | 57 (8,9) |
|  | Acupuntura | 6 (0,9) |
|  | Cirurgia Bucomaxilofacial | 11 (1,7) |
|  | Dentística | 56 (8,8) |
|  | Disfunção Temporomandibular e Dor Orofacial | 6 (0,9) |
|  | Endodontia | 51 (8,0) |
|  | Estomatologia | 9 (1,4) |
|  | Harmonização Orofacial | 29 (4,6) |
|  | Implantodontia | 70 (11,0) |
|  | Odontogeriatria | 4 (0,6) |
|  | Odontologia do Esporte | 1 (0,2) |
|  | Odontologia do Trabalho | 6 (0,9) |
| Continua |  |  |
| **Tabela 2 -** Formação e experiência dos cirurgiões-dentistas do Distrito Federal em 2023 (n=404). | | |
| 5. Especialidade |  | n (%) |
|  | Odontologia em Saúde Coletiva | 39 (6,1) |
|  | Odontologia Legal | 1 (0,2) |
|  | Odontologia para Pacientes Especiais | 2 (0,3) |
|  | Odontopediatria | 79 (12,4) |
|  | Ortodontia | 58 (9,1) |
|  | Ortopedia Facial dos Maxilares | 8 (1,3) |
|  | Patologia Oral e Maxilofacial | 5 (0,8) |
|  | Periodontia | 31 (4,9) |
|  | Prótese Bucomaxilofacial | 1 (0,2) |
|  | Prótese Dentária | 65 (10,2) |
|  | Radiologia Odontológica e Imaginologia | 11 (1,7) |
|  | Possuo pós-graduação em outra área | 31 (4,9) |

*DP = Desvio Padrão.

**Tabela 3 -** Formação dos cirurgiões-dentistas do Distrito Federal em OMI em 2023 (n=404).

| Variáveis |  |  |
| --- | --- | --- |
| 1. Já recebeu treinamento em OMI |  | n (%) |
|  | Sim | 290 (71,8) |
|  | Não | 114 (28,2) |
| 2. Onde se deu a maior parte do treinamento em OMI |  | n (%) |
|  | Graduação | 121 (41,7) |
|  | Especialização | 44 (15,2) |
|  | Mestrado | 20 (6,9) |
|  | Doutorado | 4 (1,4) |
|  | Cursos livres - exemplos: aperfeiçoamento, atualização, extensão. | 101 (34,8) |
| 3. Qual a fonte em que mais busca informação em OMI |  | n (%) |
|  | Não busco informações | 66 (16,3) |
|  | Cursos ou palestras | 134 (33,2) |
|  | Páginas da internet não científicas | 8 (2,0) |
|  | Mídias sociais (ex. Facebook, Instagram, WhatsApp, Telegram) | 27 (6,7) |
|  | Artigos científicos em português | 35 (8,7) |
|  | Artigos científicos em inglês e outros idiomas | 95 (23,5) |
|  | Livros ou guias de prática clínica | 36 (8,9) |
|  | Fontes variadas | 3 (0,7) |

Sobre a presença de barreiras para obter informação em OMI, a maioria dos profissionais não consideraram as situações apresentadas como obstáculos, já que as porcentagens das avaliações mais desfavoráveis, 4 e 5, foram as menores, conforme pode ser visto na primeira parte da Tabela 4.

As Tabelas 5, 6 e 7 trazem os resultados sobre os conhecimentos, habilidades e atitudes em Odontologia de Mínima Intervenção. Os itens de conhecimento tiveram maior quantidade de avaliações favoráveis, com destaque para a área de Reconhecimento, Redução e Remineralização. O item de conhecimento com maior porcentagem de avaliações positivas foi sobre a identificação de fatores de risco da doença cárie, somando 86,1% dos profissionais. O item com menor porcentagem de avaliações positivas foi o de conhecimento sobre a OMI para o manejo da cárie dentária, com 59,9%.

**Tabela 4 -** Barreiras dos cirurgiões-dentistas do Distrito Federal para a Odontologia de Mínima Intervenção em 2023 (n=404).

| Variáveis |  |  |  |  |  |  |
| --- | --- | --- | --- | --- | --- | --- |
|  |  |  |  |  |  |  |
| Dificuldades para o obter informação | n (%) |  |  |  |  | % |
|  | 1. Nenhuma | 2. | 3. | 4. | 5.  Muita | Soma  4- 5 |
| Acesso à tecnologia para obter informação | 294 (72,8) | 54 (13,4) | 39 (9,7) | 12 (3,0) | 5 (1,2) | 4,2% |
| Disponibilidade de fontes de informações confiáveis | 147 (36,4) | 112 (27,7) | 93 (23,0) | 42 (10,4) | 10 (2,5) | 12,9% |
| Finanças para obter acesso a fontes de informação | 156 (38,6) | 104 (25,7) | 91 (22,7) | 40 (9,9) | 13 (3,2) | 13,1% |
|  |  |  |  |  |  |  |
|  |  |  |  |  |  |  |
| Continua |  |  |  |  |  |  |
| **Tabela 4 -** Barreiras dos cirurgiões-dentistas do Distrito Federal para a Odontologia de Mínima Intervenção em 2023 (n=404). | | | | | | |
| Dificuldades para o obter informação | n (%) |  |  |  |  | % |
|  | 1. Nenhuma | 2. | 3. | 4. | 5.  Muita | Soma  4- 5 |
| Idiomas estrangeiros das fontes de informação | 163 (40,3) | 100 (24,8) | 81 (20,0) | 37 (9,2) | 23 (5,7) | 14,9% |
| Tempo e organização para atualizar o conhecimento | 54 (13,4) | 107 (26,5) | 143 (35,4) | 59 (14,6) | 41 (10,1) | 24,7% |
| Dificuldades para prática | n (%) |  |  |  |  | % |
| Confiança na eficácia da mínima intervenção | 199 (49,3) | 71 (17,6) | 63 (15,6) | 40 (9,9) | 31 (7,7) | 11,6% |
| Interesse em praticar a OMI | 222 (55,0) | 70 (17,3) | 53 (13,1) | 31 (7,7) | 28 (6,9) | 14,6% |
| Dificuldades para prática | n (%) |  |  |  |  | % |
| Habilidades práticas com a mínima intervenção | 175 (43,3) | 100 (24,8) | 67 (16,6) | 46 (11,4) | 16 (4,0) | 15,4% |
| Confiança dos pacientes nos procedimentos de mínima intervenção | 97 (24,0) | 102 (25,2) | 94 (23,3) | 63 (15,6) | 48 (11,9) | 27,5% |
| Valorização dos pacientes em remunerar procedimentos de mínima intervenção | 89 (22,0) | 65 (16,1) | 109 (27,0) | 59 (14,6) | 82 (20,3) | 34,9% |
| Remuneração adequada dos procedimentos de mínima intervenção | 71 (17,6) | 62 (15,3) | 116 (28,7) | 72 (17,8) | 83 (20,5) | 38,3% |

Sobre as habilidades em OMI (Tabela 6), os itens também tiveram maior porcentagem de avaliações favoráveis pelos profissionais. Os itens com a maior e menor porcentagem de avaliações positivas foram na área de “Reconhecimento, Redução e Remineralização”. O maior, sobre a redução da frequência de consumo de alimentos fontes de açúcares, como um fator importante para controlar o início da doença, com 90,6%. E o item com menor porcentagem de avaliações positivas, porém, ainda bom, foi sobre a exposição a fontes de flúor como um bom indicador de proteção para a doença cárie, com 73,5%.

Em atitudes em OMI, a maioria dos itens tiveram maior porcentagem de avaliações favoráveis, as maiores em Reconhecimento, Redução e Remineralização e OMI Geral. O item de atitude com maior porcentagem de avaliações positivas foi sobre a frequência de orientação de sobre a escovação dentária diária com pasta fluoretada para os pacientes de alto risco à doença cárie, com 95,6%. O item com menor quantidade de avaliações positivas foi sobre a frequência em que é deixada dentina cariada amolecida nas paredes de fundo da cavidade para evitar exposição pulpar, em restaurações de lesões de cárie profundas de dentes vitais, com 38,6%, classificado como razoável. Todos os resultados estão descritos na Tabela 7.

No que diz respeito às barreiras para praticar a OMI, a maioria dos itens não foram considerados como obstáculos pelos profissionais, já que as porcentagens das avaliações mais desfavoráveis, 4 e 5, foram as menores, conforme pode ser visto na segunda parte da Tabela 4. Excetua-se o item sobre remuneração adequada dos procedimentos de mínima intervenção, que, apesar de certa equivalência nas porcentagens, teve maior percentual de profissionais que o avaliaram de forma desfavorável (38,3%), apontando esse um obstáculo para a prática da mínima intervenção.

**Tabela 5 -** Conhecimentos dos cirurgiões-dentistas do Distrito Federal para a Odontologia de Mínima Intervenção em 2023 (n=404).

| Variáveis |  |  |  |  |  |  |
| --- | --- | --- | --- | --- | --- | --- |
| *Reconhecimento Redução e Remineralização* | n (%) |  |  |  |  | %  Classificação |
| O quanto você sabe? | 1. Nada | 2. | 3. | 4. | 5. Muito | Soma  4 - 5 |
| .... sobre a identificação dos fatores de risco da doença cárie? | 1 (0,2) | 6 (1,5) | 49 (12,1) | 160 (39,6) | 188 (46,5) | 86,1%  Excelente |
| ... a respeito do controle do início da doença cárie? | 1 (0,2) | 14 (3,5) | 53 (13,1) | 164 (40,6) | 172 (42,6) | 83,2%  Excelente |
| ... a respeito do controle do avanço da doença cárie? | 0 (0,0) | 8 (2,0) | 62 (15,3) | 185 (45,8) | 149 (36,9) | 82,7%  Excelente |
| *Restaurações minimamente invasivas e Reparo de restaurações* | n (%) |  |  |  |  | %  Classificação |
| ... sobre os procedimentos restauradores minimamente invasivos? | 6 (1,5) | 19 (4,7) | 74 (18,3) | 181 (44,8) | 124 (30,7) | 75,5%  Excelente |
| *OMI Geral* | n (%) |  |  |  |  | %  Classificação |
| ... sobre a Odontologia de Mínima Intervenção para o manejo da cárie dentária? | 12 (3,0) | 40 (9,9) | 110 (27,2) | 157 (38,9) | 85 (21,0) | 59,9%  Bom |

**Tabela 6 -** Habilidades dos cirurgiões-dentistas do Distrito Federal para a Odontologia de Mínima Intervenção em 2023 (n=404).

| Variáveis |  |  |  |  |  |  |
| --- | --- | --- | --- | --- | --- | --- |
| *Reconhecimento, Redução e Remineralização* | n (%) |  |  |  |  | %  Classificação |
| O quanto você concorda com a seguinte afirmação: | 1.  Discordo Totalmente | 2. | 3. | 4. | 5.  Concordo totalmente | Soma 4 - 5 |
| "Em pacientes de alto risco para a doença cárie, a mudança de hábitos, tal como a redução da frequência de consumo de alimentos fontes de açúcares, é um fator importante para controlar o início da doença". | 1 (0,2) | 11 (2,7) | 26 (6,4) | 96 (23,8) | 270 (66,8) | 90,6%  Excelente |
| "A exposição a fontes de flúor é um bom indicador de proteção para a doença cárie". | 9 (2,2) | 18 (4,5) | 80 (19,8) | 114 (28,2) | 183 (45,3) | 73,5%  Bom |
| Continua |  |  |  |  |  |  |
|  | | | | | | |
|  | | | | | | |
|  | | | | | | |
| **Tabela 6 -** Habilidades dos cirurgiões-dentistas do Distrito Federal para a Odontologia de Mínima Intervenção em 2023 (n=404). | | | | | | |
| *OMI Geral* | n (%) |  |  |  |  | %  Classificação |
| “O objetivo da Odontologia de Mínima Intervenção é manter os dentes saudáveis ​​e funcionais por toda a vida, e envolve a implementação de estratégias importantes para manter os dentes livres de lesões de cárie. Essas estratégias são a detecção precoce  de cárie e avaliação de risco; remineralização do esmalte e dentina desmineralizados;  medidas ótimas de prevenção de cárie; intervenções operatórias minimamente invasivas e reparo ao invés de substituição de restaurações". | 2 (0,5) | 5 (1,2) | 32 (7,9) | 82 (20,3) | 283 (70,0) | 90,3%  Excelente |
| *Restaurações minimamente invasivas e Reparo de restaurações* | n (%) |  |  |  |  | %  Classificação |
| Continua |  |  |  |  |  |  |
| **Tabela 6 -** Habilidades dos cirurgiões-dentistas do Distrito Federal para a Odontologia de Mínima Intervenção em 2023 (n=404). | | | | | | |
| *Restaurações minimamente invasivas e Reparo de restaurações* | n (%) |  |  |  |  | %  Classificação |
| "A remoção de dentina cariada diferencia-se de acordo com a profundidade das lesões de cárie, devendo ser mais conservadora em lesões profundas, a fim de evitar exposição pulpar em dentes vitais". | 22 (5,4) | 21 (5,2) | 42 (10,4) | 88 (21,8) | 231 (57,2) | 79,0%  Excelente |
| "Em restaurações defeituosas, deve-se considerar o reparo antes de se optar pela remoção da restauração e a realização de uma nova". | 24 (5,9) | 16 (4,0) | 59 (14,6) | 72 (17,8) | 233 (57,7) | 75,5%  Bom |

**Tabela 7.** Atitudes dos cirurgiões-dentistas do Distrito Federal para a Odontologia de Mínima Intervenção em 2023 (n=404).

| Variáveis |  |  |  |  |  |  |
| --- | --- | --- | --- | --- | --- | --- |
| *Reconhecimento, Redução e Remineralização* | n (%) |  |  |  |  | (%)  Classificação |
|  | 1. Nunca | 2. | 3. | 4. | 5. Sempre | Soma 4 e 5 |
| Para os pacientes de alto risco à doença cárie, com que frequência você orienta sobre a escovação dentária diária com pasta fluoretada? | 5 (1,2) | 2 (0,5) | 11 (2,7) | 29 (7,2) | 357 (88,4) | 95,6%  Excelente |
| Para os pacientes de alto risco para a doença cárie, com que frequência você orienta sobre a redução do consumo de alimentos fontes de açúcares? | 12 (3,0) | 16 (4,0) | 35 (8,7) | 86 (21,3) | 255 (63,1) | 84,4%  Excelente |
| Com que frequência você avalia o risco à doença cárie dos seus pacientes? | 19 (4,7) | 39 (9,7) | 85 (21,0) | 103 (25,5) | 158 (39,1) | 64,6%  Bom |
| *OMI Geral* |  |  |  |  |  |  |
| Com que frequência você aplica a Odontologia de Mínima Intervenção no manejo da cárie dentária na sua prática clínica diária?  Continua | 22 (5,4) | 29 (7,2) | 63 (15,6) | 119 (29,5) | 171 (42,3) | 71,8%  Bom |
| **Tabela 7** - Atitudes dos cirurgiões-dentistas do Distrito Federal para a Odontologia de Mínima Intervenção em 2023 (n=404). | | | | | | |
| *Restaurações minimamente invasivas e Reparo de restaurações* |  |  |  |  |  |  |
| Na abordagem de restaurações defeituosas, com que frequência você realiza o reparo em vez de realizar a troca total da restauração? | 18 (4,5) | 32 (7,9) | 103 (25,5) | 128 (31,7) | 123 (30,4) | 62,1%  Bom |
| Em restaurações de lesões de cárie profundas de dentes vitais, com que frequência você deixa dentina cariada amolecida nas paredes de fundo da cavidade para evitar exposição pulpar? | 98 (24,3) | 68 (16,8) | 82 (20,3) | 87 (21,5) | 69 (17,1) | 38,6%  Razoável |

**4.3.2 Resultado da regressão linear múltipla com as variáveis dependentes**

A regressão linear múltipla resultou em um modelo estatisticamente significativo para associação entre as variáveis dependentes e as variáveis preditivas, conforme pode ser visto nas tabelas 2, 3 e 5 do Apêndice 11.

Espera-se que haja uma média maior de conhecimento em OMI em 0,56 pontos entre os CDs do DF que estudaram em instituição pública quando comparados aos que estudaram em instituição particular (IC 95% 0,03; 1,09; p=0,03). Além disso, espera-se que CDs que atuem na docência/pesquisa apresentem em média 1,24 pontos a mais de conhecimento em OMI (IC 95% 0,27; 2,21; p=0,01) quando comparados aos que trabalham em clínicas particulares, públicas e no setor administrativo. Ainda, CDs que buscam por informação e os que receberam treinamento em OMI tendem a apresentar resultados de conhecimento em OMI, em média, maiores (2,35; IC 95% 1,56; 3,14; p<0,001 e 2,95; IC 95% 2,31; 3,59; p<0,001; respectivamente). Os resultados indicam que este modelo pode explicar 36% da variabilidade do conhecimento em OMI.

Espera-se que haja uma maior média na pontuação de habilidades e atitudes em OMI entre os CDs do DF do gênero feminino (1,22; IC 95% 0,07; 2,37; p=0,03) assim como entre os que estudaram em instituição pública (2,17; IC 95% 1,15; 3,18; p<0,001). Além disso, CDs que buscam por informação e os que receberam treinamento em OMI tendem a apresentar resultados de habilidades e atitudes em OMI, em média, maiores (3,06; IC 95% 1,55; 4,56; p<0,001 e 3,40; IC 95% 2,18; 4,62; p<0,001; respectivamente). Os resultados indicam que este modelo pode explicar 22% da variabilidade de habilidades e atitudes em OMI.

Espera-se que os CDs do DF que estudaram em instituição pública tenham em média menos barreiras para o conhecimento na pontuação do questionário quando comparados aos que estudaram em instituição particular (-0,88 IC 95% -1,61; -0,15; p=0,01). O mesmo acontece para os CDs que tem pós-graduação como maior titulação, atuam na docência/pesquisa ou já receberam treinamento em OMI (-1,11 IC 95% -2,22; -0,003; p=0,04; -2,56 IC 95% -3,90; -1,23; p<0,001 e -1,52 IC 95% -2,35; -0,68; p<0,001 respectivamente). Os resultados indicam que este modelo pode explicar 8% da variabilidade das barreiras para o conhecimento em OMI.

Quanto mais jovem o CD, espera-se maiores pontuações em média em barreiras para a prática (-0,07 IC 95% -0,14; 0,18; p=0,01). Os CDs do DF que já receberam treinamento em OMI tem em média menos barreiras para a prática na pontuação do questionário (-1,84 IC 95% -3,25; -0,44; p=0,01). Os resultados indicam que este modelo pode explicar 3% da variabilidade das barreiras para a prática em OMI.

4.4 DISCUSSÃO

Esse estudo avaliou os conhecimentos, habilidades, atitudes e barreiras dos cirurgiões-dentistas (CDs) sobre a Odontologia de Mínima Intervenção (OMI), correlacionando-os com o perfil e formação desses profissionais. Em geral, para os conhecimentos, habilidades e atitudes, os CDs apresentaram um maior percentual de avaliações favoráveis, o que rendeu percentuais de competências de excelentes a bons, sendo apenas um item razoável em atitudes. Com relação as barreiras para se atualizar e praticar a OMI, em geral, as avaliações não apontaram para a presença de dificuldades entre os profissionais, com exceção de um item de prática.

Sobre a avaliação de conhecimentos, o item sobre o quanto os profissionais sabem sobre a Odontologia de Mínima Intervenção para o manejo da cárie dentária foi reproduzido de estudos anteriores. Em uma revisão sistemática realizada por esta equipe, o resultado da Metanálise desse item demonstrou um percentual de 41,25% [33,57 – 49,16](5), menor do que o percentual encontrado nesse estudo.(5)

Já a avaliação geral de conhecimentos demonstrou-se favorável, assim como em estudos anteriores em outros países e com itens diferentes dos avaliados nesse estudo, nas mesmas áreas temáticas de Reconhecimento, Redução e Remineralização; Restaurações minimamente invasivas e Reparo de restaurações. (19-24) Em contraste, um estudo brasileiro que avaliou conceitos de OMI, encontrou conhecimentos de razoáveis a insuficientes.(6)

A avaliação dos profissionais sobre as habilidades e atitudes foram favoráveis em geral, assim como alguns estudos prévios em outros países, que estudaram atitudes e práticas nas áreas de Reconhecimento, Redução e Remineralização; Restaurações minimamente invasivas e Reparo de restaurações. (8, 19, 23, 25)

Alguns itens foram replicados de estudos anteriores, como foi o caso de "em restaurações defeituosas, deve-se considerar o reparo antes de se optar pela remoção da restauração e a realização de uma nova", nesse estudo com 62,1% de avaliações favoráveis, e no resultado da Metanálise realizada previamente com o mesmo item, de 25,44% [IC 14,84 - 37,73].(5) Já o item sobre atitudes em avaliação do risco de cárie dos pacientes apresentou resultados similares, 64,6% nesse estudo e 68,45% [IC 44,68 - 62,96] na Metanálise.(5)

O item com menor avaliação em todo o estudo foi em atitudes, sobre deixar dentina cariada amolecida nas paredes de fundo da cavidade para evitar exposição pulpar em lesões de cárie profundas, com 38,6% de avaliações favoráveis. Cabe ressaltar que 41,1% dos profissionais apontaram para uma baixa frequência de realização dessa técnica (pontuação 1 ou 2 da escala), sendo esse um item conflituoso entre os profissionais. Uma revisão sistemática prévia que investigou a proporção de dentistas que utiliza a remoção seletiva de tecido cariado, encontrou que quase metade dos dentistas investigados nos estudos escolheram estratégias mais invasivas para abordar lesões de cárie profundas em dentes permanentes, em lugar das estratégias baseadas em evidência científica, e que essa proporção tende a diminuir com o passar dos anos.(26)

Na avaliação das barreiras para o conhecimento e prática da OMI, apenas o item “remuneração adequada dos procedimentos de mínima intervenção” apresentou maior percentual de profissionais que apontaram esse item como um obstáculo para a prática. Em outros países, como Nova Zelândia, Estados Unidos, Alemanha e Inglaterra parece haver maior valorização por procedimentos restauradores do que procedimentos menos invasivos nos sistemas de saúde bucal.(27, 28) Por não termos ciência de outro estudo brasileiro que aborde esse fato, mais estudos que avaliem essa temática mais especificamente, sobretudo de forma qualitativa, são necessários.

O resultado da regressão linear múltipla demonstrou que, em média, os CDs que buscam por informação em OMI e que receberam treinamento na área apresentaram maiores pontuações de conhecimentos, habilidades e atitudes. Outros estudos também confirmaram uma relação significativa entre treinamento prévio em OMI e conhecimento (21), treinamento prévio e prática. (23) Já outro estudo não encontrou relação significativa. (20)

Outro achado da regressão foi a associação dos CDs do sexo feminino, e os CDs que estudaram em instituição pública, com uma maior média nas pontuações em habilidades e atitudes em OMI. De forma semelhante, um estudo encontrou uma associação positiva entre o sexo feminino e concordância com a evidência em OMI.(8)

Esse estudo teve algumas limitações metodológicas. Quanto ao método de seleção da amostra, optou-se por um método de amostragem não probabilístico, tendo em vista a impossibilidade de realizar um método de randomização da população-alvo, o que pode gerar um viés de seleção, que pode ter sido minimizado com a seleção cautelosa e ampla da primeira etapa de participantes. Outra limitação do estudo refere-se ao fato de se tratar de um estudo transversal, em que os dados estudados se referem a um recorte da realidade dos participantes estudados, podendo não refletir a realidade plena destes, podendo ocorrer viés de memória. Portanto, os resultados do presente estudo devem ser interpretados com cautela. Além disso, por se tratar de uma pesquisa com uma população local, os resultados e interpretações advindos dela não podem ser extrapolados para outras populações.

Por ser um estudo com utilização de um questionário com evidência de validação, além de amplitude no assunto e um tamanho de amostra adequado, entende-se que essa pesquisa trouxe ganhos no conhecimento da das competências e dificuldades sobre a Odontologia de Mínima Intervenção, Sugere-se a partir deste trabalho a criação de outros de forma a se ampliar o entendimento das potencialidades e dificuldades dos profissionais sobre a mínima intervenção, e aprofundar o detalhamento dessas questões com uma abordagem qualitativa.

**REFERÊNCIAS**

1. Dawett B, Atkins B, Banerjee A. A guide to building 'MI' oral healthcare practice. Br Dent J. 2017;223(3):223-7.

2. Walsh LJ, Brostek AM. Minimum intervention dentistry principles and objectives. Aust Dent J. 2013;58:3-16.

3. Innes NPT, Schwendicke F. Restorative Thresholds for Carious Lesions: Systematic Review and Meta-analysis. J Dent Res. 2017;96(5):501-8.

4. Ricketts D, T L, NP I, E K, JE C. Operative caries management in adults and children. The Cochrane database of systematic reviews. 2013;28(3):CD003808.

5. Moura RC, Santos PS, Matias PMDS, Vitali FC, Hilgert LA, Cardoso M, et al. Knowledge, attitudes, and practice of dentists on Minimal Intervention Dentistry: A systematic review and meta-analysis. Journal of dentistry. 2023;132:104484.

6. Katz CRT, De Andrade MDRB, Lira SS, Ramos Vieira EL, Heimer MV. The concepts of minimally invasive dentistry and its impact on clinical practice: A survey with a group of Brazilian professionals. Int Dent J. 2013;63(2):85-90.

7. von Elm E, Altman DG, Egger M, Pocock SJ, Gøtzsche PC, Vandenbroucke JP. The Strengthening the Reporting of Observational Studies in Epidemiology (STROBE) statement: guidelines for reporting observational studies. Journal of clinical epidemiology. 2008;61(4):344-9.

8. Kakudate N, Yokoyama Y, Sumida F, Matsumoto Y, Yamazaki H, Touge T, et al. Evidence-practice gap in minimal intervention dentistry: Findings from a dental practice-based research network. J Dent. 2020;102:103469.

9. Frencken JE, Peters MC, Manton DJ, Leal SC, Gordan VV, Eden E. Minimal intervention dentistry for managing dental caries–a review: report of a FDI task group. **Int Dent J**. 2012;62(5):223-43.

10. Innes N, Frencken J, Bjørndal L, Maltz M, Manton D, Ricketts D, et al. Managing Carious Lesions: Consensus Recommendations on Terminology. Advances in dental research. 2016;28(2):49-57.

11. Zarifian P. Objetivo competência: por uma nova lógica; tradução.2001. 197 p.

12. Norton WE, Funkhouser E, Makhija SK, Gordan VV, Bader JD, Rindal DB, et al. Concordance between clinical practice and published evidence: findings from The National Dental Practice-Based Research Network. J Am Dent Assoc. 2014;145(1):22-31.

13. Snijders TAB. Estimation On the Basis of Snowball Samples: How To Weight? <http://dxdoiorg/101177/075910639203600104>. 1992.

14. Goodman LA. Snowball Sampling. <https://doiorg/101214/aoms/1177705148>. 1961.

15. Kirkwood BR, Sterne JA. Essential medical statistics: John Wiley & Sons; 2010. 512 p.

16. Tewari N, Sultan F, Mathur VP, Rahul M, Goel S, Bansal K, et al. Global status of knowledge for prevention and emergency management of traumatic dental injuries in dental professionals: Systematic review and meta-analysis. Dent Traumatol. 2021;37(2):161-76.

17. Field A. Descobrindo a estatística usando o SPSS-5: Penso Editora; 2009. 688 p.

18. Williams MN, Grajales CAG, Kurkiewicz D. Assumptions of multiple regression: Correcting two misconceptions. Practical Assessment, Research, and Evaluation. 2013;18(1):11.

19. Kumar S, Mala N, Rana KS, Namazi N, Rela R, Kumar K. Cognizance and use of minimally invasive dentistry approach by general dentists: An overlooked companion. J Pharm Bioallied Sci. 2021;13(5):199.

20. Natarajan K, Prabakar J. Knowledge, attitude, and practice on minimally invasive dentistry among dental professionals in Chennai. Drug Invent Today. 2019;11(8):1768-72.

21. Oliveira DC, Warren JJ, Levy SM, Kolker J, Qian F, Carey C. Acceptance of minimally invasive dentistry among US dentists in public health practices. Oral Health Prev Dent. 2016;14(6):501-8.

22. Rayapudi J, Usha C. Knowledge, attitude and skills of dental practitioners of Puducherry on minimally invasive dentistry concepts: A questionnaire survey. J Conserv Dent. 2018;21(3):257-62.

23. Shah AH, Sheddi FM, Alharqan MS, Khawja SG, Vohra FM, Akram Z, et al. Knowledge and attitude among general dental practitioners towards minimally invasive dentistry in Riyadh and AlKharj. J Clin Diagn Res. 2016;10(7):90-4.

24. Suma G, Salman Y, Devadoss E. Knowledge, Attitude, Behavior, and Practice toward Minimal Intervention Dentistry among Dental Professionals in Bengaluru City, India. Journal of Health Sciences & Research,. 2017;8(1):20-4.

25. Mirsiaghi F, Leung A, Fine P, Blizard R, Louca C. An investigation of general dental practitioners' understanding and perceptions of minimally invasive dentistry. British Dental Journal. 2018;225(5):420-4.

26. Cheng L, Zhang L, Yue L, Ling J, Fan M, Yang D, et al. Expert consensus on dental caries management. International journal of oral science. 2022;14(1):17.

27. Schwendicke F, Foster Page L, Smith L, Fontana M, Thomson W, Baker SR. To fill or not to fill: a qualitative cross-country study on dentists' decisions in managing non-cavitated proximal caries lesions. Implementation science : IS. 2018;13(1):54.

28. Banerjee A. MI'opia or 20/20 vision? Br Dent J. 2013;214(3):101-5.

# 5. DISCUSSÃO GERAL E CONSIDERAÇÕES FINAIS

## 5.1 DISCUSSÃO GERAL

Essa dissertação objetivou avaliar as competências e barreiras dos cirurgiões-dentistas acerca da Odontologia de Mínima Intervenção (OMI). Foram realizados: uma Revisão Sistemática (RS); a construção e análise das propriedades psicométricas de um instrumento para avaliar competências dos cirurgiões-dentistas em OMI; um estudo transversal com os cirurgiões-dentistas do Distrito Federal. Verificou-se por meio da Revisão Sistemática que os cirurgiões-dentistas ao redor do mundo apresentaram conhecimento adequados e atitudes e práticas em OMI com necessidade de melhoria. As propriedades psiciométricas do questionário se mostraram adequadas. O estudo transversal demonstrou conhecimentos, habilidades e atitudes dos cirurgiões-dentistas adequadas em geral, com exceção de atitudes em remoção seletiva de dentina cariada; para as barreiras, apenas a remuneração em mínima intervenção apresentou-se como uma barreira para a prática.

Tanto na revisão quanto no estudo transversal, observou-se que as competências, sejam elas conhecimentos, habilidades, atitudes ou práticas apresentaram um maior percentual de avaliações favoráveis nas áreas de Reconhecimento, Redução e Remineralização da OMI. Ressalta-se que a avaliação de risco de forma individual, a detecção precoce e a remineralização de lesões iniciais de cárie fazem parte de um cuidado baseado na prevenção da doença e seu controle, e essa filosofia preventiva é a base da mínima intervenção.(1, 2)

O reconhecimento dos fatores de risco para a doença cárie, como o consumo de carboidratos refinados, e do controle da doença, como a avaliação da dieta e aconselhamento dietético, tiveram destaque nos estudos realizados. Apesar do já provado papel do açúcar como fator direto no desenvolvimento da cárie(3-6), apenas recentemente houve o reconhecimento da “epidemia” do açúcar e da necessidade de abordar sua redução frente as doenças crônicas ligadas ao seu consumo.(7) Ainda, há necessidade de maiores evidências de como elaborar meios de favorecer a mudança de comportamento dos indivíduos.(8, 9) As estratégias de saúde pública que intermedeiam sua redução de acesso, por exemplo, tem sido um importante foco,(10) e a odontologia tem se envolvido junto a essas medidas(7).

O papel do flúor na proteção a cárie bem como na remineralização de lesões cariosas, foi outro destaque nos estudos realizados, seja na recomendação do uso diário de pasta fluoretada, seja na aplicação profissional nas formas tópicas. O papel do flúor na prevenção da cárie bem como na paralisação de lesões cariosas já é bem estabelecido e amparado por evidência científica consistente(11-13), e a prevalência e taxa de progressão da cárie dentária diminuiu drasticamente devido ao seu uso disseminado nas populações.(14) Ressalta-se que o uso de fluoretos deve ser individualizado para cada paciente com base em suas características como idade, condições físicas, conhecimentos e atitudes, e por esse motivo também a avaliação de risco do paciente é tão importante.(2)

O reparo de restaurações defeituosas em lugar da troca da restauração teve resultados distintos, sendo bom no estudo transversal e insuficiente na revisão. O ato de reparar restaurações com defeito em lugar de realizar sua troca é um dos elementos-chave da mínima intervenção.(15) Há uma forte recomendação de realizar o retratamento de restaurações por meio do reparo com selagem, condicionamento ou polimento sempre que for possível, deixando a substituição para o último caso.(16) Apesar disso, a troca de restaurações de forma subjetiva e desnecessária ainda parece ser a primeira opção dos dentistas.(17) Reconhece-se que a decisão de reparar, trocar ou fazer a manutenção da restauração é desafiadora, já que não há um critério único e objetivo que delimite uma restauração como insatisfatória(18, 19). Ainda assim, a evidência clínica sugere que o tratamento conservador ainda é o melhor para restaurações defeituosas(19)

A remoção seletiva de dentina cariada é outro tópico que merece destaque, dessa vez negativo. No estudo transversal, uma maior porcentagem de profissionais não realiza com frequência a remoção seletiva em lesões de cárie com risco de exposição pulpar e na revisão este item apareceu em poucos estudos, com resultados medianos. Há forte recomendação de realizar a remoção seletiva de dentina cariada até a dentina amolecida(16), com evidência acumulada de ensaios clínicos, confirmando ser essa uma abordagem que reduz riscos quando comparada a remoção não seletiva de dentina dura ou remoção completa.(10) Ainda assim, cerca de metade dos dentistas ao redor do mundo ainda não adota essa prática.(20)

Quanto as barreiras apresentadas pelos cirurgiões-dentistas, a com maior destaque no estudo transversal foi sobre a remuneração inadequada para praticar a OMI, e essa dificuldade não foi demonstrada na revisão realizada. A questão financeira em mínima intervenção parece ser um paradigma. De um lado, ao redor do mundo, há problemas com a valorização dos procedimentos de mínima intervenção, com favorecimento aos procedimentos restauradores.(21, 22) Por outro lado, a mínima intervenção traz grande benefício ao paciente,(7) possui baixo custo e custo-efetividade comprovados.(23) Recomenda-se a partir do presente estudo, mais investigação sobre a remuneração de procedimentos de mínima intervenção, como essa barreira tem realmente afetado a prática dos profissionais, e verificar se podem existir outros fatores explicativos para isso, especialmente no contexto brasileiro. Estudos qualitativos podem prover melhores respostas para esses questionamentos.

Acerca da validação dos instrumentos de pesquisa utilizados, na revisão verificou-se que a maioria dos estudos não relatou essa etapa de forma satisfatória, e no estudo transversal, essa etapa foi amplamente discutida, fato esse que rendeu um artigo para o tema. Verificou-se que o instrumento construído para o estudo transversal obteve bons atributos e com sugestão de boa evidência de validade. Ressalta-se que ainda não existe consenso na literatura dos parâmetros exatos para que um instrumento seja considerado válido.(24) Sugere-se, então, que o instrumento tenha a maior diversidade de evidências possível, pois gera maior confiabilidade.(24) Nesse sentido, confirma-se a necessidade de realizar mais pesquisas com o instrumento criado.

## 5.2 CONSIDERAÇÕES FINAIS

A realização desse estudo permitiu verificar o cenário de competências e barreiras dos cirurgiões-dentistas sobre a Odontologia de Mínima Intervenção a nível global, e a nível local, do Distrito Federal. Conclui-se que num cenário global uma dificuldade de realizar a aplicação do conhecimento apresentado na prática clínica. Num nível local, questões pontuais precisam ser enfrentadas sobre a prática da remoção seletiva de dentina cariada e na remuneração em mínima intervenção. Sugere-se realizar mais estudos em nível global para avaliar as competências dos profissionais e possíveis barreiras enfrentadas, e a realização de estudos qualitativos nessa temática podem ampliar ainda mais essa análise.

**REFERÊNCIAS**

1. Featherstone JDB, Doméjean S. Minimal intervention dentistry: part 1. From 'compulsive' restorative dentistry to rational therapeutic strategies. British Dental Journal. 2012;213(9):441-5.

2. Fontana M, Gonzalez-Cabezas C. Minimal intervention dentistry: part 2. Caries risk assessment in adults. British dental journal. 2012;213(9):447-51.

3. Gustafsson BE, Quensel CE, Lanke LS, Lundqvist C, Grahnen H, Bonow BE, et al. The Vipeholm dental caries study; the effect of different levels of carbohydrate intake on caries activity in 436 individuals observed for five years. Acta odontologica Scandinavica. 1954;11(3-4):232-64.

4. Harris R. Biology of the Children of Hopewood House, Bowral, Australia. 4. Observations on Dental-Caries Experience Extending over Five Years (1957-61). Journal of dental research. 1963;42:1387-99.

5. Holloway PJ, James PM, Slack GL. Dental caries among the inhabitants of Tristan da Cunha. (d). The last count. Royal Society of Health journal. 1962;82:139.

6. Scheinin A, Mäkinen KK, Ylitalo K. Turku sugar studies. V. Final report on the effect of sucrose, fructose and xylitol diets on the caries incidence in man. Acta odontologica Scandinavica. 1976;34(4):179-216.

7. Innes NPT, Chu CH, Fontana M, Lo ECM, Thomson WM, Uribe S, et al. A century of change towards prevention and minimal intervention in cariology. Journal of dental research. 2019;98(6):611-7.

8. Harris R, Gamboa A, Dailey Y, Ashcroft A. One‐to‐one dietary interventions undertaken in a dental setting to change dietary behaviour. Cochrane Database of Systematic Reviews. 2012(3):CD006540.

9. Albino J, Tiwari T. Preventing Childhood Caries: A Review of Recent Behavioral Research. Journal of dental research. 2016;95(1):35-42.

10. Innes N, Frencken J, Bjørndal L, Maltz M, Manton D, Ricketts D, et al. Managing Carious Lesions: Consensus Recommendations on Terminology. Advances in dental research. 2016;28(2):49-57.

11. Marinho V, Worthington HV, Walsh T, Clarkson JE. Fluoride varnishes for preventing dental caries in children and adolescents. The Cochrane database of systematic reviews. 2013(7):CD002279.

12. Walsh T, Worthington HV, Glenny AM, Appelbe P, Marinho VC, Shi X. Fluoride toothpastes of different concentrations for preventing dental caries in children and adolescents. Cochrane database of systematic reviews. 2010(1):CD007868.

13. Wong MC, Clarkson J, Glenny AM, Lo EC, Marinho VC, Tsang BW, et al. Cochrane reviews on the benefits/risks of fluoride toothpastes. Journal of dental research. 2011;90(5):573-9.

14. Kohn WG, Maas WR, Malvitz DM, Presson SM, Shaddix KK. Recommendations for using fluoride to prevent and control dental caries in the United States. 2001;50:1-42.

15. Tyas MJ, Anusavice KJ, Frencken JE, Mount GJ. Minimal intervention dentistry—a review* FDI Commission Project 1–97. International dental journal. 2000;50(1):1-12.

16. Schwendicke F, Frencken JE, Bjørndal L, Maltz M, Manton DJ, Ricketts D, et al. Managing Carious Lesions: Consensus Recommendations on Carious Tissue Removal. Advances in dental research. 2016;28(2):49-57.

17. Leal SC, Dame-Teixeira N, Barbosa CB, Kominami PAA, Raposo F, Nakagawa EMT, et al. Minimum intervention oral care: defining the future of caries management. Brazilian oral research. 2022;36:e135.

18. Banerjee A, Watson TF. Pickard's guide to minimally invasive operative dentistry: OUP Oxford; 2015.

19. Martins BMC, Silva EJNLD, Ferreira DMTP, Reis KR, Fidalgo TKDS. Longevity of defective direct restorations treated by minimally invasive techniques or complete replacement in permanent teeth: A systematic review. Journal of dentistry. 2018;78:22-30.

20. Schwendicke F, Göstemeyer G. Understanding dentists' management of deep carious lesions in permanent teeth: a systematic review and meta-analysis. Implementation science : IS. 2016;11(1):142.

21. Schwendicke F, Foster Page L, Smith L, Fontana M, Thomson W, Baker SR. To fill or not to fill: a qualitative cross-country study on dentists' decisions in managing non-cavitated proximal caries lesions. Implementation science : IS. 2018;13(1):54.

22. Banerjee A. MI'opia or 20/20 vision? Br Dent J. 2013;214(3):101-5.

23. Schwendicke F. Less Is More? The Long-Term Health and Cost Consequences Resulting from Minimal Invasive Caries Management. Dental clinics of North America. 2019;63(4):737-49.

24. Borsa JC, Damásio BF, Bandeira DR. Adaptação e validação de instrumentos psicológicos entre culturas: algumas considerações. Paidéia (Ribeirão Preto). 2012;22:423-32.

6. PRESS RELEASE

Esse estudo avaliou as competências e as dificuldades dos dentistas sobre a Odontologia de Mínima Intervenção (OMI), uma área da odontologia para o tratamento da cárie dentária que busca a longevidade dentária. Foram realizados uma revisão sistemática, a construção e validação de um questionário e a realização de um estudo transversal no Distrito Federal. Os resultados da revisão sistemática sugerem que os conhecimentos dos dentistas ao redor do mundo sobre tópicos da OMI são aceitáveis, e as atitudes e as práticas precisam de melhorias. Para os dentistas do Distrito Federal, verificou-se competências adequadas em OMI, com exceção para um procedimento restaurador da mínima intervenção e dificultador no que diz respeito a remuneração para a prática da OMI. Mais estudos são necessários a nível global e local para melhor entender as facilidades e dificuldades que os profissionais apresentam quanto a mínima intervenção. É de vital importância entender a carga de conhecimento dos profissionais e como eles têm lidado com a cárie dentária, para então investir na melhoria do aprendizado desses profissionais, com o fim de tornar a OMI o padrão de cuidado da cárie dentária.

# APÊNDICE

## **APÊNDICE 1** – Artigo 1 publicado


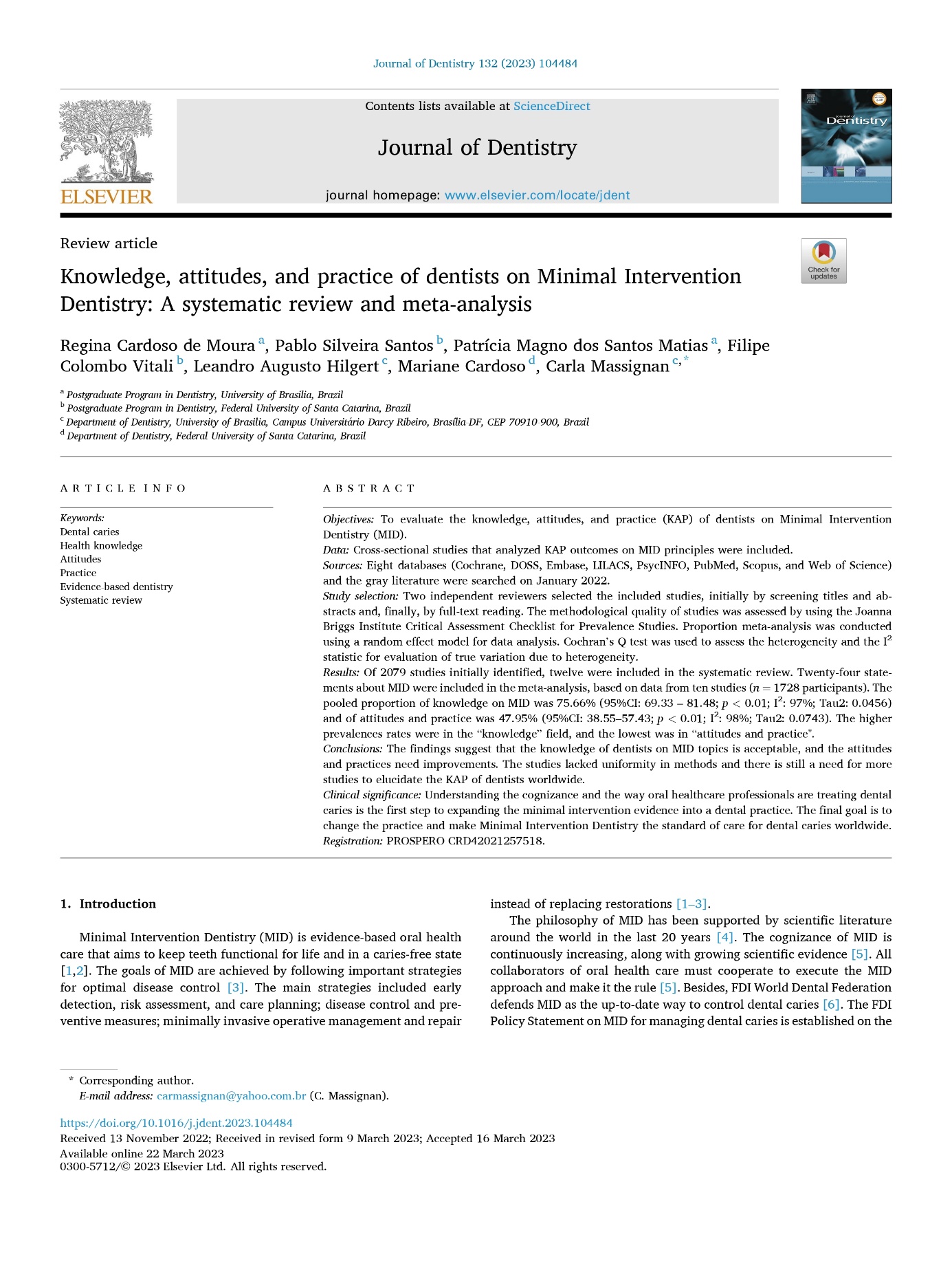


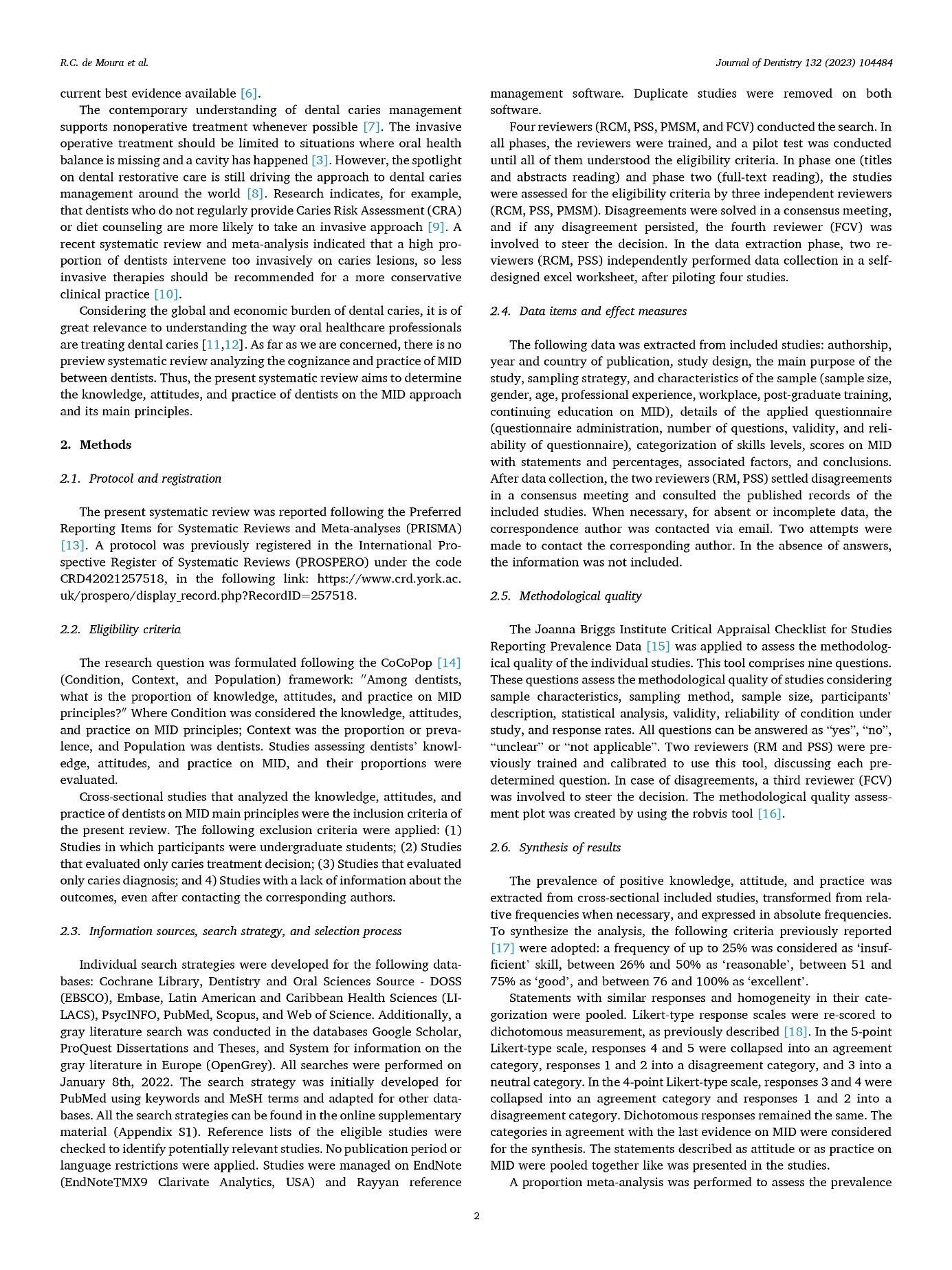

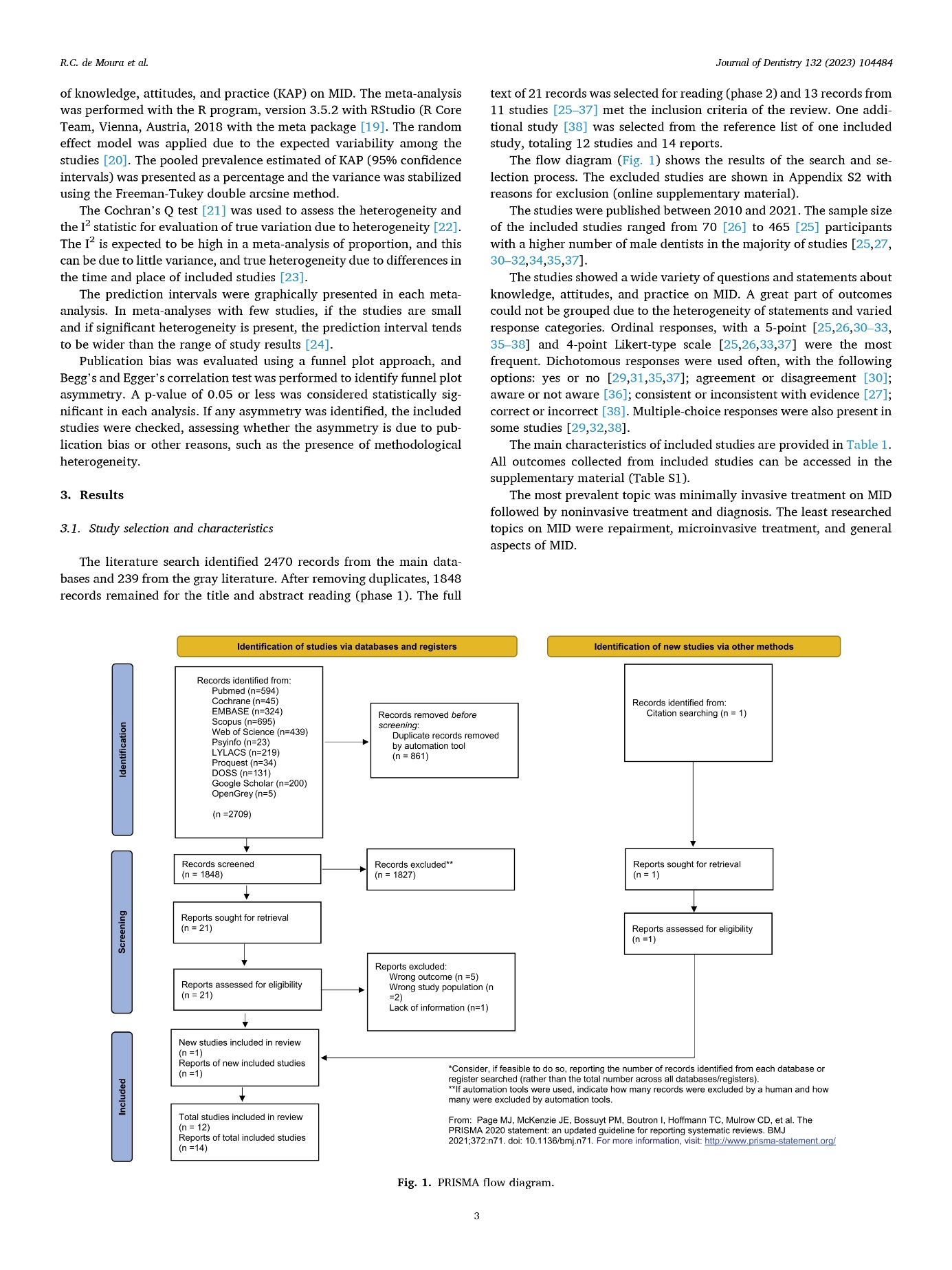

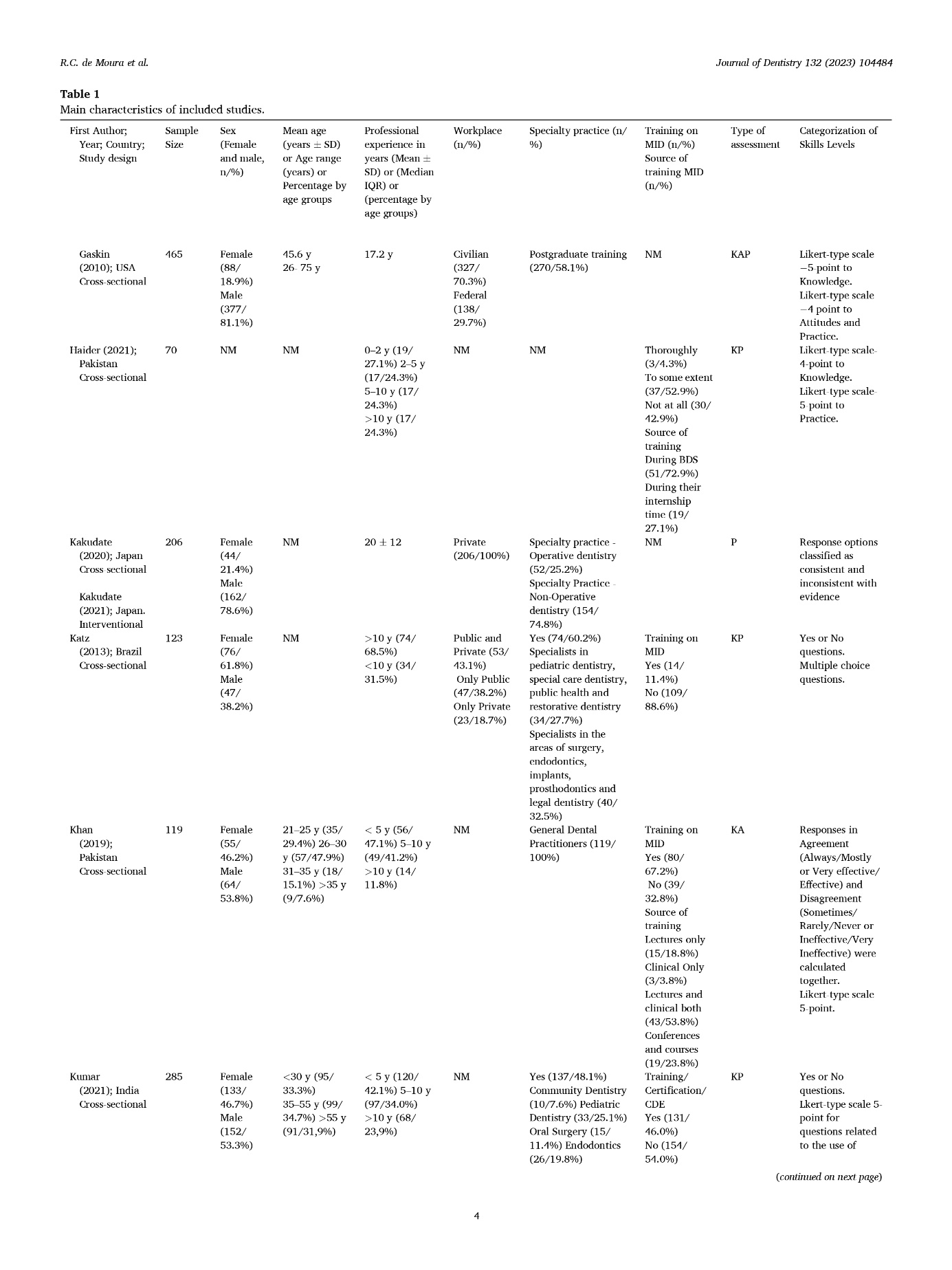

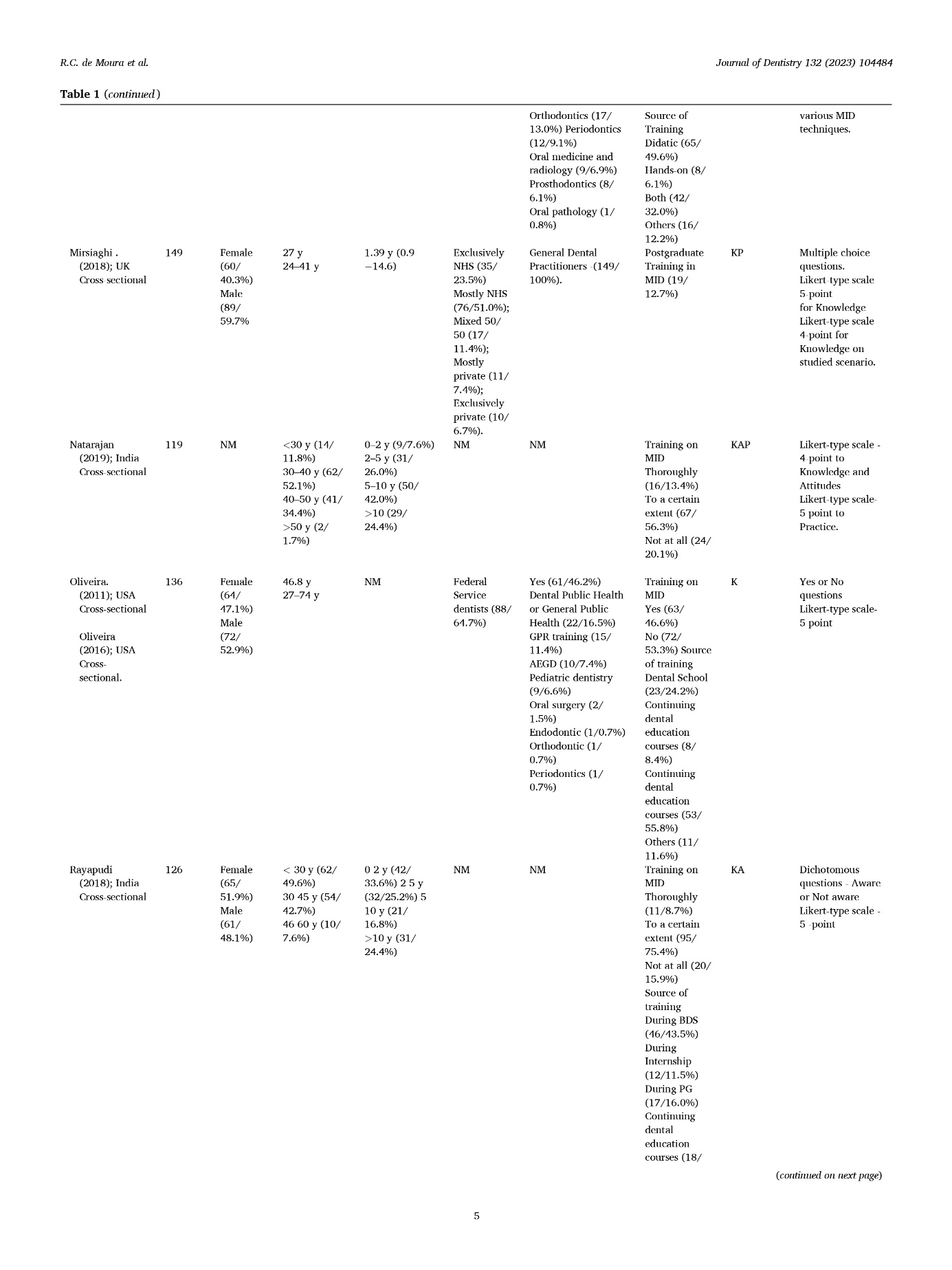

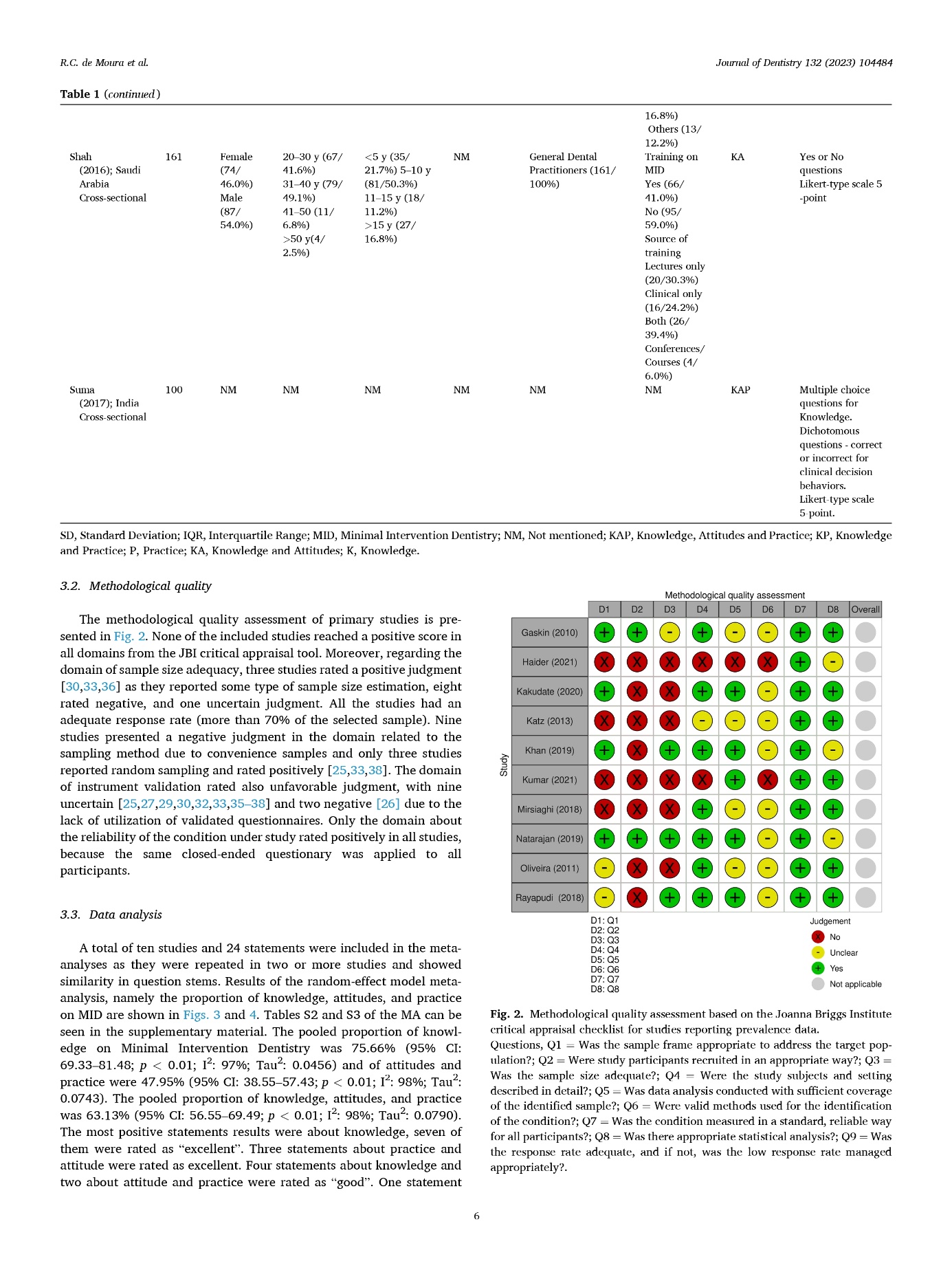

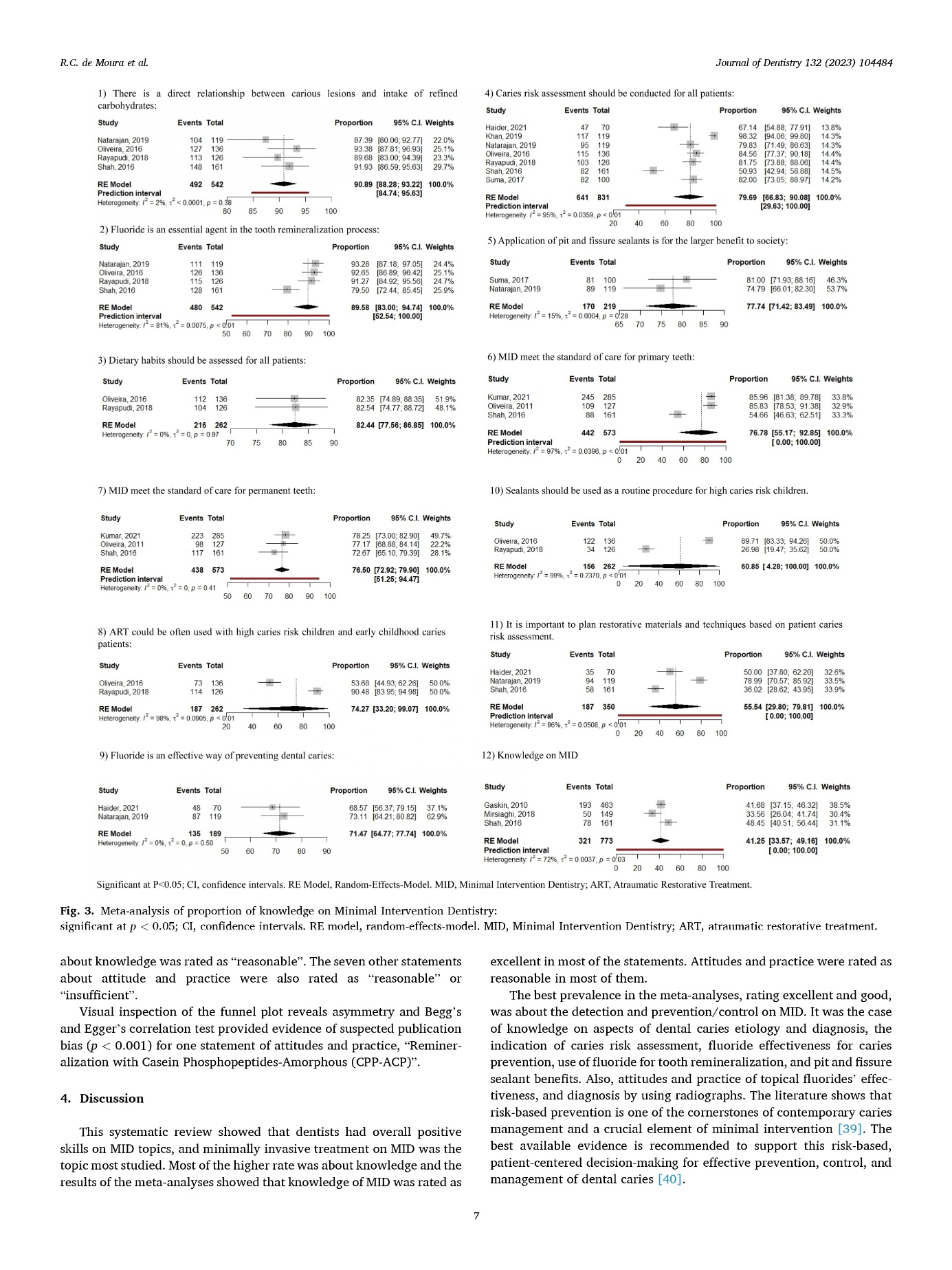

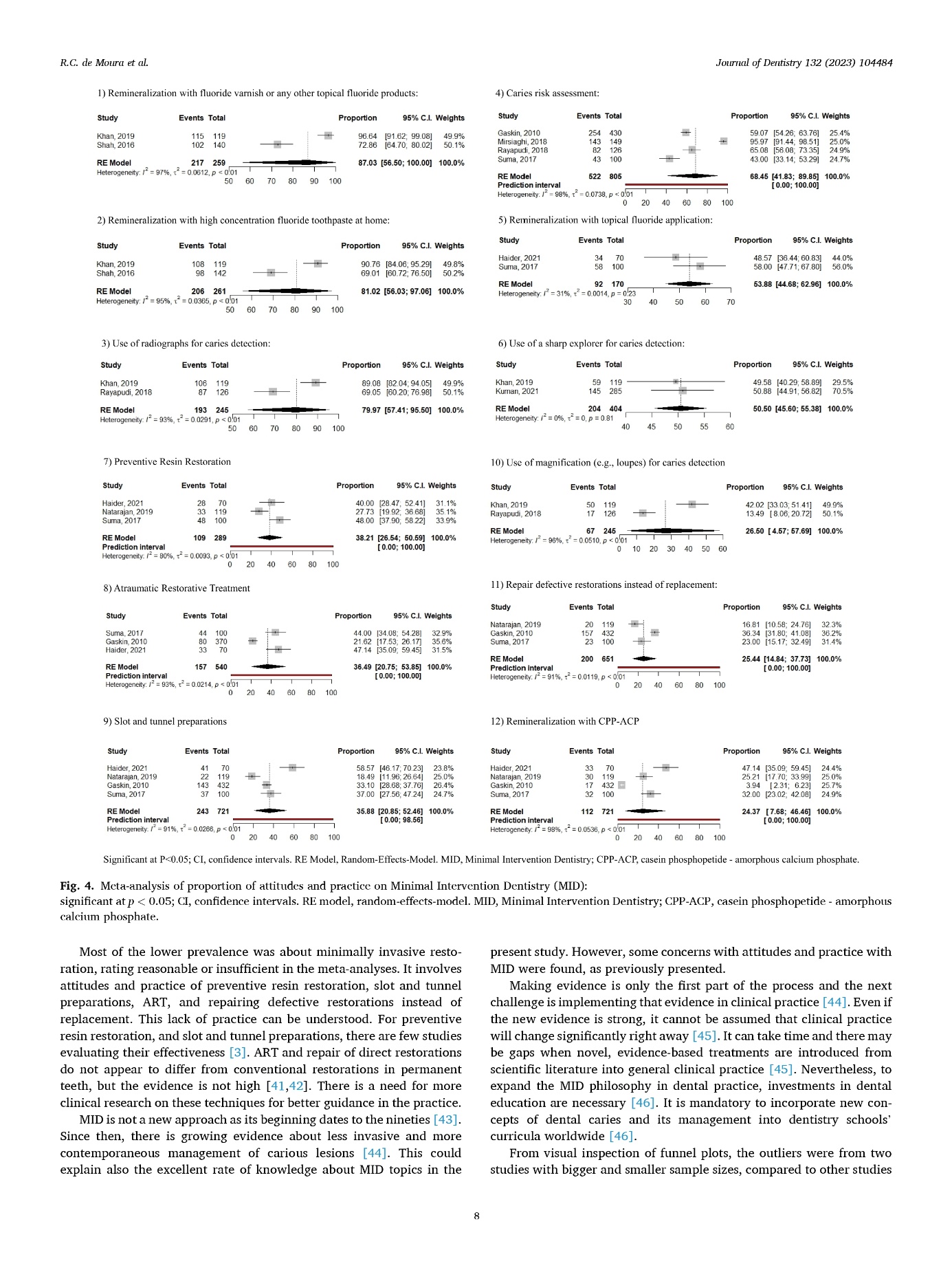

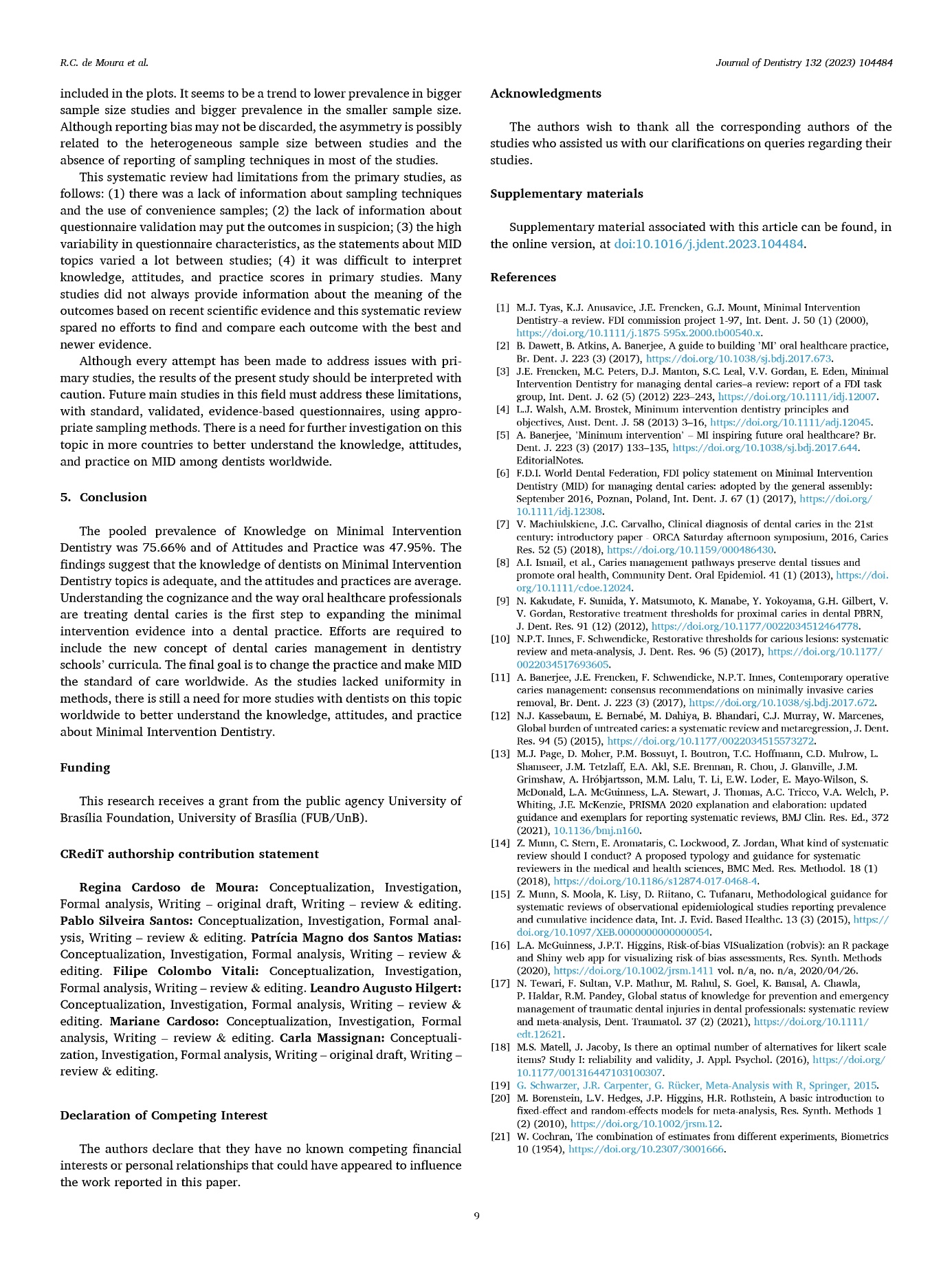

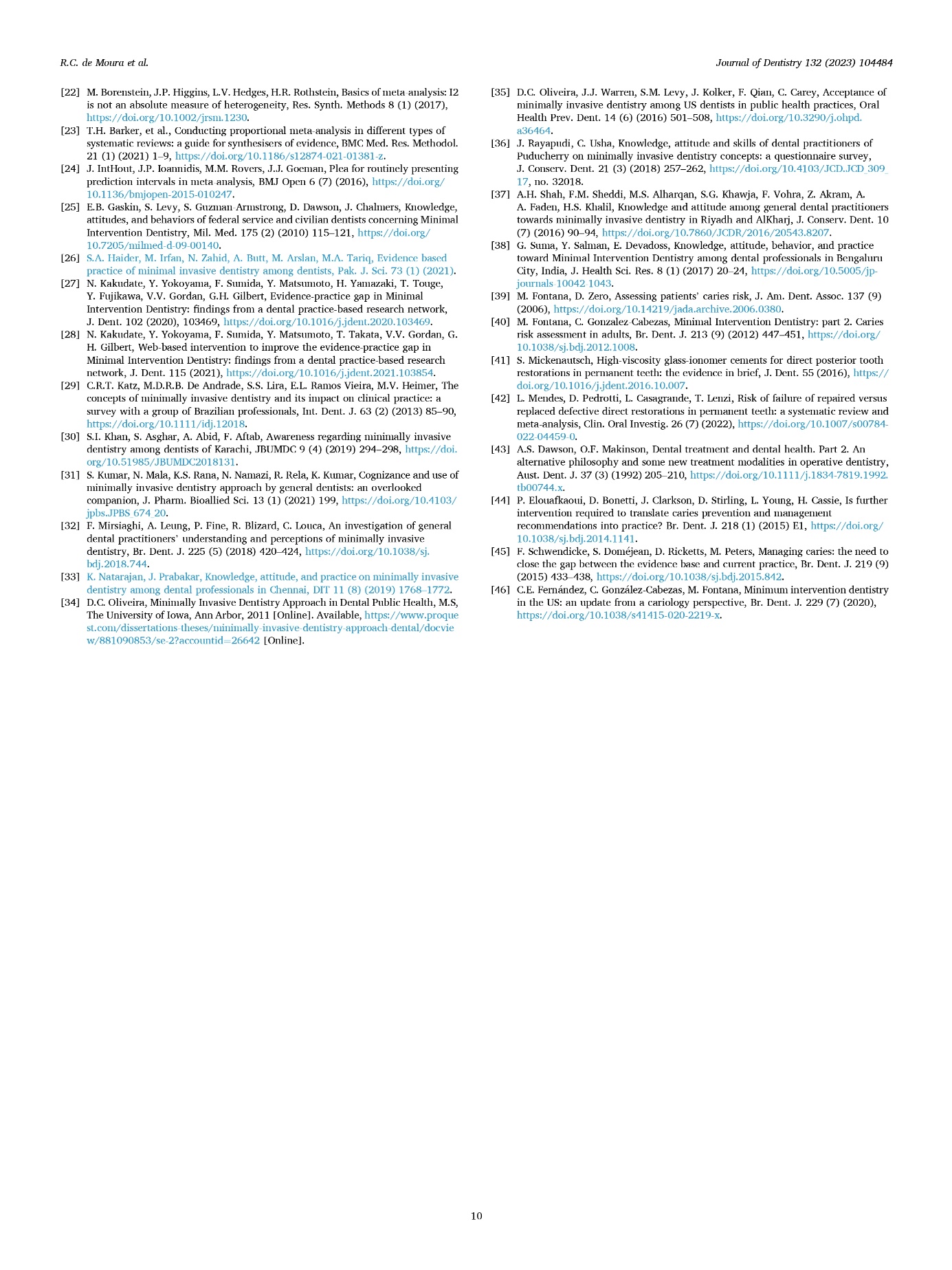


## **APÊNDICE 2** – Quadro da estratégia de busca das bases de dados

**Appendix S1** **–** Database Search Strategy.

| **Database** | **Search**  (January, 8^th^) |
| --- | --- |
| **Pubmed** | (("Minimally Invasive Dentistry" OR "Minimal Intervention Dentistry" OR "Evidence-Based Dentistry"[Mesh] OR "Evidence-Based Dentistry" OR "Dental Caries"[Title/Abstract]) AND ("Dentists"[Mesh] OR "Dentists"[Title/Abstract] OR "Dentist"[Title/Abstract] OR "Oral Medicine"[Mesh] OR "Oral Medicine" OR "Dental Practitioners"[Title/Abstract] OR "Dental Practitioner"[Title/Abstract] OR "Oral Health-Care Worker"[Title/Abstract] OR "Oral Health-Care Professionals"[Title/Abstract])) AND ("Health Knowledge, Attitudes, Practice"[Mesh] OR "Knowledge, Attitudes, Practice" OR "Health Behavior"[Mesh] OR "Health Behavior" OR "Health Behaviors" OR "Health-Related Behavior" OR "Health-Related Behaviors" OR "Health Related Behavior" OR "Health Related Behaviors" OR "Attitude Of Health Personnel"[Mesh] OR "Health Personnel Attitude" OR "Health Personnel Attitudes" OR "Staff Attitude" OR "Staff Attitudes" OR "Attitude To Health"[Mesh] OR "Health Attitude"[Title/Abstract] OR "Health Attitudes"[Title/Abstract] OR "Beliefs"[Title/Abstract] OR "Belief"[Title/Abstract] OR "Evidence-practice gap" OR "Knowledge, Attitude, Skills"[Title/Abstract] OR "Knowledge"[Title/Abstract])) |
| **Cochrane** | (("Minimally Invasive Dentistry" OR "Minimal Intervention Dentistry" OR "Dental Caries") AND ("Dentist" OR "Oral Medicine" OR "Dental Practitioner") AND ("Knowledge, Attitudes, Practice" OR "Health Attitude" OR "Belief" OR "Evidence-practice gap" OR "Knowledge, Attitude, Skills" OR "Knowledge")) in Title Abstract Keyword - (Word variations have been searched) |
| **EMBASE** | ('minimally invasive dentistry':ab,ti OR 'minimal intervention dentistry':ab,ti OR 'evidence-based dentistry':ab,ti OR 'dental caries':ab,ti) AND ('dentists':ab,ti OR 'dentist':ab,ti OR 'oral medicine':ab,ti OR 'dental practitioners':ab,ti OR 'dental practitioner':ab,ti OR 'oral health-care worker':ab,ti OR 'oral health-care professionals':ab,ti) AND ('health knowledge, attitudes, practice':ab,ti OR 'knowledge, attitudes, practice':ab,ti OR 'health behavior':ab,ti OR 'health behaviors':ab,ti OR 'health-related behavior':ab,ti OR 'health-related behaviors':ab,ti OR 'attitude of health personnel':ab,ti OR 'health personnel attitude':ab,ti OR 'health personnel attitudes':ab,ti OR 'staff attitude':ab,ti OR 'staff attitudes':ab,ti OR 'attitude to health':ab,ti OR 'health attitude':ab,ti OR 'health attitudes':ab,ti OR 'beliefs':ab,ti OR 'belief':ab,ti OR 'evidence-practice gap':ab,ti OR 'knowledge, attitude, skills':ab,ti OR 'knowledge':ab,ti) |
| **Scopus** | TITLE-ABS-KEY ( ( "Minimally Invasive Dentistry"  OR  "Minimal Intervention Dentistry"  OR  "Evidence-Based Dentistry"  OR  "Dental Caries" )  AND  ( "Dentists"  OR  "Dentist"  OR  "Oral Medicine"  OR  "Dental Practitioners"  OR  "Dental Practitioner"  OR  "Oral Health-Care Worker"  OR  "Oral Health-Care Professionals" )  AND  ( "Health Knowledge, Attitudes, Practice"  OR  "Knowledge, Attitudes, Practice"  OR  "Health Behavior"  OR  "Health Behaviors"  OR  "Health-Related Behavior"  OR  "Health-Related Behaviors"  OR  "Attitude Of Health Personnel"  OR  "Health Personnel Attitude"  OR  "Health Personnel Attitudes"  OR  "Staff Attitude"  OR  "Staff Attitudes"  OR  "Attitude To Health"  OR  "Health Attitude"  OR  "Health Attitudes"  OR  "Beliefs"  OR  "Belief"  OR  "Evidence-practice gap"  OR  "Knowledge, Attitude, Skills"  OR  "Knowledge" ) )  AND  ( LIMIT-TO ( DOCTYPE ,  "ar" ) )  AND  ( LIMIT-TO ( SUBJAREA ,  "DENT" ) ) |
| **Web of Science** | ((("Minimally Invasive Dentistry"  OR "Minimal Intervention Dentistry"  OR "Evidence-Based Dentistry”  OR "Dental Caries") )  AND (("Dentists”  OR "Dentist"  OR "Oral Medicine"  OR "Dental Practitioners"  OR "Dental Practitioner”  OR "Oral Health-Care Worker"  OR "Oral Health-Care Professionals"))  AND (("Health Knowledge, Attitudes, Practice"  OR "Knowledge, Attitudes, Practice"  OR "Health Behavior"  OR "Health Behaviors"  OR "Health-Related Behavior"  OR "Health-Related Behaviors"  OR "Attitude Of Health Personnel"  OR "Health Personnel Attitude"  OR "Health Personnel Attitudes"  OR "Staff Attitude"  OR "Staff Attitudes"  OR "Attitude To Health"  OR "Health Attitude"  OR "Health Attitudes"  OR "Beliefs"  OR "Belief"  OR "Evidence-practice gap"  OR "Knowledge, Attitude, Skills"  OR "Knowledge"))) |
| **PsycInfo** | Any Field: ("Minimally Invasive Dentistry" OR "Minimal Intervention Dentistry" OR "Evidence-Based Dentistry” OR "Dental Caries") AND ("Dentists” OR "Dentist" OR "Oral Medicine" OR "Dental Practitioners" OR "Dental Practitioner” OR "Oral Health-Care Worker" OR "Oral Health-Care Professionals") AND ("Health Knowledge, Attitudes, Practice" OR "Knowledge, Attitudes, Practice" OR "Health Behavior" OR "Health Behaviors" OR "Health-Related Behavior" OR "Health-Related Behaviors" OR "Attitude Of Health Personnel" OR "Health Personnel Attitude" OR "Health Personnel Attitudes" OR "Staff Attitude" OR "Staff Attitudes" OR "Attitude To Health" OR "Health Attitude" OR "Health Attitudes" OR "Beliefs" OR "Belief" OR "Evidence-practice gap" OR "Knowledge, Attitude, Skills" OR "Knowledge") AND Document Type: Journal Article |
| **LILACS** | ("Evidence-Based Dentistry" OR "Dental Caries" OR “dental decay” OR “odontologia baseada em evidências” OR “odontología basada en la evidencia”) AND (“dentists” OR “oral medicine” OR “dentista” OR “cirurgião-dentista”) AND ("Health Knowledge, Attitudes, Practice" OR "Knowledge, Attitudes, Practice" OR "Health Behavior" OR "Health Behaviors" OR "Health-Related Behavior" OR "Health-Related Behaviors" OR "Attitude Of Health Personnel" OR "Health Personnel Attitude" OR "Health Personnel Attitudes" OR "Staff Attitude" OR "Staff Attitudes" OR "Attitude To Health" OR "Health Attitude" OR "Health Attitudes" OR "Beliefs" OR "Belief" OR "Evidence-practice gap" OR "Knowledge, Attitude, Skills" OR "Knowledge" OR “conocimiento” OR “conhecimento”)  AND ( db:("LILACS")) |
| **ProQuest** | noft(("Minimally Invasive Dentistry" OR "Minimal Intervention Dentistry" OR "Evidence-Based Dentistry” OR "Dental Caries")) AND noft(("Dentists” OR "Dentist" OR "Oral Medicine" OR "Dental Practitioners" OR "Dental Practitioner” OR "Oral Health-Care Worker" OR "Oral Health-Care Professionals")) AND noft(("Health Knowledge, Attitudes, Practice" OR "Knowledge, Attitudes, Practice" OR "Health Behavior" OR "Health Behaviors" OR "Health-Related Behavior" OR "Health-Related Behaviors" OR "Attitude Of Health Personnel" OR "Health Personnel Attitude" OR "Health Personnel Attitudes" OR "Staff Attitude" OR "Staff Attitudes" OR "Attitude To Health" OR "Health Attitude" OR "Health Attitudes" OR "Beliefs" OR "Belief" OR "Evidence-practice gap" OR "Knowledge, Attitude, Skills" OR "Knowledge")) |
| **DOSS** | ("Minimally Invasive Dentistry" OR "Minimal Intervention Dentistry" OR "Evidence-Based Dentistry”) AND ("Dentists” OR "Dentist" OR "Oral Medicine" OR "Dental Practitioners" OR "Dental Practitioner” OR "Oral Health-Care Worker" OR "Oral Health-Care Professionals") AND ("Health Knowledge, Attitudes, Practice" OR "Knowledge, Attitudes, Practice" OR "Health Behavior" OR "Health Behaviors" OR "Health-Related Behavior" OR "Health-Related Behaviors" OR "Attitude Of Health Personnel" OR "Health Personnel Attitude" OR "Health Personnel Attitudes" OR "Staff Attitude" OR "Staff Attitudes" OR "Attitude To Health" OR "Health Attitude" OR "Health Attitudes" OR "Beliefs" OR "Belief" OR "Evidence-practice gap" OR "Knowledge, Attitude, Skills" OR "Knowledge") Full text AND Apply related words; Apply equivalent subjects |
| **Google Scholar** | ('Minimal Intervention Dentistry' OR 'Minimally Invasive Dentistry') AND (Dentist OR 'Oral Medicine') AND (Knowledge) |
| **OpenGrey** | ('Dental Caries') AND (Dentist OR 'Oral Medicine') AND (Knowledge) |

## **APÊNDICE 3** – Quadro dos artigos excluídos e razão para exclusão

**Appendix S2** - Excluded articles and reasons for exclusion (n=8).

| **Author, Year** | **Reason for exclusion** |
| --- | --- |
| Alkahtani et al 2020^1^ | 1 |
| Almahdy et al 2017 ^2^ | 2 |
| Brennan et al 2015^3^ | 2 |
| Chin et al 2016 ^4^ | 2 |
| Ganapathi et al 2020^5^ | 2 |
| Gupta et al 2015 ^6^ | 1 |
| Iqbal et al 2020^7^ | 3 |
| Reshmi et al 2020^8^ | 4 |

1) Participants were undergraduate students.

2) Studies that evaluated only caries treatment decision.

3) Studies that evaluated only caries diagnosis.

4) Studies with lack of information about the outcomes.

1. Alkahtani FM, Mallah RR, Ahmed N, Olayan A, Almalki BA. Knowledge and attitude among dental interns toward the conservative approach for incipient decay in Riyadh Region. International Journal of Medicine in Developing Countries. 2020;4(12):2153–7.

2. Almahdy A, AlShamrani M, Alqahtani M, Al M. The concepts of minimally invasive dentistry and its impact on clinical practice: a survey with a group of Dentists in Saudi Arabia. Age. 2017;3:0-52.

3. Brennan DS, Balasubramanian M, Spencer AJ. Treatment of caries in relation to lesion severity: Implications for minimum intervention dentistry. Journal of dentistry. 2015;43(1):58-65.

4. Chin ZW, Chong WS, Mani SA. Practice of Sealants and Preventive Resin Restorations Among Malaysian Dentists. Oral Health & Preventive Dentistry. 2016;14(2):125-35.

5. Ganapathi A, Prabakar J. Knowledge, Attitude, Practice About Chemo Mechanical Caries Removal Method in Dental Caries Among Dentist in Chennai City- A Cross-Sectional Study. Bioscience Biotechnology Research Communications. 2020;13(8):410-8.

6. Gupta G, Shanbhag N, Puranik MP. Perceptions regarding minimal intervention dentistry among dental interns in India: A cross-sectional survey. International Journal of Contemporary Dental and Medical Reviews. 2015;330115:1-5.

7. Iqbal N. Knowledge, Attitude and S kills of Endodontist and Pedodontist on use of recent advancements in caries diagnosis: a questionnaire based survey. International Journal of Medical Science And Diagnosis Research. 2020;4(6).

8. Reshmi B, Sri Sakthi D, Arvind S. Knowledge, Attitude and Practice Regarding Minimal Invasive Dentistry Among General Dental Practitioners in Tamil Nadu. Test Engineering and Management. 2020:17345 - 53.

## **APÊNDICE 4** – Tabela de desfechos dos estudos incluídos

**Table S1-** Outcomes of included studies.

| **First Author** | **Knowledge on MID - Question stems and percentages (total with event)** | **Attitude on MID- Question stems and percentages (total with event)** | **Practice on MID - Question stems and percentages (total with event)** | **Barriers to perform MID- Question stems and percentages (total with event)** | **Score** |
| --- | --- | --- | --- | --- | --- |
| Gaskin et al. (2010) | 1. Knowledge of Atraumatic Restorative Treatment (ART): Very Much/ Much 33.5% (155/462) - Reasonable  2. Knowledge of Minimal Intervention Dentistry (MID): Very Much/ Much 41.6% (192/463) - Reasonable | 1. Fluoride Is an Effective Remineralizing Agent, as an Indicator of Attitude Concerning Minimal Intervention Dentistry: Strongly Agree/Agree 96.3% (444/461) - Excellent  2. Restore and not extract a central incisor in HCR patient with limited finances: Always/Most of the Time 56.3% (253/449) - Good 3. Monitor and not restore a vital lower molar with a white spot lesion in LCR patient: Always /Most of the Time 70.0% (316/452) - Good 4. Monitor and not replace an anterior facial composite in LCR patient for whom esthetics is not a concern: Always/Most of the Time 67.1% (302/451) - Good   5. Monitor and not restore a proximal carious lesion in a HCR patient with limited finances: Always or Most of the Time 14.2% (64/451) - Insufficient | 1. Caries Risk Assessment:Always/ Most of the time 33.7% (145/430) - Reasonable  2. Microbial Testing: Always/Most of the time 0% (0/431) - Insufficient   3. Remineralize with CPP/ACP: Always/Most of the time 0% (2/432) - Insufficient  4. Remineralize Noncavitated Lesions: Always/Most of the time 13.8% (57/412) - Insufficient   5. Topical Fluoride Application: Always/Most of the time 41.9% (181/432) - Reasonable  6. Prescribe Chlordexidine:Always/Most of the time 6.9% (30/432) - Insufficient  7. Prescribe 5,000 ppm Fluoride Dentifrice: Always/Most of the time 20.1% (87/433) -Insufficient  8. Seal Amalgams: Always/Most of the time 13.5% (58/431) - Insufficient   9. Seal Composites: Always/Most of the time 19.6% (83/423) - Insufficient  10. Repair Restorations: Always/Most of the time 6.1% (26/424) - Insufficient  11. Slot and Tunnel Preps: Always/Most of the time 8.5% (36/422) - Insufficient  12. Redo Restorations: Always/Most of the Time 5% (21/423) - Insufficient  13. Sandwich Technique: Always or Most of the time 8.4% (32/381) - Insufficient  14. Restore with Glass Ionomer: Always or Most of the time 4.0% (17/424) - Insufficient 15. Atraumatic Restorative Treatment: Always or Most of the time 4.1% (15/370) - Insufficient | NM | Knowledge - Mean = 37.6%   Attitudes - Mean = 60.8%  Practice –  Mean = 12.4% |
| Haider et al. (2021) | 1. Do you think caries assessment should be carried out for all patients: Strongly Agree/Agree 67.1% (47/70) - Good  2. Do you think fluoride is an effective way of preventing dental caries: Strongly Agree/Agree 68.6% (48/70) - Good  3. Is it important to plan restorative materials and techniques based on patient caries risk assessment: Strongly Agree/Agree 50.0% (35/70) - Reasonable | NM | 1. Slot and tunnel preparations: Often/Always 58.6% (41/70) - Good  2. Remineralization with topical fluoride application: Often/Always 48.60% (34/70) - Reasonable 3. Remineralization with CPP-ACP: Often/Always 47.1% (33/70) - Reasonable 4. Preventive Resin Restoration in clinical practice: Often/Always 40.0% (28/70) - Reasonable 5. Atraumatic Restorative Treatment in clinical practice: Often/Always 47.1% (33/70) - Reasonable | NM | Knowledge - Mean = 61.9%  Practice –  Mean = 48.3% |
| Kakudate et al. (2020);   Kakudate et al. (2021); | NM | NM | 1. Use of air drying (≥80 % of the time): 77.2% (159/206) - Excellent 2. Assessment of caries risk for individual patients in any way: 51.5 % (106/206) - Good 3. Treatment of unrestored occlusal surface (occlusal 1): 97.1% (200/206) - Excellent 4. Treatment of unrestored occlusal surface (occlusal 2): 81.6% (168/206) - Excellent 5. Use of magnification (≥80 % of the time): 32.5% (67/206) - Reasonable 6. Treatment options for patient with deep occlusal caries (deep caries 1): 51.9% (107/206) - Good 7. Treatment options for excavation of deep caries (deep caries 2): 54.4% (112/206) - Good 8.Defective composite restoration with enamel margins: 57.8% (119/200) - Good 9. Defective composite restoration with cementumdentinal margins: 46.6% (96/200) - Reasonable 10.Lesion depth for permanent restoration (proximal caries): 52.9% (109/206) – Good | NM | Practice –  Mean = 60.4% |
| Katz et al. (2013) | 1. Concepts on dental caries: Showed knowledge 13.0% (16/123) - Insufficient 2. Concepts of the treatment of dental caries: Showed knowledge 34.1%. (42/123) - Reasonable  3 .Removal of carious dentine: Regardless of total removal 48.0% (59/123) - Reasonable  4. Material: Regardless of the material 21.1% (26/123) - Insufficient 5. Efficient restoration (hermetic): It depends of the restoration 84.6% (104/123) - Excellent | NM | 1.Knowledge about MID techniques: Yes 82.1% (101/123) - Excellent  2. Practice of MID: Yes 49.6% (61/123) - Reasonable 3.Conduct in relation to the practice of MID: Permanent 38.2% (47/123) - Reasonable 4. Patients who used MID procedures: Any patient 65.6% (40/61) - Good 5.Dental elements used MID in procedures: Primary and permanent teeth 31.1% (19/61) - Reasonable | 1. Did not believe in the technique: 22.6% (14/62)  2. Did not know how to perform them: 25.8% (16/62)   3. Did not have patient supervision: 6.5% (4/62)  4. Did not perform restorative procedures: 16.1% (10/62) No answer 29.0% (18/62) | Knowledge - Mean = 40.2%  Practice –  Mean = 53.3% |
| Khan et al. (2019) | 1. All patients should go through Caries Risk Assessment (CRA): Strongly Agree/Agree 98.3% (117/119) - Excellent 2. Tunnel and box preparations are effective cavity designs: Strongly Agree/Agree 79.0% (94/119) - Excellent | 1. Do you use of a sharp explorer for caries detection: Sometimes/Rarely/Never 50.4% (60/119) - Reasonable  2. Do you use of a blunt instrument for caries detection: Always/Mostly 63.0% (75/119) - Good  3. Do you use magnification (e.g. loupes) for caries detection: Always/Mostly 42% (50/119) - Reasonable 4.Use of radiographs for caries detection:Always/Mostly 89.1% (106/119) - Excellent 5. Do you use newer methods of caries detection like QLF,ECM, IRLF, FOTI: Always/Mostly 10.9% (13/119) - Insufficient 6.How effective is Atraumatic Restorative Treatment: Very effective/Effective 94.1% (112/119) - Excellent  7.How effective is Sandwich Technique (Glass Ionomer + Composite): Very effective/Effective 99.2% (118/119) - Excellent  8. Do you think remineralization with fluoride varnish or any other topical fluoride products is effective: Very effective/Effective 96.6% (115/119) - Excellent 9. Do you think remineralization with high concentration fluoride toothpaste at home Is effective: Very effective/Effective 90.8% (108/119) - Excellent | NM | NM | Knowledge - Mean = 88.7%   Practice –  Mean = 70.7% |
| Kumar et al. (2021); | 1. Aware of MID: Yes 95.1% (271/285) - Excellent 2. MID standard care for treatment of primary teeth: Yes 86.0% (245/285) - Excellent 3. MID standard care for treatment for permanent teeth: Yes 78.2% (223/285) - Excellent | NM | 1. Sharp explorer for caries detection: Always/ Most of the time 50.9% (145/285) - Reasonable Primary teeth 2. Atraumatic Restorative Treatmentt: Very effective/Effective 71.2% (203/285) - Good 3. Sandwich technique: Very effective/Effective 74.0% (211/285) - Good 4. Fluoride varnish: Very effective/ Effective 85.6% (244/285) - Excellent 5. Topical high-concentration fluoride at home: Very effective/ Effective 56.1% (160/285) - Good Permanent teeth 6. . Atraumatic Restorative Treatment: Very effective/Effective 57.9% (165/285) - Good 7. Sandwich technique: Very effective/Effective 55.1% (156/285) - Good 8. Fluoride varnish: Very effective/Effective 81.1% (231/285) - Excellent 9. Topical high-concentration fluoride at home: Very effective/Effective 82.1% (234/285) - Excellent | NM | Knowledge - Meean = 86.4%   Practice – Mean = 68.2% |
| Mirsiaghi et al. (2018) | 1. Knowledge on MID: A great deal/Quite a lot 33.6% (50/149) - Reasonable 1a. Would you treat and restore an interproximal lesion with a radiographic radiolucency confined to enamel, in a vital, asymptomatic, lower first molar in a low caries risk patient aged 25 years: Rarely/Never 83.9% (125/149) - Excellent 1b. Referring to the same patient, if the caries risk is high, what would your treatment be: Observe the lesion 68.9% (102/148) - Good | NM | 1.Caries risk assessment: Always/Most of the time 96% (143/149) - Excellent 2.Caries risk assessment affecting treatment planning: Always/Most of the time 91.3% (136/149) - Excellent  3. Assessment of patient’s dietary habits: Always/Most of the time 71.1% (106/149) - Good  4. Fluoride usage: Always/Most of the time 71.8% (107/149) - Good  5.Application of G. V. Black concept: Rarely/Never 64.4% (43/147) - Good 6. Frequency of adhesive restorative material usage: Always/Most of the time 65.8 (98/149) - Good  7.Frequency of amalgam usage: Always/Most of the time 41.2% (61/148) – Reasonable | NM | Knowledge - Mean = 62.1%  Practice –  Mean = 71.7% |
| Natarajan and Prabakar (2019) | 1. There is a direct relationship between carious lesions and intake of refined carbohydrates: Strongly agree/Agree 87.4% (104/119) - Excellent  2. Fluoride is an essential agent in tooth mineralization: Strongly agree/Agree 93.3% (111/119) - Excellent 3. Sealants should be indicated as a routine preventive procedure for high caries risk patients: Strongly agree/Agree 80.7% (96/119) - Excellent  4. Preventive resin restoration (PRR) is effective in the treatment of caries in permanent dentition: Strongly agree/Agree 80.7% (96/119) - Excellent | 1. Do you think caries assessment should be carried out for all patients: Strongly agree/Agree 79.8% (95/119) - Excellent 2. Do you think fluoride is an effective way of preventing dental caries: Strongly agree/Agree 73.1% (21/119) - Good 3. Is it important to plan restorative materials and techniques based on patient caries risk assessment: Strongly agree/Agree 79.0% (94/119) - Excellent 4. Do you think application of pit and fissure sealants is for the longer benefit to society: Strongly agree/Agree 74.8% (89/119) - Good 5. Use of magnification loupes – Diagnodent – is effective in diagnosing early carious lesion: Strongly agree/Agree 49.6% (59/119) - Reasonable | 1. Slot and tunnel preparations: Often/Always 18.5% (22/119) - Insufficient  2. Remineralization with CPP‑ACP: Always/Often 25.2% (30/119) - Insufficient 3. Preventive Resin Restoration in clinical practice: Always/Often 27.7% (33/119) - Reasonable  4. Repair defective restorations instead of replacement: Always/Often 16.8% (20/119) - Insufficient | NM | Knowledge - Mean = 85.5%    Attitude - Mean = 71.3%  Practice –  Mean = 22,1% |
| Oliveira. (2011);   Oliveira et al. (2016); | 1. Does MID meet the standard of care for primary teeth: Yes 85.8% (109/127) - Excellent 2. Does MID meet the standard of care for permanent teeth: Yes 77.2% (98/127) - Excellent 3. How much have you heard about MID: Very much/Much 48.5% (66/136) - Reasonable 4. There is a direct relationship between carious lesions and intake of refined carbohydrate: Strongly agree/Agree 93.4% (127/136) - Excellent 5. Fluoride is essential agent in tooth remineralization process: Strongly agree/Agree 92.6% (126/136) - Excellent  6. Sealants are not very effective in prevention of pit and fissures caries: Strongly disagree/Disagree 89.7% (122/136) - Excellent 7.Sealants should be used as a routine procedure for high caries risk children: Strongly agree/Agree 89.7% (122/136) - Excellent  8. When possible a tunnel preparation design for proximal caries lesions should be used instead of a traditional proximal box in adults: Strongly agree/Agree 34.6% (47/136) - Reasonable 9. Preventive dentistry is more important for children than adults: Strongly disagree/Disagree 60.3% (82/136) - Good  10. Caries risk assessment should be conducted with all patients: Strongly agree/Agree 84.6% (115/136) - Excellent  11. Dietary habits should be assessed for all patients: Strongly agree/Agree 82.4% (112/136) - Excellent  12. Small minimal cavity preparations compromise materials’ retention: Strongly disagree/Disagree 58.5% (80/136) - Good  13. Dentists should recommend that high caries risk patients receive diet counseling: Strongly agree/Agree 83.8% (114/136) - Excellent 14. Atraumatic Restorative Treatment does not meet the standard of care for dentistry in the USA: Strongly disagree/Disagree 55.9% (76/136) - Good  15. The use of a closed sandwich technique is appropriate for children and adults: Strongly agree/Agree 75.0% (102/136) - Good 16. Atraumatic Restorative Treatment could be often used with high caries risk children and early childhood caries patients: Strongly agree/Agree 53.7% (73/136) - Good 17. How effective is Atraumatic Restorative Treatment as caries treatment for primary teett: Very effective/effective 87.6% (92/105) - Excellent 18. How effective is Atraumatic Restorative Treatmentas caries treatment for permanent teeth: Very effective/effective 66.7% (70/105) - Good  19. How effective the sandwich technique as caries treatment for primary teeth: Very effective/effective 82.3% (93/113) - Excellent  20. How effective sandwich technique as caries treatment for permanent teeth: Very effective/effective 94.4% (102/108) - Excellent  21. How effective is fluoride varnish as caries treatment for primary teeth:Very effective/effective 86.5% (109/126) - Excellent  22. How effective is fluoride varnish as caries treatment for permanent teeth: Very effective/effective 82.7% (105/127) - Excellent  23. How effective is topical high-concentration fluoride at home as caries treatment for primary teeth: Very effective/effective 66.1% (80/121) - Good  24. How effective is topical high concentration fluoride at home as caries treatment for permanent teeth: Very effective/effective 72.0% (90/125) - Good 25. How effective is MI paste as caries treatment for primary teeth: Very effective/effective 75.8% (69/91) - Good 26. How effective is MI paste as caries treatment for permanent teeth: Very effective/effective 78.5% (73/93) - Excellent | NM | NM | NM | Knowledge - Mean =75.3% |
| Rayapudi and Usha (2018) | Awareness about:  1. Atraumatic Restorative Treatment: 90,5% (114/126) - Excellent  2. Sandwich Techinique 94,4% (119/126) - Excellent  3. Pit & Fissure Sealant 91,3% (115/126) - Excellent 4. Fissurotomy 69.0% (87/126) - Good  5. Preventive Resin Restoration 83,30% (105/126) - Excellent 6. Tunnel Preparation 72,20% (91/126) - Good 7. Fluoride is an essential agent in the tooth remineralization process 90.5% (114/126) - Excellent 8. Fluoride toothpaste 77.90% (98/126) - Excellent 9. In-office fluoride varnish 67.90% (86/126)- Good 10. Fluoride mouthrinse 66.40% (84/126) - Good 11. CPP ACP (Casein Phosphopeptide and Amorphous Calcium Phosphate) paste 64.10% (81/126) - Good 12. Xylitol chewing gum 45% (57/126) - Reasonable 13. There is a direct relationship between carious lesions and intake of refined carbohydrate: Strongly Agree/Agree 89.7% (113/126) - Excellent 14. Fluoride is an essential agent in the tooth remineralization process: Strongly Agree/Agree 91.3% (115/126) - Excellent  15. Sealant should be used as a routine procedure for high caries‑risk children: Strongly Agree/Agree 27% (34/126) - Reasonable  16. “Sandwich technique” (glass ionomers + composite) is effective in the treatment of caries in permanent teeth: Strongly Agree/Agree 94.4% (119/126) - Excellent  17. Pit and fissure sealant is effective in the treatment of caries in permanent teeth: Strongly Agree/Agree 91.3% (115/126) - Excellent  18. Preventive resin restoration is effective in the treatment of caries in permanent teeth: Strongly Agree/Agree 83.3% (105/126) - Excellent  19. Carries Risk Assesment should be conducted with all patients: Strongly Agree/Agree 81.7% (103/126) - Excellent  20. Dietary habits should be assessed in all patients: Strongly Agree/Agree 82.5% (104/126) - Excellent | 1. Identify current exposure to fluoride: Always/Most of the time 73.0% (92/126) - Good  2. Plan restorative materials and techniques based on the patient’s Caries Risk Assessment: Always/Most of the time 90.5% (114/126) - Excellent 3. Use of explorer that is not sharp: Always/Most of the time 25.4% (32/126) - Insufficient 4. Magnification (loupes/microscope): Always/Most of the time 13.5% (17/126) - Insufficient  5. Radiographs: Always/Most of the time 69.0% (87/126) - Good 6. Light transmission (FOTI: Diagnodent): Always/Most of the time 13.5% (17/126) - Insufficient | NM | NM | Knowledge - Mean = 77,7%  Attitude - Mean = 47.5% |
| Shah et al. (2016) | 1. MID principles met the standards of care for permanent teeth: Yes 72.7% (117/161) - Good 2. MID principles met standards of care for primary teeth: Yes 54.7% (88/161) - Good 3. There is a direct relationship between carious lesions and intake of refined carbohydrates: Strongly Agree/Agree 91.9% (148/161) - Excellent  4. Fluoride is an essential agent in the tooth remineralization process: Strongly Agree/Agree 79.5% (128/161) - Excellent 5. Sealants are effective for pit and fissure caries prevention: Strongly Agree/Agree 60.2% (97/161) - Good  6.Caries risk assessment should be conducted with all patients: Strongly Agree/Agree 50.9% (82/161) - Reasonable  7. Conservative cavity design like tunnel and box preparations are effective: Strongly Agree/Agree 55.9% (90/161) - Good  8. Plan restorative materials and techniques based on the patient’s caries risk assessment: Strongly Agree/Agree 36.0% (58/161) - Reasonable  9. Practitioners’ Knowledge about MID: Very much/Much 48.4% (78/161) - Reasonable | 1. Use of a sharp explorer for caries detection: Always/Most times/Often 79.5% (128/161) - Excellent  2. Use of a blunt instrument for caries detection: Always/Most times/Often 81.4% (131/161) - Excellent  3. Use of magnification (e.g. loupes) for caries detection: Always/Most times/Often 41.0% (66/161) - Reasonable  4. Use of radiographs for caries detection: Always/Most times/Often 55.3% (89/161) - Good 5. Newer methods of caries detection ECM, QLF, IRLF, FOTI: Always/Most times/Often 32.3% (52/161) - Reasonable  6. ART (Atraumatic Restorative Treatment): Very effective/Effective 78.8% (108/137) - Excellent  7. Sandwich Technique (Glass Ionomer +Composite): Very effective/Effective 75.4% (107/142) - Good  8. Remineralization with fluoride varnish or any other topical fluoride products: Very effective/Effective 72.9% (102/140) - Good  9. Remineralization with high concentration fluoride toothpaste at home (Duraphat 2800/5000 ppm F): Very effective/Effective 69.0% (98/142) - Good | NM | NM | Knowledge - Mean = 61.1%  Attitude - Mean = 65.1% |
| Suma and Salman (2017) | 1. Atraumatic restorative treatment is done with the help of: Correct 99% (99/100) - Excellent 2. Material used for pit and fissure sealants are: Correct 82% (82/100) - Excellent 3. Preventive resin restoration is based on principles of: Correct 80% (80/100) - Excellent 4. Which of the following is not used for remineralization of teeth: Correct 96% (96/100) - Excellent | 1. Do you think fluoride application is an  effective way of preventing dental caries: Strongly Agree/Agree 96% (96/100) - Excellent  2. Do you think G. V. Black’s “extension for prevention” is relevant for initial caries: Strongly Disagree/Disagree 60% (60/100) - Good  3. Do you think adhesive restorative materials have helped in preserving tooth structure: Strongly Agree/Agree 78% (78/100) - Excellent  4. Do you think caries risk assessment should be carried out for all patients: Agree 59/100 (59%) Strongly Agree/Agree 82% (82/100) - Excellent 5. Do you think application of pit and fissure sealants is for the larger benefit to society? Strongly Agree/Agree 81% (81/100) - Excellent | 1. Opacity with air-drying: White/brown is not consistent with the clinical appearance of sound enamel and is limited to the confines of the pit and fissure area - fluoride and/or pit and fissure sealants Correct 99% (99/100) - Excellent  2. Opacity without air-drying: White, brown, which is wider than the natural fissure/fossa, i.e., not consistent with the clinical appearance of sound enamel - fluoride and/or pit and fissure sealants: Correct 48% (48/100) - Reasonable 3. Surface integrity loss: The base and walls of the cavity within enamel and dentin are not visible - Sealants and Preventive Resin Restoration: Correct 79% (79/100) - Excellent 4. Underlying gray shadow: This lesion appears as a shadow of discolored dentin visible through an apparently intact enamel surface, which may or may not show signs of localized breakdown - surgical or conventional GV Black principle: Correct 48% (48/100) - Reasonable  5. Distinct cavity: There is frank cavitations, and dentin is exposed - surgical or conventional GV Black principle: Correct 100% (100/100) (100%) - Excellent  6. Extensive cavity: Obvious loss of tooth structure and dentin is clearly visible on the walls and at the base in a cavity that involves at least half of the tooth surface - surgical or conventional GV Black principle: Correct 95% (95/100) - Excellent 7. Caries risk assessment (diet, oral hygiene, salivary test, etc.) Always/ Most of the time/Often 43% (43/100) - Reasonable  8. Remineralize with CPP/amorphous calcium phosphate: Always/ Most of the time/Often 32% (32/100) - Reasonable  9. Remineralize with topical fluoride application: Always/Most of the time/Often 58% (58/100) - Good  10. Prescribe chlorhexidine for caries control: Always/ Most of the time/ Often 40% (40/100) - Reasonable  11 . Seal adjacent pits and fissures of amalgam restorations with a sealant: Always/Most of the time/Often 31% (31/100) - Reasonable  12. Seal adjacent pits and fissures of composite restorations with a sealant: Always/ Most of the time/Often 48% (48/100) - Reasonable  13. Repair defective restorations instead of replacement: Always/ Most of the time/Often 23% (23/100) - Insufficient  14. Slot and tunnel preparations: Always/Most of the time/Often 37% (37/100) - Reasonable 15. Preventive resin restoration: Always/ Most of the time/Often 48% (48/100) -Reasonable 16. Atraumatic restorative treatment: Always/ Most of the time/Often 44% (44/100) - Reasonable | NM | Knowledge - Mean = 89.3%  Attitude - Mean = 74.8%  Practice - Mean = 54.6% |

MID, Minimal Intervention Dentistry; NM, Not Mentioned.

## **APÊNDICE 5** – Tabela de Metanálise de proporção de conhecimentos em Odontologia de Mínima Intervenção

**Table 3 -** Proportion meta-analysis of Knowledge on Minimal Intervention Dentistry

| Knowledge on MID | Studies | Sample | Prevalence (%) | CI 95% | p-value | I^2^ | Tau^2^ | Classification |
| --- | --- | --- | --- | --- | --- | --- | --- | --- |
| There is a direct relationship between carious lesions and intake of refined carbohydrates | 4 | 542 | 90.89 | 88.28 – 93.22 | 0.38 | 2 | <0.0001 | Excellent |
| Fluoride is an essential agent in the tooth remineralization process | 4 | 542 | 89.58 | 83.00 – 94.74 | < 0.01 | 81 | 0.0075 | Excellent |
| Dietary habits should be assessed for all patients | 2 | 262 | 82.44 | 77.56 – 86.85 | 0.97 | 0 | 0 | Excellent |
| Caries risk assessment should be conducted for all patients | 7 | 831 | 79.69 | 66.83 – 90.08 | < 0.01 | 95 | 0.0359 | Excellent |
| Application of pit and fissure sealants is for the larger benefit to society | 2 | 219 | 77.74 | 71.42 – 83.49 | 0.28 | 15 | 0.0004 | Excellent |
| MID meet the standard of care for primary teeth | 3 | 573 | 76.78 | 55.17 – 92.85 | < 0.01 | 97 | 0.0396 | Excellent |
| MID meet the standard of care for permanent teeth | 3 | 573 | 76.50 | 72.92 – 79.90 | 0.41 | 0 | 0 | Excellent |
| ART could be often used with high caries risk children and early childhood caries patients | 2 | 262 | 74.27 | 33.20 – 99.07 | <0.01 | 98 | 0.0905 | Good |
| Fluoride is an effective way of preventing dental caries | 2 | 189 | 71.47 | 64.77 – 77.74 | 0.50 | 0 | 0 | Good |
| Sealants should be used as a routine procedure for high caries risk children | 2 | 262 | 60.85 | 4.28 – 100.00 | <0.01 | 99 | 0.2370 | Good |
| It is important to plan restorative materials and techniques based on patient caries risk assessment. based on patient’s caries risk assessment | 3 | 350 | 55.54 | 29.80 – 79.81 | < .001 | 96 | 0.0508 | Good |
| Knowledge on MID | 3 | 773 | 41.25 | 33.57 – 49.16 | 0.03 | 72 | 0.0037 | Reasonable |

MID, Minimal Intervention Dentistry; ART, Atraumatic Restorative Treatment.

*CI 95%=confidence interval 95%; I^2^=ratio of true heterogeneity to total observed variation.

** A frequency of up to 25% was considered as ‘insufficient’ skill, between 26% and 50% as ‘reasonable’,

between 51 and 75% as ‘good’, and between 76 and 100% as ‘excellent’.^16^

## **APÊNDICE 6** – Tabela de Metanálise de proporção de atitudes e prática em Odontologia de Mínima Intervenção

**Table 4 -** Proportion meta-analysis of Attitude and Practice on Minimal Intervention Dentistry

| Attitude and Practice on MID | Studies | Sample | Prevalence % | CI 95% | p-value | I^2^ | Tau^2^ | Classification** |
| --- | --- | --- | --- | --- | --- | --- | --- | --- |
| Remineralization with fluoride varnish or any other topical fluoride products | 2 | 259 | 87.03 | 56.50 – 100.00 | <0.01 | 97 | 0.0612 | Excellent |
| Remineralization with high concentration fluoride toothpaste at home | 2 | 261 | 81.02 | 56.03 – 97.06 | <0.01 | 95 | 0.0365 | Excellent |
| Use of radiographs for caries detection | 2 | 245 | 79.97 | 57.41 – 95.50 | <0.01 | 93 | 0.0291 | Excellent |
| Caries risk assessment | 4 | 805 | 68.45 | 41.83 – 89.85 | < .001 | 98 | 0.0738 | Good |
| Remineralization with topical fluoride application | 2 | 170 | 53.88 | 44.68 – 62.96 | 0.23 | 31 | 0.0014 | Good |
| Use of a sharp explorer for caries detection | 2 | 404 | 50.50 | 45.60 – 55.38 | 0.81 | 0 | 0 | Reasonable |
| Preventive Resin Restoration | 3 | 289 | 38.21 | 26.54 –  50.59 | < .001 | 80 | 0.0093 | Reasonable |
| Atraumatic Restorative Treatment | 3 | 540 | 36.49 | 20.75 – 53.85 | < .001 | 93 | 0.0214 | Reasonable |
| Slot and tunnel preparations | 4 | 721 | 35.88 | 20.85 – 52.46 | < .001 | 91 | 0.0266 | Reasonable |
| Use of magnification (e.g., loupes) for caries detection | 2 | 245 | 26.50 | 4.57 – 57.69 | <0.01 | 96 | 0.0510 | Reasonable |
| Repair defective restorations instead of replacement | 3 | 651 | 25.44 | 14.84 – 37.73 | < .001 | 91 | 0.0119 | Insufficient |
| Remineralization with CPP-ACP | 4 | 721 | 24.37 | 7.68 – 46.46 | <0.01 | 98 | 0.0536 | Insufficient |

MID, Minimal Intervention Dentistry; CPP-ACP, casein phosphopetide - amorphous calcium phosphate.

*CI 95%=confidence interval 95%; I^2^=ratio of true heterogeneity to total observed variation.

** A frequency of up to 25% was considered as ‘insufficient’ skill, between 26% and 50% as

‘reasonable’, between 51 and 75% as ‘good’, and between 76 and 100% as ‘excellent’.^16^

## **APÊNDICE 7 –** Questionário de pesquisa – estudo transversal


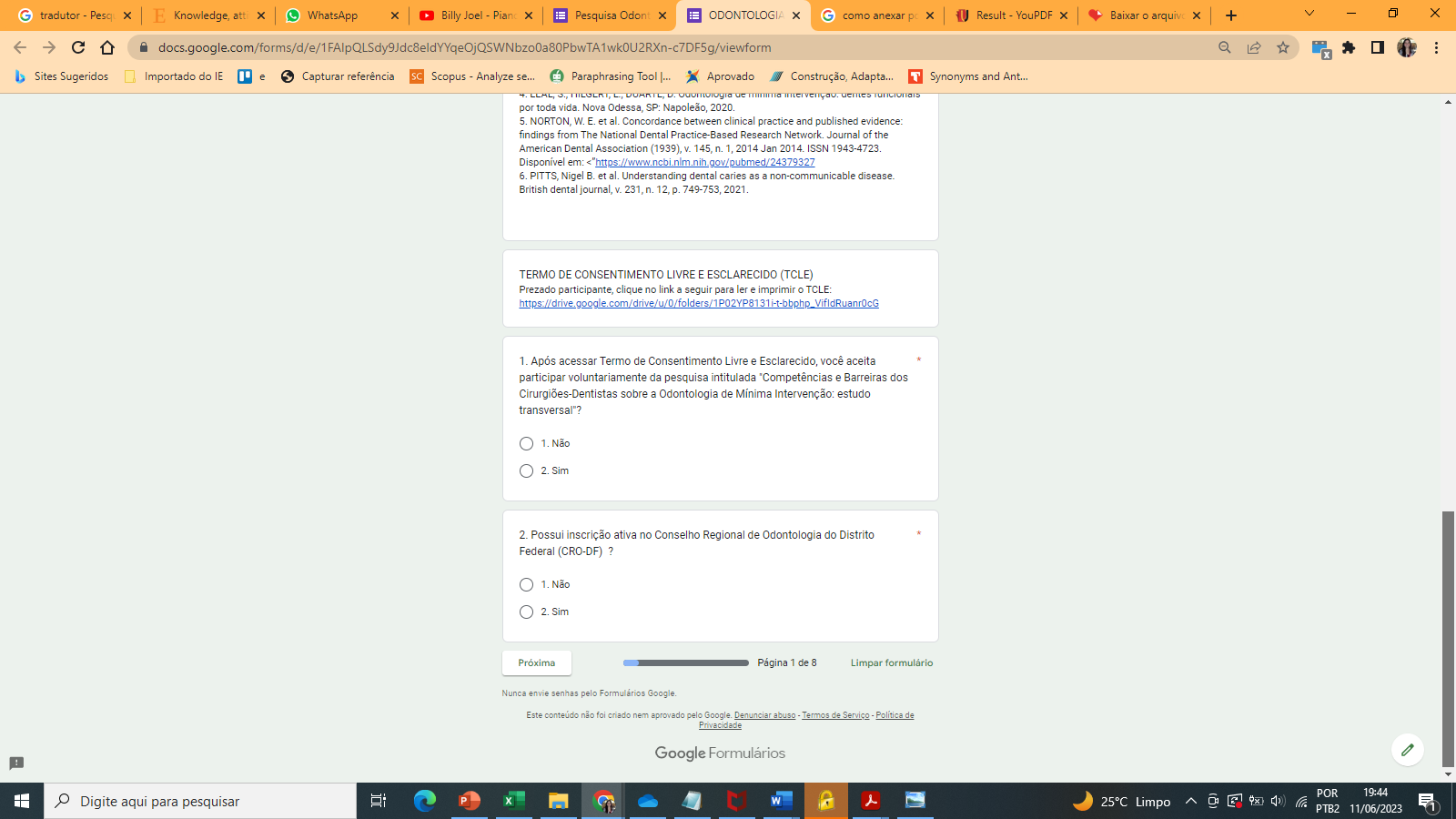


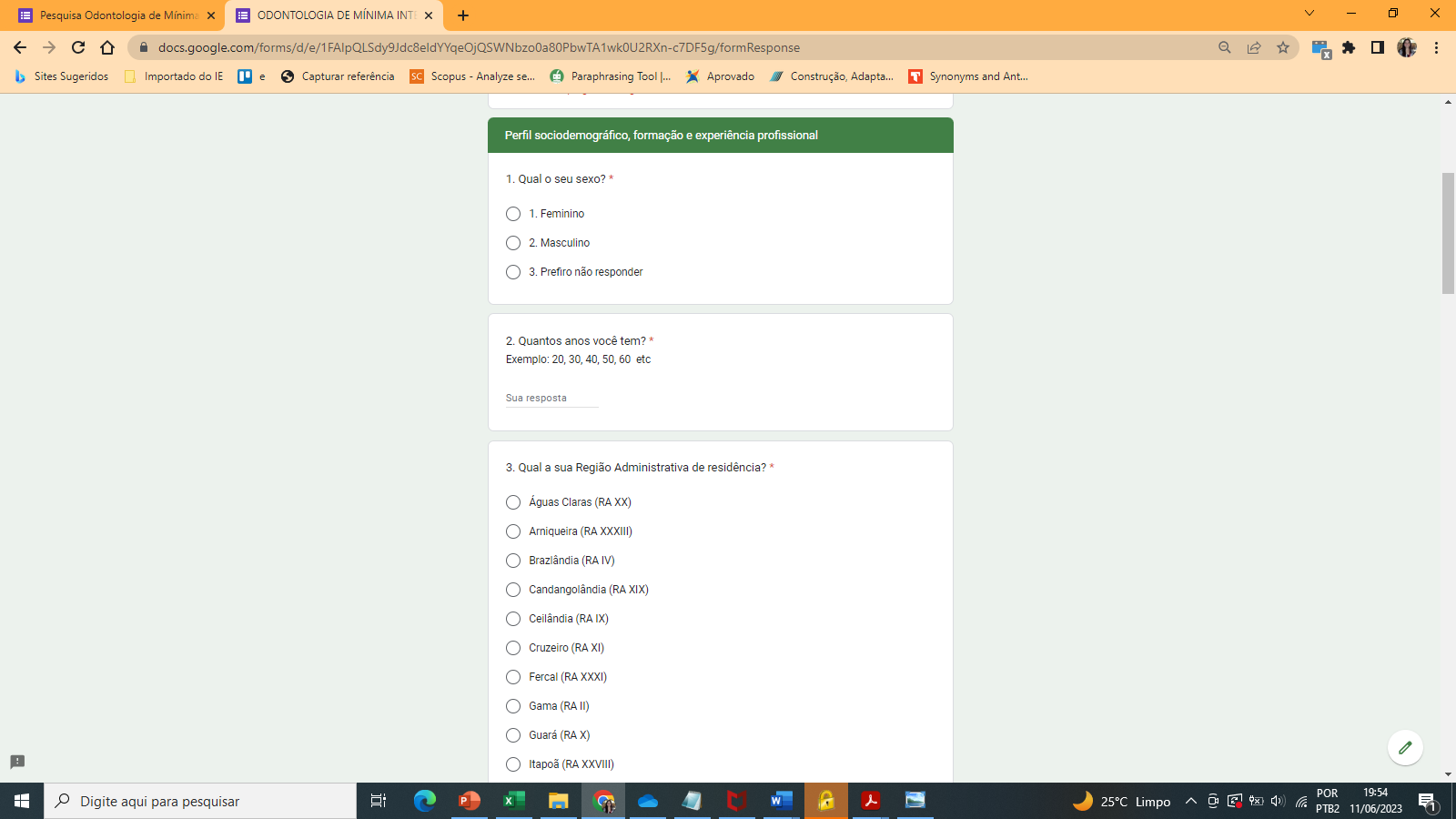


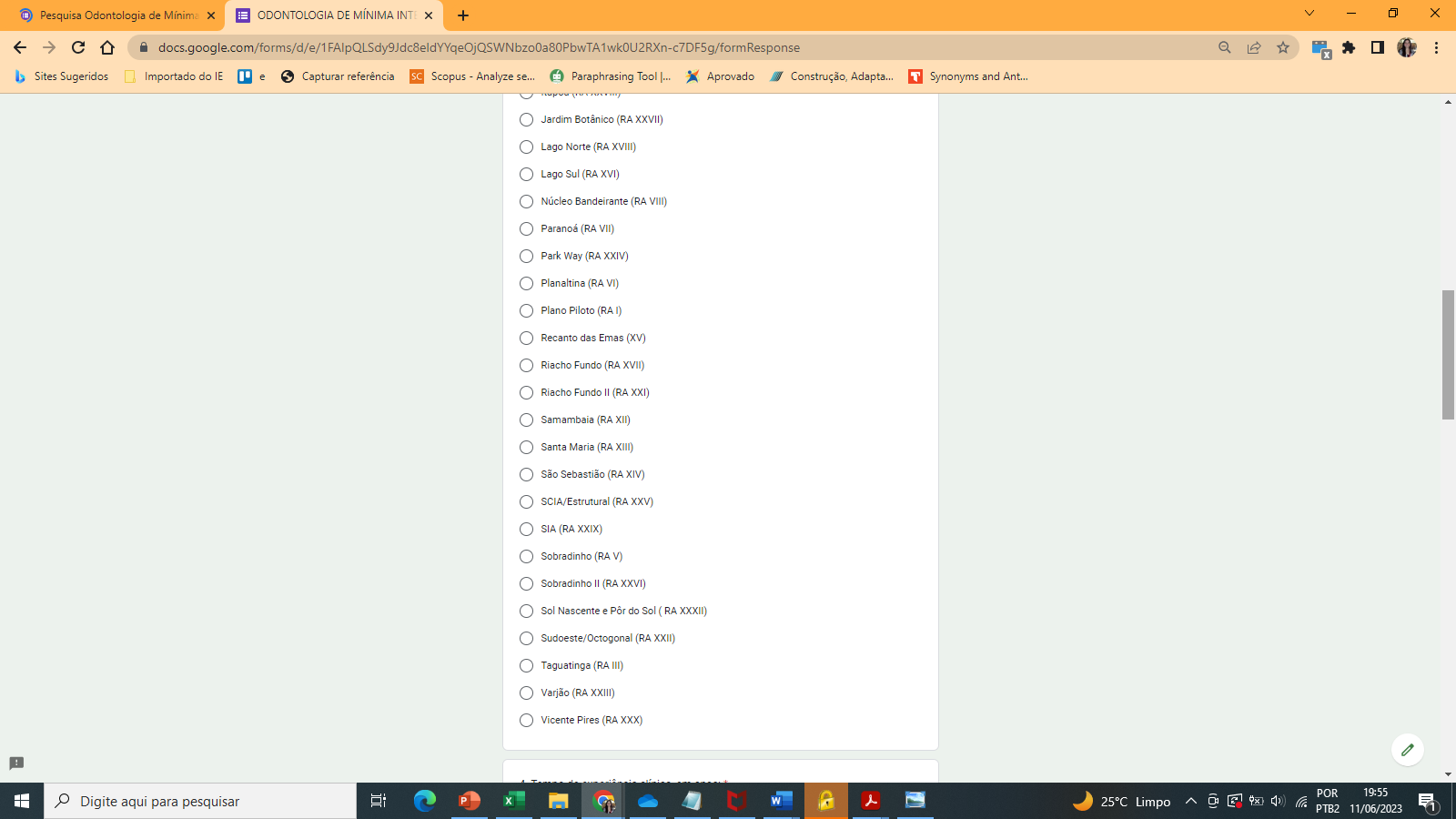


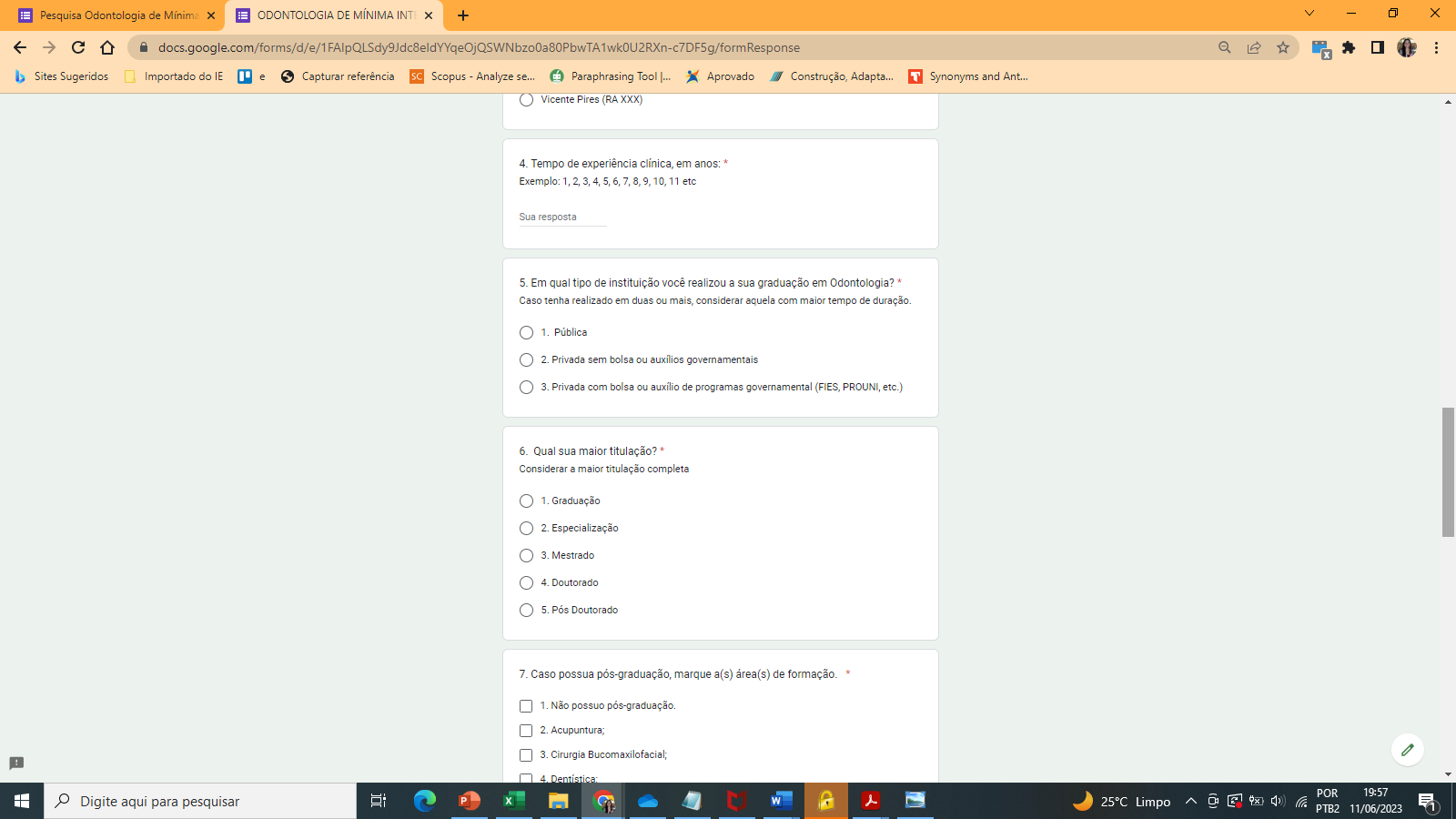


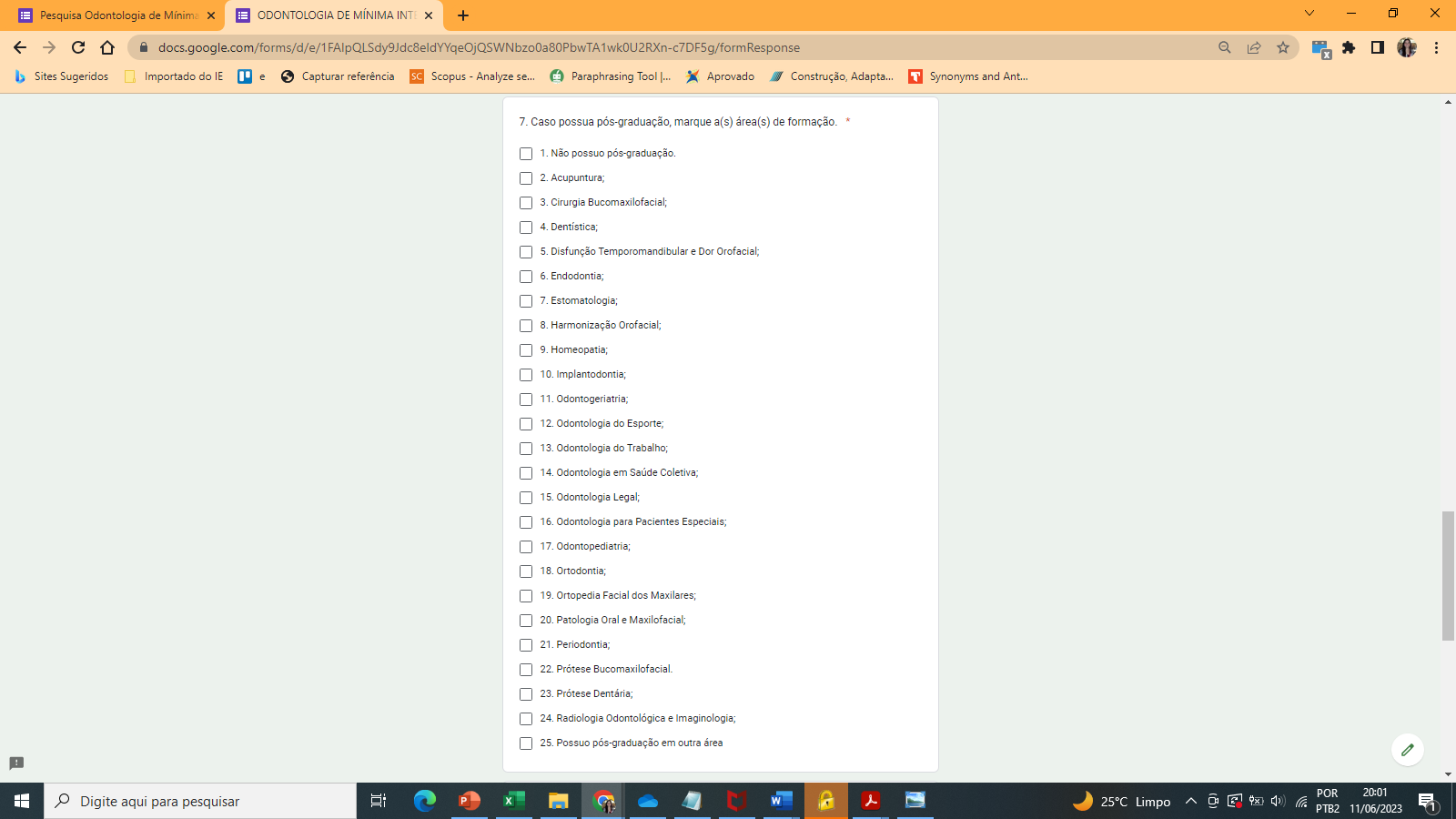


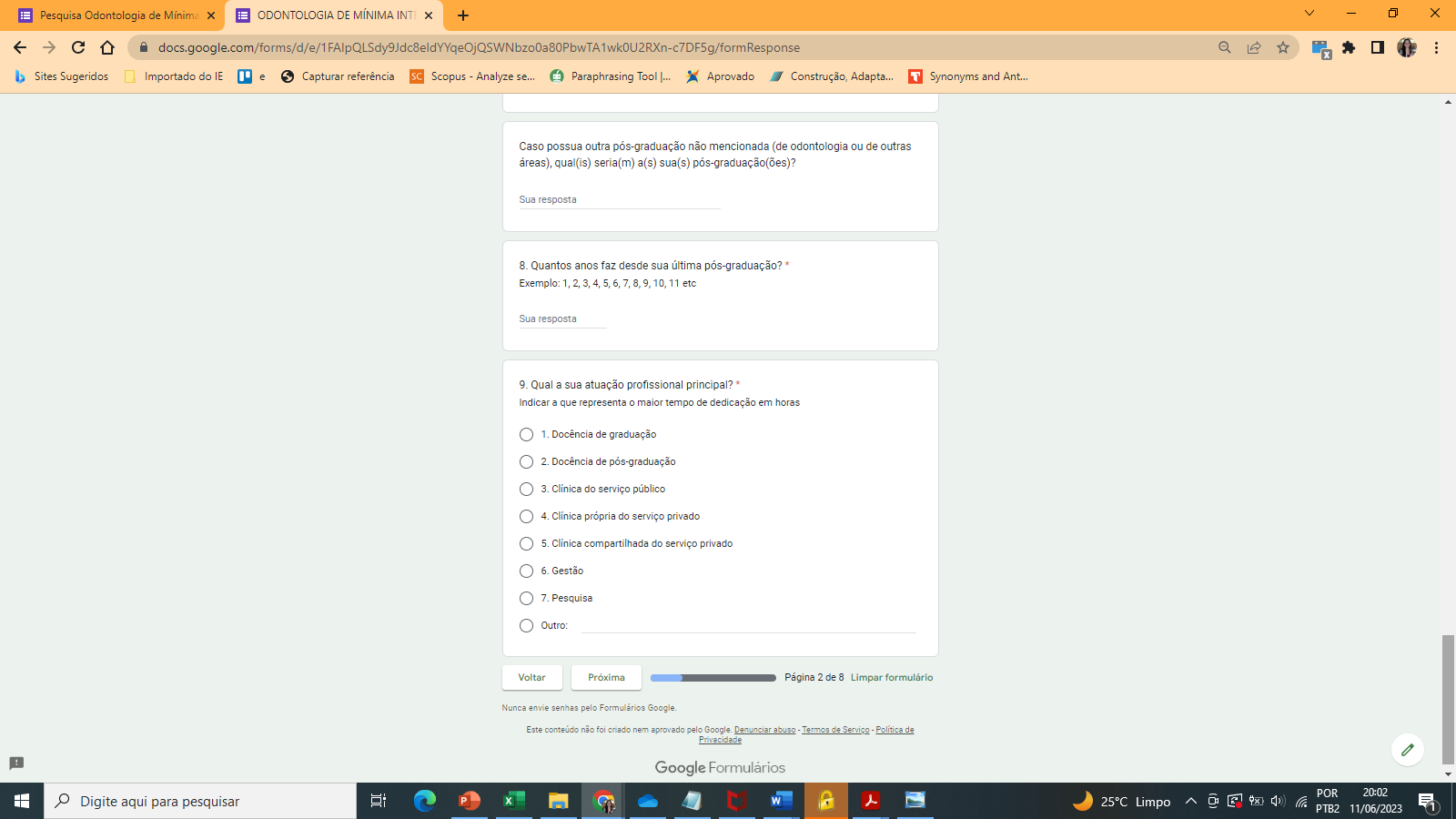


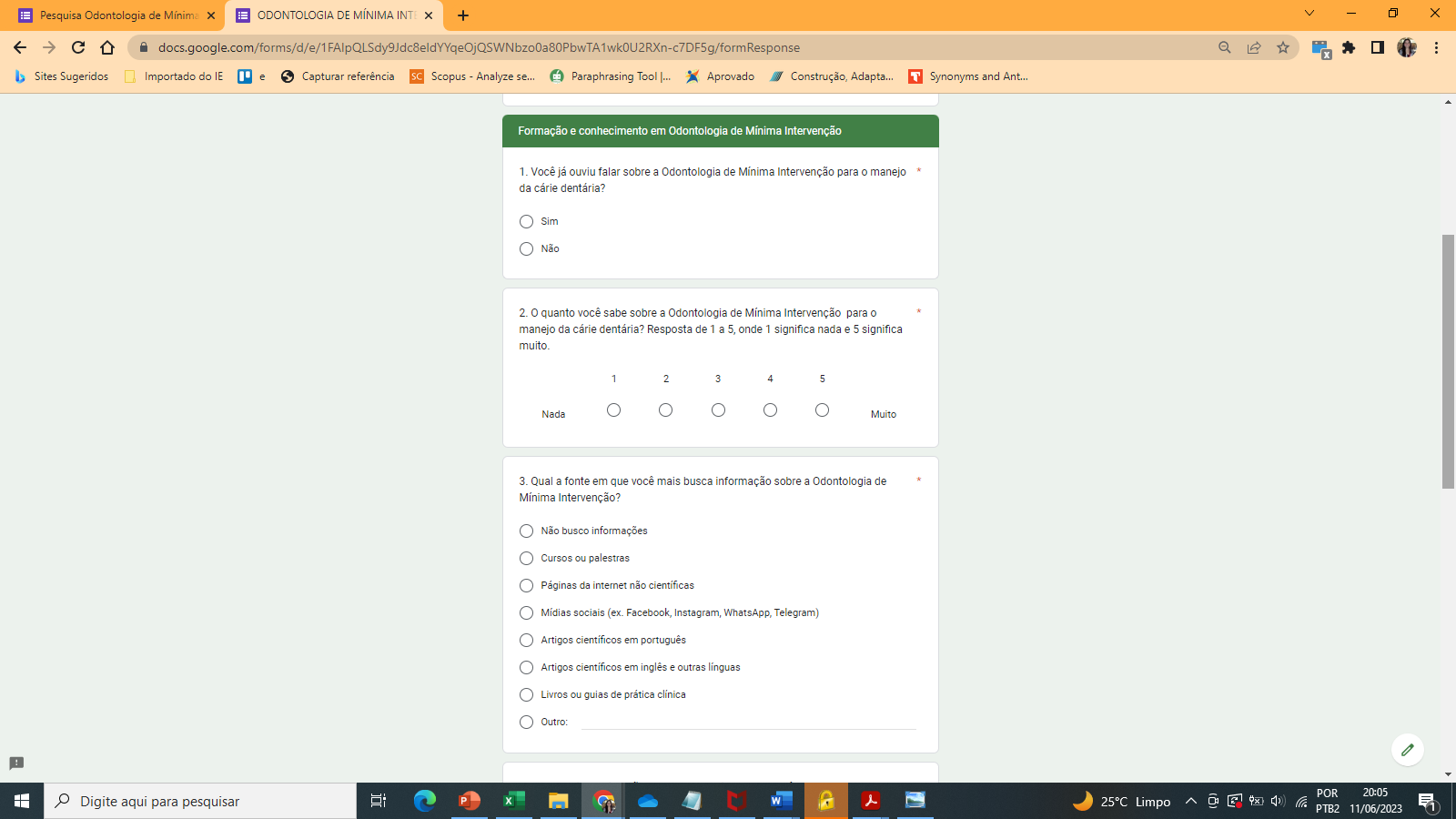


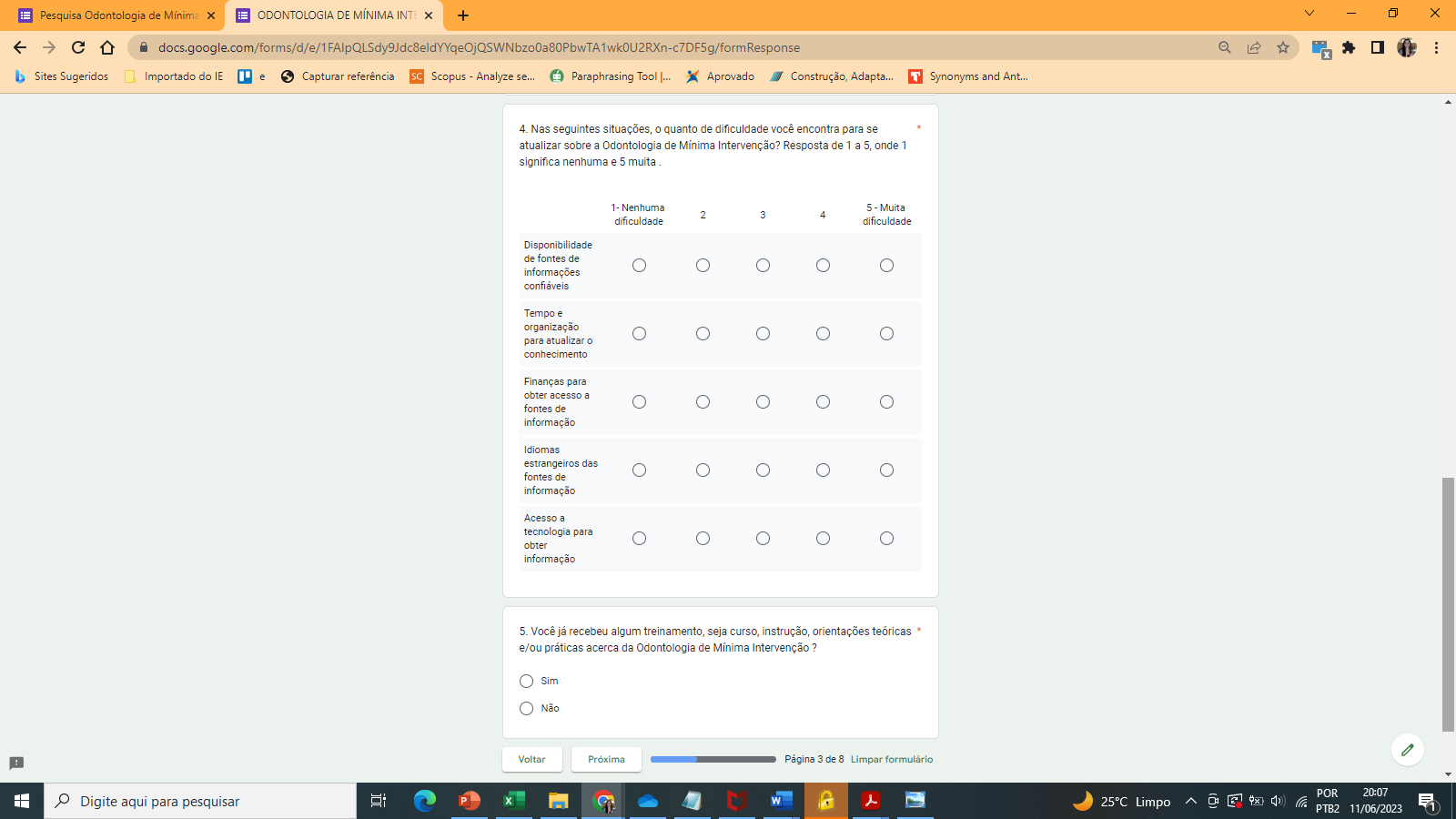


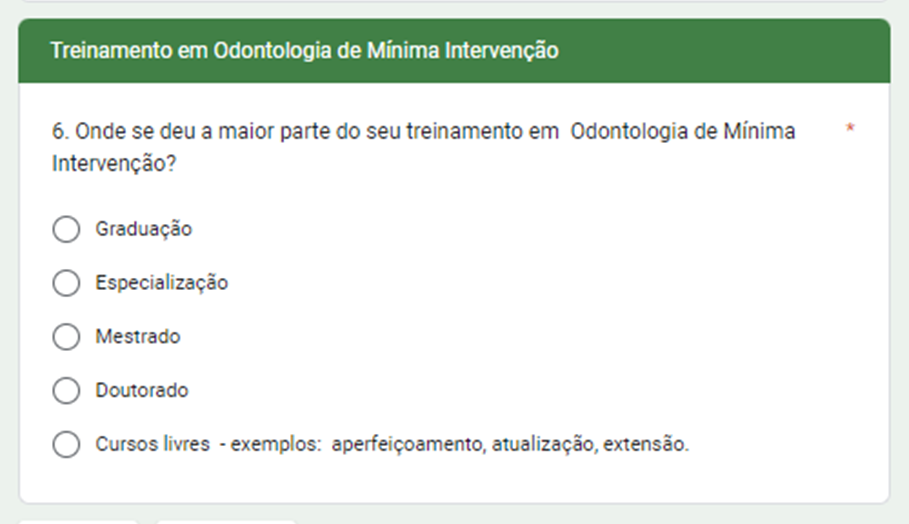


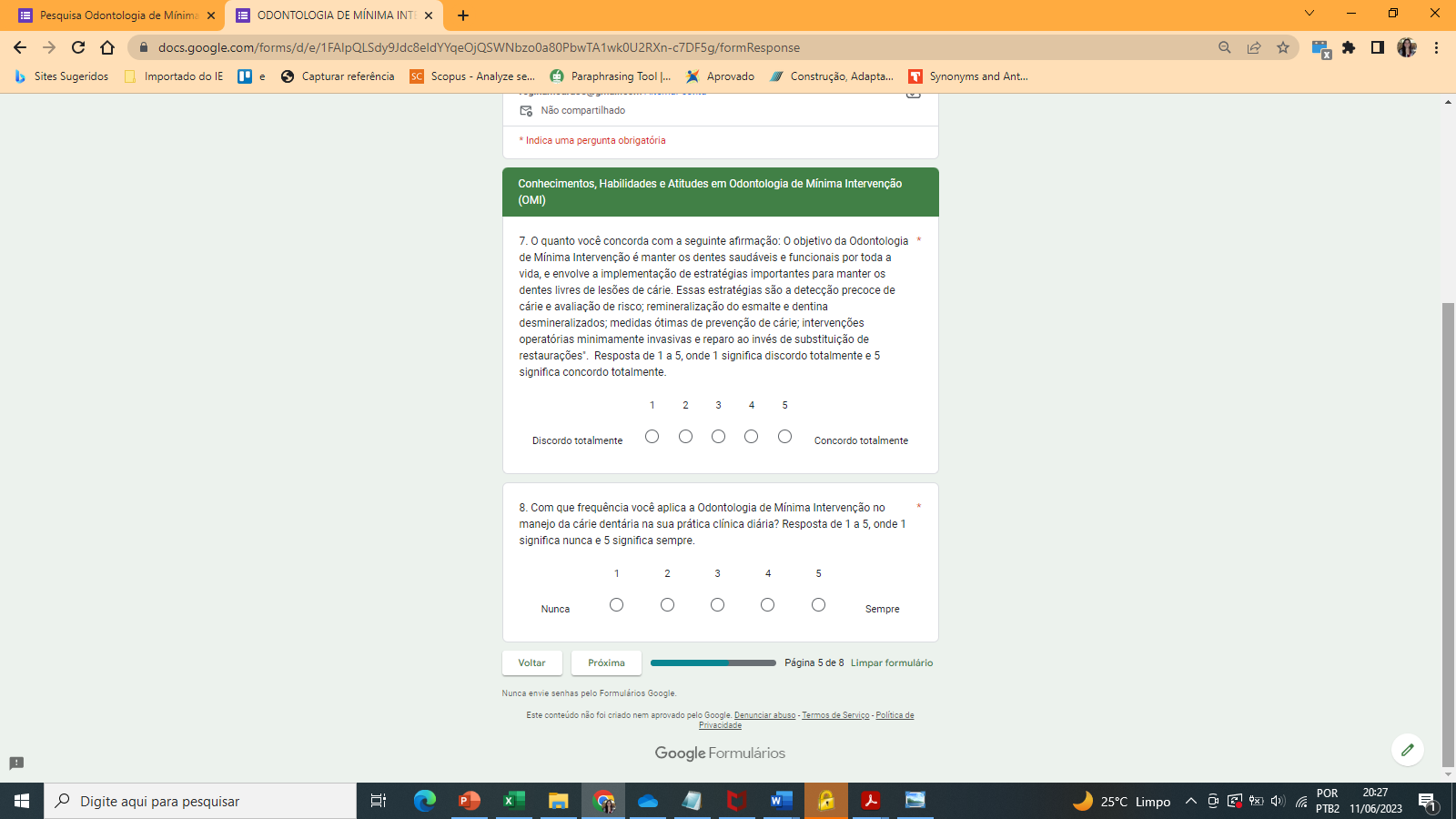


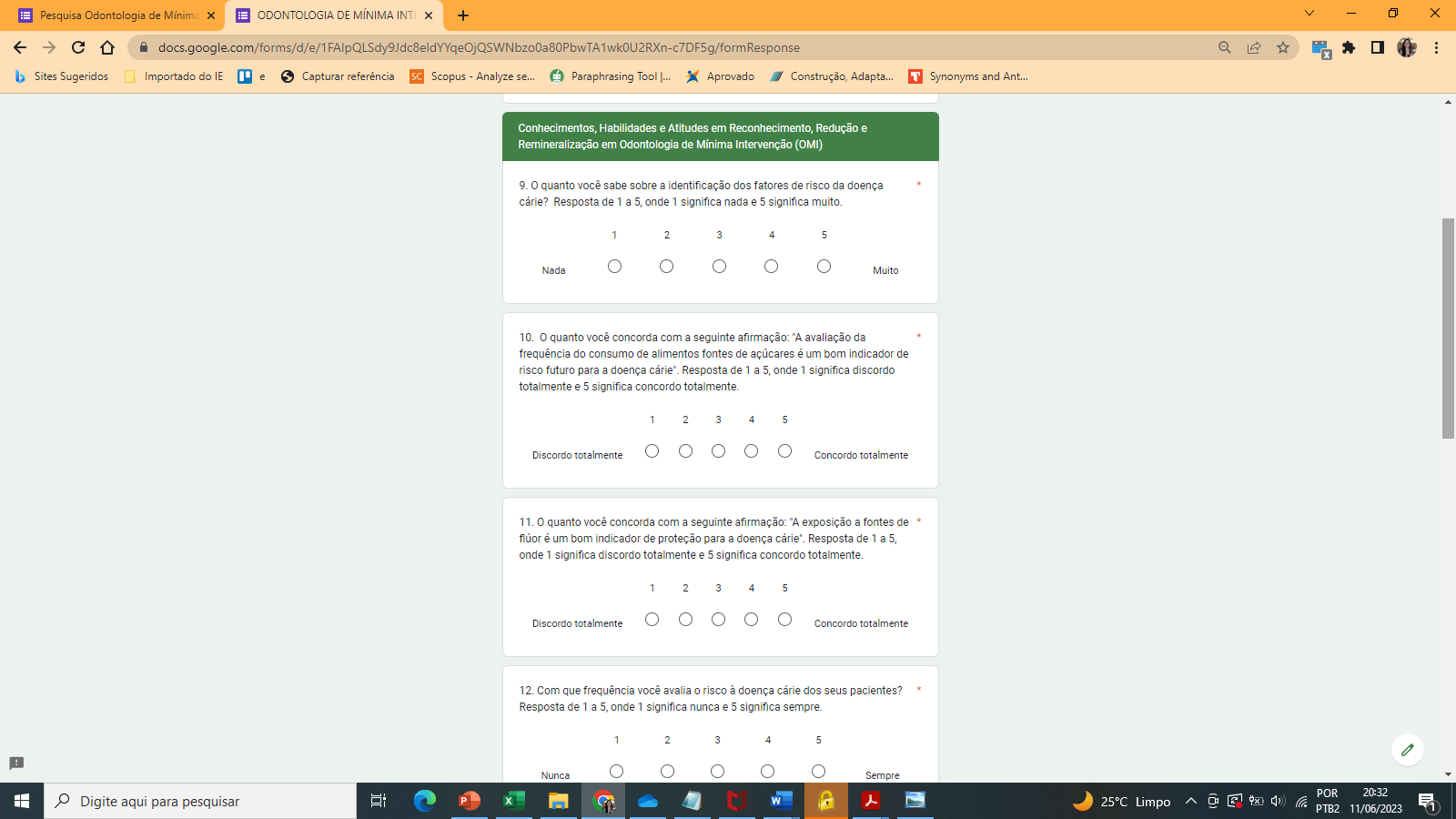


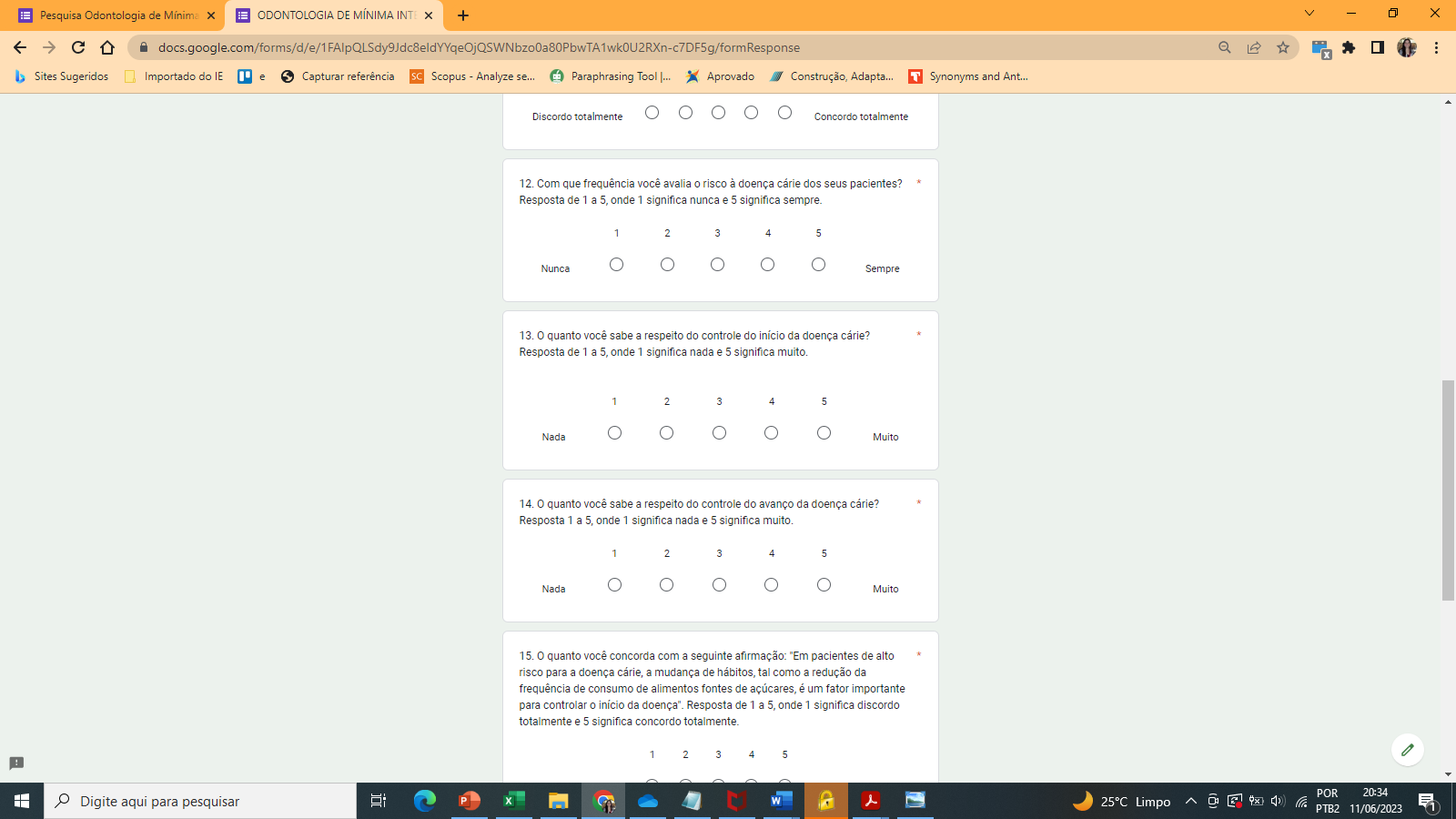


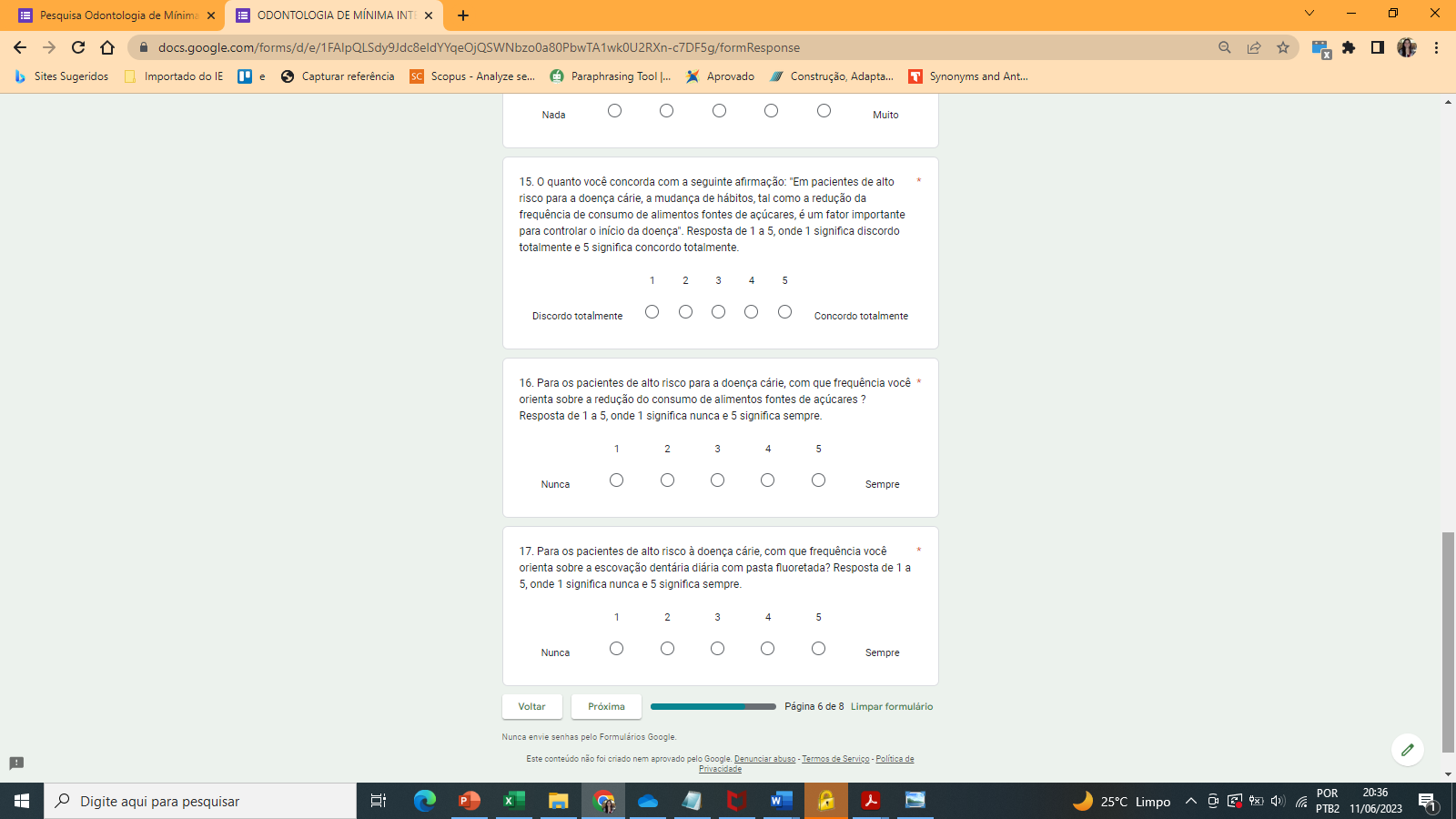


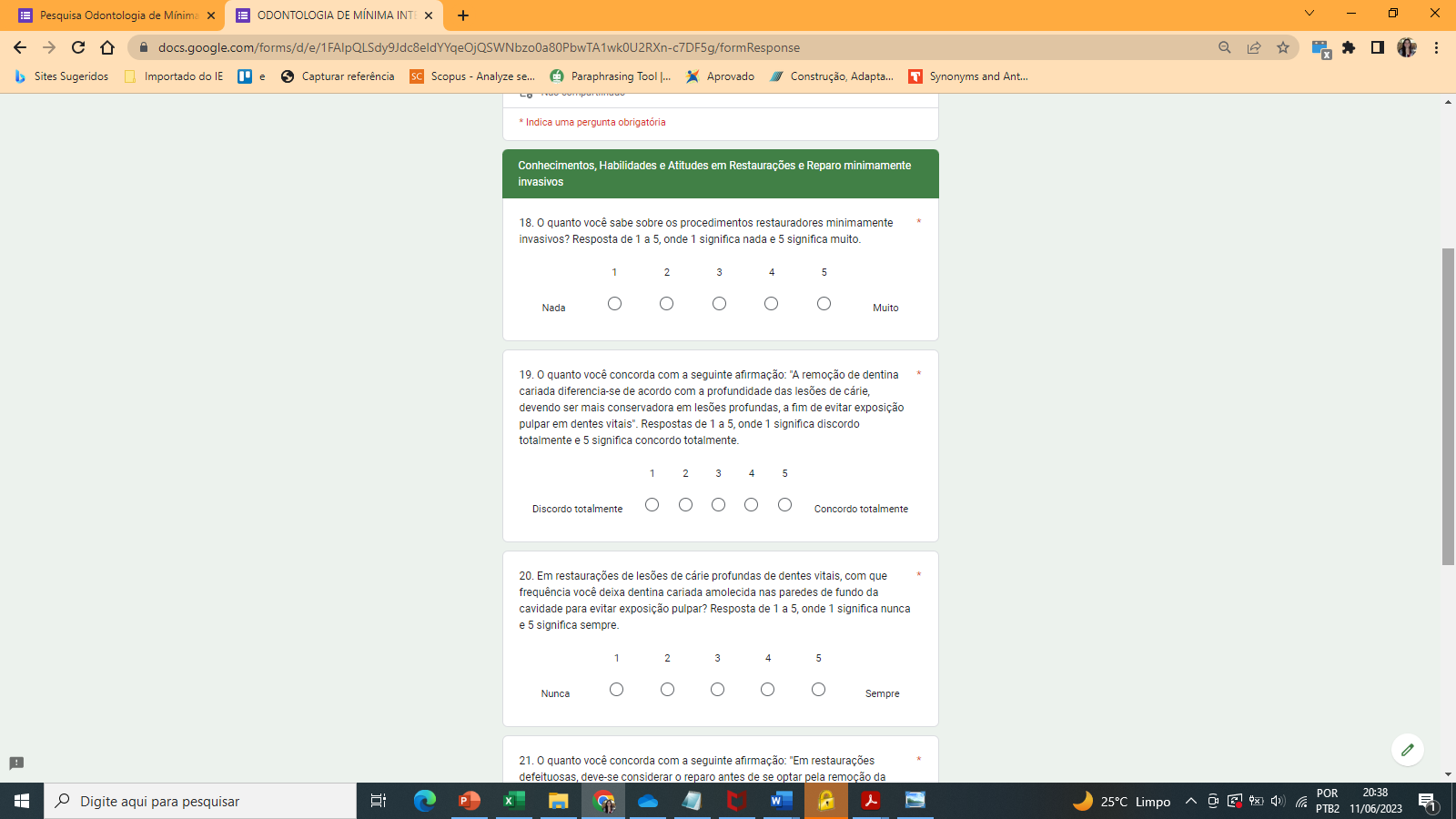


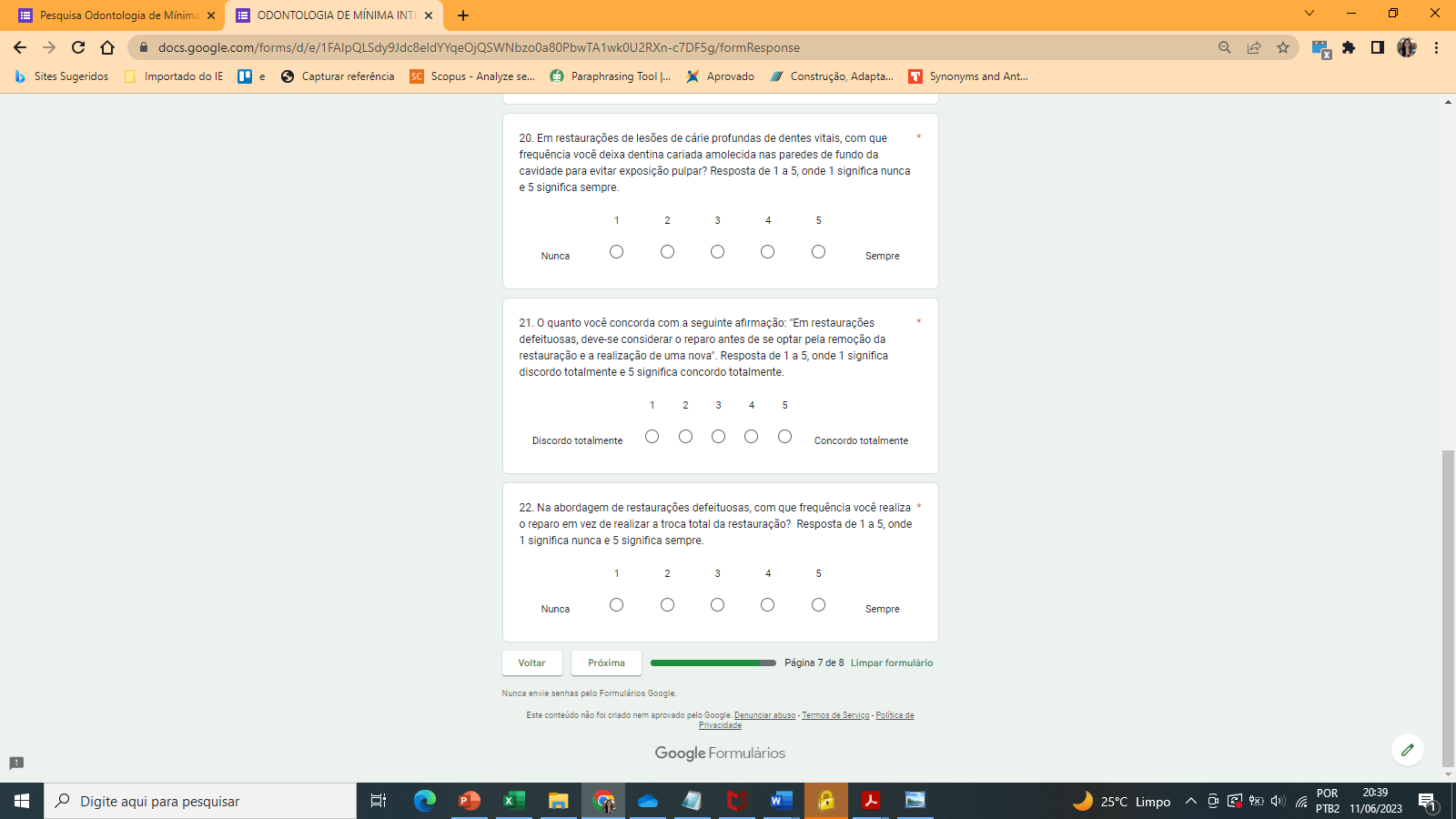


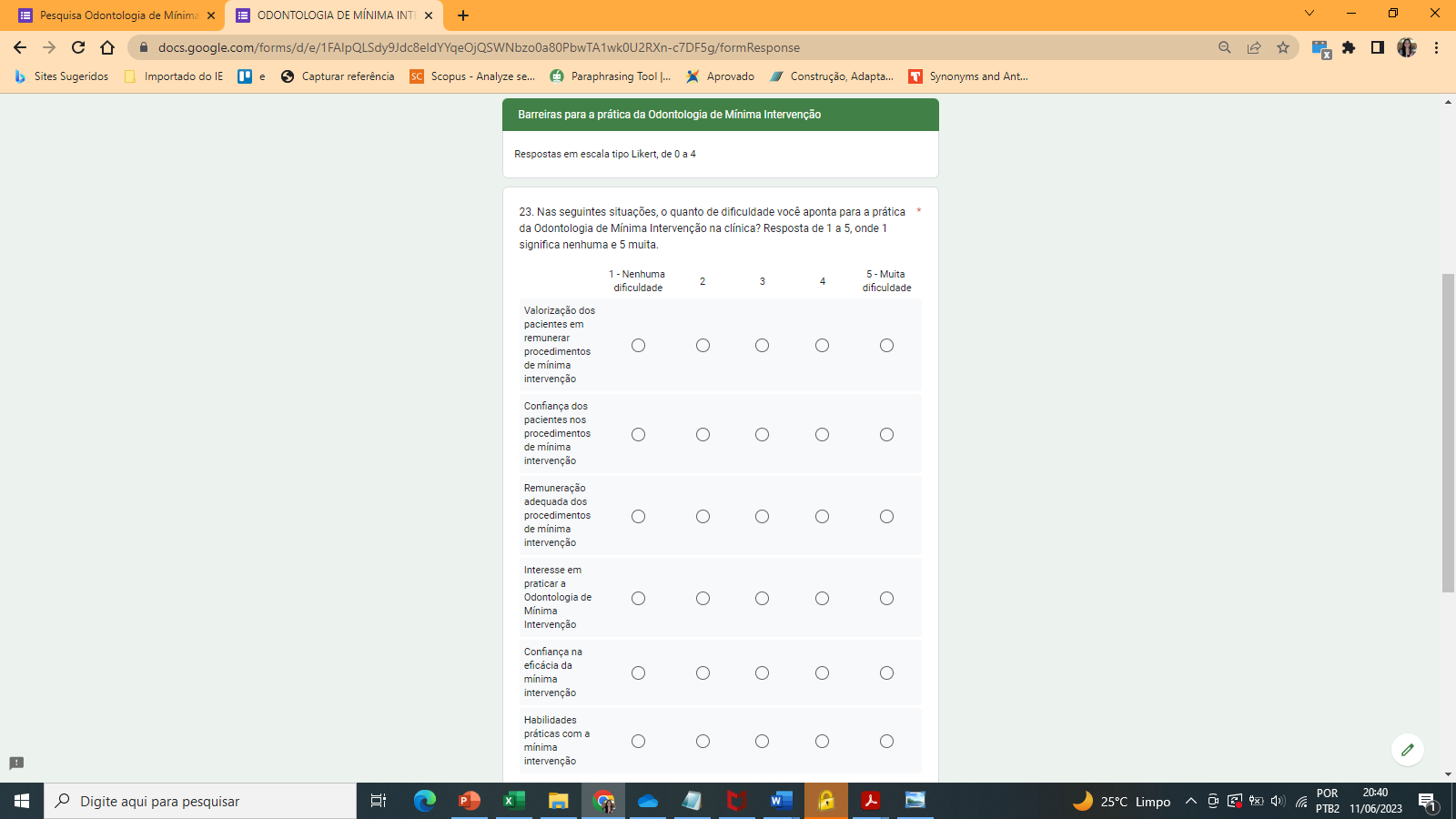


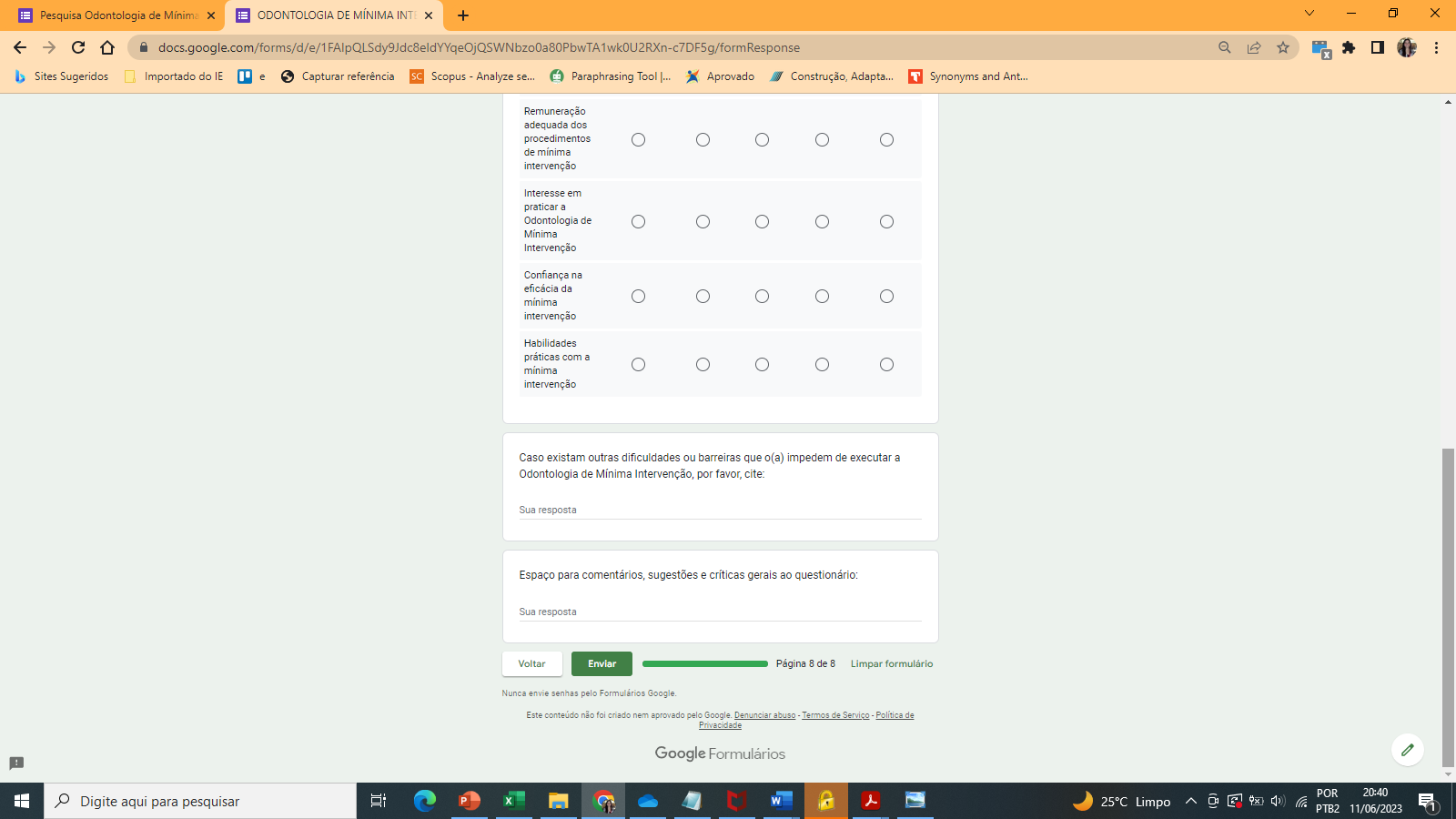


## **APÊNDICE 8** – Quadro com termos de busca de experts no assunto competências em Odontologia de Mínima Intervenção para base SCOPUS

| **Odontologia de Mínima Intervenção** | **Cirurgiões-dentistas** | **Competências** |
| --- | --- | --- |
| "Minimally Invasive Dentistry"  OR  "Minimal Intervention Dentistry"  OR  "Evidence-Based Dentistry"  OR  "Dental Caries" | "Dentists"  OR  “Dentist”  OR  "Oral Medicine"  OR  “Oral Medicine”  OR  "Dental Practitioners"  OR  "Dental Practitioner"  OR  "Oral Health-Care Worker"  OR  "Oral Health-Care Professionals" | “Health Knowledge, Attitudes, Practice"  OR  "Knowledge, Attitudes, Practice"  OR  "Health Behavior"  OR  "Health Behavior"  OR  "Health Behaviors"  OR  "Health-Related Behavior"  OR  "Health-Related Behaviors"  OR  "Health Related Behavior"  OR  "Health Related Behaviors"  OR  "Attitude Of Health Personnel"  OR  "Health Personnel Attitude"  OR  "Health Personnel Attitudes" OR  "Staff Attitude"  OR  "Staff Attitudes"  OR  "Attitude To Health”  OR  "Health Attitude"  OR  "Health Attitudes"  OR  "Beliefs"  OR  “Belief”  OR  "Evidence-practice gap"  OR "  Knowledge, Attitude, Skills"  OR  "Knowledge" |

## **APÊNDICE 9** – Checklist STROBE para o estudo “Conhecimentos, habilidades, atitudes e barreiras dos cirurgiões-dentistas do Distrito Federal sobre a Odontologia de Mínima Intervenção: estudo transversal”

|  | **Item No** | **Recommendation** |  | **Page No** |  |
| --- | --- | --- | --- | --- | --- |
| **Title and abstract** | 1 | (*a*) Indicate the study’s design with a commonly used term in the title or the abstract |  | 1 |  |
|  |  | (*b*) Provide in the abstract an informative and balanced summary of what was done and what was found |  | 1 |  |
| **Introduction** | | |  |  |  |
| Background/rationale | 2 | Explain the scientific background and rationale for the investigation being reported |  | 1,2 |  |
| Objectives | 3 | State specific objectives, including any prespecified hypotheses |  | 2 |  |
| **Methods** | | |  |  |  |
| Study design | 4 | Present key elements of study design early in the paper |  | 3 |  |
| Setting | 5 | Describe the setting, locations, and relevant dates, including periods of recruitment, exposure, follow-up, and data collection |  | 3 |  |
| Participants | 6 | (*a*) Give the eligibility criteria, and the sources and methods of selection of participants |  | 3 |  |
| Variables | 7 | Clearly define all outcomes, exposures, predictors, potential confounders, and effect modifiers. Give diagnostic criteria, if applicable |  | 3,4 |  |
| Data sources/ measurement | 8* | For each variable of interest, give sources of data and details of methods of assessment (measurement). Describe comparability of assessment methods if there is more than one group |  | 3,4 |  |
| Bias | 9 | Describe any efforts to address potential sources of bias |  | - |  |
| Study size | 10 | Explain how the study size was arrived at |  | 5 |  |
| Quantitative variables | 11 | Explain how quantitative variables were handled in the analyses. If applicable, describe which groupings were chosen and why |  | 5, 6 |  |
| Statistical methods | 12 | (*a*) Describe all statistical methods, including those used to control for confounding |  | 6 |  |
|  |  | (*b*) Describe any methods used to examine subgroups and interactions |  | - |  |
|  |  | (*c*) Explain how missing data were addressed |  | - |  |
|  |  | (*d*) If applicable, describe analytical methods taking account of sampling strategy |  | - |  |
|  |  | (*e*) Describe any sensitivity analyses |  | - |  |
| **Results** | | |  |  |  |
| Participants | 13* | (a) Report numbers of individuals at each stage of study—eg numbers potentially eligible, examined for eligibility, confirmed eligible, included in the study, completing follow-up, and analysed |  | 6 |  |
|  |  | (b) Give reasons for non-participation at each stage |  | 6 |  |
|  |  | (c) Consider use of a flow diagram |  | - |  |
| Descriptive data | 14* | (a) Give characteristics of study participants (eg demographic, clinical, social) and information on exposures and potential confounders |  | 7-10 |  |
|  |  | (b) Indicate number of participants with missing data for each variable of interest |  | 7 -10 |  |
| Outcome data | 15* | Report numbers of outcome events or summary measures |  | 10-18 |  |
| Main results | 16 | (*a*) Give unadjusted estimates and, if applicable, confounder-adjusted estimates and their precision (eg, 95% confidence interval). Make clear which confounders were adjusted for and why they were included |  | 18, 19 |  |
|  |  | (*b*) Report category boundaries when continuous variables were categorized |  | 18 |  |
|  |  | (*c*) If relevant, consider translating estimates of relative risk into absolute risk for a meaningful time period |  | - |  |
| Other analyses | 17 | Report other analyses done—eg analyses of subgroups and interactions, and sensitivity analyses |  | - |  |
| **Discussion** | | |  |  |  |
| Key results | 18 | Summarise key results with reference to study objectives |  | 19 |  |
| Limitations | 19 | Discuss limitations of the study, taking into account sources of potential bias or imprecision. Discuss both direction and magnitude of any potential bias |  | 21 |  |
| Interpretation | 20 | Give a cautious overall interpretation of results considering objectives, limitations, multiplicity of analyses, results from similar studies, and other relevant evidence |  | 21,22 |  |
| Generalisability | 21 | Discuss the generalisability (external validity) of the study results |  | 21 |  |
| **Other information** | | |  |  |  |
| Funding | 22 | Give the source of funding and the role of the funders for the present study and, if applicable, for the original study on which the present article is based |  | - |  |

## **APÊNDICE 10 –** Quadro com questões de conhecimentos, habilidades, atitudes e barreiras sobre OMI

| **ÁREA TEMÁTICA** | **CONHECIMENTO** | **HABILIDADE** | **ATITUDE** | **BARREIRAS PARA CONHECIMENTO** | **BARREIRAS PARA PRÁTICA** |
| --- | --- | --- | --- | --- | --- |
| **OMI (Geral)** | **1.** Você já ouviu falar sobre a Odontologia de Mínima Intervenção para o manejo da cárie dentária?  Resposta Sim ou Não **2.** O quanto você sabe sobre a Odontologia de Mínima Intervenção para o manejo da cárie dentária? Resposta de 1 a 5, onde 1 significa nada e 5 significa muito. | **7.** O quanto você concorda com a seguinte afirmação: O objetivo da Odontologia de Mínima Intervenção é manter os dentes saudáveis ​​e funcionais por toda a vida, e envolve a implementação de estratégias importantes para manter os dentes livres de lesões de cárie. Essas estratégias são a detecção precoce de cárie e avaliação de risco; remineralização do esmalte e dentina desmineralizados; medidas ótimas de prevenção de cárie; intervenções operatórias minimamente invasivas e reparo ao invés de substituição de restaurações".  Resposta de 1 a 5, onde 1 significa discordo totalmente e 5 significa concordo totalmente. | **8.** Com que frequência você aplica a Odontologia de Mínima Intervenção no manejo da cárie dentária na sua prática clínica diária? Resposta de 1 a 5, onde 1 significa nunca e 5 significa sempre. | **4**. Nas seguintes situações, o quanto de dificuldade você encontra para se atualizar sobre a Odontologia de Mínima Intervenção?  Resposta de 1 a 5, onde 1 significa nenhuma e 5 muita    4.1 Disponibilidade de fontes de informações confiáveis  4.2 Tempo e organização para atualizar o conhecimento  4.3 Finanças para obter acesso a fontes de informação  4.4 Idiomas estrangeiros das fontes de informação  4.5 Acesso a tecnologia para obter informação | **23.** Nas seguintes situações, o quanto de dificuldade você aponta para a prática da Odontologia de Mínima Intervenção na clínica? Resposta de 1 a 5, onde 1 significa nenhuma e 5 muita.  23.1 Valorização dos pacientes em remunerar procedimentos de mínima intervenção  23.2 Confiança dos pacientes nos procedimentos de mínima intervenção  23.3 Remuneração adequada dos procedimentos de mínima intervenção  23.4 Interesse em praticar a Odontologia de Mínima Intervenção  23.5 Confiança na eficácia da mínima intervenção  23.6 Habilidades práticas com a mínima intervenção |
| **Reconhecimento, Redução e Regeneração** | **9.** O quanto você sabe sobre a identificação dos fatores de risco da doença cárie? Resposta de 1 a 5, onde 1 significa nada e 5 significa muito.  **13.** O quanto você sabe a respeito do controle do início da doença cárie? Resposta de 1 a 5, onde 1 significa nada e 5 significa muito.  **14.** O quanto você sabe a respeito do controle do avanço da doença cárie? Resposta 1 a 5, onde 1 significa nada e 5 significa muito. | **10.** O quanto você concorda com a seguinte afirmação: "A avaliação da frequência do consumo de alimentos fontes de açúcares é um bom indicador de risco futuro para a doença cárie". Resposta de 1 a 5, onde 1 significa discordo totalmente e 5 significa concordo totalmente. **11.** O quanto você concorda com a seguinte afirmação: "A exposição a fontes de flúor é um bom indicador de proteção para a doença cárie". Resposta de 1 a 5, onde 1 significa discordo totalmente e 5 significa concordo totalmente. **15.** O quanto você concorda com a seguinte afirmação: "Em pacientes de alto risco para a doença cárie, a mudança de hábitos, tal como a redução da frequência de consumo de alimentos fontes de açúcares, é um fator importante para controlar o início da doença". Resposta de 1 a 5, onde 1 significa discordo totalmente e 5 significa concordo totalmente. | **12.** Com que frequência você avalia o risco à doença cárie dos seus pacientes? Resposta de 1 a 5, onde 1 significa nunca e 5 significa sempre.   **16.** Para os pacientes de alto risco para a doença cárie, com que frequência você orienta sobre a redução do consumo de alimentos fontes de açúcares ? Resposta de 1 a 5, onde 1 significa nunca e 5 significa sempre.   **17.** Para os pacientes de alto risco à doença cárie, com que frequência você orienta sobre a escovação dentária diária com pasta fluoretada? Resposta de 1 a 5, onde 1 significa nunca e 5 significa sempre. |  |  |
| **Restaurações minimamente invasivas e Reparo de restaurações** | **18.** O quanto você sabe sobre os procedimentos restauradores minimamente invasivos? Resposta de 1 a 5, onde 1 significa nada e 5 significa muito. | **19.** O quanto você concorda com a seguinte afirmação: "A remoção de dentina cariada diferencia-se de acordo com a profundidade das lesões de cárie, devendo ser mais conservadora em lesões profundas, a fim de evitar exposição pulpar em dentes vitais". Respostas de 1 a 5, onde 1 significa discordo totalmente e 5 significa concordo totalmente.   **21**. O quanto você concorda com a seguinte afirmação: "Em restaurações defeituosas, deve-se considerar o reparo antes de se optar pela remoção da restauração e a realização de uma nova". Resposta de 1 a 5, onde 1 significa discordo totalmente e 5 significa concordo totalmente. | **20.** Em restaurações de lesões de cárie profundas de dentes vitais, com que frequência você deixa dentina cariada amolecida nas paredes de fundo da cavidade para evitar exposição pulpar? Resposta de 1 a 5, onde 1 significa nunca e 5 significa sempre.  **22.** Na abordagem de restaurações defeituosas, com que frequência você realiza o reparo em vez de realizar a troca total da restauração? Resposta de 1 a 5, onde 1 significa nunca e 5 significa sempre. |  |  |

## **APÊNDICE 11 –** Descritivo da análise estatística completa

**Quadro 1:** Determinação das Variáveis.

| **Nome** | **Tipo da variável** | **Tratamento da variável** |
| --- | --- | --- |
| Conhecimento em OMI | Contínua (5 itens estruturados, escala Likert de 1 a 5, variando entre 5 e 25 pontos, quanto maior o escore, maior a competência avaliada) | Variável dependente (análise 1) |
| Habilidade e Atitude em OMI | Contínua (10 itens estruturados, escala Likert de 1 a 5, variando entre 10 e cinquenta pontos, quanto maior o escore, maior a competência avaliada) | Variável dependente (análise 2) |
| Barreira para conhecimento em OMI | Contínua (5 itens estruturados, escala Likert de 1 a 5, variando entre 5 e 25 pontos, quanto maior o escore, menor a competência avaliada) | Variável dependente (análise 3) |
| Barreira para prática em OMI | Contínua (6 itens estruturados, escala Likert de 1 a 5, variando entre 6 e 30 pontos, quanto maior o escore, menor a competência avaliada) | Variável dependente (análise 4) |
| Gênero | Contínua | Variável independente |
| Região Administrativa de Moradia | Categórica (Plano Piloto; Demais regiões) | Variável independente |
| Tempo de experiência (anos) | Contínua | Variável independente |
| Instituição de graduação | Categórica (Pública; Privada (com ou sem bolsa)) | Variável independente |
| Maior titulação | Categórica (Graduação; Pós-graduação (especialização, mestrado, doutorado, pós doutorado)) | Variável independente |
| Atuação profissional | Categórica (Docência (graduação, pós graduação, pesquisa); Outros) | Variável independente |
| Já ouviu falar em OMI | Categórica (Sim; Não) | Variável de ajuste |
| Busca por informações | Categórica (Sim (em qualquer meio); Não) | Variável independente |
| Receber treinamento em OMI | Categórica (Sim; Não) | Variável de ajuste |

OMI=Odontologia de mínima intervenção

**Regressão linear múltipla para avaliar os fatores que podem influenciar nos conhecimentos, habilidades e atitudes, além das barreiras em relação à OMI pelos CDs do DF**

**Passo 1:** Normalidade das variáveis dependentes (conhecimentos, habilidade e atitudes, e barreiras conhecimento, barreiras prática) e das variáveis independentes: nenhuma assume distribuição normal (p<0,001) (Teste de Shapiro-Wilk). A regressão linear múltipla foi mantida porque os resíduos dos modelos de regressão múltipla com essas variáveis apresentaram distribuição normal pela análise gráfica (FIELD, 2009). Além disso, não há violação dos outros pressupostos da regressão linear múltipla (WILLIAMS *et al.*, 2013). Foi realizada avaliação de como as variáveis estão relacionadas pela Correlação linear de Spearman.

**Passo 2:** Regressão linear simples

**Passo 3:** Regressão linear múltipla onde todas as variáveis com valor de p ≤ 20 na regressão simples foram adicionadas para o modelo de regressão múltipla além das variáveis de ajuste “Já ouviu falar em OMI” e “Receber treinamento em OMI”.

**Tabela 1:** Correlação linear entre média de conhecimento e de habilidade/atitude com as variáveis independentes (n=404).

| **Variáveis** | **Conhecimento** | | **Habilidade e Atitude** | |
| --- | --- | --- | --- | --- |
|  | **ρ** | **p valor** | **ρ** | **p valor** |
| Gênero | 0,13 | **0,004** | 0,11 | **0,01** |
| Idade (anos) | -0,12 | **0,006** | 0,11 | **0,01** |
| Região Administrativa de Moradia | 0,04 | 0,17 | 0,13 | **0,003** |
| Tempo de experiência (anos) | -0,14 | **0,002** | 0,10 | **0,02** |
| Instituição de graduação | 0,11 | **0,01** | 0,22 | **<0,001** |
| Maior titulação | -0,05 | 0,15 | 0,007 | 0,44 |
| Atuação profissional | 0,16 | **0,001** | 0,09 | **0,02** |
| Já ouviu falar em OMI | 0,23 | **<0,001** | 0,15 | **0,001** |
| Busca por informações | 0,43 | **<0,001** | 0,36 | **<0,001** |
| Receber treinamento em OMI | 0,52 | **<0,001** | 0,33 | **<0,001** |

Correlação linear de Spearman. OMI=Odontologia de mínima intervenção.

**Tabela 2:** Regressão linear múltipla para associação entre conhecimento em OMI e as variáveis preditivas (n=404).

| **Variável** | **Conhecimento em OMI** | | | |
| --- | --- | --- | --- | --- |
|  | **β IC (95%)**  **Não ajustada** | **p valor** | **β IC (95%)**  **Ajustada** | **p valor** |
| Gênero |  |  |  |  |
| Feminino | 1,02 (0,28; 1,76) | **0.007** | 0,56 (-0,04; 1,16) | 0,06 |
| Masculino |  |  |  |  |
| Idade (anos) | -0,04 (-0,07;-0,009) | **0,01** | 0,04 (-0,02; 0,11) | 0,22 |
| Região Administrativa de Moradia |  |  |  |  |
| Plano Piloto | 0,34 (-0,39; 1,07) | 0,35 |  |  |
| Outras regiões |  |  |  |  |
| Tempo de experiência (anos) | -0.04 (-0.07; -0,01) | **0,003** | -0,06 (-0,13; 0,01) | 0,09 |
| Instituição de graduação |  |  |  |  |
| Pública | 0,74 (0,08; 1,40) | **0,02** | 0,56 (0,03; 1,09) | **0,03** |
| Privada |  |  |  |  |
| Maior titulação |  |  |  |  |
| Pós-graduação | -0,49 (-1,42; 0,44) | 0,30 |  |  |
| Graduação |  |  |  |  |
| Atuação profissional |  |  |  |  |
| Docência/Pesquisa | 2,01 (0,81; 3,20) | **0.001** | 1,24 (0,27; 2,21) | **0,01** |
| Outros |  |  |  |  |
| Já ouviu falar em OMI |  |  |  |  |
| Sim | 5,06 (3,01; 7,11) | **<0,001** | 1,00 (-0,78; 2,79) | 0,27 |
| Não |  |  |  |  |
| Busca por informações |  |  |  |  |
| Sim | 3,97 (3,17; 4,76) | **<0,001** | 2,35 (1,56; 3,14) | **<0,001** |
| Não |  |  |  |  |
| Receber treinamento em OMI |  |  |  |  |
| Sim | 3,89 (3,27; 4,51) | **<0,001** | 2,95 (2,31; 3,59) | **<0,001** |
| Não |  |  |  |  |

R2 0,38; R2 ajustado 0,36. Negrito estatisticamente significativo. Todas as variáveis independentes com p ≤ 20 na regressão simples foram incluídas no modelo ajustado. As variáveis “Já ouviu falar em OMI” e “Receber treinamento em OMI” foram consideradas variáveis de ajuste. OMI=Odontologia de mínima intervenção.

A análise resultou em um modelo estatisticamente significativo [F (8, 395) = 30,24; p<0,001; R2 0,38]. Instituição de graduação (β padronizado=0,08; t=2,09; p=0,03); atuação profissional (β padronizado=0,10; t=2,51; p=0,01); busca por informações (β padronizado=0,26; t=5,85; p<0,001) e receber treinamento em OMI (β padronizado=0,39; t=9,09; p<0,001) são previsores do conhecimento em OMI.

Espera-se que haja uma média maior do conhecimento em OMI entre os CDs do DF que estudaram em instituição pública em 0,56 pontos quando comparados aos que estudaram em instituição particular (IC 95% 0,03; 1,09; p=0,03). Além disso, CDs que atuam na docência/pesquisa apresentaram em média 1,24 pontos mais em conhecimento em OMI (IC 95% 0,27; 2,21; p=0,01) quando comparados aos que trabalham em clínicas particulares, públicas e administrativo. Ainda, CDs que buscam por informação e os que receberam treinamento em OMI apresentaram resultados de conhecimento em OMI, em média, maiores (2,35; IC 95% 1,56; 3,14; p<0,001 e 2,95; IC 95% 2,31; 3,59; p<0,001; respectivamente). Os resultados indicam que este modelo pode explicar 36% da variabilidade do conhecimento em OMI.

Parâmetros da regressão linear múltipla:

- Independência dos resíduos: Teste de Durbin-Watson igual a 1,92 (aceitável entre 1,5 e 2,5); logo os resíduos são independentes
- Ausência de Multicoloneariedade:

- Fator inflacionário de variância (VIF): gênero 1,03; idade 8,07; tempo de experiência 8,69; instituição de graduação 1,02; atuação profissional 1,01; já ouviu falar em OMI 1,13; busca por informações 1,26 e recebeu treinamento em OMI 1,21 (VIF >10 podem causar problemas de multicolinearidade (correlação elevadas entre as variáveis explicativas)) (GUJARATI, 2011); logo não há multicolinearidade.

- Tolerância: gênero 0,96; idade 0,11; tempo de experiência 0,11; instituição de graduação 0,98; atuação profissional 0,98; já ouviu falar em OMI 0,88; busca por informações 0,79 e recebeu treinamento em OMI 0,82 (Valor de tolerância > 0,10 indica ausência de multicolinearidade); logo não há multicolinearidade.

- Ausência de outliers: Valores preditos padronizados (-2,95; 1,54) e resíduos padronizados (-3,06; 2,49) (valores de referência entre -3,00 e 3,00); logo não há outliers. Além disso, Distância de Cook (0,00; 0,03), valores maiores do que 1 indicam influência significativa.
- Normalidade dos resíduos: Análise gráfica (Figura 1).


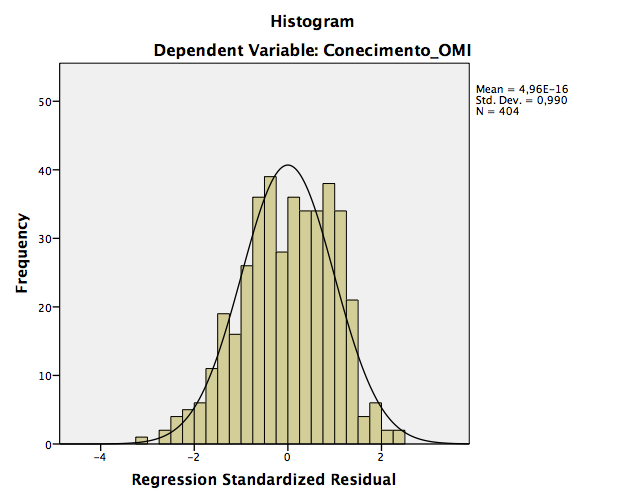


A


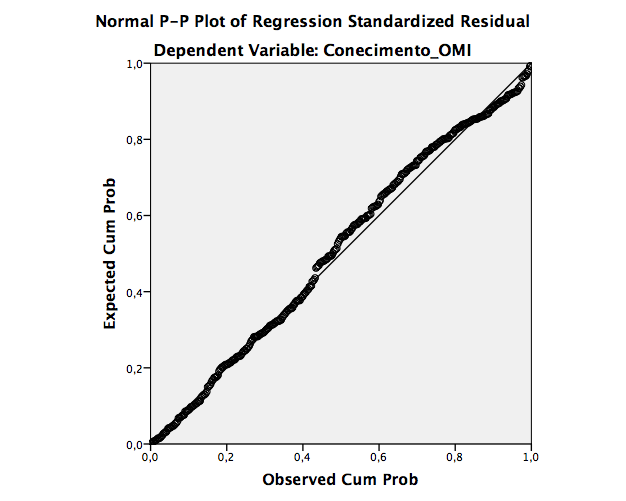


B

Figura 1 A e B. Normalidade dos resíduo da regressão linear múltipla entre conhecimento em OMI e variáveis independentes.

- Homocedasticidade: Variâncias iguais dos resíduos; análise gráfica (Figura 2).


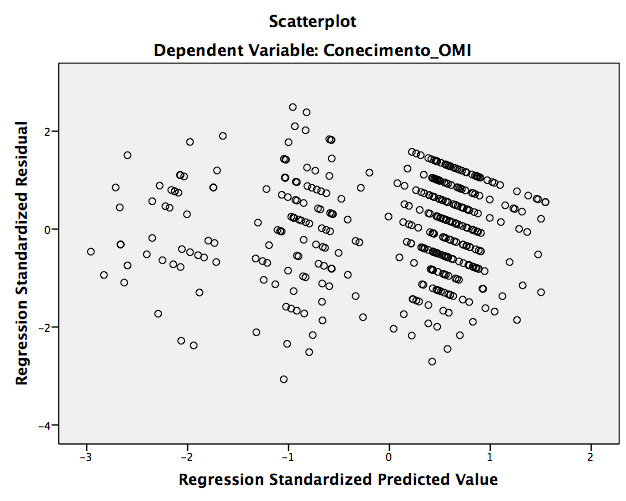


Figura 2. Homocedaticidade dos resíduo da regressão linear múltipla entre conhecimento em OMI e variáveis independentes.

**Tabela 3:** Regressão linear múltipla para associação entre habilidade e atitude em OMI e as variáveis preditivas (n=404).

| **Variável** | **Habilidade e Atitude em OMI** | | | |
| --- | --- | --- | --- | --- |
|  | **β IC (95%)**  **Não ajustada** | **p valor** | **β IC (95%)**  **Ajustada** | **p valor** |
| Gênero |  |  |  |  |
| Feminino | 1,43 (0,16; 2,70) | **0,02** | 1,22 (0,07; 2,37) | **0,03** |
| Masculino |  |  |  |  |
| Idade (anos) | 0,06 (0,01; 0,11) | **0,02** | 0,12 (-0,01; 0,26) | 0,08 |
| Região Administrativa de Moradia |  |  |  |  |
| Plano Piloto | 1,72 (0,48; 2,97) | **0,007** | 0,59 (-0,56; 1,76) | 0,31 |
| Outras regiões |  |  |  |  |
| Tempo de experiência (anos) | 0.05 (0.001; 0,10) | **0,04** | -0,04 (-0,17; 0,09) | 0,55 |
| Instituição de graduação |  |  |  |  |
| Pública | 2,57 (1,47; 3,68) | **<0,001** | 2,17 (1,15; 3,18) | **<0,001** |
| Privada |  |  |  |  |
| Maior titulação |  |  |  |  |
| Pós-graduação | 0,12 (-1,47; 1,71) | 0,88 |  |  |
| Graduação |  |  |  |  |
| Atuação profissional |  |  |  |  |
| Docência/Pesquisa | 1,99 (-0,07; 4,05) | **0,05** | 0,86 (-0,99; 2,72) | 0,36 |
| Outros |  |  |  |  |
| Já ouviu falar em OMI |  |  |  |  |
| Sim | 5,73 (2,17; 9,29) | **0,002** | 0,83 (-2,55; 4,21) | 0,62 |
| Não |  |  |  |  |
| Busca por informações |  |  |  |  |
| Sim | 3,13 (3,70; 6,56) | **<0,001** | 3,06 (1,55; 4,56) | **<0,001** |
| Não |  |  |  |  |
| Receber treinamento em OMI |  |  |  |  |
| Sim | 4,19 (3,02; 5,37) | **<0,001** | 3,40 (2,18; 4,62) | **<0,001** |
| Não |  |  |  |  |

R2 0,23; R2 ajustado 0,22. Negrito estatisticamente significativo. Todas as variáveis independentes com p ≤ 20 na regressão simples foram incluídas no modelo ajustado. As variáveis “Já ouviu falar em OMI” e “Receber treinamento em OMI” foram consideradas variáveis de ajuste. OMI=Odontologia de mínima intervenção.

A análise resultou em um modelo estatisticamente significativo [F (9, 394) = 13,74; p<0,001; R2 0,23]. Gênero (β padronizado=0,09; t=11,88; p=0,03); instituição de graduação (β padronizado=0,18; t=4,22; p<0,001); busca por informações (β padronizado=0,19; t=4,00; p<0,001) e receber treinamento em OMI (β padronizado=0,26; t=5,49; p<0,001) são previsores das habilidades e atitudes em OMI.

Espera-se que haja uma maior média na pontuação de habilidades e atitudes em OMI entre os CDs do DF do gênero feminino (1,22; IC 95% 0,07; 2,37; p=0,03) assim como entre os que estudaram em instituição pública (2,17; IC 95% 1,15; 3,18; p<0,001). Além disso, CDs que buscam por informação e os que receberam treinamento em OMI apresentaram resultados de habilidades e atitudes em OMI, em média, maiores (3,06; IC 95% 1,55; 4,56; p<0,001 e 3,40; IC 95% 2,18; 4,62; p<0,001; respectivamente). Os resultados indicam que este modelo pode explicar 22% da variabilidade de habilidades e atitudes em OMI.

Parâmetros da regressão linear múltipla:

- Independência dos resíduos: Teste de Durbin-Watson igual a 2,01 (aceitável entre 1,5 e 2,5); logo os resíduos são independentes
- Ausência de Multicoloneariedade:

- Fator inflacionário de variância (VIF): gênero 1,03; idade 8,77; região de moradia 1,10; tempo de experiência 8,74; instituição de graduação 1,02; atuação profissional 1,03; já ouviu falar em OMI 1,13; busca por informações 1,26 e recebeu treinamento em OMI 1,23 (VIF >10 podem causar problemas de multicolinearidade (correlação elevadas entre as variáveis explicativas)) (GUJARATI, 2011); logo não há multicolinearidade.

- Tolerância: gênero 0,96; idade 0,11; idade região de moradia 0,90; tempo de experiência 0,11; instituição de graduação 0,95; atuação profissional 0,96; já ouviu falar em OMI 0,88; busca por informações 0,79 e recebeu treinamento em OMI 0,80 (Valor de tolerância > 0,10 indica ausência de multicolinearidade); logo não há multicolinearidade.

- Ausência de outliers: Valores preditos padronizados (-3,04; 2,40) e resíduos padronizados (-4,50; 2,46) (valores de referência entre -3,00 e 3,00); logo há outliers. Além disso, Distância de Cook (0,00; 0,74), valores maiores do que 1 indicam influência significativa.
- Normalidade dos resíduos: Análise gráfica (Figura 3).


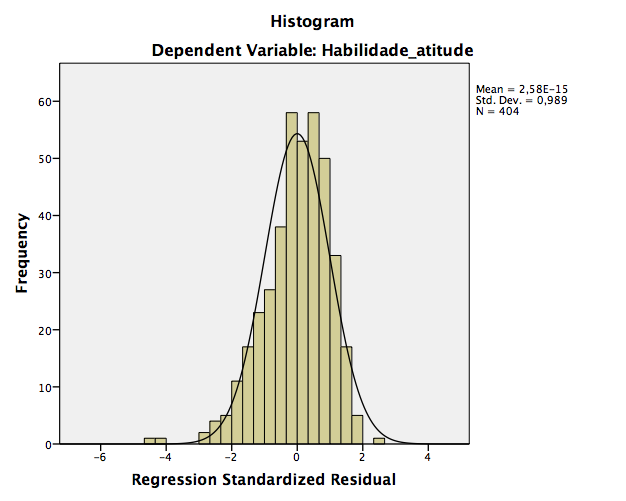


A


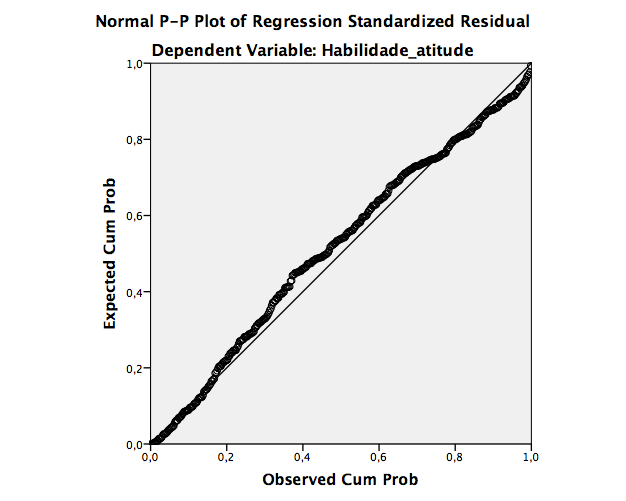


B

Figura 3 A e B. Normalidade dos resíduo da regressão linear múltipla entre habilidades e atitudes em OMI e variáveis independentes.

- Homocedasticidade: Variâncias iguais dos resíduos; análise gráfica (Figura 4).


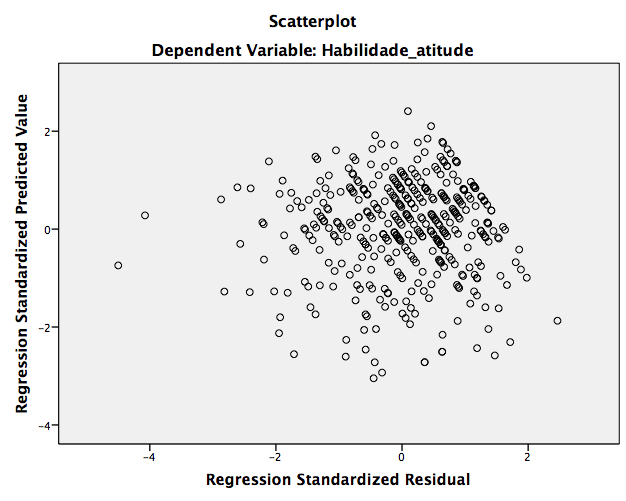


Figura 4. Homocedaticidade dos resíduo da regressão linear múltipla entre habilidades e atitudes em OMI e variáveis independentes.

**Tabela 4:** Correlação linear entre média de barreiras para conhecimento e de barreiras para prática em OMI com as variáveis independentes (n=404).

| **Variáveis** | **Barreiras Conhecimento** | | **Barreiras**  **Prática** | |
| --- | --- | --- | --- | --- |
|  | **ρ** | **p valor** | **ρ** | **p valor** |
| Gênero | 0,06 | 0,08 | -0,02 | 0,31 |
| Idade (anos) | 0,03 | 0,25 | -0,13 | **0,003** |
| Região Administrativa de Moradia | -0,01 | 0,40 | -0,09 | **0,03** |
| Tempo de experiência (anos) | 0,01 | 0,35 | -0,15 | **0,001** |
| Instituição de graduação | -0,13 | **0,003** | -0,04 | 0,21 |
| Maior titulação | -0,10 | **0,01** | -0,09 | **0,02** |
| Atuação profissional | -0,19 | **<0,001** | -0,07 | 0,08 |
| Já ouviu falar em OMI | 0,001 | 0,49 | -0,02 | 0,33 |
| Busca por informações | -0,03 | 0,25 | -0,09 | **0,02** |
| Receber treinamento em OMI | -0,16 | **<0,001** | -0,12 | **0,005** |

Correlação linear de Spearman. OMI=Odontologia de mínima intervenção.

**Tabela 5:** Regressão linear múltipla para associação entre barreiras para conhecimento em OMI e as variáveis preditivas (n=404).

| **Variável** | **Barreiras para Conhecimento em OMI** | | | |
| --- | --- | --- | --- | --- |
|  | **β IC (95%)**  **Não ajustada** | **p valor** | **β IC (95%)**  **Ajustada** | **p valor** |
| Gênero |  |  |  |  |
| Feminino | 0,59 (-0,25; 1,44) | 0,16 | 0,73 (-0,08; -1,56) | 0,08 |
| Masculino |  |  |  |  |
| Idade (anos) | 0,01 (-0,02;0,04) | 0,51 |  |  |
| Região Administrativa de Moradia |  |  |  |  |
| Plano Piloto | -0,10 (-0,93; 0,72) | 0,08 | 0,31 (-0,53; 1,15) | 0,46 |
| Outras regiões |  |  |  |  |
| Tempo de experiência (anos) | 0.007 (-0.02; 0,04) | 0,07 | 0,01 (-0,02; 0,04) | 0,54 |
| Instituição de graduação |  |  |  |  |
| Pública | -1,05 (-1,80; -0,31) | **0,005** | -0,88 (-1,61; -0,15) | **0,01** |
| Privada |  |  |  |  |
| Maior titulação |  |  |  |  |
| Pós-graduação | -1,11 (-2,17; -0,06) | **0,03** | -1,11 (-2,22; -0,003) | **0,04** |
| Graduação |  |  |  |  |
| Atuação profissional |  |  |  |  |
| Docência/Pesquisa | -2,71 (-4,06; -1,35) | **<0,001** | -2,56 (-3,90; -1,23) | **<0,001** |
| Outros |  |  |  |  |
| Já ouviu falar em OMI |  |  |  |  |
| Sim | 0,01 (-2,38; 2,41) | 0,99 | 1,10 (-1,25;1,45) | 0,36 |
| Não |  |  |  |  |
| Busca por informações |  |  |  |  |
| Sim | -0,34 (-1,34; 0,66) | 0,50 |  |  |
| Não |  |  |  |  |
| Receber treinamento em OMI |  |  |  |  |
| Sim | -1,50 (-2,32; -0,69) | **<0,001** | -1,52 (-2,35; -0,68) | **<0,001** |
| Não |  |  |  |  |

R2 0,10; R2 ajustado 0,08. Negrito estatisticamente significativo. Todas as variáveis independentes com p ≤ 20 na regressão simples foram incluídas no modelo ajustado. As variáveis “Já ouviu falar em OMI” e “Receber treinamento em OMI” foram consideradas variáveis de ajuste. OMI=Odontologia de mínima intervenção.

A análise resultou em um modelo estatisticamente significativo [F (8, 395) = 5,49; p<0,001; R2 0,08]. Instituição de graduação (β padronizado=0,11; t=-2,38; p=0,01); titulação (β padronizado=0,10; t=-1,97; p=0,04); atuação profissional (β padronizado=-0,18; t=-3,78; p<0,001) e receber treinamento em OMI (β padronizado=-0,18; t=-3,58; p<0,001) são previsores da barreiras para o conhecimento em OMI.

CDs do DF que estudaram em instituição pública tem em média menos barreiras para o conhecimentos na pontuação do questionário quando comparados aos que estudaram em instituição particular (-0,88 IC 95% -1,61; -0,15; p=0,01). O mesmo é verdadeiro para os CDs que tem pós-graduação como maior titulação, atuam na docência/pesquisa ou já receberam treinamento em OMI (-1,11 IC 95% -2,22; -0,003; p=0,04; -2,56 IC 95% -3,90; -1,23; p<0,001 e -1,52 IC 95% -2,35; -0,68; p<0,001 respectivamente) Os resultados indicam que este modelo pode explicar 8% da variabilidade das barreiras para o conhecimento em OMI.

Parâmetros da regressão linear múltipla:

- Independência dos resíduos: Teste de Durbin-Watson igual a 1,84 (aceitável entre 1,5 e 2,5); logo os resíduos são independentes
- Ausência de Multicoloneariedade:

- Fator inflacionário de variância (VIF): gênero 1,03; região de moradia 1,10; tempo de experiência 1,30; instituição de graduação 1,03; maior titulação 1,19; atuação profissional 1,02; já ouviu falar em OMI 1,05 e recebeu treinamento em OMI 1,11 (VIF >10 podem causar problemas de multicolinearidade (correlação elevadas entre as variáveis explicativas)) (GUJARATI, 2011); logo não há multicolinearidade.

- Tolerância: gênero 0,97; região de moradia 0,90; tempo de experiência 0,76; instituição de graduação 0,96; maior titulação 0,83; atuação profissional 0,97; já ouviu falar em OMI 0,94 e recebeu treinamento em OMI 0,89 (Valor de tolerância > 0,10 indica ausência de multicolinearidade); logo não há multicolinearidade.

- Ausência de outliers: Valores preditos padronizados (-2,87; 2,20) e resíduos padronizados (-1,95; 3,40) (valores de referência entre -3,00 e 3,00); logo há outliers. Além disso, Distância de Cook (0,00; 0,03), valores maiores do que 1 indicam influência significativa.
- Normalidade dos resíduos: Análise gráfica (Figura 5).


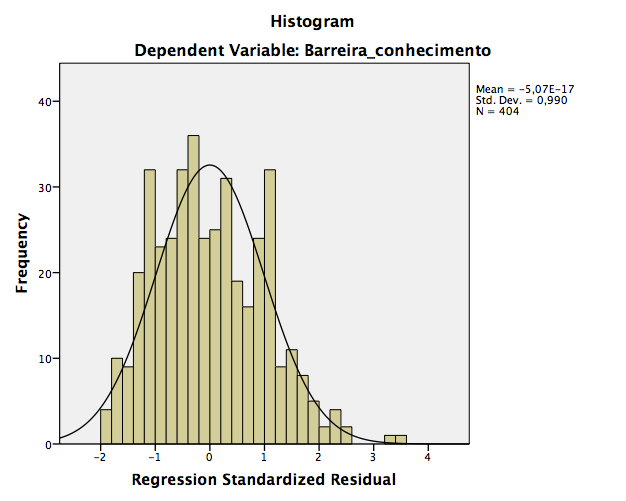


A


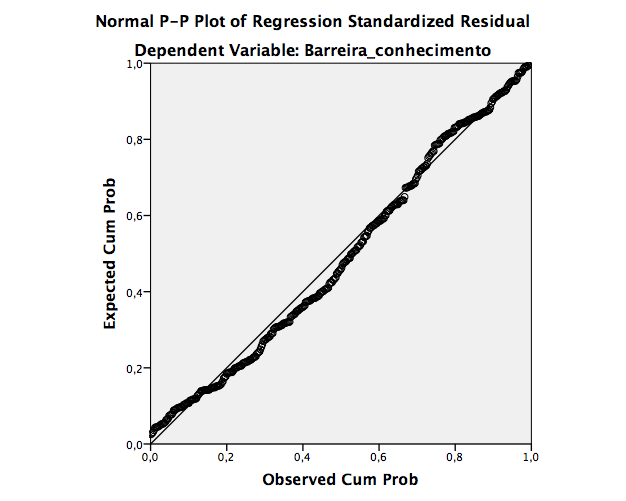


B

Figura 5 A e B. Normalidade dos resíduo da regressão linear múltipla entre conhecimento em OMI e variáveis independentes.

- Homocedasticidade: Variâncias iguais dos resíduos; análise gráfica (Figura 6).


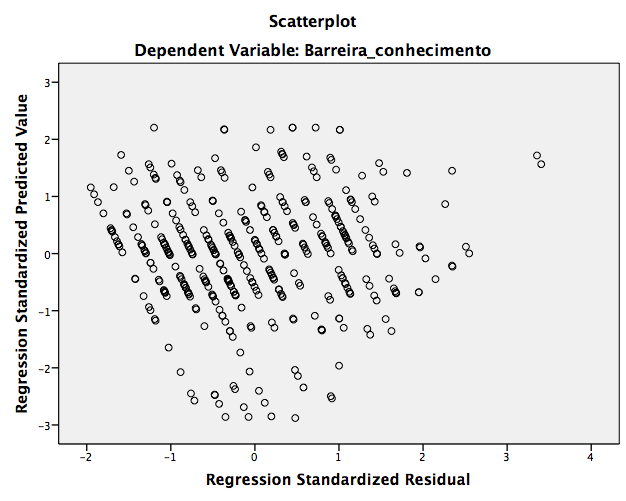


Figura 6. Homocedaticidade dos resíduo da regressão linear múltipla entre conhecimento em OMI e variáveis independentes.

**Tabela 6:** Regressão linear múltipla para associação entre barreiras para prática em OMI e as variáveis preditivas (n=404).

| **Variável** | **Barreiras para Prática em OMI** | | | |
| --- | --- | --- | --- | --- |
|  | **β IC (95%)**  **Não ajustada** | **p valor** | **β IC (95%)**  **Ajustada** | **p valor** |
| Gênero |  |  |  |  |
| Feminino | -0,32 (-1,64; 0,99) | 0,63 |  |  |
| Masculino |  |  |  |  |
| Idade (anos) | -0,07 (-0,13;-0,02) | **0,006** | -0,07 (-0,14; 0,18) | **0,01** |
| Região Administrativa de Moradia |  |  |  |  |
| Plano Piloto | -1,24 (-2,53; 0,05) | 0,06 | -0,49 (-1,82; 0,83) | 0,46 |
| Outras regiões |  |  |  |  |
| Tempo de experiência (anos) | -0,08 (-0,13; -0,02) | **0,003** |  |  |
| Instituição de graduação |  |  |  |  |
| Pública | -0,47 (-1,64; 0,69) | 0,42 |  |  |
| Privada |  |  |  |  |
| Maior titulação |  |  |  |  |
| Pós-graduação | -1,63 (-3,27; 0,01) | **0,05** | -0,85 (-2,57; 0,87) | 0,33 |
| Graduação |  |  |  |  |
| Atuação profissional |  |  |  |  |
| Docência/Pesquisa | -1,52 (-3,66; 0,61) | 0,16 | -1,17 (-3,31; 0,96) | 0,28 |
| Outros |  |  |  |  |
| Já ouviu falar em OMI |  |  |  |  |
| Sim | -0,81 (-4,53; 2,91) | 0,66 | 0,68 (-3,20; 4,57) | 0,72 |
| Não |  |  |  |  |
| Busca por informações |  |  |  |  |
| Sim | -1,53 (-3,09; 0,02) | **0,05** | -0,67 (-2,40; 1,04) | 0,43 |
| Não |  |  |  |  |
| Receber treinamento em OMI |  |  |  |  |
| Sim | -1,67 (-2,94; -0,39) | **0,01** | -1,84 (-3,25; -0,44) | **0,01** |
| Não |  |  |  |  |

R2 0,05; R2 ajustado 0,03. Negrito estatisticamente significativo. Todas as variáveis independentes com p ≤ 20 na regressão simples foram incluídas no modelo ajustado. As variáveis “Já ouviu falar em OMI” e “Receber treinamento em OMI” foram consideradas variáveis de ajuste. OMI=Odontologia de mínima intervenção.

A análise resultou em um modelo estatisticamente significativo [F (8, 395) = 2,91; p<0,001; R2 0,03]. Idade (β padronizado=-0,14; t=-2,56; p=0,01) e receber treinamento em OMI (β padronizado=-0,14; t=-2,58; p=0,01) são previsores das barreiras para a prática em OMI.

Quanto mais jovem o CD, maiores as pontuações em média em barreiras para a prática (-0,07 IC 95% -0,14; 0,18; p=0,01). CDs do DF que já receberam treinamento em OMI tem em média menos barreiras para a prática na pontuação do questionário (-1,84 IC 95% -3,25; -0,44; p=0,01). Os resultados indicam que este modelo pode explicar 3% da variabilidade das barreiras para a preatica em OMI (o modelo é pouco explicativo).

Parâmetros da regressão linear múltipla:

- Independência dos resíduos: Teste de Durbin-Watson igual a 1,93 (aceitável entre 1,5 e 2,5); logo os resíduos são independentes
- Ausência de Multicoloneariedade:

- Fator inflacionário de variância (VIF): idade 1,24; região de moradia 1,09; maior titulação 1,12; atuação profissional 1,03; já ouviu falar em OMI 1,13, busca por informação 1,25 e recebeu treinamento em OMI 1,23 (VIF >10 podem causar problemas de multicolinearidade (correlação elevadas entre as variáveis explicativas)) (GUJARATI, 2011); logo não há multicolinearidade.

- Tolerância: idade 0,80; região de moradia 0,91; maior titulação 0,88; atuação profissional 0,96; já ouviu falar em OMI 0,88, busca por informação 0,79 e recebeu treinamento em OMI 0,80 (Valor de tolerância > 0,10 indica ausência de multicolinearidade); logo não há multicolinearidade. Tempo de experiência havia apresentado tolerância de 0,10 e foi removido do modelo.

- Ausência de outliers: Valores preditos padronizados (-3,13; 3,02) e resíduos padronizados (-1,99; 2,52) (valores de referência entre -3,00 e 3,00); logo há outliers. Além disso, Distância de Cook (0,00; 0,06), valores maiores do que 1 indicam influência significativa.
- Normalidade dos resíduos: Análise gráfica (Figura 7).


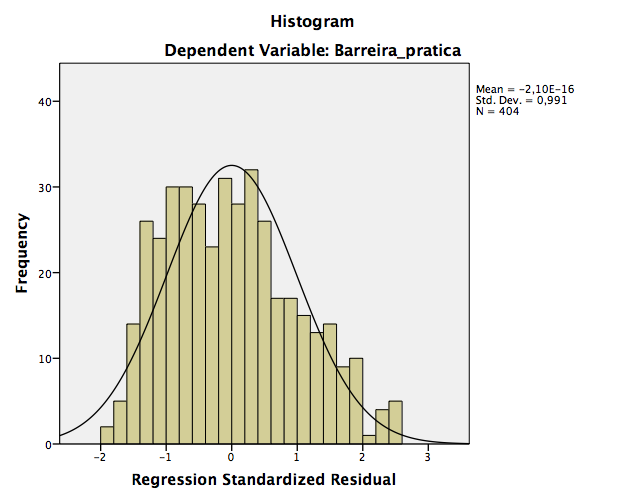


A


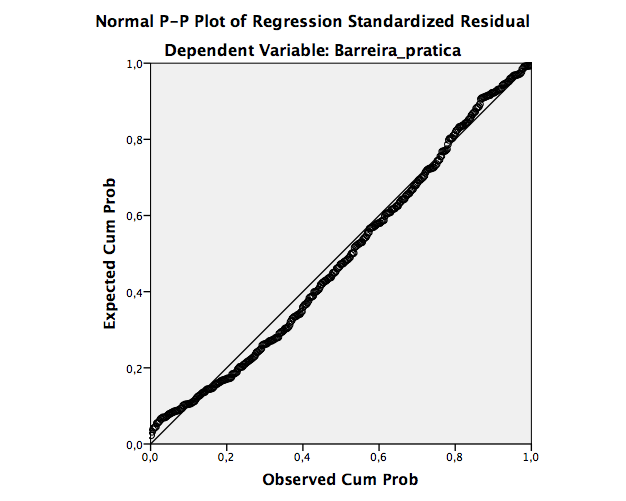


B

Figura 7 A e B. Normalidade dos resíduo da regressão linear múltipla entre conhecimento em OMI e variáveis independentes.

- Homocedasticidade: Variâncias iguais dos resíduos; análise gráfica (Figura 8).


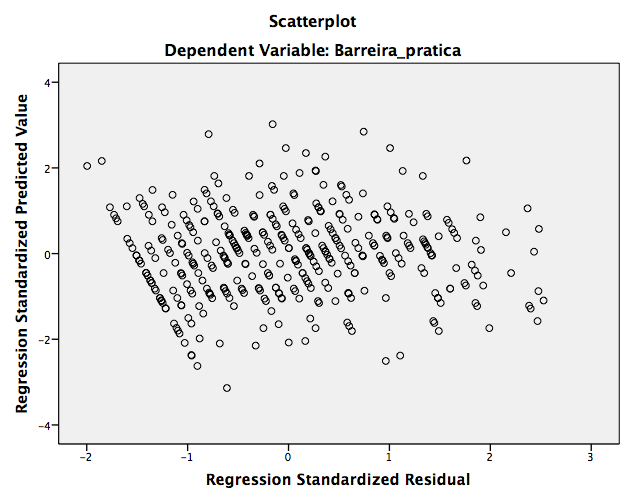


Figura 8. Homocedaticidade dos resíduo da regressão linear múltipla entre conhecimento em OMI e variáveis independentes.

**REFERÊNCIAS DA ANÁLISE ESTATÍSTICA**

Field, A. Descobrindo a estatística usando o SPSS-5. Penso Editora, 2009.

Gujarati, DN; Porter, DC. Econometria Básica-5. Amgh Editora, 2011.

Williams, MN; Grajales, CAG; Kurkiewicz, D. Assumptions of multiple regression: Correcting two misconceptions. 2013. ISSN 1531-7714.

# ANEXO

## **ANEXO 1 –** Parecer Consubstanciado do Comitê De Ética em Pesquisa (CEP/FS/UnB)


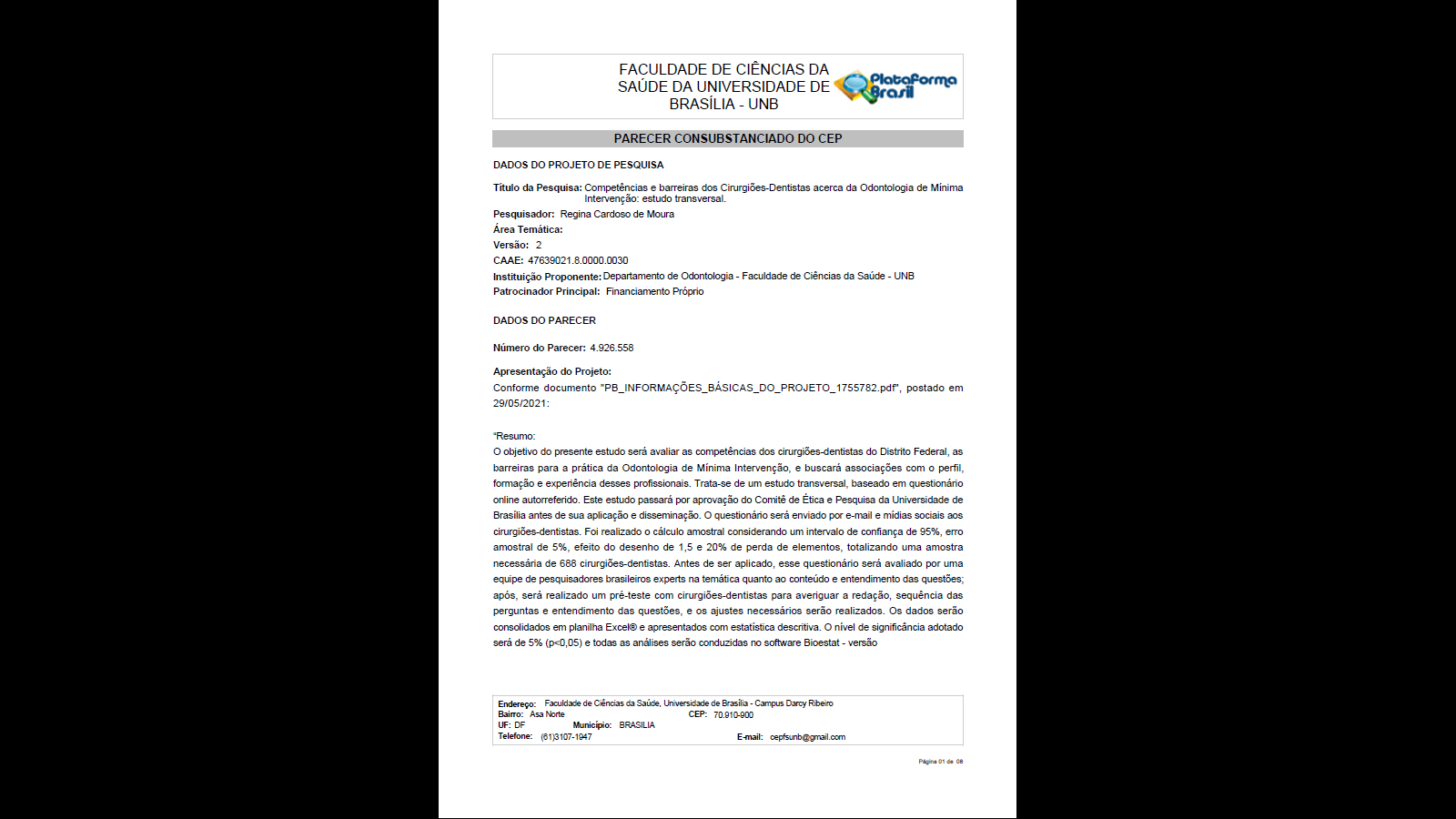


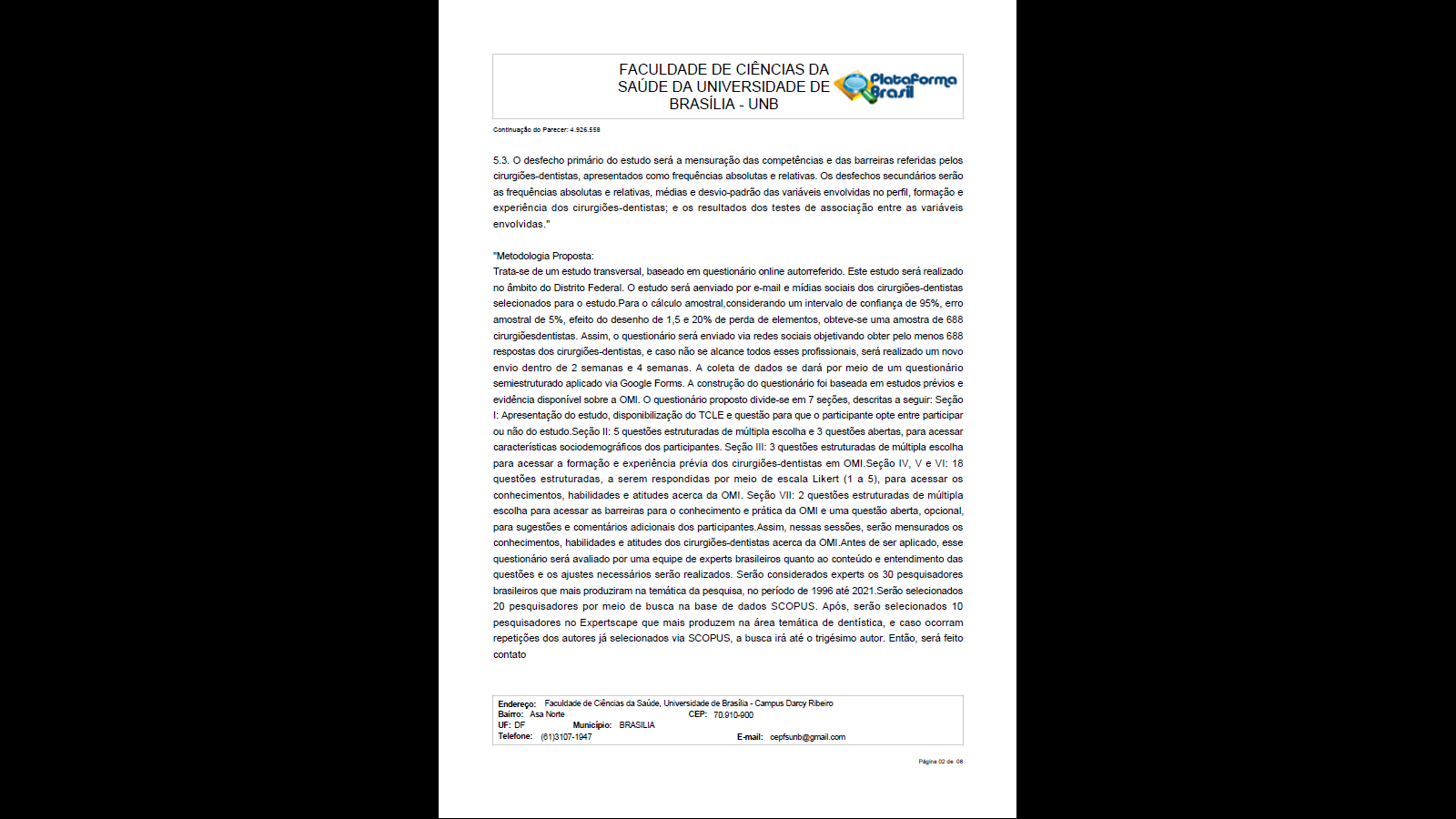


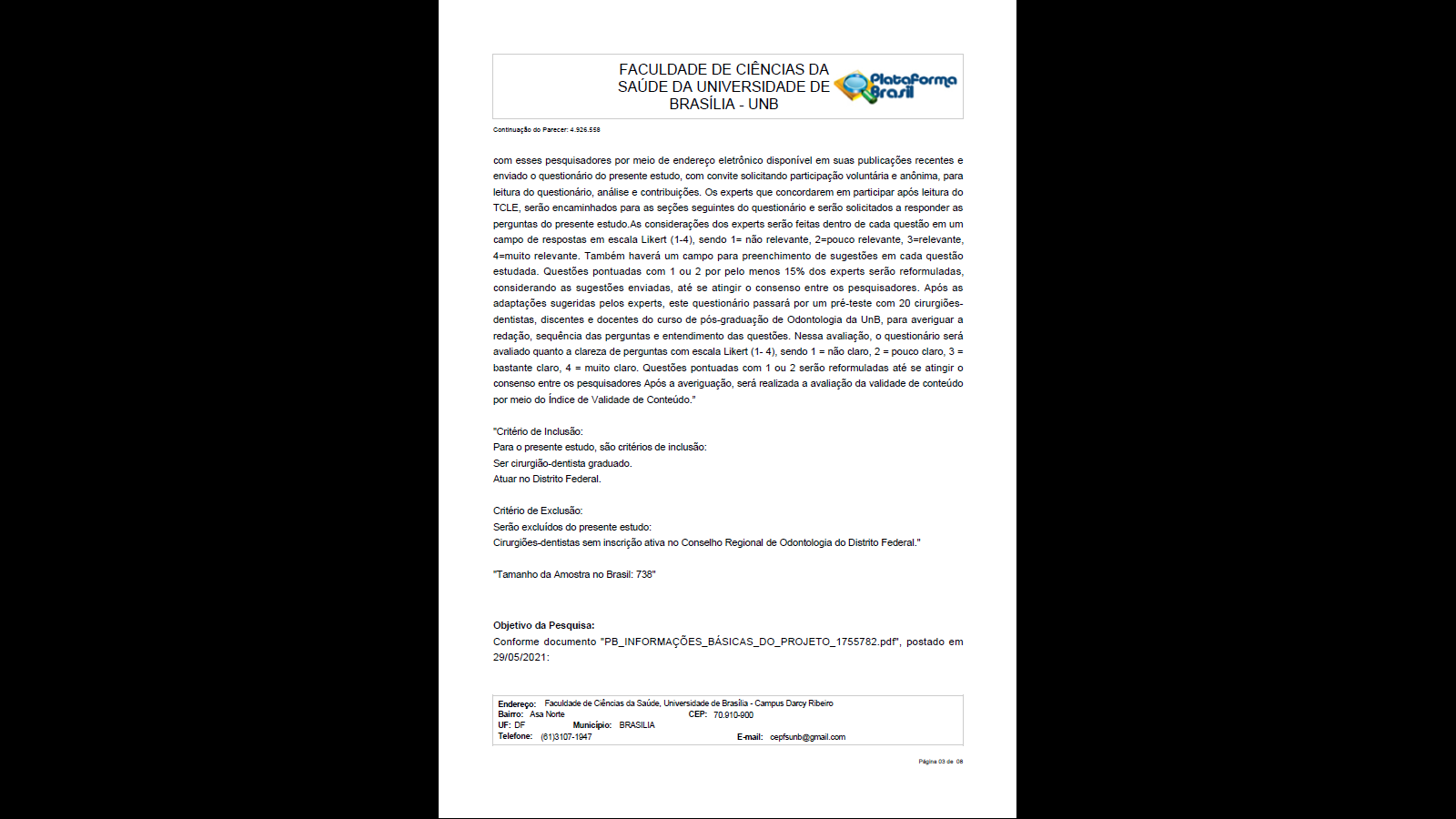


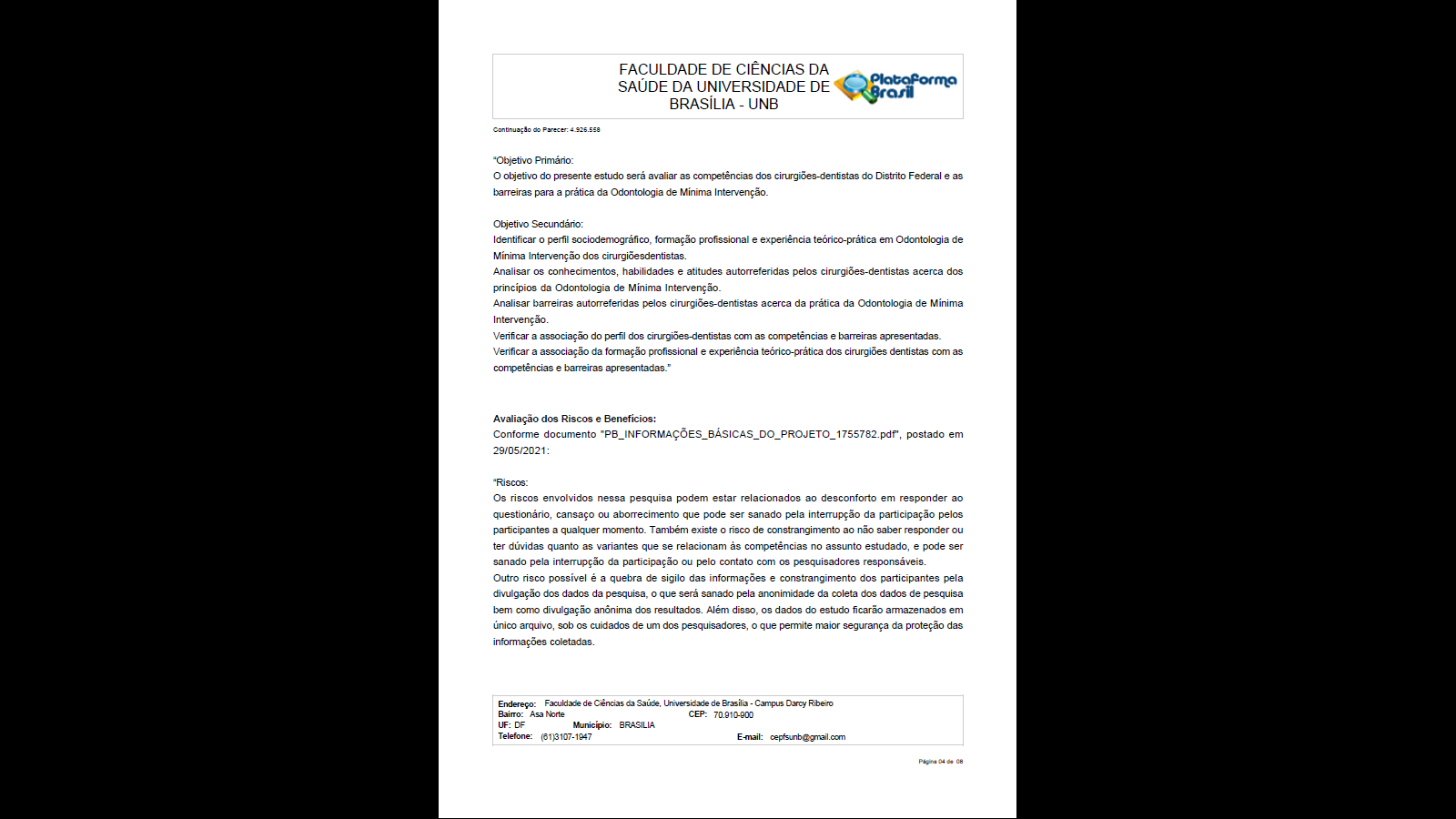


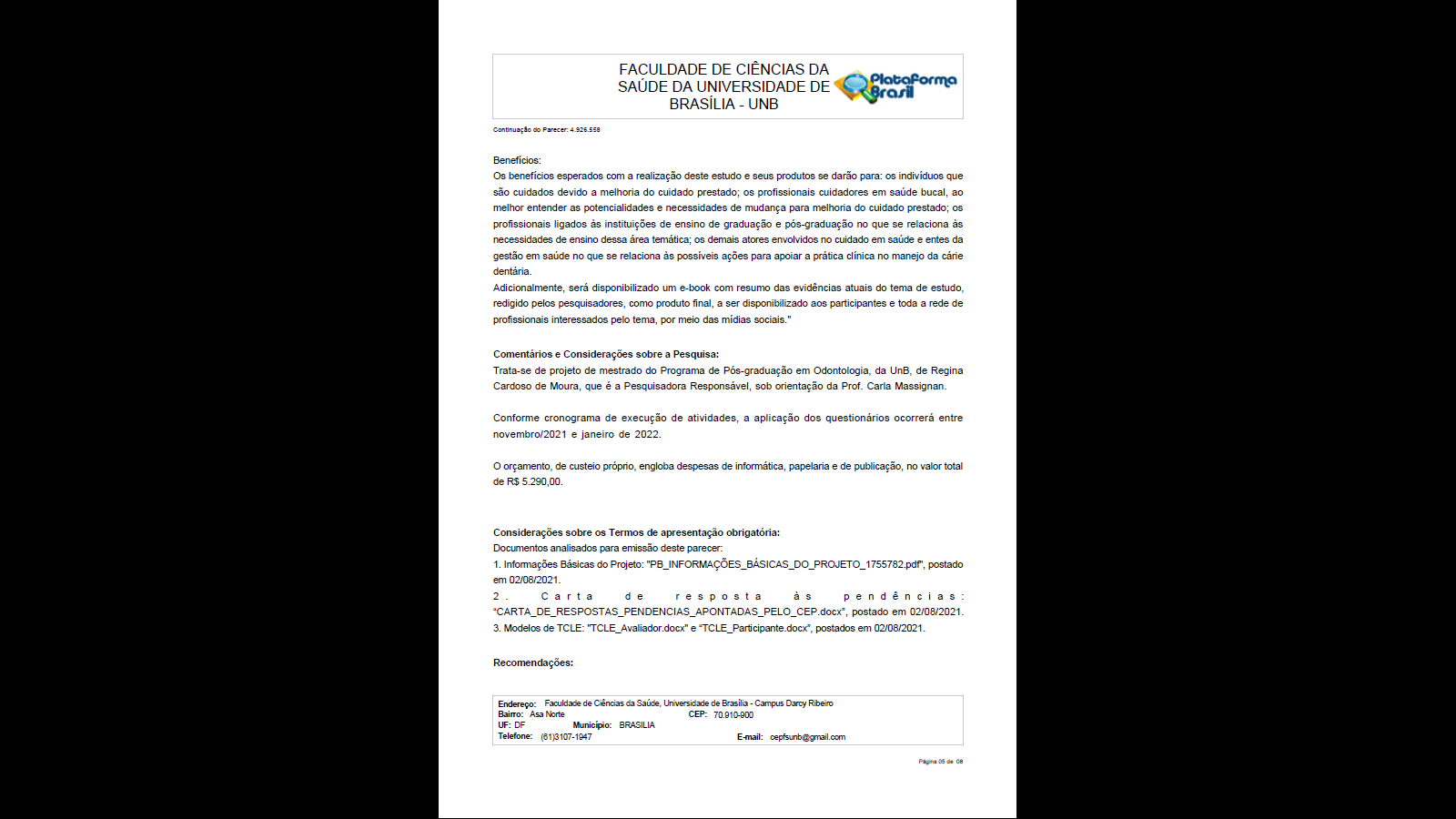


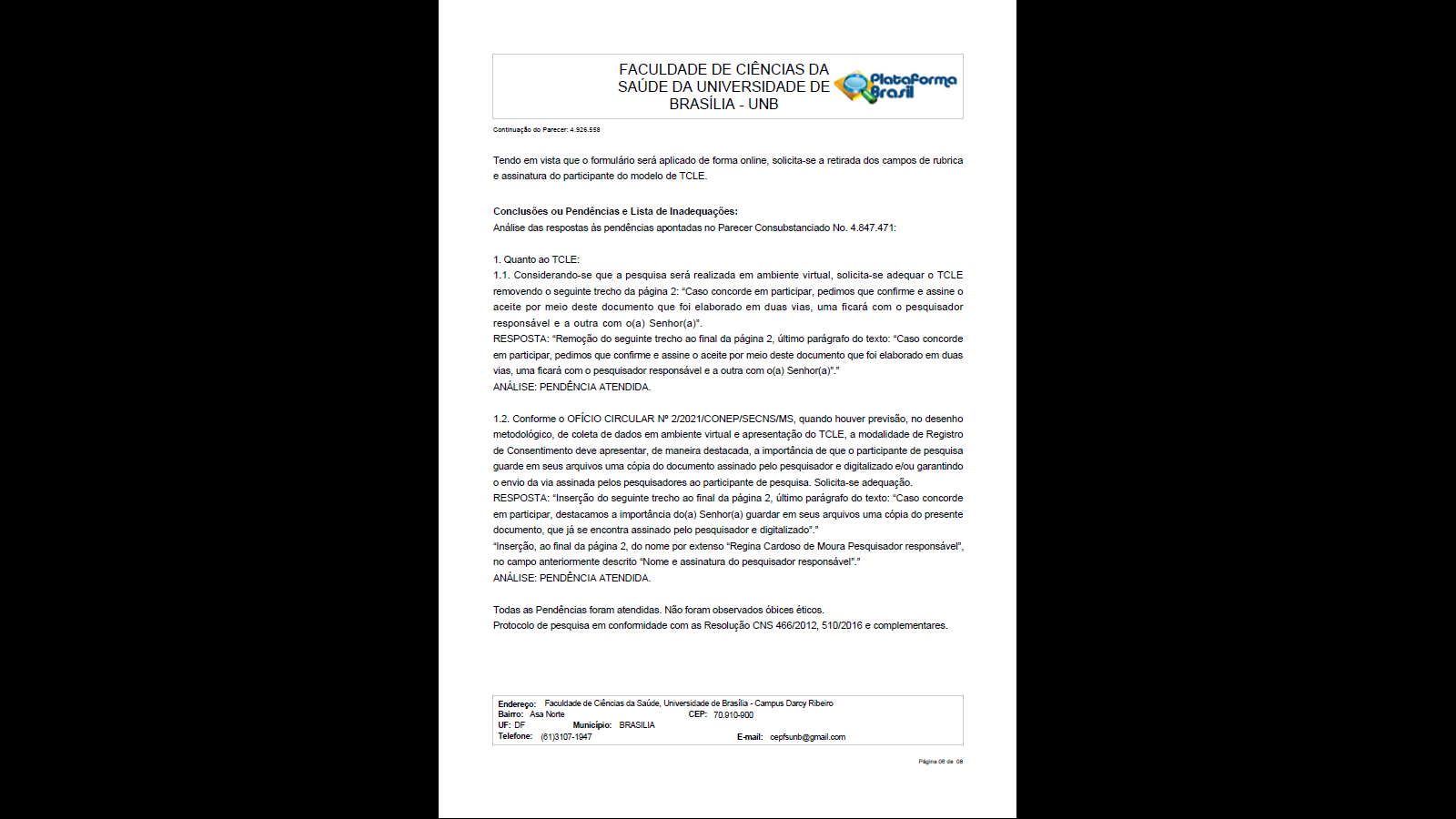


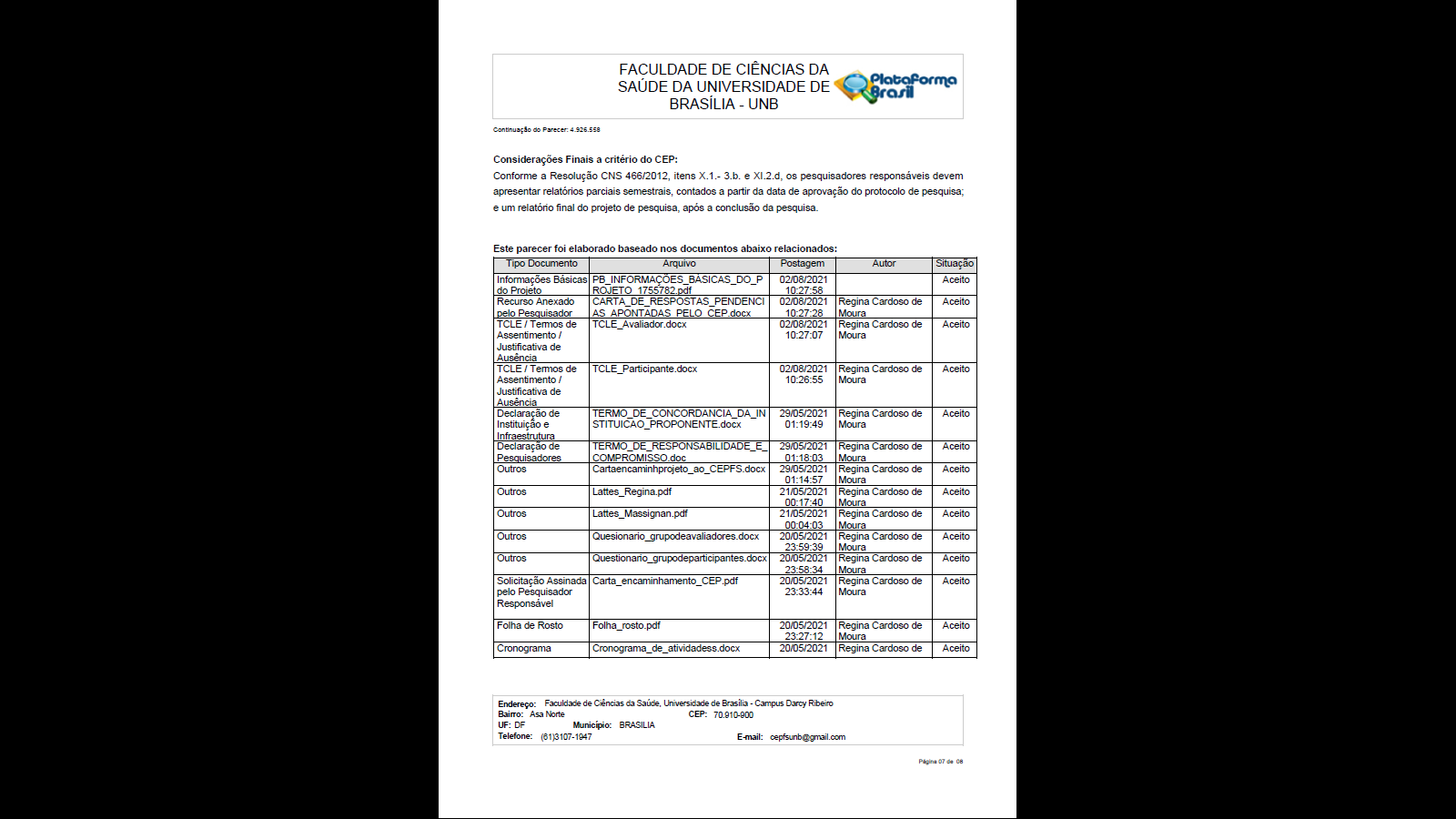


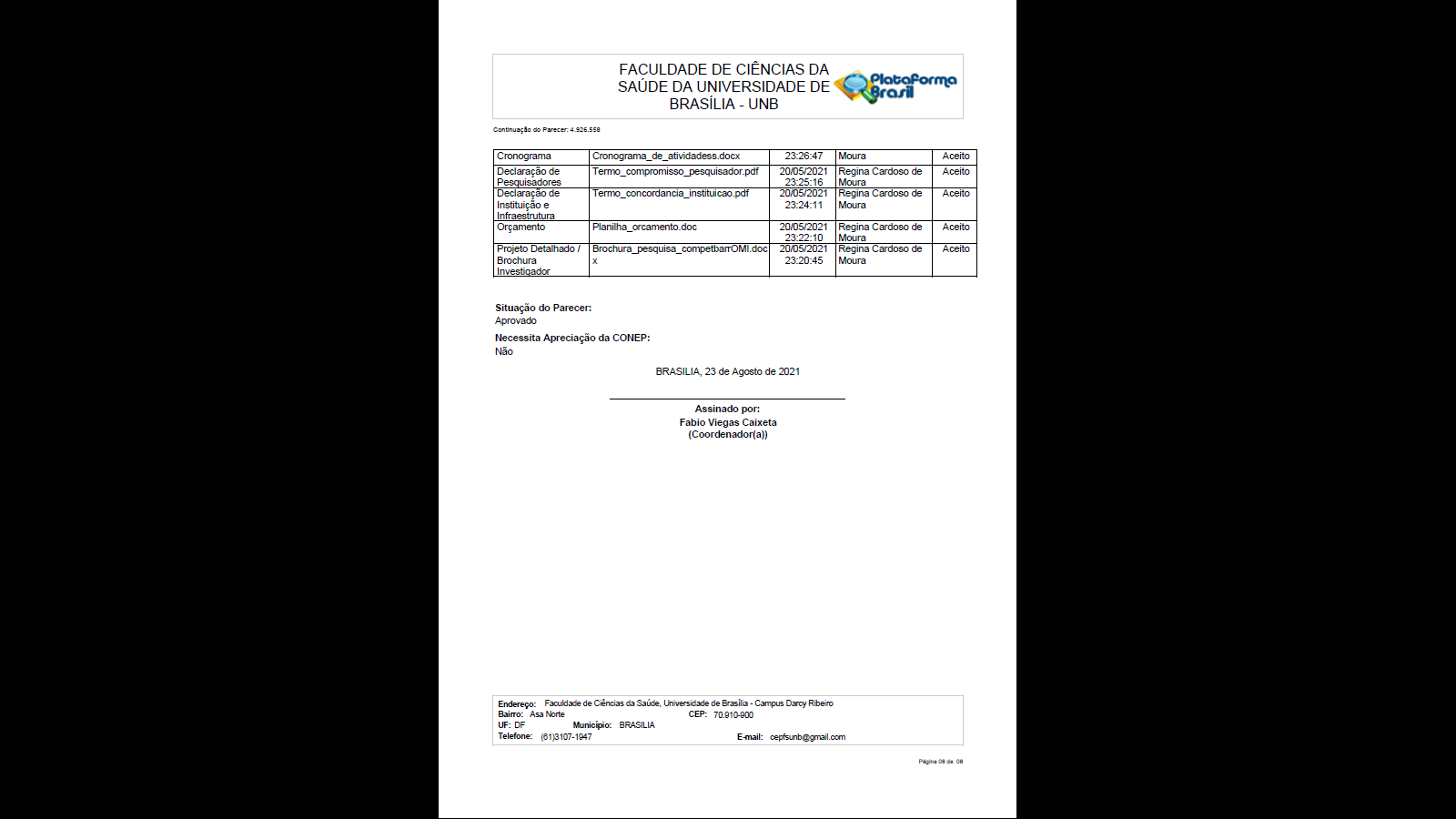


1. Artigo publicado no Journal of Dentisty, DOI: 10.1016/j.jdent.2023.104484 (Apêndice 1) [↑](#footnote-ref-1)
